# Population structure of *Salmonella enterica* Typhi in Harare, Zimbabwe (2012–19) before typhoid conjugate vaccine roll-out: a genomic epidemiology study

**DOI:** 10.1016/S2666-5247(23)00214-8

**Published:** 2023-12

**Authors:** Gaetan Thilliez, Tapfumanei Mashe, Blessmore V Chaibva, Valerie Robertson, Matt Bawn, Andrew Tarupiwa, Faustinos T Takawira, Marleen M Kock, Stanley Midzi, Lusubilo W Mwamakamba, Jorge Matheu, Agnes Juru, Robert A Kingsley, Marthie M Ehlers

**Affiliations:** a Department of Medical Microbiology, University of Pretoria, Pretoria, South Africa; b National Microbiology Reference Laboratory, Harare, Zimbabwe; c Microbes and Food Safety, Quadram Institute Bioscience, Norwich, UK; d Ministry of Health and Child Care, Harare, Zimbabwe; e Department of Laboratory Diagnostic and Investigative Sciences, University of Zimbabwe, Harare, Zimbabwe; f Department of Medical Microbiology, National Health Laboratory Service, Pretoria, South Africa; g World Health Organization, Harare, Zimbabwe; h World Health Organization Regional Office for Africa, Brazzaville, Republic of the Congo; i World Health Organization, Geneva, Switzerland; j School of Biological Sciences, University of East Anglia, Norwich, UK; k Earlham Institute, Norwich, UK; l Faculty of Biological Sciences, University of Leeds, Leeds, UK

## Abstract

**Background:**

The continued emergence of *Salmonella enterica* serovar Typhi, with ever increasing antimicrobial resistance, necessitates the use of vaccines in endemic countries. A typhoid fever outbreak in Harare, Zimbabwe, in 2018 from a multidrug resistant *S* Typhi with additional resistance to ciprofloxacin was the catalyst for the introduction of a typhoid conjugate vaccine programme. We aimed to investigate the emergence and evolution of antimicrobial resistance of endemic *S* Typhi in Zimbabwe and to determine the population structure, gene flux, and sequence polymorphisms of strains isolated before a typhoid conjugate vaccine programme to provide a baseline for future evaluation of the effect of the vaccination programme.

**Methods:**

In this genomic epidemiology study, we used short-read whole-genome sequencing of *S* Typhi isolated from clinical cases of typhoid fever in Harare, Zimbabwe, between Jan 1, 2012, and Feb 9, 2019, to determine the *S* Typhi population structure, gene flux, and sequence polymorphisms and reconstructed the evolution of antimicrobial resistance. Maximum likelihood time-scaled phylogenetic trees of Zimbabwe isolates in the context of global isolates obtained from the National Center for Biotechnology Information were constructed to infer spread and emergence of antimicrobial resistance.

**Findings:**

The population structure of *S* Typhi in Harare, Zimbabwe, from 2012 to 2019 was dominated by multidrug resistant genotype 4.3.1.1.EA1 (H58) that spread to Zimbabwe from neighbouring countries in around 2009 (95% credible interval 2008·5–2010·0). Acquisition of an IncN plasmid carrying antimicrobial resistance genes including a *qnrS* gene and a mutation in the quinolone resistance determining region of *gyrA* gene contributed to non-susceptibility and resistance to quinolone antibiotics. A minority population of antimicrobial susceptible *S* Typhi genotype 3.3.1 strains were present throughout.

**Interpretation:**

The currently dominant *S* Typhi population is genotype 4.3.1.1 that spread to Zimbabwe and acquired additional antimicrobial resistance though acquisition of a plasmid and mutation in the *gyrA* gene. This study provides a baseline population structure for future evaluation of the effect of the typhoid conjugate vaccine programme in Harare.

**Funding:**

Bill & Melinda Gates Foundation and the Biotechnology and Biological Sciences Research Council Institute Strategic Programme.

## Introduction

Typhoid fever is a systemic disease caused by *Salmonella enterica* serotype Typhi that remains an important cause of morbidity and mortality in low-resource settings.^1^ The current global burden of disease is estimated to be between 11 million and 18 million infections resulting in 135 900 deaths annually, with the majority recorded in south Asia and Africa.^2^ Before the use of antimicrobial therapy for management of typhoid fever, case fatality rates exceeded 20% due to complications such as intestinal perforation.^3^ Timely access to effective antimicrobial therapy is crucial to preventing complications such as intestinal perforation and death.^4^ Fluoroquinolones have been generally used in resource-limited countries as the primary therapy for typhoid for decades.^3^ Emergence of *S* Typhi with reduced susceptibility to fluoroquinolone and cephalosporin resistance has resulted in an increased reliance on azithromycin and carbapenems.^5^ The emergence and increase in antimicrobial resistance worldwide have focused increasing attention on the use of typhoid vaccines.^5^, ^6^

Multiple outbreaks of typhoid fever have been reported in Zimbabwe since 2009, with most beginning during the rainy season (October to March).^7^, ^8^ Standard treatment for suspected typhoid fever in Zimbabwe is primarily ciprofloxacin, followed by azithromycin or ceftriaxone in the case of treatment failure or severe disease. A typhoid outbreak associated with *S* Typhi with either reduced susceptibility (*qnrS* gene) or resistance (*gyrA* mutations) to ciprofloxacin was detected in Gweru, Zimbabwe, in September, 2018.^9^ Analysis of 29 strains of *S* Typhi isolated from an outbreak of 22 479 suspected cases of typhoid fever, of which 760 were confirmed cases, between 2012 and 2019 revealed that most isolates during this period were of genotype H58 that encoded resistance to aminoglycoside, β-lactam, phenicol, sulphonamide, tetracycline, and fluoroquinolone antibiotics.^9^ In response, an emergency reactive vaccination campaign using typhoid conjugate vaccine was implemented in Harare suburbs including Mbare, Glen View, and Kuwadzana from late February to March, 2019. 318 698 people aged 6 months to 15 years—except in Mbare where adults up to the age of 45 years were included—were vaccinated.^7^ Initial reports suggested that the vaccine provided moderate protection against typhoid fever, with an adjusted vaccine effectiveness of up to 67%.^7^

**Research in context**

**Evidence before this study**

A considerable body of literature on the genome sequence and genomic epidemiology of *Salmonella enterica* serovar Typhi in a global and local context is available. We searched PubMed using the terms “Typhi” AND “genomics” AND “epidemiology” and after excluding studies on Paratyphi we found over 100 studies using genomic or subgenomic methodologies, mostly since the year 2010. Search terms were in English using the default search parameters and no additional restrictions were applied. These studies included multiple analyses of global collections that focused on the genomics of dominant clones, antimicrobial resistance genes, and global spread. Multiple studies on specific countries in Asia, Africa, Oceania, and Latin America describe locally dominant *S* Typhi genotypes and antimicrobial resistance. A single study from Zimbabwe focused on an epidemic in Gweru City, and the relationship between genotype and antimicrobial resistance was reported from analysis of a small number of whole-genome sequences.

**Added value of this study**

The study reports the genomic epidemiology of *S* Typhi in Harare, Zimbabwe, predominantly from the year before a typhoid conjugate vaccine vaccination programme in March, 2019. Additionally, *S* Typhi genotypes endemic in Zimbabwe were placed in the phylogenetic context of globally distributed *S* Typhi focusing on evidence for the spread of genotypes in eastern and southern Africa. The data from our study provide temporal and mechanistic understanding of the emergence of antimicrobial resistance since 2012 in Zimbabwe that provided the impetus to initiate a vaccination programme. The population structure of *S* Typhi in Zimbabwe before the vaccination programme is defined for future reference.

**Implications of all the available evidence**

An understanding of the baseline population structure and genotypic variation of *S* Typhi in Harare will enable evaluation of potential changes in genotypes following the typhoid conjugate vaccine vaccination programme that might be linked to escape mutants or changes in transmission routes.

The aim of this study was to investigate the population structure of *S* Typhi isolates from urban areas of Harare to establish the history of spread of the current endemic clones in the context of the global *S* Typhi population and to understand the molecular basis and evolution of antimicrobial resistance in Zimbabwe. We focused on the population structure of *S* Typhi before the typhoid conjugate vaccine vaccination programme to provide a baseline for future evaluation of potential effects of the programme on endemic *S* Typhi in Harare, but included some isolates from Gweru City, Zimbabwe for context.

## Methods

### Study design and data sources

In this genomic epidemiology study, we retrospectively analysed suspected typhoid fever cases from Harare, Zimbabwe and compared these cases with a global collection of isolates.

Typhoid fever is a reportable disease in Zimbabwe with many of the cases from 2012 to 2019 occurring in high density populations of urban Harare situated in northeastern Zimbabwe. The Harare cases occurred between Jan 1, 2012, and Feb 9, 2019, and epidemiological data were obtained from the Harare city department health reports and clinical records. Although typhoid outbreaks were first reported in 2009, our genomic epidemiology analysis was limited to 2012–19 based on the availability of archived *S* Typhi isolates. These suspected typhoid cases were identified during routine clinical diagnosis using the standard case definition defined by the Ministry of Health and Child Care of Zimbabwe: gradual onset of steadily increasing and then persistently high fever, chills, malaise, headache, and sometimes, abdominal pain and constipation or diarrhoea. No exclusion or inclusion criteria were applied to the cases used in our analysis. 57 isolates of 128 confirmed cases of typhoid fever in Harare between Jan 1, 2018, and Dec 31, 2018, were available from archived collections at the National Microbiology Reference Laboratory, Harare, and were included in the present study. Additional whole-genome sequences of *S* Typhi isolates from a previous study in Zimbabwe were also included for context and included two isolates from 2019; both were from before the vaccination programme that occurred in Zimbabwe in February and March, 2019. Confirmation of typhoid fever was done by isolation of *S* Typhi from blood, rectal swab, or stool. Identification of suspected cases of typhoid fever and microbiological confirmation was carried out during routine diagnosis. To put the whole-genome sequence of Harare isolates into a global context we compared them with the whole-genome sequence of additional *S* Typhi isolates available in the National Center for Biotechnology Information database. These confirmed typhoid cases occurred between 1905 and Feb 9, 2019. The collection was a convenience sample of strains from Zimbabwe from stool or blood. To establish this convenience sample of *S* Typhi strains before the typhoid conjugate vaccine vaccination, data for all suspected and confirmed typhoid fever cases that were presented at health centres in Harare city between Jan 1, 2018, and Dec 31, 2018, were reviewed.

Serotyping and antimicrobial susceptibility testing of strains is described in the appendix (p 2).

Ethics approval for this study was granted by the University of Pretoria, South Africa (779/2018) and Medical Research Council of Zimbabwe (MRCZ/A/2369). Consent was not required for the use of secondary data and the study was deemed exempt by the Medical Research Council of Zimbabwe.

### Whole-genome sequencing and computational analysis

We investigated the phylogenetic relationship of *S* Typhi isolated from clinical cases of typhoid fever in Zimbabwe using whole-genome sequencing. These whole-genome sequences were either previously described^9^, ^10^ or sequenced as part of this study. Isolates were cultured on MacConkey agar for 18–20 h at 37°C. A single colony was used to inoculate LB Broth and genomic DNA was extracted from 1 mL of liquid culture using a Promega Wizard kit (Promega, Madison, WI, USA). DNA was quantified using the Qubit 3 and Nanodrop (Thermofisher, Horsham, UK). Library preparation of genomic DNA was done using the low input, transposase enabled pipeline, as described previously.^11^ Sequencing was performed using Nextseq (Illumina, San Diego, CA, USA) with a Mid Output Flowcell (NSQ 500 Mid Output KT v2; Illumina, San Diego, CA, USA). Quality control, genotyping, determination of single nucleotide polymorphisms, phylogenetic reconstruction, determination of the pangenome, ancestral state reconstruction and time dependent phylogeny, and sequence assembly are described in the appendix (pp 2–3).

### Role of the funding source

The funders of the study had no role in study design, data collection, data analysis, data interpretation, or writing of the report.

## Results

95 *S* Typhi isolates from Harare, Zimbabwe, and 1904 *S* Typhi isolates from the global collection met the inclusion criteria for this analysis. The 95 *S* Typhi isolates comprised: 28 isolated in Zimbabwe;^9^ ten travel-associated cases isolated in the UK and associated with travel to Zimbabwe, reported previously;^10^ and 57 that were sequenced as part of this study. Accession numbers for raw sequence data are shown in the appendix (pp 12–63). The 1904 global isolates were collected from 65 countries spanning six continents (Asia, Africa, North America, South America, Europe, and Australia and Oceania; appendix p 10).

**Figure 1:**
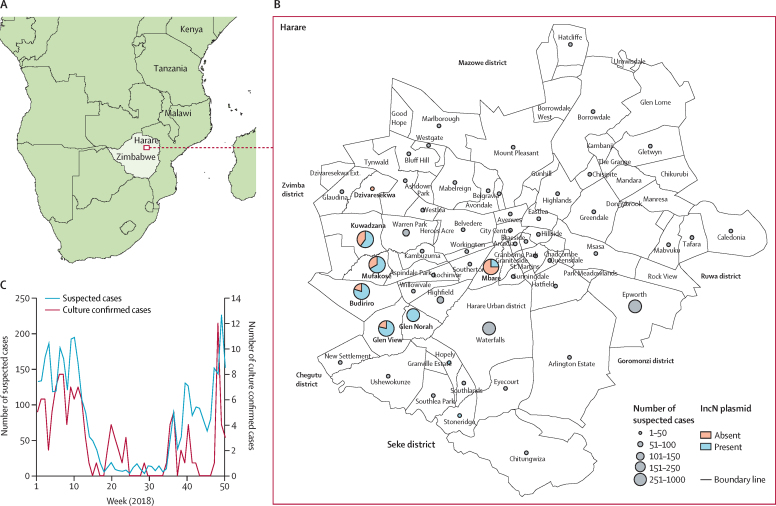
Epidemiology of typhoid fever in Harare, Zimbabwe, in 2018 (A) Map of southern Africa showing the location of Zimbabwe and Harare. (B) Geographical distribution of suspected and confirmed cases of typhoid fever in Harare in 2018. Suburbs and number of cases are indicated. (C) Number of suspected cases of typhoid fever per week during 2018.

In total, 3946 suspected typhoid fever cases were reported in Harare, Zimbabwe (figure 1), based on the standard case definition of the Ministry of Health and Child Care, Zimbabwe. Of these, 411 cases (stool specimen, rectal swab, or blood) were tested for *S* Typhi by culture and 128 (31%) were positive. The weekly number of suspected cases was high for the first 12 weeks of 2018 (ranging from n=113 to n=198) followed by a gradual decline to a relatively low level between week 18 and week 35 (ranging from n=2 to n=17; figure 1C). An increase in overall daily cases was observed from week 37 peaking in week 51 (n=221; figure 1C). Of the 3946 suspected typhoid fever cases, 128 were confirmed by culture tests and 57 were available for whole-genome sequencing and analysis.

**Figure 2:**
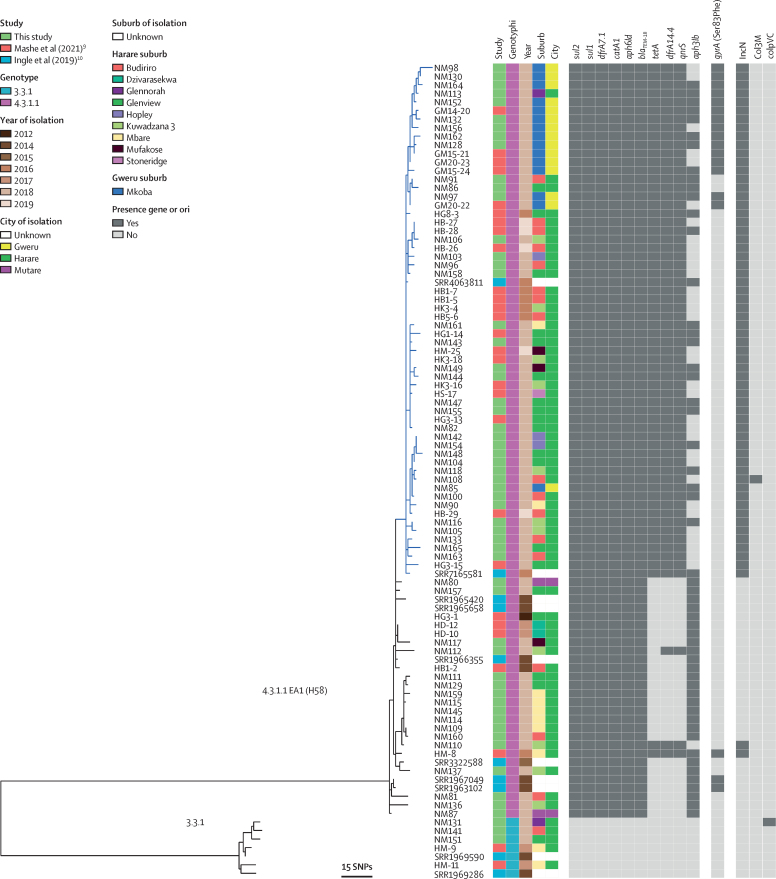
Phylogenetic relationship and genomic characteristics of 95 *S* Typhi strains isolated from Harare, Gweru, and Mutare from Jan 1, 2012, to Feb 9, 2019 A maximum likelihood phylogenetic tree constructed using nucleotide sequence variation in the shared genome of 95 *S* Typhi strains with reference to *S* Typhi CT18 whole-genome sequence assembly^12^ and rooted to the reference as an outgroup. Source of the sequence data (study), the genotype (genotyphi), year of isolation (year), and the city (location), and city suburb (suburb) are indicated. The approximate number of SNPs are indicated (bar). SNP=single-nucleotide polymorphism. *S* Typhi=*Salmonella enterica* serovar Typhi.

A maximum likelihood phylogenetic tree based on variation in the core genome sequence revealed a population structure for *S* Typhi isolated from Zimbabwe consisting of two subclades corresponding to genotypes 4.3.1.1.EA1 (H58; 88 [93%] of 95 isolates) and 3.3.1 (seven [7%]). The 69 S Typhi isolates from the 2018 outbreak were present throughout a phylogenetic tree of available data from Zimbabwe, including genotypes of both 4.3.1.1.EA1 and 3.3.1, and were closely related to isolates from 2012 to 2018 from Harare and Gweru, Zimbabwe and five isolates from 2019 (figure 2). *S* Typhi isolates of genotype 4.3.1.1.EA1 (H58) encoded between six and ten antimicrobial resistance genes, and resistance genes were not detected in any isolates of genotype 3.3.1. All isolates of 4.3.1.1.EA1 (H58) had *aph-6, bla*_TEM-1B_, *dfrA7.1, catA1, sul1,* and *sul2* genes conferring resistance to aminoglycosides, penicillin and older cephalosporins, trimethoprim, phenicols, and sulphonamides, respectively (figure 2). These genes were likely to present on the chromosome inserted between the *cyaA* and *cyaY* genes (appendix p 4), as reported previously.^13^ 62 (65%) isolates had three additional antimicrobial resistance genes, *tetA, dfrA14*, and *qnrS*, the presence of which coincided with the detection of sequence from an IncN plasmid (subtype PST3). In six of nine suburbs, the IncN plasmid was present in at least two-thirds of isolates (12 [75%] of 16 in Budiriro, 14 [78%] of 18 in Glenview, four [100%] in Hopley, seven [64%] of 11 in Kuwadzana, two [66%] of three in Mufakose, and four [100%] in Stoneridge), while notably, only two (25%) of eight of isolates from Mbare contained the plasmid (figure 1B). The presence of *tetA* coincided with resistance to tetracycline and the presence of *qnrS* coincided with intermediate resistance (non-susceptibility) to ciprofloxacin.^9^ Most strains (60 [97%] of 62) with *tetA, dfrA14*, and *qnrS* genes were present in a single distal subclade within the Zimbabwe 4.3.1.1.EA1 population structure (blue subclade in figure 2), with two isolates with this antimicrobial resistance profile situated in a more-basal rooted clade. All the isolates with the *tetA, dfrA14*, and *qnrS* genes were isolated from 2016 to 2019. The *aph3lb* gene has a complex distribution within the 4.3.1.1.EA1 (H58) population in Zimbabwe consistent with multiple acquisitions or losses. Although the *aph3lb* gene was present in all but one isolate in the basal-rooted clade, it was sporadically distributed within the distal clade containing the IncN plasmid. 18 (19%) of 95 isolates contained a mutation in the *gyrA* gene predicted to result in a Ser83Phe substitution in GyrA, known to result in increased minimum inhibitory concentration for fluoroquinolone antibiotics.^14^ GyrA Ser83Phe was present in two clusters of 13 and two isolates within the distal clade containing the IncN plasmid, and three isolates from the basally rooted clade, all of which except two also had the *qnrS* gene. No mutations in *parC* or *gyrB* were present.

To investigate the clade-specific gene content of *S* Typhi circulating in Zimbabwe we determined the pangenome of all 95 isolates from Zimbabwe (appendix p 5) by comparing all predicted protein sequences using Roary software. Three large clusters of genes co-occurred at a similar frequency and phylogenetic distribution in either all genotype 4.3.1.1.EA1 strains, a subset of 4.3.1.1.EA1, or only 3.3.1, and were designated groups 1, 2, and 3, respectively (appendix p 5). Group 1 contained genes present within a transposon containing the *aph-6, bla*_TEM-1B_, *dfrA7.1*, and *catA1* resistance genes, described previously.^15^ Group 2 contained genes consistent with a plasmid including the IncN replicon and the *tetA, dfrA14,* and *qnrS* genes. Alignment of the nucleotide sequence of group 3 genes to sequences in the National Center for Biotechnology Information database using the Basic Local Alignment Search Tool identified multiple prophage genes (appendix p 6). A putative prophage that we designated ZIM331 was most closely related to prophage P88 and consisted of 47 predicted coding sequences of which 36 had similarity to genes with functions associated with prophage functions and seven genes encoding hypothetical proteins of unknown function. A cluster of four genes were putative cargo genes and exhibited sequence similarity to *hxsDBCA*, a super-family of genes that encode proteins with diverse activity in metabolic processes.^16^

**Figure 3:**
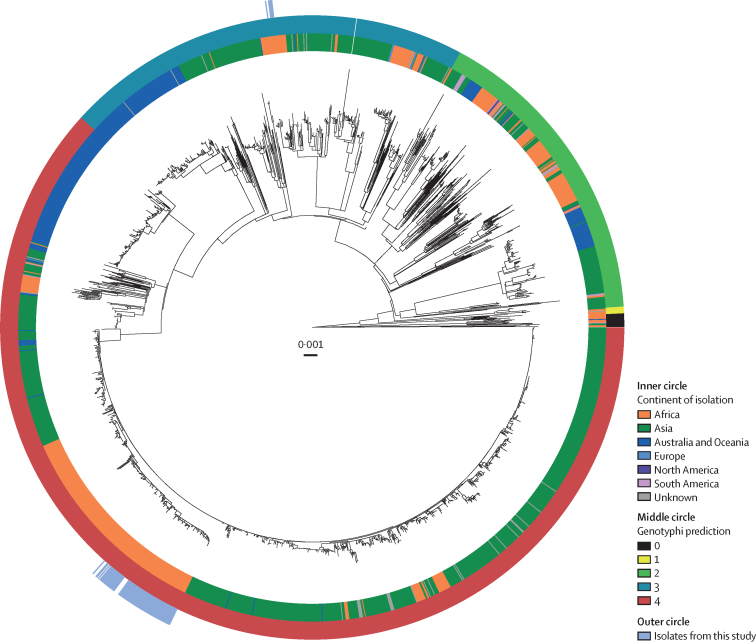
Phylogenetic relationship of 95 *S* Typhi strains isolates from Zimbabwe in the context of 1904 *S* Typhi strains isolated from globally dispersed locations Maximum likelihood phylogenetic tree constructed based on variation in shared nucleotide sequence with reference to *S* Typhi CT18 whole-genome sequence assembly.^12^ Continent of isolation (inner circle), genotype based on TyphiNET designation (middle circle), and isolates reported in this study (outer circle) are indicated. *S* Typhi=*Salmonella enterica* serovar Typhi.

To investigate the phylogenetic relationship of 95 *S* Typhi isolates from Zimbabwe in the context of the global *S* Typhi population structure we constructed a maximum likelihood tree including the 1904 *S* Typhi isolates from 65 countries, described previously (figure 3).^10^, ^13^, ^17^ A cluster of seven genotype 3.3.1 isolates were closely related to other isolates from east and southern African countries (appendix p 7). Most isolates belonged to clade 4 and particularly subclade 4.3 that includes H58 (figure 3). To further resolve the phylogenetic structure of isolates in subclade 4.3, Zimbabwe, and the global collection, a phylogenetic tree was constructed based on variation in the core genome sequence of subclade 4.3 only (appendix p 8). Genotype 4.3.1.1.EA1 isolates from Zimbabwe were present on a distal lineage within a subclade formed by isolates from east Africa and southern Africa. The ladder topology of this part of the phylogenetic tree was consistent with multiple transmission events in a southerly direction from Kenya to Tanzania, Malawi, and into Zimbabwe, followed by local spread.

**Figure 4:**
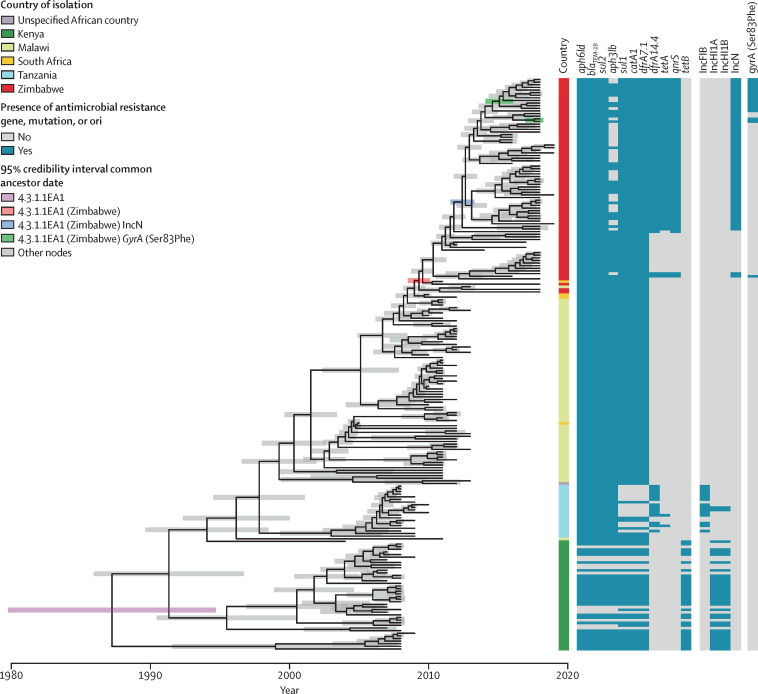
Time-scaled phylogenetic tree of genotype 4.3.1.1.EA1 *S* Typhi isolates Terminal branch lengths are constrained to date of isolation and the 95% credibility interval is indicated by shaded bar colour-coded to identify nodes corresponding to the common ancestor of the 4.3.1.1.EA1 clade, 4.3.1.1.EA1 isolated from Zimbabwe, 4.3.1.1.EA1 from Zimbabwe carrying an IncN plasmid, and 4.3.1.1.EA1 from Zimbabwe with mutations in the quinolone resistance-determining regions of *gyrA*. Country of isolation and presence or absence of antimicrobial resistance and replicon are indicated. *S* Typhi=*Salmonella enterica* serovar Typhi.

To estimate the time of key spread and evolutionary events associated with antimicrobial resistance in the emergence of the 4.3.1.1.EA1 Zimbabwe endemic clone, a subtree containing genomes from the 4.3.1.1.EA1 subclade was extracted from the genotype 4.3 clade maximum likelihood tree. Linear regression analysis indicated a strong temporal signal for the accumulation of single nucleotide polymorphisms in the 4.3.1.1.EA1 subtree that was absent if date of isolation was randomly assigned to taxa. (appendix p 9). A time-scaled tree constructed by Bayesian inference indicated that the most recent common ancestor of the 4.3.1.1.EA1 clade existed around 1987 (95% credible interval [CrI] 1977·5–1994·0; figure 4). Most of the deeply rooted isolates, that were from Kenya, had IncH1 replicon genes that correlated with the presence of *aph6ld, bla*_TEM_, *sul2, aph3lb, sul1, catA1, dfrA7.1*, and *tetB* antimicrobial resistance genes. Isolates from Tanzania, Malawi, South Africa, and Zimbabwe did not have the IncH1 replicon genes but most had *aph*6ld, *bla*_TEM_, *sul1, sul2, aph3lb, catA1*, and *dfrA7.1*, but not *tetB*. 14 of 20 isolates from Tanzania had IncFIB replicon genes and had lost *sul1, catA1, dfrA7.1*, and *tetB*, but gained a *dfrA14.4* gene. This finding was consistent with acquisition of the IncHI1 plasmid in Kenya followed by sporadic losses. Most isolates from Malawi, South Africa, and Zimbabwe had the *aph6ld, bla*_TEM_, *sul2, aph3lb, sul1, catA1, dfrA7.1*, and *tetB* antimicrobial resistance genes, despite not having the IncHI1 replicon genes that coincided in isolates from Kenya. The exception was the sporadic apparent loss of the *aph3lb* gene from 30 of the 95 isolates from Zimbabwe, an event not observed in isolates from Kenya, Tanzania, Malawi, or South Africa.

The most recent common ancestor of all *S* Typhi 4.3.1.1.EA1 isolates from Zimbabwe was estimated to have existed around 2009 (95% CrI 2008·5–2010·0), consistent with epidemiological records indicating increased outbreaks of typhoid fever from this time.^7^, ^9^ Additional evolution of antimicrobial resistance was also exclusively observed in isolates from Zimbabwe with the apparent acquisition of *dfrA14, qnrS*, and *tetA* antimicrobial resistance genes whose presence coincided with the presence of IncN replicon genes. The common ancestor of the IncN-containing isolates was around 2012 (95% CrI 2011·5–2013·3). Isolates with a *gyrA* mutation resulting in the Ser83Phe substitution associated with fluoroquinolone resistance shared a common ancestor or were isolated since around 2015 (95% CrI 2014–2016).

## Discussion

Recurrent outbreaks of typhoid fever have been recorded in Zimbabwe since 2009, with a notable outbreak of multidrug resistant *S* Typhi occurring in Harare in 2018.^9^ We found that during the period of 2012–19, strains of genotype 4.3.1.1.EA1, also known as H58, and genotype 3.3.1 were endemic. Both genotypes were likely to have been endemic throughout this time because they each formed clusters of closely related strains that were distinct from clusters from other geographical locations.^9^ The absence of 3.3.1 in the years 2012 and 2019 was probably because of the relatively small number of isolates investigated in these years (one isolate in 2012 and two in 2019). The majority of *S* Typhi isolates from Zimbabwe in this study (88 [93%] of 95) were a sublineage of the globally distributed genotype 4.3.1.1 that appear to have spread to Zimbabwe from countries in east Africa in the past two decades and were characterised by multidrug resistance encoded on a transposon.^5^ Evolution of the 4.3.1.1.EA1 epidemic clade in Zimbabwe was ongoing during the past decade with the emergence of variants with an IncN plasmid encoding antimicrobial resistance genes and mutations in the *gyrA* gene conferring clinically significant changes in susceptibility to ciprofloxacin. In contrast, genotype 3.3.1 did not have antimicrobial resistance genes and was isolated predominantly in east and southern Africa.

All the *S* Typhi genotype 4.3.1.1 isolates from Zimbabwe formed a discrete cluster within a subclade designated genotype 4.3.1.1.EA1, composed entirely of isolates from east and southern African, or isolates from travel-associated cases to this region.^18^ 4.3.1.1.EA1 was in turn rooted within isolates from southern Asia, consistent with initial introduction from southern Asia into Kenya and Tanzania and subsequent spread south into Malawi, as reported previously.^13^, ^19^ Our analyses confirmed further transmission of this clone to Zimbabwe. The strong association of isolates from each country into distinct subclades within the genotype 4.3.1.1.EA1 population structure suggests that spread resulted from a single transmission event into each country followed by local transmission of a clone. Multiple transmission events would be expected to result in a greater degree of mixing of isolates from each country in the phylogenetic tree, although additional analysis of more recent isolates from Kenya, Tanzania, and Malawi might reveal other transmission events. The common ancestor of all Zimbabwe isolates was from around 2009, marking the earliest date for introduction of genotype 4.3.1.1.EA1 into Zimbabwe. This introduction coincides with reports of renewed outbreaks in Zimbabwe from this time for unknown reasons,^20^ but might be due to the arrival of this new genotype.

To date, genotype 3.3.1 isolates have garnered little attention compared with 4.3.1.1 as they are relatively rare and susceptible to antibiotics and consequently their global spread remains to be determined. 34 isolates of genotype 3.3.1 were present in the global strain collection of 1904 whole-genome sequences used in this study; although, only 60 were available on TyphiNET out of 5327 *S* Typhi genomes (accessed Aug 1, 2022),^21^ suggesting that this genotype remains relatively rare or under-sampled globally. Nonetheless, the 34 genotype 3.3.1 isolates were from ten different countries, with 21 (62%) from east and southern Africa and ten (30%) from Asia. Notably, in common with genotype 4.3.1.1.EA1 isolates, genotype 3.3.1 isolates largely clustered based on the continent and the country of origin, consistent with international spread and subsequent domestic transmission of local clones. In contrast to genotype 4.3.1.1.EA1, the topology of genotype 3.3.1 phylogeny consisted of country-specific clades with deeply rooted branches consistent with rapid initial spread globally and little current evidence of further spread since. Additional genome sequences are needed to investigate the time and phylogeographical spread in detail. The five genotype 3.3.1 strains isolated in Harare, Zimbabwe, were from four different suburbs, consistent with a wide distribution in Harare.

The evolution of *S* Typhi strains with ever greater resistance to antimicrobials through acquisition of antimicrobial resistance genes or mutations in drug targets or efflux pumps is continuously reducing the options for therapy.^5^, ^22^ Our analysis of the evolution of the 4.3.1.1.EA1 clade highlighted a concerning trend of increased resistance in Zimbabwe. Deeply rooted 4.3.1.1.EA1 clades containing strains isolated before 2010 in Kenya were multidrug resistant due to antimicrobial resistance genes on an IncHI1 plasmid typical of genotype 4.3.1.1 isolates from south Asia.^13^ As 4.3.1.1.EA1 spread south through Tanzania, isolates appear to have lost the IncHI1 plasmid but retained the antimicrobial resistance genes, probably because of their incorporation into the chromosome as previously described.^13^ The ancestral strain that spread to Zimbabwe around 2009 was of this genotype, but within 3 years an ancestor to 59 (72%) of 82 isolates in this clade had gained an IncN plasmid containing additional genes including the *qnrS* gene conferring resistance to quinolone antibiotics. Notably, isolates from Mbare had an unusually low frequency of carrying this plasmid, highlighting the potential of genomic epidemiology to guide tailored treatment based on geographical distribution of genotypes and antimicrobial resistance profile. Cautious interpretation of these observations is advisable due to the low number of isolates from each suburb investigated. The IncN plasmid is predicted to contain around 50 genes, and it is possible that the energy cost of maintaining this plasmid might only have been favourable following the loss of the IncHI1 plasmid, which contained up to 225 genes,^23^ but this hypothesis remains to be investigated. Fluoroquinolone resistance in *S* Typhi is normally associated with mutations in the quinolone resistance determining region (QRDR) of *gyrA* and *parC*. A previous study reported that QRDR mutations emerged independently on at least 94 occasions globally but almost exclusively in south Asia.^19^ We detected at least four independent acquisitions of QRDR mutations in the *gyrA* gene. Notably, two of the mutation events that accounted for 15 of 18 isolates also contained the *qnrS* gene on the IncN plasmid, suggesting that accumulation of additional QRDR mutations might further increase fluoroquinolone. Diagnostic tests based on detection of *qnrS* or QRDR mutations might be useful in clinical management of typhoid fever in Zimbabwe.^24^

Although, to our knowledge, this study analysed the largest number of *S* Typhi isolates from Zimbabwe to date, gaps in available data limited the potential conclusions in some cases. Although the availability of genomic data of isolates from African countries over a 15-year period yielded accurate time-scaled phylogenies, the absence of contemporary isolates from Kenya, Tanzania, and Malawi limited the interpretation of recent transmission. Further analysis of contemporary isolates might help identify consequences of the vaccination programme in Zimbabwe. Furthermore, the relatively low number of isolates analysed from Zimbabwe limited the identification of genotypes present at particularly low frequency, but genotypes that were previously rare might increase in prevalence following the vaccination programme.

In response to the 2018 ciprofloxacin-resistant typhoid outbreak, Zimbabwe carried out a mass typhoid vaccination campaign from February to March, 2019, in nine suburbs of Harare with typhoid conjugate vaccine. Over 318 000 doses were administered targeting children aged between 6 months and 15 years, except in Mbare where adults aged up to 45 years were included, in affected communities. Previously, whole-genome sequencing was retrospectively used to investigate the effect on the *S* Typhi population in Thailand following a national immunisation programme in 1977 in response to a large outbreak.^25^ *S* Typhi isolates from after the immunisation programme were found to be travel-associated cases from neighbouring countries. Our study provides a detailed insight into the emergence and baseline population structure of *S* Typhi in Zimbabwe before the recent immunisation programme to enable assessment of the impact this programme on the population structure of *S* Typhi in the future. These data will enable subsequent study of the effect of the vaccination programme in Zimbabwe on transmission or emergence of escape mutants through subsequent genomic epidemiology in the coming years informing health-care policy.

## Data sharing

All sequence data are freely available from the Short Read Archive of the National Center for Biotechnology Information under accession numbers listed in the appendix (pp 12–63). Sample metadata are summarised in the appendix (pp 12–63).

## Declaration of interests

We declare no competing interests.
