# Supplementary material for: Population structure of Salmonella enterica Typhi in Harare, Zimbabwe (2012–19) before typhoid conjugate vaccine roll-out: a genomic epidemiology study
Source: Lancet Microbe. 2023 Dec;4(12):e1005–14. doi: 10.1016/S2666-5247(23)00214-8 (PMC10686908; doi:10.1016/S2666-5247(23)00214-8)
Supplement: Supplementary appendix [file mmc1.pdf]

THE LANCET
Microbe

Supplementary appendix

This appendix formed part of the original submission and has been peer reviewed. We post it as supplied by the authors.

Supplement to: Thilliez G, Mashe T, Chaibva BV, et al. Population structure of *Salmonella enterica* Typhi in Harare, Zimbabwe (2012–19) before typhoid conjugate vaccine roll-out: a genomic epidemiology study. *Lancet Microbe* 2023; published online Nov 9. https://doi.org/10.1016/S2666-5247(23)00214-8.

Contents **page**

Supplementary Methods	2
Supplementary Figure 1	4
Supplementary Figure 2	5
Supplementary Figure 3	6
Supplementary Figure 4	7
Supplementary Figure 5	8
Supplementary Figure 6	9
References	10
Supplementary Table 1	12

Supplementary Methods

Serotyping and antimicrobial susceptibility testing of strains. *S*. Typhi strains were identified using biochemical and slide agglutination using *Salmonella* Polyvalent O, Polyvalent H, O9 and dH antisera (MAST® ASSURE, Mast Group Ltd, UK) according to manufacturer's instructions. Susceptibility of 29 *S*. Typhi (2018) was determined previously (1) using disc diffusion tests with concentrations of antibiotics as follows: ampicillin (10 µg), chloramphenicol (30 µg), trimethoprim/sulfamethoxazole (1.25/23.75 µg), ceftriaxone (30 µg), azithromycin (15 µg), ciprofloxacin (5 µg) and tetracycline (30 µg) (Mast, Hampshire, UK. Zone diameters were measured and interpreted using CLSI guidelines (2). Sequence accession number and associated metadata for each isolate is provided in Supplementary Table 1.

Computational analysis of sequence data. Read quality was assessed with fastp (3) and summarized with multiqc (4). Bracken (5) was used to assess the level of contamination. Sequences with a theoretical read depth below 20x, or with less than 80% of *Salmonella* reads were excluded from further analysis.

The prediction of genotypes was carried out using the updated GenoTyphi scheme (6, 7). Maximum-likelihood phylogenetic trees were constructed from the core single-nucleotide polymorphism (SNP) alignment with reference to *S.* Typhi strain CT18 (8) using snippy version 4.3.6 as previously described (9). The root node of trees was identified by including outgroups that were removed from final version of the tree (Supplementary table 2). RAxML (version 8.2.10) (10) was used to construct maximum likelihood phylogenetic trees from the core alignment, with the generalized time-reversible model and a Gamma distribution (GTR+Γ substitution GTRGAMMA in RAxML) to model site-specific rate variation. Support for the maximum-likelihood phylogeny was assessed with rapid bootstraps based on the MRE_IGN Bootstrapping criterion. For time-scaled phylogenetic trees, the 4.3.1.1EA1 subtree was extracted using the tree_subset function from treeio (11) and dating of nodes was performed using bactDating (12) using the root from the subtree. Strict gamma, relaxed gamma, mixed gamma, arc, carc and mixedcarc clock models were tested and compared using the BactDating modelcompare function. The arc model was used for the analysis as it showed the lowest deviance information criterion (DIC) (12).

Antimicrobial resistance genes and plasmid replicons were identified using ARIBA version 2.14.6 (13) with the Plasmidfinder (version 1.2) (14) and ResFinder (version 3.1) (15) databases. Mutations in the *gyrA*, *gyrB* and *parC* chromosomal genes were detected using resistance gene identifier (RGI; version 5.1.1) (13). Genome assembly was carried out using SPAdes version 3.13.0 (16) with default parameters. The quality of the assembly was assessed with the quality assessment tool for genome assemblies QUAST version 5.0.2 (17). Assemblies larger than 5.5 MB were excluded from further analysis. Gene models and annotation was carried out using Prokka version 1.14.5 and Bandage (18, 19). For determination of pangenome and accessory genome, assembled and annotated genome sequences were used as input for Roary version 3.11.2 (20) to identify gene families and their distribution within *S*. Typhi isolates from Zimbabwe. The gene presence absence matrix was filtered to focus on genes present in at least 3 isolates (~3%) and at most 90 isolates (~95%). Regions of interest were extracted from the relevant genomes for further analysis. Prophage annotation was done using a combination of Prokka to generate the gene model and by manual curation using the output from BLASTp derived annotation of the ORF against nr database (21). Nucleotide sequence BLAST results of prophage ZIM331 against the P88 reference (NC_026014) was visualized using genoPlotR (22).

Supplementary Figures

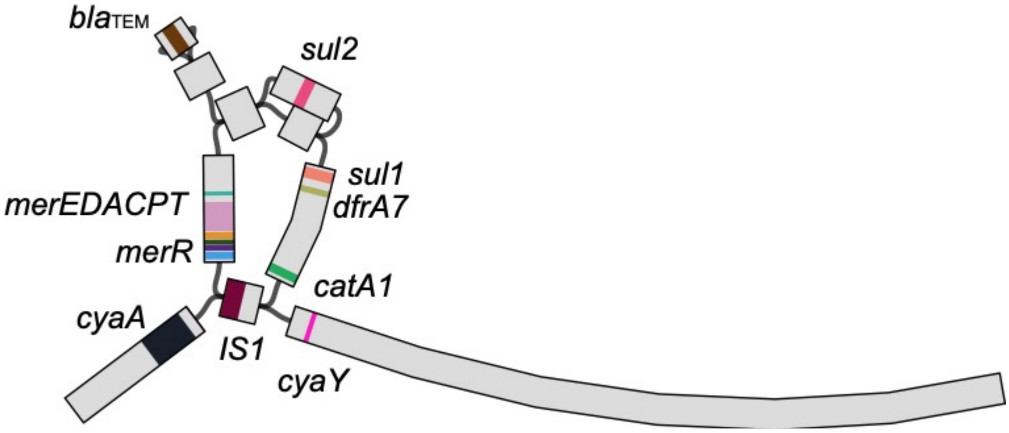

Supplementary Figure 1. Visualisation of a representative nucelotide sequence assembly graph is consistent with insertion of AMR genes in the chromsome. Contiguously assembled sequence (contigs, grey bars), connections between contigs in the graph (grey lines) and position of genes and insertion sequence (coloured bars on contigs) represent the possible assembly of short read sequence. The assembly graphs are consistent with the insertion of AMR genes *aph-6*, bla_{TEM-1B}, *dfr*A7.1, *catA1, sul1* and *sul2* and mercury resistance genes on the chromosome between the *cyaA* and *cyaY* genes.

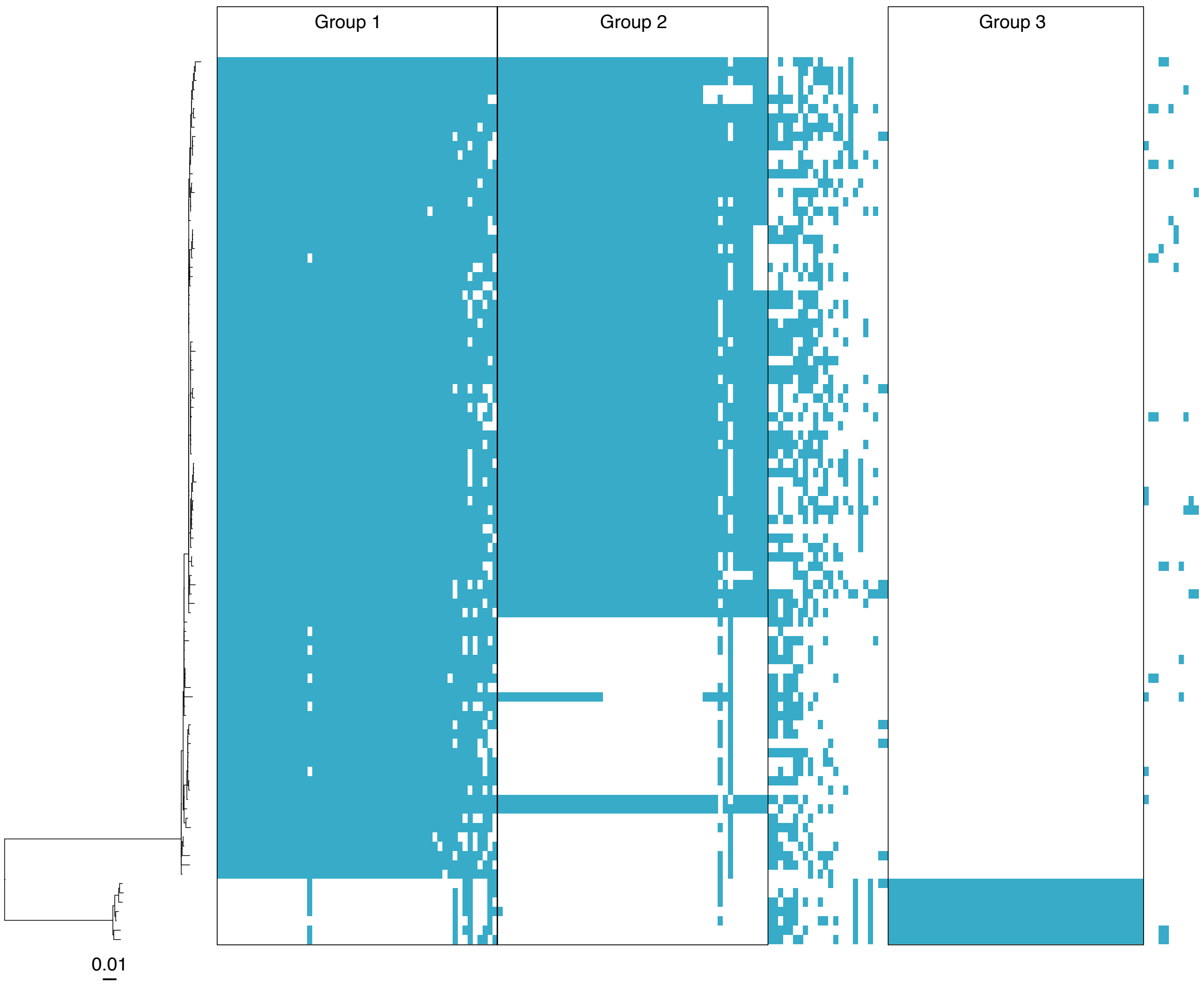

Supplementary Figure 2. Accessory genome of 95 *S*. Typhi isolates from typhoid fever cases linked to Zimbabwe. Gene families (columns) present in each genome (blue) are arranged by frequency at which they occur in 95 genomes of *S*. Typhi strains isolated in Zimbabwe (n=85) or in the UK and associated with travel to Zimbabwe (n=10). Only genes present in greater than two isolates (~3%) and less than 91 isolates (~95%) are shown. Genes with a similar frequency and phylogenetic distribution were classified as group 1, 2 and 3 that correlate with genes present on a composite transposon, IncN plasmid and prophage element, respectively.

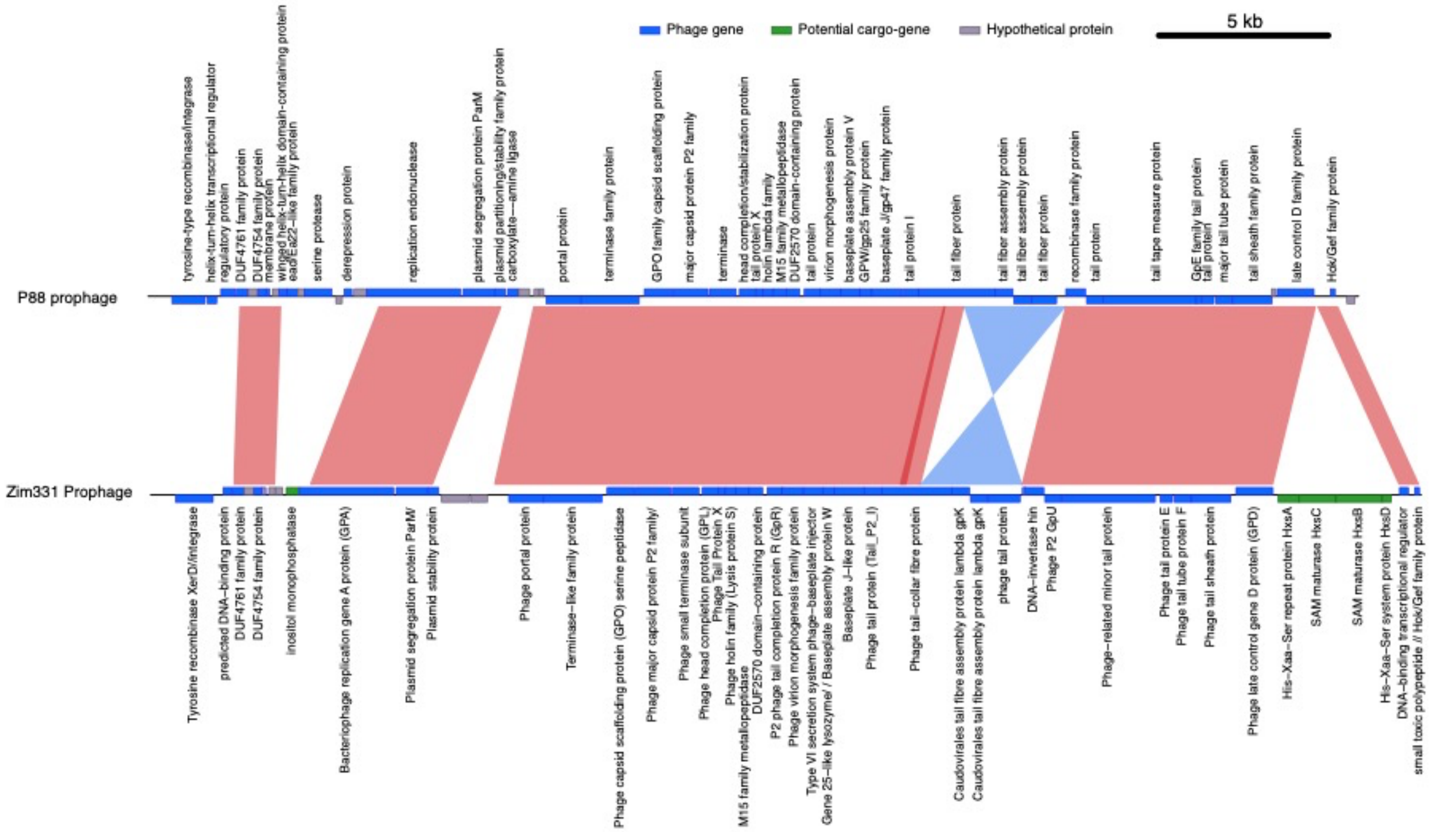

Supplementary Figure 3. Comparison of prophage Zim331 with prophage P88. A gene model predicted using prokka show genes with predicted phage functions (blue bars), potential cargo genes (green bars) and hypothetical proteins with no known function (grey bars) based on sequence alignment in the NCBI database, are indicated for prophage P88 and prophage Zim331. Predicted function for proteins encoded by genes are indicated and regions exhibiting >90% sequence identity in direct alignment (red) or reverse and complement alignment (blue) are indicated.

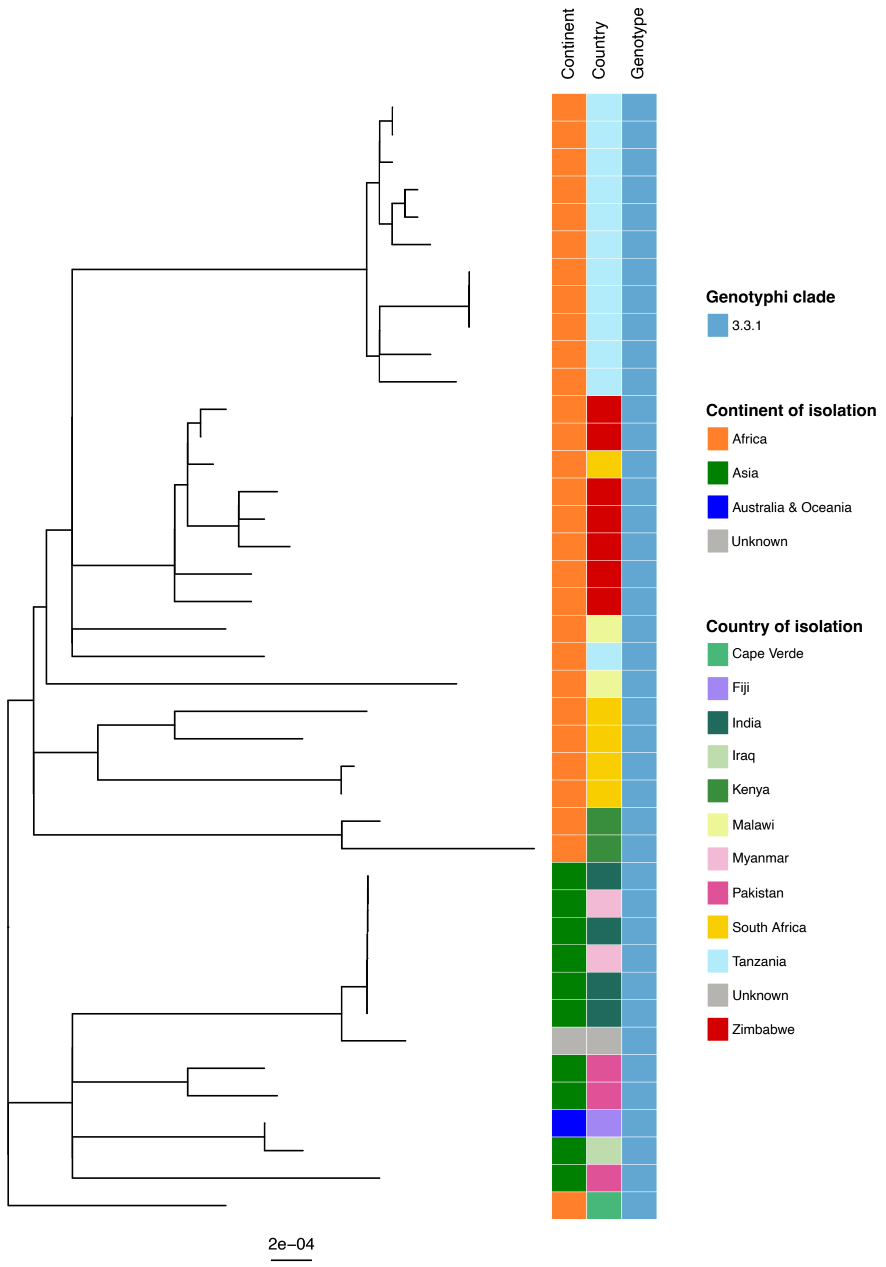

Supplemental Figure 4. Phylogeny of the subclade 3.3.1 extracted from the global tree. Continent and Country of isolation are represented on a colour coded on as indicated on the key

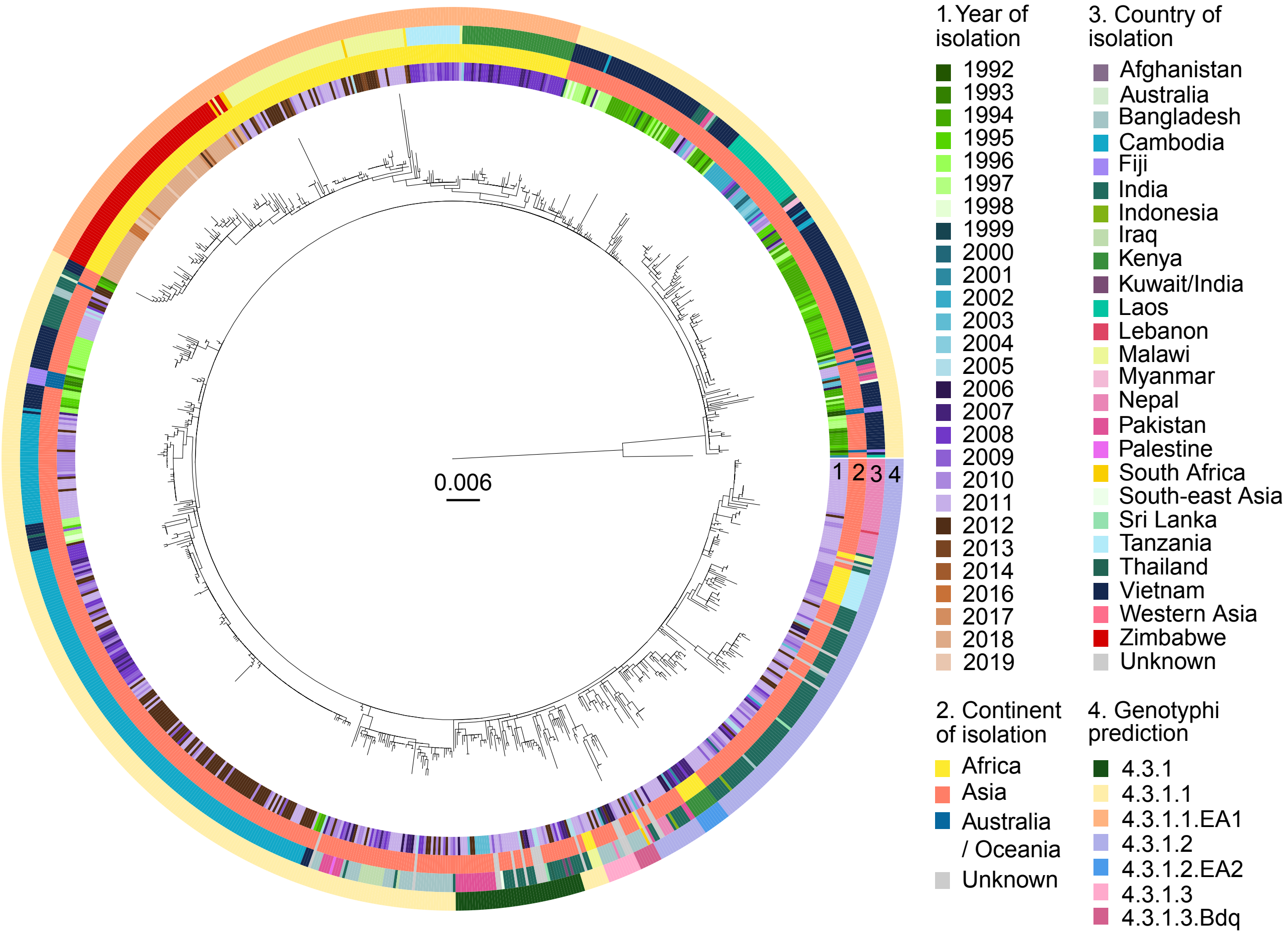

Supplementary Figure 5. Phylogenetic relationship of genotype 4.3.1 *S*. Typhi strains isolates from Zimbabwe and globally dispersed locations. Maximum likelihood phylogenetic tree constructed based on variation in shared nucleotide sequence with reference to *S* Typhi CT18 whole genome sequence assembly. Year of isolation, continent of isolation, country of isolation and genotype based on genotyphi designation are indicated in concentric circles color coded as indicated in the key (inset).

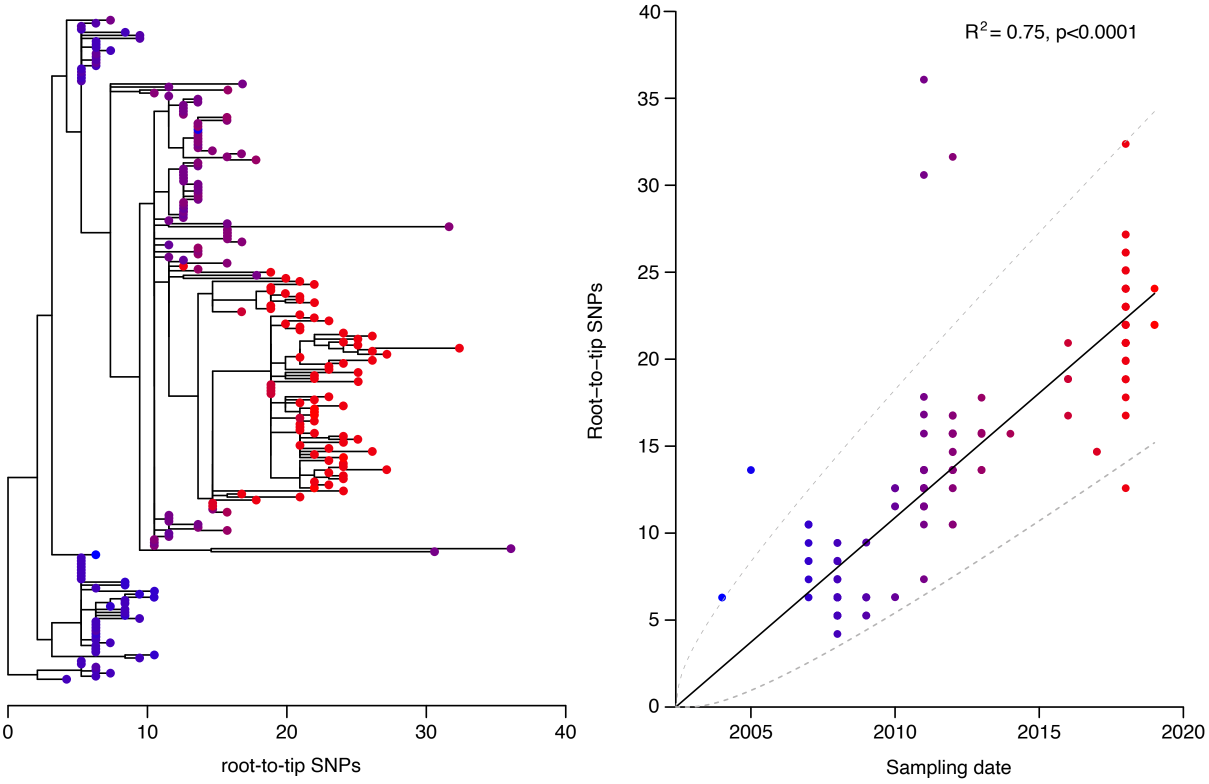

Supplemental Figure 6. Root to tip regression analysis indicating temporal signal for the accumulation of SNPs within the 4.3.1.1EA1 clade.

References

1. Mashe T, Leekitcharoenphon P, Mtapuri-Zinyowera S, Kingsley RA, Robertson V, Tarupiwa A, et al. Salmonella enterica serovar Typhi H58 clone has been endemic in Zimbabwe from 2012 to 2019. J Antimicrob Chemother. 2021;76(5):1160-7.
2. CLSI. Performance standards for antimicrobial susceptibility testing, M100, 31st ed. Clinical and Laboratory Standards Institute, Wayne, PA. 2021.
3. Chen S, Zhou Y, Chen Y, Gu J. fastp: an ultra-fast all-in-one FASTQ preprocessor. Bioinformatics. 2018;34(17):i884-i90.
4. Ewels P, Magnusson M, Lundin S, Kaller M. MultiQC: summarize analysis results for multiple tools and samples in a single report. Bioinformatics. 2016;32(19):3047-8.
5. Lu J, Breitwieser FP, Thielen P, Salzberg SL. Bracken: estimating species abundance in metagenomics data. PeerJ Computer Science. 2017;3:e104.
6. Wong VK, Baker S, Connor TR, Pickard D, Page AJ, Dave J, et al. An extended genotyping framework for Salmonella enterica serovar Typhi, the cause of human typhoid. Nat Commun. 2016;7:12827.
7. Dyson ZA, Holt KE. Five Years of GenoTyphi: Updates to the Global Salmonella Typhi Genotyping Framework. J Infect Dis. 2021;224(12 Suppl 2):S775-S80.
8. Parkhill J, Dougan G, James K, Thomson N, Pickard D, Wain J, et al. Complete genome sequence of a multiple drug resistant Salmonella enterica serovar Typhi CT18. Nature. 2001;413(6858):848-52.
9. Branchu P, Charity O, Bawn M, Thilliez G, Dallman TJ, Petrovska L, et al. SGI-4 in monophasic Salmonella Typhimurium ST34 is a novel ICE that enhances resistance to copper. Frontiers in microbiology. 2019;10:1118.
10. Stamatakis A. RAxML-VI-HPC: maximum likelihood-based phylogenetic analyses with thousands of taxa and mixed models. Bioinformatics. 2006;22(21):2688-90.
11. Yu G, Smith DK, Zhu H, Guan Y, Lam TTY. ggtree: an R package for visualization and annotation of phylogenetic trees with their covariates and other associated data. Methods in Ecology and Evolution. 2017;8(1):28-36.
12. Didelot X, Croucher NJ, Bentley SD, Harris SR, Wilson DJ. Bayesian inference of ancestral dates on bacterial phylogenetic trees. Nucleic acids research. 2018;46(22):e134-e.
13. Hunt M, Mather AE, Sánchez-Busó L, Page AJ, Parkhill J, Keane JA, et al. ARIBA: rapid antimicrobial resistance genotyping directly from sequencing reads. Microbial Genomics. 2017;3(10).
14. Carattoli A, Zankari E, García-Fernández A, Voldby Larsen M, Lund O, Villa L, et al. In Silico Detection and Typing of Plasmids using PlasmidFinder and Plasmid Multilocus Sequence Typing. Antimicrobial Agents and Chemotherapy. 2014;58(7):3895-903.
15. Zankari E, Hasman H, Cosentino S, Vestergaard M, Rasmussen S, Lund O, et al. Identification of acquired antimicrobial resistance genes. J Antimicrob Chemother. 2012;67(11):2640-4.
16. Bankevich A, Nurk S, Antipov D, Gurevich AA, Dvorkin M, Kulikov AS, et al. SPAdes: a new genome assembly algorithm and its applications to single-cell sequencing. Journal of computational biology. 2012;19(5):455-77.
17. Gurevich A, Saveliev V, Vyahhi N, Tesler G. QUAST: quality assessment tool for genome assemblies. Bioinformatics. 2013;29(8):1072-5.
18. Wick RR, Schultz MB, Zobel J, Holt KE. Bandage: interactive visualization of de novo genome assemblies. Bioinformatics. 2015;31(20):3350-2.

19. Seemann T. Prokka: rapid prokaryotic genome annotation. Bioinformatics. 2014;30(14):2068-9.
20. Page AJ, Cummins CA, Hunt M, Wong VK, Reuter S, Holden MT, et al. Roary: rapid large-scale prokaryote pan genome analysis. Bioinformatics. 2015;31(22):3691-3.
21. Camacho C, Coulouris G, Avagyan V, Ma N, Papadopoulos J, Bealer K, et al. BLAST+: architecture and applications. BMC Bioinformatics. 2009;10:421.
22. Guy L, Kultima JR, Andersson SG. genoPlotR: comparative gene and genome visualization in R. Bioinformatics. 2010;26(18):2334-5.

Supplementary Table 1. Sequence accession and associated metadata for S. Typhi strains used in this study

SRA_samp_id	Read_ID	Sample_id	Genotyphi	Age	Sex	Specimen	Year	Continent	Region	Country	Town	Suburb	reference
QIB-NM100	SRR20666403	NM100	4.3.1.1.EA1	21	M	Blood	2018	Africa	Southern Africa	Zimbabwe	Harare	Budiriro 1	This Study
QIB-NM105	SRR20666402	NM105	4.3.1.1.EA1	22	M	Stool	2018	Africa	Southern Africa	Zimbabwe	Harare	Kuwadzana 3	This Study
QIB-NM106	SRR20666391	NM106	4.3.1.1.EA1	56	F	Stool	2018	Africa	Southern Africa	Zimbabwe	Harare	Kuwadzana 3	This Study
QIB-NM108	SRR20666380	NM108	4.3.1.1.EA1	12	F	Blood	2018	Africa	Southern Africa	Zimbabwe	Harare	Budiriro 1	This Study
QIB-NM113	SRR20666369	NM113	4.3.1.1.EA1	11	M	Blood	2018	Africa	Southern Africa	Zimbabwe	Harare	Glennorah	This Study
QIB-NM114	SRR20666358	NM114	4.3.1.1.EA1	21	F	Blood	2018	Africa	Southern Africa	Zimbabwe	Harare	Mbare	This Study
QIB-NM115	SRR20666350	NM115	4.3.1.1.EA1	43	M	Stool	2018	Africa	Southern Africa	Zimbabwe	Harare	Mbare	This Study
QIB-NM116	SRR20666349	NM116	4.3.1.1.EA1	21	M	Blood	2018	Africa	Southern Africa	Zimbabwe	Harare	Kuwadzana 3	This Study
QIB-NM118	SRR20666348	NM118	4.3.1.1.EA1	33	F	Blood	2018	Africa	Southern Africa	Zimbabwe	Harare	Kuwadzana 3	This Study
QIB-NM98	SRR20666347	NM98	4.3.1.1.EA1	36	F	Blood	2018	Africa	Southern Africa	Zimbabwe	Gweru	Mkoba 18	This Study
QIB-NM80	SRR20666401	NM80	4.3.1.1.EA1	35	M	Stool	2018	Africa	Southern Africa	Zimbabwe	Mutare	Mutare	This Study
QIB-NM81	SRR20666400	NM81	4.3.1.1.EA1	17	M	Blood	2018	Africa	Southern Africa	Zimbabwe	Harare	Budiriro 1	This Study
QIB-NM82	SRR20666399	NM82	4.3.1.1.EA1	9	F	Stool	2018	Africa	Southern Africa	Zimbabwe	Harare	Glenview 1	This Study
QIB-NM85	SRR20666398	NM85	4.3.1.1.EA1	25	F	Blood	2018	Africa	Southern Africa	Zimbabwe	Gweru	Mkoba 15	This Study
QIB-NM87	SRR20666397	NM87	4.3.1.1.EA1	11	M	Stool	2018	Africa	Southern Africa	Zimbabwe	Mutare	Mutare	This Study
QIB-NM90	SRR20666396	NM90	4.3.1.1.EA1	34	M	Stool	2018	Africa	Southern Africa	Zimbabwe	Harare	Mbare	This Study
QIB-NM91	SRR20666395	NM91	4.3.1.1.EA1	70	F	Blood	2018	Africa	Southern Africa	Zimbabwe	Harare	Budiriro 1	This Study
QIB-NM96	SRR20666394	NM96	4.3.1.1.EA1	21	M	Blood	2018	Africa	Southern Africa	Zimbabwe	Harare	Budiriro 1	This Study
QIB-NM97	SRR20666393	NM97	4.3.1.1.EA1	35	M	Blood	2018	Africa	Southern Africa	Zimbabwe	Gweru	Mkoba 20	This Study
QIB-NM103	SRR20666392	NM103	4.3.1.1.EA1	16	M	Blood	2018	Africa	Southern Africa	Zimbabwe	Harare	Hopley	This Study
QIB-NM104	SRR20666390	NM104	4.3.1.1.EA1	45	F	Blood	2018	Africa	Southern Africa	Zimbabwe	Harare	Glenview 1	This Study
QIB-NM109	SRR20666389	NM109	4.3.1.1.EA1	21	M	Blood	2018	Africa	Southern Africa	Zimbabwe	Harare	Mbare	This Study
QIB-NM110	SRR20666388	NM110	4.3.1.1.EA1	11	F	Stool	2018	Africa	Southern Africa	Zimbabwe	Harare	Kuwadzana 3	This Study
QIB-NM111	SRR20666387	NM111	4.3.1.1.EA1	22	M	Stool	2018	Africa	Southern Africa	Zimbabwe	Harare	Glenview 1	This Study
QIB-NM112	SRR20666386	NM112	4.3.1.1.EA1	40	F	Blood	2018	Africa	Southern Africa	Zimbabwe	Harare	Kuwadzana 3	This Study
QIB-NM117	SRR20666385	NM117	4.3.1.1.EA1	55	F	Blood	2018	Africa	Southern Africa	Zimbabwe	Harare	Mufakose	This Study
QIB-NM128	SRR20666384	NM128	4.3.1.1.EA1	17	M	Stool	2018	Africa	Southern Africa	Zimbabwe	Gweru	Mkoba 14	This Study
QIB-NM129	SRR20666383	NM129	4.3.1.1.EA1	28	F	Blood	2018	Africa	Southern Africa	Zimbabwe	Harare	Glenview 1	This Study
QIB-NM130	SRR20666382	NM130	4.3.1.1.EA1	21	M	Blood	2018	Africa	Southern Africa	Zimbabwe	Gweru	Mkoba 15	This Study
QIB-NM132	SRR20666381	NM132	4.3.1.1.EA1	26	F	Blood	2018	Africa	Southern Africa	Zimbabwe	Gweru	Mkoba 13	This Study
QIB-NM133	SRR20666379	NM133	4.3.1.1.EA1	31	M	Blood	2018	Africa	Southern Africa	Zimbabwe	Harare	Budiriro 1	This Study
QIB-NM136	SRR20666378	NM136	4.3.1.1.EA1	29	F	Blood	2018	Africa	Southern Africa	Zimbabwe	Harare	Kuwadzana 3	This Study
QIB-NM137	SRR20666377	NM137	4.3.1.1.EA1	27	M	Blood	2018	Africa	Southern Africa	Zimbabwe	Harare	Kuwadzana 3	This Study
QIB-NM142	SRR20666376	NM142	4.3.1.1.EA1	23	F	Blood	2018	Africa	Southern Africa	Zimbabwe	Harare	Hopley	This Study
QIB-NM143	SRR20666375	NM143	4.3.1.1.EA1	12	M	Blood	2018	Africa	Southern Africa	Zimbabwe	Harare	Glenview 8	This Study
QIB-NM144	SRR20666374	NM144	4.3.1.1.EA1	36	F	Stool	2018	Africa	Southern Africa	Zimbabwe	Harare	Glenview 1	This Study
QIB-NM145	SRR20666373	NM145	4.3.1.1.EA1	21	M	Blood	2018	Africa	Southern Africa	Zimbabwe	Harare	Mbare	This Study
QIB-NM147	SRR20666372	NM147	4.3.1.1.EA1	49	F	Blood	2018	Africa	Southern Africa	Zimbabwe	Harare	Glenview 1	This Study
QIB-NM148	SRR20666371	NM148	4.3.1.1.EA1	37	F	Blood	2018	Africa	Southern Africa	Zimbabwe	Harare	Glenview 1	This Study
QIB-NM149	SRR20666370	NM149	4.3.1.1.EA1	39	F	Blood	2018	Africa	Southern Africa	Zimbabwe	Harare	Mufakose	This Study
QIB-NM152	SRR20666368	NM152	4.3.1.1.EA1	27	M	Stool	2018	Africa	Southern Africa	Zimbabwe	Gweru	Mkoba 20	This Study

QIB-NM154	SRR20666367	NM154	4.3.1.1.EA1	20	F	Blood	2018	Africa	Southern Africa	Zimbabwe	Harare	Hopley	This Study
QIB-NM155	SRR20666366	NM155	4.3.1.1.EA1	19	M	Blood	2018	Africa	Southern Africa	Zimbabwe	Harare	Glenview 1	This Study
QIB-NM156	SRR20666365	NM156	4.3.1.1.EA1	14	M	Stool	2018	Africa	Southern Africa	Zimbabwe	Gweru	Mkoba 20	This Study
QIB-NM157	SRR20666364	NM157	4.3.1.1.EA1	14	M	Stool	2018	Africa	Southern Africa	Zimbabwe	Harare	Glenview 2	This Study
QIB-NM158	SRR20666363	NM158	4.3.1.1.EA1	36	F	Blood	2018	Africa	Southern Africa	Zimbabwe	Harare	Glenview 1	This Study
QIB-NM159	SRR20666362	NM159	4.3.1.1.EA1	59	F	Stool	2018	Africa	Southern Africa	Zimbabwe	Harare	Mbare	This Study
QIB-NM160	SRR20666361	NM160	4.3.1.1.EA1	38	M	Stool	2018	Africa	Southern Africa	Zimbabwe	Harare	Budiriro 1	This Study
QIB-NM161	SRR20666360	NM161	4.3.1.1.EA1	50	F	Blood	2018	Africa	Southern Africa	Zimbabwe	Harare	Mbare	This Study
QIB-NM162	SRR20666359	NM162	4.3.1.1.EA1	16	M	Blood	2018	Africa	Southern Africa	Zimbabwe	Gweru	Mkoba 14	This Study
QIB-NM163	SRR20666357	NM163	4.3.1.1.EA1	25	M	Blood	2018	Africa	Southern Africa	Zimbabwe	Harare	Budiriro 1	This Study
QIB-NM164	SRR20666356	NM164	4.3.1.1.EA1	44	M	Blood	2018	Africa	Southern Africa	Zimbabwe	Gweru	Mkoba 15	This Study
QIB-NM165	SRR20666355	NM165	4.3.1.1.EA1	26	M	Blood	2018	Africa	Southern Africa	Zimbabwe	Harare	Glenview 1	This Study
QIB-NM86	SRR20666354	NM86	4.3.1.1.EA1	22	M	Blood	2018	Africa	Southern Africa	Zimbabwe	Harare	Glenview 1	This Study
QIB-NM131	SRR20666353	NM131	3.3.1	56	F	Blood	2018	Africa	Southern Africa	Zimbabwe	Harare	Glennorah	This Study
QIB-NM141	SRR20666352	NM141	3.3.1	29	M	Blood	2018	Africa	Southern Africa	Zimbabwe	Harare	Budiriro 1	This Study
QIB-NM151	SRR20666351	NM151	3.3.1	30	F	Stool	2018	Africa	Southern Africa	Zimbabwe	Harare	Glenview 1	This Study
HG3-1	ERR4870975	HG3-1	4.3.1.1.EA1	34	F	Stool	2012	Africa	Southern Africa	Zimbabwe	Harare	Glenview 3	Mashe et al 2021
HB1-2	ERR4870976	HB1-2	4.3.1.1.EA1	8	M	Blood	2014	Africa	Southern Africa	Zimbabwe	Harare	Budiriro 1	Mashe et al 2021
HG8-3	ERR4870977	HG8-3	4.3.1.1.EA1	26	F	Blood	2016	Africa	Southern Africa	Zimbabwe	Harare	Glenview 8	Mashe et al 2021
HK3-4	ERR4870978	HK3-4	4.3.1.1.EA1	7	F	Blood	2016	Africa	Southern Africa	Zimbabwe	Harare	Kuwadzana 3	Mashe et al 2021
HB1-5	ERR4870979	HB1-5	4.3.1.1.EA1	3	M	Blood	2016	Africa	Southern Africa	Zimbabwe	Harare	Budiriro 1	Mashe et al 2021
HB5-6	ERR4870980	HB5-6	4.3.1.1.EA1	21	M	Blood	2016	Africa	Southern Africa	Zimbabwe	Harare	Budiriro 5	Mashe et al 2021
HB1-7	ERR4870981	HB1-7	4.3.1.1.EA1	21	M	Blood	2016	Africa	Southern Africa	Zimbabwe	Harare	Budiriro 1	Mashe et al 2021
HM-8	ERR4870982	HM-8	4.3.1.1.EA1	5	F	Blood	2016	Africa	Southern Africa	Zimbabwe	Harare	Mbare	Mashe et al 2021
HM-9	ERR4870983	HM-9	3.3.1	20	M	Stool	2017	Africa	Southern Africa	Zimbabwe	Harare	Dzivarasekwa	Mashe et al 2021
HD-10	ERR4870984	HD-10	4.3.1.1.EA1	7	F	Blood	2017	Africa	Southern Africa	Zimbabwe	Harare	Dzivarasekwa	Mashe et al 2021
HM-11	ERR4870985	HM-11	3.3.1	10	F	Blood	2017	Africa	Southern Africa	Zimbabwe	Harare	Glenview 3	Mashe et al 2021
HD-12	ERR4870986	HD-12	4.3.1.1.EA1	13	F	Blood	2017	Africa	Southern Africa	Zimbabwe	Harare	Glenview 1	Mashe et al 2021
HG3-13	ERR4870987	HG3-13	4.3.1.1.EA1	16	F	Stool	2018	Africa	Southern Africa	Zimbabwe	Harare	Glenview 3	Mashe et al 2021
HG1-14	ERR4870988	HG1-14	4.3.1.1.EA1	6	M	Stool	2018	Africa	Southern Africa	Zimbabwe	Harare	Kuwadzana 3	Mashe et al 2021
HG3-15	ERR4870989	HG3-15	4.3.1.1.EA1	18	M	Blood	2018	Africa	Southern Africa	Zimbabwe	Harare	Stoneridge	Mashe et al 2021
HK3-16	ERR4870990	HK3-16	4.3.1.1.EA1	19	M	Blood	2018	Africa	Southern Africa	Zimbabwe	Harare	Kuwadzana 3	Mashe et al 2021
HS-17	ERR4870991	HS-17	4.3.1.1.EA1	64	F	Blood	2018	Africa	Southern Africa	Zimbabwe	Harare	Hopley	Mashe et al 2021
HK3-18	ERR4870992	HK3-18	4.3.1.1.EA1	23	F	Blood	2018	Africa	Southern Africa	Zimbabwe	Harare	Mkoba 14	Mashe et al 2021

HH-19	ERR4870993	HH-19	4.3.1.1.EA1	21	M	Stool	2018	Africa	Southern Africa	Zimbabwe	Harare	Mkoba 15	Mashe et al 2021
GM14-20	ERR4870994	GM14-20	4.3.1.1.EA1	9	F	Blood	2018	Africa	Southern Africa	Zimbabwe	Gweru	Mkoba 20	Mashe et al 2021
GM15-21	ERR4870995	GM15-21	4.3.1.1.EA1	48	M	Blood	2018	Africa	Southern Africa	Zimbabwe	Gweru	Mkoba 20	Mashe et al 2021
GM20-22	ERR4870996	GM20-22	4.3.1.1.EA1	44	M	Blood	2018	Africa	Southern Africa	Zimbabwe	Gweru	Mkoba 15	Mashe et al 2021
GM20-23	ERR4870997	GM20-23	4.3.1.1.EA1	17	M	Blood	2018	Africa	Southern Africa	Zimbabwe	Gweru	Mufakose	Mashe et al 2021
GM15-24	ERR4870998	GM15-24	4.3.1.1.EA1	34	M	Blood	2018	Africa	Southern Africa	Zimbabwe	Gweru	Budiriro	Mashe et al 2021
HM-25	ERR4870999	HM-25	4.3.1.1.EA1	15	F	Blood	2019	Africa	Southern Africa	Zimbabwe	Harare	Budiriro	Mashe et al 2021
HB-26	ERR4871000	HB-26	4.3.1.1.EA1	13	F	Blood	2019	Africa	Southern Africa	Zimbabwe	Harare	Budiriro	Mashe et al 2021
HB-27	ERR4871001	HB-27	4.3.1.1.EA1	13	F	Blood	2019	Africa	Southern Africa	Zimbabwe	Harare	Budiriro	Mashe et al 2021
HB-28	ERR4871002	HB-28	4.3.1.1.EA1	6	F	Blood	2019	Africa	Southern Africa	Zimbabwe	Harare	Mbare	Mashe et al 2021
HB-29	ERR4871003	HB-29	4.3.1.1.EA1	14	M	Blood	2019	Africa	Southern Africa	Zimbabwe	Harare	Mbare	Mashe et al 2021
unknown	SRR1963102	SRR1963102	4.3.1.1.EA1	unknown	unknown	unknown	2014	Africa (UK travel)	Southern Africa (UK travel)	Zimbabwe (UK travel)	unknown	unknown	Ingle et al 2019
unknown	SRR1965420	SRR1965420	4.3.1.1.EA1	unknown	unknown	unknown	2014	Africa (UK travel)	Southern Africa (UK travel)	Zimbabwe (UK travel)	unknown	unknown	Ingle et al 2019
unknown	SRR1965658	SRR1965658	4.3.1.1.EA1	unknown	unknown	unknown	2014	Africa (UK travel)	Southern Africa (UK travel)	Zimbabwe (UK travel)	unknown	unknown	Ingle et al 2019
unknown	SRR1966355	SRR1966355	4.3.1.1.EA1	unknown	unknown	unknown	2014	Africa (UK travel)	Southern Africa (UK travel)	Zimbabwe (UK travel)	unknown	unknown	Ingle et al 2019
unknown	SRR1967049	SRR1967049	4.3.1.1.EA1	unknown	unknown	unknown	2014	Africa (UK travel)	Southern Africa (UK travel)	Zimbabwe (UK travel)	unknown	unknown	Ingle et al 2019
unknown	SRR1969286	SRR1969286	3.3.1	unknown	unknown	unknown	2014	Africa (UK travel)	Southern Africa (UK travel)	Zimbabwe (UK travel)	unknown	unknown	Ingle et al 2019
unknown	SRR1969590	SRR1969590	3.3.1	unknown	unknown	unknown	2014	Africa (UK travel)	Southern Africa (UK travel)	Zimbabwe (UK travel)	unknown	unknown	Ingle et al 2019
unknown	SRR3322588	SRR3322588	4.3.1.1.EA1	unknown	unknown	unknown	2015	Africa (UK travel)	Southern Africa (UK travel)	Zimbabwe (UK travel)	unknown	unknown	Ingle et al 2019
unknown	SRR4063811	SRR4063811	4.3.1.1.EA1	unknown	unknown	unknown	2016	Africa (UK travel)	Southern Africa (UK travel)	Zimbabwe (UK travel)	unknown	unknown	Ingle et al 2019
unknown	SRR7165581	SRR7165581	4.3.1.1.EA1	unknown	unknown	unknown	2016	Africa (UK travel)	Southern Africa (UK travel)	Zimbabwe (UK travel)	unknown	unknown	Ingle et al 2019
H05118260	ERR1017039	N/A	4.3.1.1	unknown	unknown	Blood	2005	Unknown	Unknown	Unknown	unknown	unknown	Wong et al, 2016
H05196407	ERR1017040	N/A	4.3.1	unknown	unknown	Blood	2005	Unknown	Unknown	Unknown	unknown	unknown	Wong et al, 2016
H05272442	ERR1017041	N/A	3.1.1	unknown	unknown	Blood	2005	Unknown	Unknown	Unknown	unknown	unknown	Wong et al, 2016
H05406403	ERR1017042	N/A	4.3.1.1	unknown	unknown	Blood	2005	Unknown	Unknown	Unknown	unknown	unknown	Wong et al, 2016
H06016481	ERR1017045	N/A	4.3.1.1	unknown	unknown	Blood	2005	Unknown	Unknown	Unknown	unknown	unknown	Wong et al, 2016
H06156550	ERR1017048	N/A	3.0.1	unknown	unknown	Blood	2006	Unknown	Unknown	Unknown	unknown	unknown	Wong et al, 2016
H06384614	ERR1017052	N/A	4.3.1	unknown	unknown	Blood	2006	Asia	South Asia	Pakistan	unknown	unknown	Wong et al, 2016
H06394364	ERR1017054	N/A	4.3.1.1	unknown	unknown	Blood	2006	Asia	South Asia	Bangladesh	unknown	unknown	Wong et al, 2016
H06414501	ERR1017055	N/A	3.3.2	unknown	unknown	Blood	2006	Asia	South Asia	Bangladesh	unknown	unknown	Wong et al, 2016
H06434426	ERR1017060	N/A	2.0.2	unknown	unknown	Blood	2006	Unknown	Unknown	Unknown	unknown	unknown	Wong et al, 2016
H07014193	ERR1017063	N/A	2.2	unknown	unknown	Blood	2006	Asia	South Asia	Pakistan	unknown	unknown	Wong et al, 2016

H07044209	ERR1017064	N/A	4.3.1.2	unknown	unknown	Blood	2007	Asia	South Asia	India	unknown	unknown	Wong et al, 2016
H07288307	ERR1017067	N/A	4.3.1.2	unknown	unknown	Blood	2007	Asia	South Asia	Nepal	unknown	unknown	Wong et al, 2016
H07288308	ERR1017068	N/A	4.3.1	unknown	unknown	Blood	2007	Asia	South Asia	India	unknown	unknown	Wong et al, 2016
H07324312	ERR1017069	N/A	4.3.1.1	unknown	unknown	Blood	2007	Asia	South Asia	Pakistan	unknown	unknown	Wong et al, 2016
H07336300	ERR1017070	N/A	4.3.1.2	unknown	unknown	Blood	2007	Asia	South Asia	Pakistan	unknown	unknown	Wong et al, 2016
H07336301	ERR1017071	N/A	4.3.1.1	unknown	unknown	Blood	2007	Asia	South Asia	Bangladesh	unknown	unknown	Wong et al, 2016
H07364324	ERR1017072	N/A	4.3.1.1	unknown	unknown	Blood	2007	Asia	South Asia	Bangladesh	unknown	unknown	Wong et al, 2016
H07384494	ERR1017074	N/A	4.3.1.2	unknown	unknown	Blood	2007	Asia	South Asia	India	unknown	unknown	Wong et al, 2016
H07394454	ERR1017075	N/A	4.3.1	unknown	unknown	Blood	2007	Asia	South Asia	India	unknown	unknown	Wong et al, 2016
H07402281	ERR1017077	N/A	4.3.1.3	unknown	unknown	Blood	2007	Asia	South Asia	Bangladesh	unknown	unknown	Wong et al, 2016
H07404418	ERR1017078	N/A	3.1.1	unknown	unknown	Blood	2007	Africa	West Africa	Ghana	unknown	unknown	Wong et al, 2016
H08038152	ERR1017081	N/A	2.2.2	unknown	unknown	Blood	2008	Asia	South Asia	India	unknown	unknown	Wong et al, 2016
H08082282	ERR1017083	N/A	4.3.1.2	unknown	unknown	Blood	2008	Asia	South Asia	India	unknown	unknown	Wong et al, 2016
H08294356	ERR1017087	N/A	4.3.1.1	unknown	unknown	Blood	2008	Asia	South Asia	Bangladesh	unknown	unknown	Wong et al, 2016
H08302395	ERR1017088	N/A	3.2.2	unknown	unknown	Blood	2008	Asia	South Asia	Bangladesh	unknown	unknown	Wong et al, 2016
H08302396	ERR1017089	N/A	4.3.1.1	unknown	unknown	Blood	2008	Asia	South Asia	Bangladesh	unknown	unknown	Wong et al, 2016
H08328499	ERR1017090	N/A	4.3.1	unknown	unknown	Blood	2008	Asia	W Asia/S Asia	Kuwait/India	unknown	unknown	Wong et al, 2016
H08384665	ERR1017091	N/A	3.3	unknown	unknown	Blood	2008	Asia	South Asia	Pakistan	unknown	unknown	Wong et al, 2016
H08398180	ERR1017092	N/A	4.3.1	unknown	unknown	Blood	2008	Asia	W Asia/S Asia	Kuwait/India	unknown	unknown	Wong et al, 2016
H08428259	ERR1017094	N/A	4.3.1.2	unknown	unknown	Blood	2008	Asia	South Asia	India	unknown	unknown	Wong et al, 2016
H08434321	ERR1017095	N/A	3.3	unknown	unknown	Blood	2008	Asia	South Asia	India/Pakistan	unknown	unknown	Wong et al, 2016
H09044141	ERR1017096	N/A	3.1.1	unknown	unknown	Blood	2009	Africa	West Africa	Nigeria	unknown	unknown	Wong et al, 2016
H0905277	ERR1017097	N/A	4.3.1.1	unknown	unknown	Blood	2009	Asia	South Asia	Pakistan	unknown	unknown	Wong et al, 2016
H09132110	ERR1017098	N/A	3.3.1	unknown	unknown	Blood	2009	Asia	South Asia	Pakistan	unknown	unknown	Wong et al, 2016
IB5335	ERR108648	N/A	4.3.1.1.EA1	unknown	unknown	Blood	2009	Africa	East Africa	Tanzania	unknown	unknown	Wong et al, 2015
IB5336	ERR108649	N/A	2.5	unknown	unknown	Blood	2009	Africa	East Africa	Tanzania	unknown	unknown	Wong et al, 2015
IB5339	ERR108652	N/A	3.3.1	unknown	unknown	Blood	2009	Africa	East Africa	Tanzania	unknown	unknown	Wong et al, 2015
IB5340	ERR108653	N/A	3.3.1	unknown	unknown	Blood	2009	Africa	East Africa	Tanzania	unknown	unknown	Wong et al, 2015
IB5341	ERR108654	N/A	4.3.1.2	unknown	unknown	Blood	2009	Africa	East Africa	Tanzania	unknown	unknown	Wong et al, 2015
IB5342	ERR108655	N/A	3.3.1	unknown	unknown	Blood	2009	Africa	East Africa	Tanzania	unknown	unknown	Wong et al, 2015
IB5343	ERR108656	N/A	4.3.1.1.EA1	unknown	unknown	Blood	2009	Africa	East Africa	Tanzania	unknown	unknown	Wong et al, 2015
IB5345	ERR108658	N/A	4.3.1.1.EA1	unknown	unknown	Blood	2009	Africa	East Africa	Tanzania	unknown	unknown	Wong et al, 2015
IB5347	ERR108659	N/A	4.3.1.1.EA1	unknown	unknown	Blood	2009	Africa	East Africa	Tanzania	unknown	unknown	Wong et al, 2015
IB5348	ERR108660	N/A	4.3.1.1.EA1	unknown	unknown	Blood	2009	Africa	East Africa	Tanzania	unknown	unknown	Wong et al, 2015
IB5351	ERR108663	N/A	4.3.1.2	unknown	unknown	Blood	2010	Africa	East Africa	Tanzania	unknown	unknown	Wong et al, 2015
IB5352	ERR108664	N/A	4.3.1.1.EA1	unknown	unknown	Blood	2010	Africa	East Africa	Tanzania	unknown	unknown	Wong et al, 2015
IB5353	ERR108665	N/A	4.3.1.2	unknown	unknown	Blood	2010	Africa	East Africa	Tanzania	unknown	unknown	Wong et al, 2015
IB5354	ERR108666	N/A	4.3.1.2	unknown	unknown	Blood	2010	Africa	East Africa	Tanzania	unknown	unknown	Wong et al, 2015
IB5355	ERR108667	N/A	4.3.1.2	unknown	unknown	Blood	2010	Africa	East Africa	Tanzania	unknown	unknown	Wong et al, 2015
IB5356	ERR108668	N/A	4.3.1.2	unknown	unknown	Blood	2010	Africa	East Africa	Tanzania	unknown	unknown	Wong et al, 2015
IB5357	ERR108669	N/A	4.3.1.2	unknown	unknown	Blood	2010	Africa	East Africa	Tanzania	unknown	unknown	Wong et al, 2015
IB5358	ERR108670	N/A	4.3.1.2	unknown	unknown	Blood	2010	Africa	East Africa	Tanzania	unknown	unknown	Wong et al, 2015
IB5359	ERR108671	N/A	3.3.1	unknown	unknown	Blood	2010	Africa	East Africa	Tanzania	unknown	unknown	Wong et al, 2015
IB5360	ERR108672	N/A	3.3.1	unknown	unknown	Blood	2009	Africa	East Africa	Tanzania	unknown	unknown	Wong et al, 2015
IB5366	ERR108676	N/A	4.3.1.1.EA1	unknown	unknown	Blood	2009	Africa	East Africa	Tanzania	unknown	unknown	Wong et al, 2015

IB5367	ERR108677	N/A	3.3.1	unknown	unknown	Blood	2009	Africa	East Africa	Tanzania	unknown	unknown	Wong et al, 2015
IB5368	ERR108678	N/A	2.5.1	unknown	unknown	Blood	2009	Africa	East Africa	Tanzania	unknown	unknown	Wong et al, 2015
IB5369	ERR108679	N/A	4.3.1.1.EA1	unknown	unknown	Blood	2009	Africa	East Africa	Tanzania	unknown	unknown	Wong et al, 2015
IB5370	ERR108680	N/A	3.3.1	unknown	unknown	Blood	2010	Africa	East Africa	Tanzania	unknown	unknown	Wong et al, 2015
IB5371	ERR108681	N/A	3.3.1	unknown	unknown	Blood	2010	Africa	East Africa	Tanzania	unknown	unknown	Wong et al, 2015
IB5374	ERR108684	N/A	3.3.1	unknown	unknown	Blood	2010	Africa	East Africa	Tanzania	unknown	unknown	Wong et al, 2015
IB5375	ERR108685	N/A	4.3.1.2	unknown	unknown	Blood	2010	Africa	East Africa	Tanzania	unknown	unknown	Wong et al, 2015
IB5376	ERR108686	N/A	4.3.1.2	unknown	unknown	Blood	2010	Africa	East Africa	Tanzania	unknown	unknown	Wong et al, 2015
IB5378	ERR108687	N/A	4.3.1.2	unknown	unknown	Blood	2010	Africa	East Africa	Tanzania	unknown	unknown	Wong et al, 2015
IB5379	ERR108688	N/A	4.3.1.2	unknown	unknown	Blood	2010	Africa	East Africa	Tanzania	unknown	unknown	Wong et al, 2015
IB5380	ERR108689	N/A	4.3.1.2	unknown	unknown	Blood	2010	Africa	East Africa	Tanzania	unknown	unknown	Wong et al, 2015
IB5381	ERR108690	N/A	3.3.1	unknown	unknown	Blood	2010	Africa	East Africa	Tanzania	unknown	unknown	Wong et al, 2015
IB5382	ERR108691	N/A	4.3.1.2	unknown	unknown	Blood	2010	Africa	East Africa	Tanzania	unknown	unknown	Wong et al, 2015
IB5383	ERR108692	N/A	3.3.1	unknown	unknown	Blood	2009	Africa	East Africa	Tanzania	unknown	unknown	Wong et al, 2015
IB5384	ERR108693	N/A	4.3.1.1.EA1	unknown	unknown	Blood	2009	Africa	East Africa	Tanzania	unknown	unknown	Wong et al, 2015
IB5385	ERR108694	N/A	4.3.1.1.EA1	unknown	unknown	Blood	2010	Africa	East Africa	Tanzania	unknown	unknown	Wong et al, 2015
MDUST73	ERR204244	N/A	4.2.1	unknown	unknown	Unknown	2008	Australia & Oceania	Oceania	Fiji	unknown	unknown	Wong et al, 2015
MDUST74	ERR204245	N/A	4.2.1	unknown	unknown	Unknown	2008	Australia & Oceania	Oceania	Fiji	unknown	unknown	Wong et al, 2015
MDUST75	ERR204246	N/A	4.2.2	unknown	unknown	Unknown	2008	Australia & Oceania	Oceania	Fiji	unknown	unknown	Wong et al, 2015
MDUST76	ERR204247	N/A	4.2.2	unknown	unknown	Unknown	2008	Australia & Oceania	Oceania	Fiji	unknown	unknown	Wong et al, 2015
MDUST77	ERR204248	N/A	4.2.1	unknown	unknown	Unknown	2008	Australia & Oceania	Oceania	Fiji	unknown	unknown	Wong et al, 2015
MDUST78	ERR204249	N/A	4.2.1	unknown	unknown	Unknown	2008	Australia & Oceania	Oceania	Fiji	unknown	unknown	Wong et al, 2015
MDUST79	ERR204250	N/A	4.2.1	unknown	unknown	Unknown	2008	Australia & Oceania	Oceania	Fiji	unknown	unknown	Wong et al, 2015
MDUST80	ERR204251	N/A	4.2.2	unknown	unknown	Unknown	2008	Australia & Oceania	Oceania	Fiji	unknown	unknown	Wong et al, 2015
MDUST81	ERR204252	N/A	4.2.1	unknown	unknown	Unknown	2008	Australia & Oceania	Oceania	Fiji	unknown	unknown	Wong et al, 2015
MDUST82	ERR204253	N/A	4.2.1	unknown	unknown	Unknown	2008	Australia & Oceania	Oceania	Fiji	unknown	unknown	Wong et al, 2015
MDUST83	ERR204254	N/A	4.2.2	unknown	unknown	Unknown	2008	Australia & Oceania	Oceania	Fiji	unknown	unknown	Wong et al, 2015
MDUST84	ERR204255	N/A	4.2.1	unknown	unknown	Unknown	2008	Australia & Oceania	Oceania	Fiji	unknown	unknown	Wong et al, 2015
MDUST85	ERR204256	N/A	4.2.1	unknown	unknown	Unknown	2008	Australia & Oceania	Oceania	Fiji	unknown	unknown	Wong et al, 2015
MDUST86	ERR204257	N/A	4.2.1	unknown	unknown	Unknown	2008	Australia & Oceania	Oceania	Fiji	unknown	unknown	Wong et al, 2015
MDUST87	ERR204258	N/A	4.2.1	unknown	unknown	Unknown	2008	Australia & Oceania	Oceania	Fiji	unknown	unknown	Wong et al, 2015
MDUST88	ERR204259	N/A	4.2.1	unknown	unknown	Unknown	2008	Australia & Oceania	Oceania	Fiji	unknown	unknown	Wong et al, 2015
MDUST89	ERR204260	N/A	4.2.2	unknown	unknown	Unknown	2008	Australia & Oceania	Oceania	Fiji	unknown	unknown	Wong et al, 2015
MDUST90	ERR204261	N/A	4.2.2	unknown	unknown	Unknown	2008	Australia & Oceania	Oceania	Fiji	unknown	unknown	Wong et al, 2015

MDUST91	ERR204262	N/A	4.2.2	unknown	unknown	Unknown	2008	Australia & Oceania	Oceania	Fiji	unknown	unknown	Wong et al, 2015
MDUST92	ERR204263	N/A	4.2.2	unknown	unknown	Unknown	2008	Australia & Oceania	Oceania	Fiji	unknown	unknown	Wong et al, 2015
MDUST93	ERR204264	N/A	4.2.2	unknown	unknown	Unknown	2008	Australia & Oceania	Oceania	Fiji	unknown	unknown	Wong et al, 2015
MDUST94	ERR204265	N/A	4.2.2	unknown	unknown	Unknown	2008	Australia & Oceania	Oceania	Fiji	unknown	unknown	Wong et al, 2015
MDUST95	ERR204266	N/A	4.2.2	unknown	unknown	Unknown	2008	Australia & Oceania	Oceania	Fiji	unknown	unknown	Wong et al, 2015
MDUST96	ERR204267	N/A	4.2.2	unknown	unknown	Unknown	2008	Australia & Oceania	Oceania	Fiji	unknown	unknown	Wong et al, 2015
MDUST98	ERR204269	N/A	4.2.2	unknown	unknown	Unknown	2008	Australia & Oceania	Oceania	Fiji	unknown	unknown	Wong et al, 2015
MDUST99	ERR204270	N/A	4.2.1	unknown	unknown	Unknown	2008	Australia & Oceania	Oceania	Fiji	unknown	unknown	Wong et al, 2015
MDUST100	ERR204271	N/A	4.2.1	unknown	unknown	Unknown	2008	Australia & Oceania	Oceania	Fiji	unknown	unknown	Wong et al, 2015
MDUST101	ERR204272	N/A	4.2.2	unknown	unknown	Unknown	2008	Australia & Oceania	Oceania	Fiji	unknown	unknown	Wong et al, 2015
MDUST102	ERR204273	N/A	4.2.2	unknown	unknown	Unknown	2008	Australia & Oceania	Oceania	Fiji	unknown	unknown	Wong et al, 2015
MDUST103	ERR204274	N/A	4.2.2	unknown	unknown	Unknown	2008	Australia & Oceania	Oceania	Fiji	unknown	unknown	Wong et al, 2015
MDUST104	ERR204275	N/A	4.2.2	unknown	unknown	Unknown	2008	Australia & Oceania	Oceania	Fiji	unknown	unknown	Wong et al, 2015
MDUST105	ERR204276	N/A	4.2.2	unknown	unknown	Unknown	2008	Australia & Oceania	Oceania	Fiji	unknown	unknown	Wong et al, 2015
Ke378	ERR212564	N/A	4.3.1.1.EA1	unknown	unknown	Blood	2008	Africa	East Africa	Kenya	unknown	unknown	Wong et al, 2015
Ke451	ERR212636	N/A	4.3.1.1.EA1	unknown	unknown	Blood	2008	Africa	East Africa	Kenya	unknown	unknown	Wong et al, 2015
Ke452	ERR212637	N/A	4.3.1.2.EA2	unknown	unknown	Blood	2008	Africa	East Africa	Kenya	unknown	unknown	Wong et al, 2015
Ke453	ERR212638	N/A	4.3.1.1.EA1	unknown	unknown	Blood	2008	Africa	East Africa	Kenya	unknown	unknown	Wong et al, 2015
Ke454	ERR212639	N/A	4.3.1.1.EA1	unknown	unknown	Blood	2008	Africa	East Africa	Kenya	unknown	unknown	Wong et al, 2015
Ke455	ERR212640	N/A	4.3.1.1.EA1	unknown	unknown	Blood	2008	Africa	East Africa	Kenya	unknown	unknown	Wong et al, 2015
Ke458	ERR212643	N/A	4.3.1.1.EA1	unknown	unknown	Blood	2008	Africa	East Africa	Kenya	unknown	unknown	Wong et al, 2015
Ke461	ERR212646	N/A	4.3.1.1.EA1	unknown	unknown	Blood	2008	Africa	East Africa	Kenya	unknown	unknown	Wong et al, 2015
Ke462	ERR212647	N/A	2.5	unknown	unknown	Blood	2008	Africa	East Africa	Kenya	unknown	unknown	Wong et al, 2015
Ke463	ERR212648	N/A	4.3.1.1.EA1	unknown	unknown	Blood	2008	Africa	East Africa	Kenya	unknown	unknown	Wong et al, 2015
Ke466	ERR212651	N/A	4.3.1.1.EA1	unknown	unknown	Blood	2008	Africa	East Africa	Kenya	unknown	unknown	Wong et al, 2015
Ke467	ERR212652	N/A	4.3.1.1.EA1	unknown	unknown	Blood	2008	Africa	East Africa	Kenya	unknown	unknown	Wong et al, 2015
Ke468	ERR212653	N/A	4.3.1.1.EA1	unknown	unknown	Blood	2008	Africa	East Africa	Kenya	unknown	unknown	Wong et al, 2015
Ke469	ERR212654	N/A	4.3.1.1.EA1	unknown	unknown	Blood	2008	Africa	East Africa	Kenya	unknown	unknown	Wong et al, 2015
Ke470	ERR212655	N/A	4.3.1.1.EA1	unknown	unknown	Blood	2008	Africa	East Africa	Kenya	unknown	unknown	Wong et al, 2015
Ke472	ERR212657	N/A	4.3.1.1.EA1	unknown	unknown	Blood	2008	Africa	East Africa	Kenya	unknown	unknown	Wong et al, 2015
Ke474	ERR212659	N/A	4.3.1.1.EA1	unknown	unknown	Blood	2008	Africa	East Africa	Kenya	unknown	unknown	Wong et al, 2015
Ke475	ERR212660	N/A	4.3.1.1.EA1	unknown	unknown	Blood	2008	Africa	East Africa	Kenya	unknown	unknown	Wong et al, 2015
Ke476	ERR212661	N/A	4.3.1.1.EA1	unknown	unknown	Blood	2008	Africa	East Africa	Kenya	unknown	unknown	Wong et al, 2015
Ke477	ERR212662	N/A	4.3.1.1.EA1	unknown	unknown	Blood	2008	Africa	East Africa	Kenya	unknown	unknown	Wong et al, 2015
Ke479	ERR212664	N/A	4.3.1.1.EA1	unknown	unknown	Blood	2008	Africa	East Africa	Kenya	unknown	unknown	Wong et al, 2015
Ke480	ERR212665	N/A	4.3.1.1.EA1	unknown	unknown	Blood	2008	Africa	East Africa	Kenya	unknown	unknown	Wong et al, 2015

Ke481	ERR212666	N/A	4.3.1.1.EA1	unknown	unknown	Blood	2008	Africa	East Africa	Kenya	unknown	unknown	Wong et al, 2015
Ke482	ERR212667	N/A	4.3.1.1.EA1	unknown	unknown	Blood	2008	Africa	East Africa	Kenya	unknown	unknown	Wong et al, 2015
Ke492	ERR212677	N/A	4.3.1.2.EA2	unknown	unknown	Blood	2001	Africa	East Africa	Kenya	unknown	unknown	Wong et al, 2015
MDUST1	ERR213219	N/A	4.2.2	unknown	unknown	Unknown	2011	Australia & Oceania	Oceania	Fiji	unknown	unknown	Wong et al, 2015
MDUST2	ERR213220	N/A	4.2.2	unknown	unknown	Unknown	2011	Australia & Oceania	Oceania	Fiji	unknown	unknown	Wong et al, 2015
MDUST3	ERR213221	N/A	4.2.2	unknown	unknown	Unknown	2011	Australia & Oceania	Oceania	Fiji	unknown	unknown	Wong et al, 2015
MDUST4	ERR213222	N/A	4.2.2	unknown	unknown	Unknown	2011	Australia & Oceania	Oceania	Fiji	unknown	unknown	Wong et al, 2015
MDUST5	ERR213223	N/A	4.2.2	unknown	unknown	Unknown	2011	Australia & Oceania	Oceania	Fiji	unknown	unknown	Wong et al, 2015
MDUST6	ERR213224	N/A	4.2.2	unknown	unknown	Unknown	2011	Australia & Oceania	Oceania	Fiji	unknown	unknown	Wong et al, 2015
MDUST7	ERR213225	N/A	4.2.2	unknown	unknown	Unknown	2011	Australia & Oceania	Oceania	Fiji	unknown	unknown	Wong et al, 2015
MDUST8	ERR213226	N/A	4.2.2	unknown	unknown	Unknown	2011	Australia & Oceania	Oceania	Fiji	unknown	unknown	Wong et al, 2015
MDUST9	ERR213227	N/A	4.2.2	unknown	unknown	Unknown	2011	Australia & Oceania	Oceania	Fiji	unknown	unknown	Wong et al, 2015
MDUST10	ERR213228	N/A	4.2.2	unknown	unknown	Unknown	2011	Australia & Oceania	Oceania	Fiji	unknown	unknown	Wong et al, 2015
MDUST11	ERR213229	N/A	4.2.2	unknown	unknown	Unknown	2011	Australia & Oceania	Oceania	Fiji	unknown	unknown	Wong et al, 2015
MDUST12	ERR213230	N/A	4.2.2	unknown	unknown	Unknown	2011	Australia & Oceania	Oceania	Fiji	unknown	unknown	Wong et al, 2015
MDUST13	ERR213231	N/A	4.2.2	unknown	unknown	Unknown	2011	Australia & Oceania	Oceania	Fiji	unknown	unknown	Wong et al, 2015
MDUST14	ERR213232	N/A	4.2.2	unknown	unknown	Unknown	2011	Australia & Oceania	Oceania	Fiji	unknown	unknown	Wong et al, 2015
MDUST15	ERR213233	N/A	4.2.1	unknown	unknown	Unknown	2011	Australia & Oceania	Oceania	Fiji	unknown	unknown	Wong et al, 2015
MDUST16	ERR213234	N/A	4.2.2	unknown	unknown	Unknown	2011	Australia & Oceania	Oceania	Fiji	unknown	unknown	Wong et al, 2015
MDUST17	ERR213235	N/A	4.2.2	unknown	unknown	Unknown	2011	Australia & Oceania	Oceania	Fiji	unknown	unknown	Wong et al, 2015
MDUST18	ERR213236	N/A	4.2.2	unknown	unknown	Unknown	2011	Australia & Oceania	Oceania	Fiji	unknown	unknown	Wong et al, 2015
MDUST19	ERR213237	N/A	4.2.2	unknown	unknown	Unknown	2011	Australia & Oceania	Oceania	Fiji	unknown	unknown	Wong et al, 2015
MDUST20	ERR213238	N/A	4.2.2	unknown	unknown	Unknown	2011	Australia & Oceania	Oceania	Fiji	unknown	unknown	Wong et al, 2015
MDUST21	ERR213239	N/A	4.2.2	unknown	unknown	Unknown	2011	Australia & Oceania	Oceania	Fiji	unknown	unknown	Wong et al, 2015
MDUST22	ERR213240	N/A	4.2.2	unknown	unknown	Unknown	2011	Australia & Oceania	Oceania	Fiji	unknown	unknown	Wong et al, 2015
MDUST23	ERR213241	N/A	4.2.2	unknown	unknown	Unknown	2011	Australia & Oceania	Oceania	Fiji	unknown	unknown	Wong et al, 2015
MDUST24	ERR213242	N/A	4.2.2	unknown	unknown	Unknown	2011	Australia & Oceania	Oceania	Fiji	unknown	unknown	Wong et al, 2015
MDUST25	ERR213243	N/A	4.2.2	unknown	unknown	Unknown	2011	Australia & Oceania	Oceania	Fiji	unknown	unknown	Wong et al, 2015
MDUST26	ERR213244	N/A	4.2.2	unknown	unknown	Unknown	2011	Australia & Oceania	Oceania	Fiji	unknown	unknown	Wong et al, 2015

MDUST27	ERR213245	N/A	4.2.2	unknown	unknown	Unknown	2011	Australia & Oceania	Oceania	Fiji	unknown	unknown	Wong et al, 2015
MDUST28	ERR213246	N/A	4.2.2	unknown	unknown	Unknown	2011	Australia & Oceania	Oceania	Fiji	unknown	unknown	Wong et al, 2015
MDUST29	ERR213247	N/A	4.2.2	unknown	unknown	Unknown	2011	Australia & Oceania	Oceania	Fiji	unknown	unknown	Wong et al, 2015
MDUST30	ERR213248	N/A	4.2.2	unknown	unknown	Stool	2009	Australia & Oceania	Oceania	Fiji	unknown	unknown	Wong et al, 2015
MDUST31	ERR213249	N/A	4.2.1	unknown	unknown	Blood	2000	Australia & Oceania	Oceania	Fiji	unknown	unknown	Wong et al, 2015
MDUST32	ERR213250	N/A	4.2.1	unknown	unknown	Blood	2000	Australia & Oceania	Oceania	Fiji	unknown	unknown	Wong et al, 2015
MDUST33	ERR213251	N/A	4.2.1	unknown	unknown	Stool	1997	Australia & Oceania	Oceania	Fiji	unknown	unknown	Wong et al, 2015
MDUST34	ERR213252	N/A	4.2.1	unknown	unknown	Pus	1997	Australia & Oceania	Oceania	Fiji	unknown	unknown	Wong et al, 2015
MDUST35	ERR213253	N/A	4.2	unknown	unknown	Blood	1996	Australia & Oceania	Oceania	Fiji	unknown	unknown	Wong et al, 2015
MDUST36	ERR213254	N/A	4.2.1	unknown	unknown	Stool	1996	Australia & Oceania	Oceania	Fiji	unknown	unknown	Wong et al, 2015
MDUST37	ERR213255	N/A	4.2.1	unknown	unknown	Stool	1996	Australia & Oceania	Oceania	Fiji	unknown	unknown	Wong et al, 2015
MDUST38	ERR213256	N/A	4.2.1	unknown	unknown	Stool	1996	Australia & Oceania	Oceania	Fiji	unknown	unknown	Wong et al, 2015
MDUST39	ERR213257	N/A	4.2.1	unknown	unknown	Stool	1996	Australia & Oceania	Oceania	Fiji	unknown	unknown	Wong et al, 2015
MDUST40	ERR213258	N/A	4.2.1	unknown	unknown	Stool	1996	Australia & Oceania	Oceania	Fiji	unknown	unknown	Wong et al, 2015
MDUST41	ERR213259	N/A	4.2.1	unknown	unknown	Blood	1996	Australia & Oceania	Oceania	Fiji	unknown	unknown	Wong et al, 2015
MDUST42	ERR213260	N/A	4.2.3	unknown	unknown	Blood	1996	Australia & Oceania	Oceania	Fiji	unknown	unknown	Wong et al, 2015
MDUST43	ERR213261	N/A	4.2.3	unknown	unknown	Blood	1996	Australia & Oceania	Oceania	Fiji	unknown	unknown	Wong et al, 2015
MDUST44	ERR213262	N/A	3.5	unknown	unknown	Blood	1996	Australia & Oceania	Oceania	Fiji	unknown	unknown	Wong et al, 2015
MDUST45	ERR213263	N/A	0.1	unknown	unknown	Blood	1996	Australia & Oceania	Oceania	Fiji	unknown	unknown	Wong et al, 2015
MDUST46	ERR213264	N/A	2	unknown	unknown	Blood	1996	Australia & Oceania	Oceania	Fiji	unknown	unknown	Wong et al, 2015
MDUST47	ERR213265	N/A	4.2.3	unknown	unknown	Blood	1996	Australia & Oceania	Oceania	Fiji	unknown	unknown	Wong et al, 2015
MDUST48	ERR213266	N/A	2.3.5	unknown	unknown	Stool	1996	Australia & Oceania	Oceania	Fiji	unknown	unknown	Wong et al, 2015
MDUST49	ERR213267	N/A	4.2	unknown	unknown	Stool	1996	Australia & Oceania	Oceania	Fiji	unknown	unknown	Wong et al, 2015
MDUST50	ERR213268	N/A	4.2	unknown	unknown	Stool	1996	Australia & Oceania	Oceania	Fiji	unknown	unknown	Wong et al, 2015
MDUST51	ERR213269	N/A	4.2.3	unknown	unknown	Stool	1995	Australia & Oceania	Oceania	Fiji	unknown	unknown	Wong et al, 2015
MDUST52	ERR213270	N/A	4.2	unknown	unknown	Stool	1995	Australia & Oceania	Oceania	Fiji	unknown	unknown	Wong et al, 2015
MDUST53	ERR213271	N/A	4.2.3	unknown	unknown	Stool	1995	Australia & Oceania	Oceania	Fiji	unknown	unknown	Wong et al, 2015
MDUST54	ERR213272	N/A	4.2.3	unknown	unknown	Stool	1995	Australia & Oceania	Oceania	Fiji	unknown	unknown	Wong et al, 2015

MDUST55	ERR213273	N/A	4.3.1.1	unknown	unknown	Blood	1995	Australia & Oceania	Oceania	Fiji	unknown	unknown	Wong et al, 2015
MDUST56	ERR213274	N/A	2.3.5	unknown	unknown	Stool	1994	Australia & Oceania	Oceania	Fiji	unknown	unknown	Wong et al, 2015
MDUST57	ERR213275	N/A	2.3.5	unknown	unknown	Blood	1994	Australia & Oceania	Oceania	Fiji	unknown	unknown	Wong et al, 2015
MDUST58	ERR213276	N/A	4.2.3	unknown	unknown	Stool	1994	Australia & Oceania	Oceania	Fiji	unknown	unknown	Wong et al, 2015
MDUST59	ERR213277	N/A	4.2.3	unknown	unknown	Stool	1994	Australia & Oceania	Oceania	Fiji	unknown	unknown	Wong et al, 2015
MDUST60	ERR213278	N/A	4.2.3	unknown	unknown	Stool	1994	Australia & Oceania	Oceania	Fiji	unknown	unknown	Wong et al, 2015
MDUST61	ERR213279	N/A	4.2.3	unknown	unknown	Stool	1994	Australia & Oceania	Oceania	Fiji	unknown	unknown	Wong et al, 2015
MDUST62	ERR213280	N/A	4.2.1	unknown	unknown	Blood	1994	Australia & Oceania	Oceania	Fiji	unknown	unknown	Wong et al, 2015
MDUST63	ERR213281	N/A	2.3.5	unknown	unknown	Stool	1994	Australia & Oceania	Oceania	Fiji	unknown	unknown	Wong et al, 2015
MDUST64	ERR213282	N/A	2.3.5	unknown	unknown	Stool & Blood	1994	Australia & Oceania	Oceania	Fiji	unknown	unknown	Wong et al, 2015
MDUST65	ERR213283	N/A	2.3.5	unknown	unknown	Stool	1994	Australia & Oceania	Oceania	Fiji	unknown	unknown	Wong et al, 2015
MDUST66	ERR213284	N/A	2.3.5	unknown	unknown	Stool	1994	Australia & Oceania	Oceania	Fiji	unknown	unknown	Wong et al, 2015
MDUST67	ERR213285	N/A	4.3.1.1	unknown	unknown	Stool	1994	Australia & Oceania	Oceania	Fiji	unknown	unknown	Wong et al, 2015
MDUST68	ERR213286	N/A	2.3.5	unknown	unknown	Stool	1994	Australia & Oceania	Oceania	Fiji	unknown	unknown	Wong et al, 2015
MDUST69	ERR213287	N/A	4.2.3	unknown	unknown	Stool	1996	Australia & Oceania	Oceania	Fiji	unknown	unknown	Wong et al, 2015
MDUST70	ERR213288	N/A	4.2.2	unknown	unknown	Unknown	2008	Australia & Oceania	Oceania	Fiji	unknown	unknown	Wong et al, 2015
MDUST71	ERR213289	N/A	4.2.2	unknown	unknown	Unknown	2008	Australia & Oceania	Oceania	Fiji	unknown	unknown	Wong et al, 2015
MDUST72	ERR213290	N/A	4.2.2	unknown	unknown	Unknown	2008	Australia & Oceania	Oceania	Fiji	unknown	unknown	Wong et al, 2015
Ke062	ERR217406	N/A	4.3.1.1.EA1	unknown	unknown	Blood	2004	Africa	East Africa	Kenya	unknown	unknown	Wong et al, 2015
1010996	ERR279098	N/A	2.2	unknown	unknown	Blood	2011	Africa	Southern Africa	Malawi	unknown	unknown	Wong et al, 2015
1014063	ERR279099	N/A	4.3.1.1.EA1	unknown	unknown	Blood	2011	Africa	Southern Africa	Malawi	unknown	unknown	Wong et al, 2015
1019695	ERR279100	N/A	4.3.1.1.EA1	unknown	unknown	Blood	2011	Africa	Southern Africa	Malawi	unknown	unknown	Wong et al, 2015
1019515	ERR279101	N/A	4.3.1.1.EA1	unknown	unknown	Blood	2011	Africa	Southern Africa	Malawi	unknown	unknown	Wong et al, 2015
1021678	ERR279102	N/A	4.3.1.1.EA1	unknown	unknown	Blood	2011	Africa	Southern Africa	Malawi	unknown	unknown	Wong et al, 2015
1025912	ERR279103	N/A	4.3.1.1.EA1	unknown	unknown	Blood	2011	Africa	Southern Africa	Malawi	unknown	unknown	Wong et al, 2015
1030208	ERR279105	N/A	4.3.1.1.EA1	unknown	unknown	Blood	2012	Africa	Southern Africa	Malawi	unknown	unknown	Wong et al, 2015
1008634	ERR279106	N/A	4.3.1.1.EA1	unknown	unknown	Blood	2011	Africa	Southern Africa	Malawi	unknown	unknown	Wong et al, 2015
1013486	ERR279107	N/A	4.1.1	unknown	unknown	Blood	2011	Africa	Southern Africa	Malawi	unknown	unknown	Wong et al, 2015
1021106	ERR279109	N/A	4.3.1.1.EA1	unknown	unknown	Blood	2011	Africa	Southern Africa	Malawi	unknown	unknown	Wong et al, 2015
1021701	ERR279110	N/A	2.4.1	unknown	unknown	Blood	2011	Africa	Southern Africa	Malawi	unknown	unknown	Wong et al, 2015
1025691	ERR279111	N/A	2.4.1	unknown	unknown	Blood	2011	Africa	Southern Africa	Malawi	unknown	unknown	Wong et al, 2015
1031809	ERR279113	N/A	4.3.1.1.EA1	unknown	unknown	Blood	2012	Africa	Southern Africa	Malawi	unknown	unknown	Wong et al, 2015
1007914	ERR279114	N/A	4.3.1.1.EA1	unknown	unknown	Blood	2011	Africa	Southern Africa	Malawi	unknown	unknown	Wong et al, 2015
1013305	ERR279115	N/A	4.3.1.1	unknown	unknown	Blood	2011	Africa	Southern Africa	Malawi	unknown	unknown	Wong et al, 2015

1016889	ERR279116	N/A	4.3.1.1.EA1	unknown	unknown	Blood	2011	Africa	Southern Africa	Malawi	unknown	unknown	Wong et al, 2015
1018216	ERR279117	N/A	4.1.1	unknown	unknown	Blood	2011	Africa	Southern Africa	Malawi	unknown	unknown	Wong et al, 2015
1023407	ERR279118	N/A	4.3.1.1.EA1	unknown	unknown	Blood	2011	Africa	Southern Africa	Malawi	unknown	unknown	Wong et al, 2015
1031785	ERR279121	N/A	4.3.1.1.EA1	unknown	unknown	Blood	2012	Africa	Southern Africa	Malawi	unknown	unknown	Wong et al, 2015
1007917	ERR279122	N/A	4.3.1.1.EA1	unknown	unknown	Blood	2011	Africa	Southern Africa	Malawi	unknown	unknown	Wong et al, 2015
1012911	ERR279123	N/A	4.3.1.1.EA1	unknown	unknown	Blood	2011	Africa	Southern Africa	Malawi	unknown	unknown	Wong et al, 2015
1016653	ERR279124	N/A	4.3.1.1.EA1	unknown	unknown	Blood	2011	Africa	Southern Africa	Malawi	unknown	unknown	Wong et al, 2015
1020791	ERR279125	N/A	4.3.1.1.EA1	unknown	unknown	Blood	2011	Africa	Southern Africa	Malawi	unknown	unknown	Wong et al, 2015
1022981	ERR279126	N/A	4.1.1	unknown	unknown	Blood	2011	Africa	Southern Africa	Malawi	unknown	unknown	Wong et al, 2015
1022553	ERR279128	N/A	4.3.1.1.EA1	unknown	unknown	Blood	2011	Africa	Southern Africa	Malawi	unknown	unknown	Wong et al, 2015
1030630	ERR279129	N/A	4.3.1.1.EA1	unknown	unknown	Blood	2012	Africa	Southern Africa	Malawi	unknown	unknown	Wong et al, 2015
1014451	ERR279130	N/A	4.3.1.1.EA1	unknown	unknown	Blood	2011	Africa	Southern Africa	Malawi	unknown	unknown	Wong et al, 2015
1018211	ERR279131	N/A	4.3.1.1.EA1	unknown	unknown	Blood	2011	Africa	Southern Africa	Malawi	unknown	unknown	Wong et al, 2015
1016605	ERR279132	N/A	4.3.1.1.EA1	unknown	unknown	Blood	2011	Africa	Southern Africa	Malawi	unknown	unknown	Wong et al, 2015
1021854	ERR279133	N/A	4.3.1.1.EA1	unknown	unknown	Blood	2011	Africa	Southern Africa	Malawi	unknown	unknown	Wong et al, 2015
1022075	ERR279134	N/A	4.1.1	unknown	unknown	Blood	2011	Africa	Southern Africa	Malawi	unknown	unknown	Wong et al, 2015
1032927	ERR279136	N/A	4.3.1.1.EA1	unknown	unknown	Blood	2012	Africa	Southern Africa	Malawi	unknown	unknown	Wong et al, 2015
1014383	ERR279138	N/A	4.3.1.1.EA1	unknown	unknown	Blood	2011	Africa	Southern Africa	Malawi	unknown	unknown	Wong et al, 2015
1017142	ERR279139	N/A	4.1.1	unknown	unknown	Blood	2011	Africa	Southern Africa	Malawi	unknown	unknown	Wong et al, 2015
1020184	ERR279140	N/A	3.3.1	unknown	unknown	Blood	2011	Africa	Southern Africa	Malawi	unknown	unknown	Wong et al, 2015
1023267	ERR279141	N/A	2.4.1	unknown	unknown	Blood	2011	Africa	Southern Africa	Malawi	unknown	unknown	Wong et al, 2015
1021624	ERR279142	N/A	4.3.1.1.EA1	unknown	unknown	Blood	2011	Africa	Southern Africa	Malawi	unknown	unknown	Wong et al, 2015
1024341	ERR279143	N/A	4.3.1.1	unknown	unknown	Blood	2011	Africa	Southern Africa	Malawi	unknown	unknown	Wong et al, 2015
1032463	ERR279144	N/A	4.3.1.1.EA1	unknown	unknown	Blood	2012	Africa	Southern Africa	Malawi	unknown	unknown	Wong et al, 2015
A53009	ERR279145	N/A	2.4.1	unknown	unknown	Blood	2008	Africa	Southern Africa	Malawi	unknown	unknown	Wong et al, 2015
1013803	ERR279146	N/A	4.3.1.1	unknown	unknown	Blood	2011	Africa	Southern Africa	Malawi	unknown	unknown	Wong et al, 2015
1017137	ERR279147	N/A	4.3.1.1.EA1	unknown	unknown	Blood	2011	Africa	Southern Africa	Malawi	unknown	unknown	Wong et al, 2015
1019839	ERR279148	N/A	4.3.1.1.EA1	unknown	unknown	Blood	2011	Africa	Southern Africa	Malawi	unknown	unknown	Wong et al, 2015
1022401	ERR279149	N/A	4.3.1.1.EA1	unknown	unknown	Blood	2011	Africa	Southern Africa	Malawi	unknown	unknown	Wong et al, 2015
1020976	ERR279150	N/A	4.3.1.1.EA1	unknown	unknown	Blood	2011	Africa	Southern Africa	Malawi	unknown	unknown	Wong et al, 2015
1023999	ERR279151	N/A	4.3.1.1.EA1	unknown	unknown	Blood	2011	Africa	Southern Africa	Malawi	unknown	unknown	Wong et al, 2015
1032281	ERR279152	N/A	4.3.1.1.EA1	unknown	unknown	Blood	2012	Africa	Southern Africa	Malawi	unknown	unknown	Wong et al, 2015
A53789	ERR279153	N/A	4.3.1	unknown	unknown	Blood	2009	Africa	Southern Africa	Malawi	unknown	unknown	Wong et al, 2015
1012752	ERR279154	N/A	2.4.1	unknown	unknown	Blood	2011	Africa	Southern Africa	Malawi	unknown	unknown	Wong et al, 2015
1020366	ERR279155	N/A	4.3.1.1.EA1	unknown	unknown	Blood	2011	Africa	Southern Africa	Malawi	unknown	unknown	Wong et al, 2015
1019833	ERR279156	N/A	4.3.1.1.EA1	unknown	unknown	Blood	2011	Africa	Southern Africa	Malawi	unknown	unknown	Wong et al, 2015
1029975	ERR279158	N/A	4.3.1.1.EA1	unknown	unknown	Blood	2012	Africa	Southern Africa	Malawi	unknown	unknown	Wong et al, 2015
1028143	ERR279159	N/A	4.3.1.1.EA1	unknown	unknown	Blood	2012	Africa	Southern Africa	Malawi	unknown	unknown	Wong et al, 2015
1032249	ERR279160	N/A	4.3.1.1.EA1	unknown	unknown	Blood	2012	Africa	Southern Africa	Malawi	unknown	unknown	Wong et al, 2015
D50739	ERR279161	N/A	2.4.1	unknown	unknown	Blood	2009	Africa	Southern Africa	Malawi	unknown	unknown	Wong et al, 2015
1012727	ERR279162	N/A	4.3.1.1.EA1	unknown	unknown	Blood	2011	Africa	Southern Africa	Malawi	unknown	unknown	Wong et al, 2015
1020059	ERR279163	N/A	4.3.1.1.EA1	unknown	unknown	Blood	2011	Africa	Southern Africa	Malawi	unknown	unknown	Wong et al, 2015
1019806	ERR279164	N/A	4.3.1.1.EA1	unknown	unknown	Blood	2011	Africa	Southern Africa	Malawi	unknown	unknown	Wong et al, 2015
1018617	ERR279165	N/A	4.3.1.1.EA1	unknown	unknown	Blood	2011	Africa	Southern Africa	Malawi	unknown	unknown	Wong et al, 2015
1029451	ERR279166	N/A	4.3.1.1.EA1	unknown	unknown	Blood	2012	Africa	Southern Africa	Malawi	unknown	unknown	Wong et al, 2015

1032168	ERR279168	N/A	4.3.1.1.EA1	unknown	unknown	Blood	2012	Africa	Southern Africa	Malawi	unknown	unknown	Wong et al, 2015
A55865	ERR279169	N/A	2.2	unknown	unknown	Blood	2009	Africa	Southern Africa	Malawi	unknown	unknown	Wong et al, 2015
1014152	ERR279170	N/A	4.3.1.1.EA1	unknown	unknown	Blood	2011	Africa	Southern Africa	Malawi	unknown	unknown	Wong et al, 2015
1021788	ERR279173	N/A	4.3.1.1.EA1	unknown	unknown	Blood	2011	Africa	Southern Africa	Malawi	unknown	unknown	Wong et al, 2015
1028963	ERR279174	N/A	4.3.1.1.EA1	unknown	unknown	Blood	2012	Africa	Southern Africa	Malawi	unknown	unknown	Wong et al, 2015
1026460	ERR279175	N/A	4.3.1.1.EA1	unknown	unknown	Blood	2012	Africa	Southern Africa	Malawi	unknown	unknown	Wong et al, 2015
1031581	ERR279176	N/A	4.3.1.1.EA1	unknown	unknown	Blood	2012	Africa	Southern Africa	Malawi	unknown	unknown	Wong et al, 2015
A32420	ERR279177	N/A	2.2	unknown	unknown	Blood	2004	Africa	Southern Africa	Malawi	unknown	unknown	Wong et al, 2015
A33112	ERR279178	N/A	2	unknown	unknown	Blood	2005	Africa	Southern Africa	Malawi	unknown	unknown	Wong et al, 2015
A40201	ERR279182	N/A	2.4.1	unknown	unknown	Blood	2006	Africa	Southern Africa	Malawi	unknown	unknown	Wong et al, 2015
D28995	ERR279187	N/A	2.4.1	unknown	unknown	Blood	2005	Africa	Southern Africa	Malawi	unknown	unknown	Wong et al, 2015
A39483	ERR279189	N/A	4.1.1	unknown	unknown	Blood	2006	Africa	Southern Africa	Malawi	unknown	unknown	Wong et al, 2015
A40566	ERR279190	N/A	2.4.1	unknown	unknown	Blood	2006	Africa	Southern Africa	Malawi	unknown	unknown	Wong et al, 2015
D41342	ERR279191	N/A	4.1.1	unknown	unknown	Blood	2007	Africa	Southern Africa	Malawi	unknown	unknown	Wong et al, 2015
D7558	ERR279344	N/A	4.3.1	unknown	unknown	Not provided	2006	Asia	South Asia	India	unknown	unknown	Wong et al, 2015
D7649	ERR279345	N/A	4.3.1	unknown	unknown	Not provided	2007	Asia	South Asia	India	unknown	unknown	Wong et al, 2015
C3551	ERR279346	N/A	4.3.1.2	unknown	unknown	Not provided	2005	Asia	South Asia	India	unknown	unknown	Wong et al, 2015
C3891	ERR279347	N/A	4.3.1.2	unknown	unknown	Not provided	2005	Asia	South Asia	India	unknown	unknown	Wong et al, 2015
C3495	ERR279348	N/A	4.3.1.1	unknown	unknown	Not provided	2005	Asia	South Asia	India	unknown	unknown	Wong et al, 2015
C3634	ERR279349	N/A	4.3.1.1	unknown	unknown	Not provided	2005	Asia	South Asia	India	unknown	unknown	Wong et al, 2015
E2889	ERR279350	N/A	4.3.1.2	unknown	unknown	Not provided	2006	Asia	South Asia	India	unknown	unknown	Wong et al, 2015
E2990	ERR279351	N/A	4.3.1.2	unknown	unknown	Not provided	2006	Asia	South Asia	India	unknown	unknown	Wong et al, 2015
E1240	ERR279352	N/A	2.2.4	unknown	unknown	Not provided	2004	Asia	South Asia	India	unknown	unknown	Wong et al, 2015
E1303	ERR279353	N/A	2.2.4	unknown	unknown	Not provided	2004	Asia	South Asia	India	unknown	unknown	Wong et al, 2015
Quailes	ERR294858	N/A	3.1	unknown	unknown	Gallbladder fluid	1958	North America	North America	United States of America	unknown	unknown	Wong et al, 2015
2008-001909	ERR319402	N/A	4.3.1.1	unknown	unknown	Blood	2008	Asia	Southeast Asia	Cambodia	unknown	unknown	Wong et al, 2015
2008-002399	ERR319403	N/A	4.3.1.1	unknown	unknown	Blood	2008	Asia	Southeast Asia	Cambodia	unknown	unknown	Wong et al, 2015
2008-003956	ERR319404	N/A	4.3.1.1	unknown	unknown	Blood	2008	Asia	Southeast Asia	Cambodia	unknown	unknown	Wong et al, 2015
2008-004254	ERR319405	N/A	4.3.1.1	unknown	unknown	Blood	2008	Asia	Southeast Asia	Cambodia	unknown	unknown	Wong et al, 2015
2002-216507	ERR319406	N/A	4.3.1.1	unknown	unknown	Blood	2008	Asia	Southeast Asia	Cambodia	unknown	unknown	Wong et al, 2015
2003-016955	ERR319408	N/A	4.3.1.1	unknown	unknown	Blood	2008	Asia	Southeast Asia	Cambodia	unknown	unknown	Wong et al, 2015
2008-006312	ERR319409	N/A	4.3.1.1	unknown	unknown	Blood	2008	Asia	Southeast Asia	Cambodia	unknown	unknown	Wong et al, 2015
2005-008425	ERR319410	N/A	4.3.1.1	unknown	unknown	Blood	2008	Asia	Southeast Asia	Cambodia	unknown	unknown	Wong et al, 2015
2008-006300	ERR319411	N/A	4.3.1.1	unknown	unknown	Blood	2008	Asia	Southeast Asia	Cambodia	unknown	unknown	Wong et al, 2015
2009-000156	ERR319412	N/A	4.3.1.1	unknown	unknown	Blood	2009	Asia	Southeast Asia	Cambodia	unknown	unknown	Wong et al, 2015
2009-000892	ERR319413	N/A	4.3.1.1	unknown	unknown	Blood	2009	Asia	Southeast Asia	Cambodia	unknown	unknown	Wong et al, 2015
2008-002960	ERR319414	N/A	4.3.1.1	unknown	unknown	Blood	2009	Asia	Southeast Asia	Cambodia	unknown	unknown	Wong et al, 2015
2009-004002	ERR319415	N/A	4.3.1.1	unknown	unknown	Blood	2009	Asia	Southeast Asia	Cambodia	unknown	unknown	Wong et al, 2015
2009-006046	ERR319416	N/A	4.3.1.1	unknown	unknown	Blood	2009	Asia	Southeast Asia	Cambodia	unknown	unknown	Wong et al, 2015
2009-007146	ERR319417	N/A	4.3.1.1	unknown	unknown	Blood	2009	Asia	Southeast Asia	Cambodia	unknown	unknown	Wong et al, 2015
2005-010587	ERR319418	N/A	3.2.1	unknown	unknown	Blood	2009	Asia	Southeast Asia	Cambodia	unknown	unknown	Wong et al, 2015
2003-008924	ERR319419	N/A	4.3.1.1	unknown	unknown	Blood	2009	Asia	Southeast Asia	Cambodia	unknown	unknown	Wong et al, 2015
2009-011935	ERR319420	N/A	4.3.1.1	unknown	unknown	Blood	2009	Asia	Southeast Asia	Cambodia	unknown	unknown	Wong et al, 2015
2009-012898	ERR319421	N/A	4.3.1.1	unknown	unknown	Blood	2009	Asia	Southeast Asia	Cambodia	unknown	unknown	Wong et al, 2015
2010-005196	ERR319422	N/A	4.3.1.1	unknown	unknown	Blood	2010	Asia	Southeast Asia	Cambodia	unknown	unknown	Wong et al, 2015

2010-011361	ERR319423	N/A	4.3.1.1	unknown	unknown	Blood	2010	Asia	Southeast Asia	Cambodia	unknown	unknown	Wong et al, 2015
2010-005872	ERR319424	N/A	4.3.1.1	unknown	unknown	Blood	2010	Asia	Southeast Asia	Cambodia	unknown	unknown	Wong et al, 2015
2006-011021	ERR319425	N/A	4.3.1.1	unknown	unknown	Blood	2010	Asia	Southeast Asia	Cambodia	unknown	unknown	Wong et al, 2015
2010-006551	ERR319426	N/A	4.3.1.1	unknown	unknown	Blood	2010	Asia	Southeast Asia	Cambodia	unknown	unknown	Wong et al, 2015
2007-013365	ERR319427	N/A	4.3.1.1	unknown	unknown	Blood	2010	Asia	Southeast Asia	Cambodia	unknown	unknown	Wong et al, 2015
2010-006866	ERR319428	N/A	4.3.1.1	unknown	unknown	Blood	2010	Asia	Southeast Asia	Cambodia	unknown	unknown	Wong et al, 2015
2007-012990	ERR319429	N/A	4.3.1.1	unknown	unknown	Blood	2010	Asia	Southeast Asia	Cambodia	unknown	unknown	Wong et al, 2015
2001-100510	ERR319431	N/A	4.3.1.1	unknown	unknown	Blood	2010	Asia	Southeast Asia	Cambodia	unknown	unknown	Wong et al, 2015
2010-007898	ERR319432	N/A	4.3.1.1	unknown	unknown	Blood	2010	Asia	Southeast Asia	Cambodia	unknown	unknown	Wong et al, 2015
2010-008154	ERR319433	N/A	4.3.1.1	unknown	unknown	Blood	2010	Asia	Southeast Asia	Cambodia	unknown	unknown	Wong et al, 2015
2010-007462	ERR319434	N/A	4.3.1.1	unknown	unknown	Blood	2010	Asia	Southeast Asia	Cambodia	unknown	unknown	Wong et al, 2015
2010-002605	ERR319435	N/A	4.3.1.1	unknown	unknown	Blood	2010	Asia	Southeast Asia	Cambodia	unknown	unknown	Wong et al, 2015
2010-009987	ERR319436	N/A	4.3.1.1	unknown	unknown	Blood	2010	Asia	Southeast Asia	Cambodia	unknown	unknown	Wong et al, 2015
2010-010162	ERR319437	N/A	4.3.1.1	unknown	unknown	Blood	2010	Asia	Southeast Asia	Cambodia	unknown	unknown	Wong et al, 2015
2010-002948	ERR319438	N/A	4.3.1.1	unknown	unknown	Blood	2010	Asia	Southeast Asia	Cambodia	unknown	unknown	Wong et al, 2015
2010-010972	ERR319439	N/A	4.3.1.1	unknown	unknown	Blood	2010	Asia	Southeast Asia	Cambodia	unknown	unknown	Wong et al, 2015
2010-011187	ERR319440	N/A	4.3.1.1	unknown	unknown	Blood	2010	Asia	Southeast Asia	Cambodia	unknown	unknown	Wong et al, 2015
2010-011712	ERR319441	N/A	4.3.1.1	unknown	unknown	Blood	2010	Asia	Southeast Asia	Cambodia	unknown	unknown	Wong et al, 2015
01-2011-000112	ERR319442	N/A	4.3.1.1	unknown	unknown	Blood	2011	Asia	Southeast Asia	Cambodia	unknown	unknown	Wong et al, 2015
01-2011-000483	ERR319443	N/A	4.3.1.1	unknown	unknown	Blood	2011	Asia	Southeast Asia	Cambodia	unknown	unknown	Wong et al, 2015
2005-009945	ERR319444	N/A	4.3.1.1	unknown	unknown	Blood	2011	Asia	Southeast Asia	Cambodia	unknown	unknown	Wong et al, 2015
2007-021515	ERR319445	N/A	4.3.1.1	unknown	unknown	Blood	2011	Asia	Southeast Asia	Cambodia	unknown	unknown	Wong et al, 2015
2006-007642	ERR319446	N/A	4.3.1.1	unknown	unknown	Blood	2011	Asia	Southeast Asia	Cambodia	unknown	unknown	Wong et al, 2015
2002-216622	ERR319447	N/A	4.3.1.1	unknown	unknown	Blood	2011	Asia	Southeast Asia	Cambodia	unknown	unknown	Wong et al, 2015
2010-004004	ERR319448	N/A	4.3.1.1	unknown	unknown	Blood	2011	Asia	Southeast Asia	Cambodia	unknown	unknown	Wong et al, 2015
01-2010-004103	ERR319449	N/A	4.3.1.1	unknown	unknown	Blood	2011	Asia	Southeast Asia	Cambodia	unknown	unknown	Wong et al, 2015
01-2011-000113	ERR319450	N/A	4.3.1.1	unknown	unknown	Blood	2011	Asia	Southeast Asia	Cambodia	unknown	unknown	Wong et al, 2015
01-2011-000309	ERR319451	N/A	4.3.1.1	unknown	unknown	Blood	2011	Asia	Southeast Asia	Cambodia	unknown	unknown	Wong et al, 2015
2010-021566	ERR319452	N/A	4.3.1.1	unknown	unknown	Blood	2011	Asia	Southeast Asia	Cambodia	unknown	unknown	Wong et al, 2015
2011-007445	ERR319453	N/A	4.3.1.1	unknown	unknown	Blood	2011	Asia	Southeast Asia	Cambodia	unknown	unknown	Wong et al, 2015
2005-011215	ERR319454	N/A	4.3.1.1	unknown	unknown	Blood	2011	Asia	Southeast Asia	Cambodia	unknown	unknown	Wong et al, 2015
2009-000374	ERR319455	N/A	4.3.1.1	unknown	unknown	Blood	2011	Asia	Southeast Asia	Cambodia	unknown	unknown	Wong et al, 2015
01-2011-001522	ERR319456	N/A	4.3.1.1	unknown	unknown	Blood	2011	Asia	Southeast Asia	Cambodia	unknown	unknown	Wong et al, 2015
2011-007660	ERR319457	N/A	4.3.1.1	unknown	unknown	Blood	2011	Asia	Southeast Asia	Cambodia	unknown	unknown	Wong et al, 2015
2011-007792	ERR319458	N/A	4.3.1.1	unknown	unknown	Blood	2011	Asia	Southeast Asia	Cambodia	unknown	unknown	Wong et al, 2015
2011-007930	ERR319459	N/A	4.3.1.1	unknown	unknown	Blood	2011	Asia	Southeast Asia	Cambodia	unknown	unknown	Wong et al, 2015
2006-008616	ERR319460	N/A	4.3.1.1	unknown	unknown	Blood	2011	Asia	Southeast Asia	Cambodia	unknown	unknown	Wong et al, 2015
2007-015726	ERR319461	N/A	4.3.1.1	unknown	unknown	Blood	2011	Asia	Southeast Asia	Cambodia	unknown	unknown	Wong et al, 2015
BC2011-1325	ERR319462	N/A	3.2.1	unknown	unknown	Blood	2011	Asia	Southeast Asia	Cambodia	unknown	unknown	Wong et al, 2015
BC2011-1311	ERR319463	N/A	4.3.1.1	unknown	unknown	Blood	2011	Asia	Southeast Asia	Cambodia	unknown	unknown	Wong et al, 2015
BC2011-1307	ERR319464	N/A	4.3.1.1	unknown	unknown	Blood	2011	Asia	Southeast Asia	Cambodia	unknown	unknown	Wong et al, 2015
BC2011-1305	ERR319465	N/A	4.3.1.1	unknown	unknown	Blood	2011	Asia	Southeast Asia	Cambodia	unknown	unknown	Wong et al, 2015
BC20111457	ERR319466	N/A	4.3.1.1	unknown	unknown	Blood	2011	Asia	Southeast Asia	Cambodia	unknown	unknown	Wong et al, 2015
BC2011-1461	ERR319467	N/A	4.3.1.1	unknown	unknown	Blood	2011	Asia	Southeast Asia	Cambodia	unknown	unknown	Wong et al, 2015
BC2011-1534	ERR319468	N/A	4.3.1.1	unknown	unknown	Blood	2011	Asia	Southeast Asia	Cambodia	unknown	unknown	Wong et al, 2015

BC2011-1612	ERR319469	N/A	4.3.1.1	unknown	unknown	Blood	2011	Asia	Southeast Asia	Cambodia	unknown	unknown	Wong et al, 2015
BC2011-1683	ERR319470	N/A	4.3.1.1	unknown	unknown	Blood	2011	Asia	Southeast Asia	Cambodia	unknown	unknown	Wong et al, 2015
BC2011-1739	ERR319471	N/A	4.3.1.1	unknown	unknown	Blood	2011	Asia	Southeast Asia	Cambodia	unknown	unknown	Wong et al, 2015
2011-018938	ERR319472	N/A	4.3.1.1	unknown	unknown	Blood	2012	Asia	Southeast Asia	Cambodia	unknown	unknown	Wong et al, 2015
2012-002759	ERR319473	N/A	4.3.1.1	unknown	unknown	Stool	2012	Asia	Southeast Asia	Cambodia	unknown	unknown	Wong et al, 2015
01-2010-002043	ERR319474	N/A	4.3.1.1	unknown	unknown	Blood	2012	Asia	Southeast Asia	Cambodia	unknown	unknown	Wong et al, 2015
01-2012-001846	ERR319475	N/A	4.3.1.1	unknown	unknown	Blood	2012	Asia	Southeast Asia	Cambodia	unknown	unknown	Wong et al, 2015
2012-007197	ERR319476	N/A	4.3.1.1	unknown	unknown	Blood	2012	Asia	Southeast Asia	Cambodia	unknown	unknown	Wong et al, 2015
01-2012-002175	ERR319477	N/A	4.3.1.1	unknown	unknown	Blood	2012	Asia	Southeast Asia	Cambodia	unknown	unknown	Wong et al, 2015
2012-008606	ERR319478	N/A	4.3.1.1	unknown	unknown	Blood	2012	Asia	Southeast Asia	Cambodia	unknown	unknown	Wong et al, 2015
2012-009077	ERR319479	N/A	4.3.1.1	unknown	unknown	Blood	2012	Asia	Southeast Asia	Cambodia	unknown	unknown	Wong et al, 2015
01-2012-002473	ERR319480	N/A	4.3.1.1	unknown	unknown	Blood	2012	Asia	Southeast Asia	Cambodia	unknown	unknown	Wong et al, 2015
01-2010-005183	ERR319481	N/A	4.3.1.1	unknown	unknown	Blood	2012	Asia	Southeast Asia	Cambodia	unknown	unknown	Wong et al, 2015
01-2012-002529	ERR319482	N/A	4.3.1.1	unknown	unknown	Blood	2012	Asia	Southeast Asia	Cambodia	unknown	unknown	Wong et al, 2015
2006-014301	ERR319483	N/A	4.3.1.1	unknown	unknown	Blood	2012	Asia	Southeast Asia	Cambodia	unknown	unknown	Wong et al, 2015
2012-009618	ERR319484	N/A	4.3.1.1	unknown	unknown	Blood	2012	Asia	Southeast Asia	Cambodia	unknown	unknown	Wong et al, 2015
2008-017452	ERR319485	N/A	4.3.1.1	unknown	unknown	Blood	2012	Asia	Southeast Asia	Cambodia	unknown	unknown	Wong et al, 2015
2012-010059	ERR319486	N/A	4.3.1.1	unknown	unknown	Blood	2012	Asia	Southeast Asia	Cambodia	unknown	unknown	Wong et al, 2015
2012-010060	ERR319487	N/A	4.3.1.1	unknown	unknown	Blood	2012	Asia	Southeast Asia	Cambodia	unknown	unknown	Wong et al, 2015
01-2012-002669	ERR319488	N/A	4.3.1.1	unknown	unknown	Blood	2012	Asia	Southeast Asia	Cambodia	unknown	unknown	Wong et al, 2015
2010-006045	ERR319489	N/A	4.3.1.1	unknown	unknown	Blood	2012	Asia	Southeast Asia	Cambodia	unknown	unknown	Wong et al, 2015
01-2012-002666	ERR319490	N/A	4.3.1.1	unknown	unknown	Blood	2012	Asia	Southeast Asia	Cambodia	unknown	unknown	Wong et al, 2015
2012-009913	ERR319492	N/A	4.3.1.1	unknown	unknown	Blood	2012	Asia	Southeast Asia	Cambodia	unknown	unknown	Wong et al, 2015
2005-010317	ERR319494	N/A	4.3.1.1	unknown	unknown	Blood	2012	Asia	Southeast Asia	Cambodia	unknown	unknown	Wong et al, 2015
2012-010406	ERR319495	N/A	4.3.1.1	unknown	unknown	Blood	2012	Asia	Southeast Asia	Cambodia	unknown	unknown	Wong et al, 2015
A140	ERR326598	N/A	2.1.8	unknown	unknown	Blood	2010	Asia	Southeast Asia	Indonesia	unknown	unknown	Wong et al, 2015
A268	ERR326599	N/A	4.1	unknown	unknown	Blood	2009	Asia	Southeast Asia	Indonesia	unknown	unknown	Wong et al, 2015
A270	ERR326600	N/A	1	unknown	unknown	Blood	2010	Asia	Southeast Asia	Indonesia	unknown	unknown	Wong et al, 2015
A271	ERR326601	N/A	4.1	unknown	unknown	Blood	2009	Asia	Southeast Asia	Indonesia	unknown	unknown	Wong et al, 2015
A272	ERR326602	N/A	4.1	unknown	unknown	Blood	2010	Asia	Southeast Asia	Indonesia	unknown	unknown	Wong et al, 2015
A273	ERR326603	N/A	4.1	unknown	unknown	Blood	2009	Asia	Southeast Asia	Indonesia	unknown	unknown	Wong et al, 2015
A274	ERR326604	N/A	3	unknown	unknown	Blood	2006	Asia	Southeast Asia	Indonesia	unknown	unknown	Wong et al, 2015
A275	ERR326605	N/A	4.1	unknown	unknown	Blood	2009	Asia	Southeast Asia	Indonesia	unknown	unknown	Wong et al, 2015
A276	ERR326606	N/A	4.1	unknown	unknown	Blood	2008	Asia	Southeast Asia	Indonesia	unknown	unknown	Wong et al, 2015
A277	ERR326607	N/A	4.1	unknown	unknown	Blood	2009	Asia	Southeast Asia	Indonesia	unknown	unknown	Wong et al, 2015
A278	ERR326608	N/A	4.1	unknown	unknown	Blood	2009	Asia	Southeast Asia	Indonesia	unknown	unknown	Wong et al, 2015
A281	ERR326610	N/A	3	unknown	unknown	Blood	2008	Asia	Southeast Asia	Indonesia	unknown	unknown	Wong et al, 2015
A282	ERR326611	N/A	3.1.2	unknown	unknown	Blood	2010	Asia	Southeast Asia	Indonesia	unknown	unknown	Wong et al, 2015
A283	ERR326612	N/A	4.1	unknown	unknown	Blood	2010	Asia	Southeast Asia	Indonesia	unknown	unknown	Wong et al, 2015
A284	ERR326613	N/A	3	unknown	unknown	Blood	2011	Asia	Southeast Asia	Indonesia	unknown	unknown	Wong et al, 2015
A285	ERR326614	N/A	4.1	unknown	unknown	Blood	2010	Asia	Southeast Asia	Indonesia	unknown	unknown	Wong et al, 2015
A286	ERR326615	N/A	2.1.6	unknown	unknown	Blood	2010	Asia	Southeast Asia	Indonesia	unknown	unknown	Wong et al, 2015
A287	ERR326616	N/A	3.1.2	unknown	unknown	Blood	2008	Asia	Southeast Asia	Indonesia	unknown	unknown	Wong et al, 2015
A340	ERR326618	N/A	2.1.9	unknown	unknown	Blood	2010	Asia	Southeast Asia	Indonesia	unknown	unknown	Wong et al, 2015
A341	ERR326619	N/A	3.1.2	unknown	unknown	Blood	2008	Asia	Southeast Asia	Indonesia	unknown	unknown	Wong et al, 2015

A344-1st	ERR326622	N/A	4.1	unknown	unknown	Blood	2010	Asia	Southeast Asia	Indonesia	unknown	unknown	Wong et al, 2015
A347	ERR326623	N/A	2.1.8	unknown	unknown	Blood	2007	Asia	Southeast Asia	Indonesia	unknown	unknown	Wong et al, 2015
A348	ERR326624	N/A	2.1.9	unknown	unknown	Blood	2012	Asia	Southeast Asia	Indonesia	unknown	unknown	Wong et al, 2015
A350	ERR326625	N/A	2.1.6	unknown	unknown	Blood	2010	Asia	Southeast Asia	Indonesia	unknown	unknown	Wong et al, 2015
A352	ERR326626	N/A	3	unknown	unknown	Blood	2010	Asia	Southeast Asia	Indonesia	unknown	unknown	Wong et al, 2015
A354	ERR326627	N/A	3	unknown	unknown	Blood	2011	Asia	Southeast Asia	Indonesia	unknown	unknown	Wong et al, 2015
A356	ERR326629	N/A	2.1.9	unknown	unknown	Blood	2010	Asia	Southeast Asia	Indonesia	unknown	unknown	Wong et al, 2015
A357	ERR326630	N/A	3.1.2	unknown	unknown	Blood	2009	Asia	Southeast Asia	Indonesia	unknown	unknown	Wong et al, 2015
A358	ERR326631	N/A	4.1	unknown	unknown	Blood	2009	Asia	Southeast Asia	Indonesia	unknown	unknown	Wong et al, 2015
A359	ERR326632	N/A	3.1.2	unknown	unknown	Blood	2010	Asia	Southeast Asia	Indonesia	unknown	unknown	Wong et al, 2015
A360	ERR326633	N/A	4.1	unknown	unknown	Blood	2009	Asia	Southeast Asia	Indonesia	unknown	unknown	Wong et al, 2015
A363	ERR326634	N/A	3.1.2	unknown	unknown	Blood	2009	Asia	Southeast Asia	Indonesia	unknown	unknown	Wong et al, 2015
A365	ERR326635	N/A	2.1.9	unknown	unknown	Blood	2007	Asia	Southeast Asia	Indonesia	unknown	unknown	Wong et al, 2015
A366	ERR326636	N/A	2.1.6	unknown	unknown	Blood	2009	Asia	Southeast Asia	Indonesia	unknown	unknown	Wong et al, 2015
A368	ERR326637	N/A	2.1.9	unknown	unknown	Blood	2009	Asia	Southeast Asia	Indonesia	unknown	unknown	Wong et al, 2015
A369	ERR326638	N/A	4.1	unknown	unknown	Blood	2008	Asia	Southeast Asia	Indonesia	unknown	unknown	Wong et al, 2015
A370	ERR326639	N/A	3.1.2	unknown	unknown	Blood	2010	Asia	Southeast Asia	Indonesia	unknown	unknown	Wong et al, 2015
A373	ERR326640	N/A	2.1.8	unknown	unknown	Blood	2008	Asia	Southeast Asia	Indonesia	unknown	unknown	Wong et al, 2015
A374	ERR326641	N/A	4.1	unknown	unknown	Blood	2008	Asia	Southeast Asia	Indonesia	unknown	unknown	Wong et al, 2015
A375	ERR326642	N/A	2.1.9	unknown	unknown	Blood	2005	Asia	Southeast Asia	Indonesia	unknown	unknown	Wong et al, 2015
A377	ERR326644	N/A	2.1.9	unknown	unknown	Blood	2010	Asia	Southeast Asia	Indonesia	unknown	unknown	Wong et al, 2015
A378	ERR326645	N/A	2.1.6	unknown	unknown	Blood	2008	Asia	Southeast Asia	Indonesia	unknown	unknown	Wong et al, 2015
A379	ERR326646	N/A	4.1	unknown	unknown	Blood	2010	Asia	Southeast Asia	Indonesia	unknown	unknown	Wong et al, 2015
A380	ERR326647	N/A	3	unknown	unknown	Blood	2006	Asia	Southeast Asia	Indonesia	unknown	unknown	Wong et al, 2015
A382	ERR326648	N/A	2.1.9	unknown	unknown	Blood	2009	Asia	Southeast Asia	Indonesia	unknown	unknown	Wong et al, 2015
A385	ERR326649	N/A	4.1	unknown	unknown	Blood	2012	Asia	Southeast Asia	Indonesia	unknown	unknown	Wong et al, 2015
A389	ERR326650	N/A	2.1.8	unknown	unknown	Blood	2006	Asia	Southeast Asia	Indonesia	unknown	unknown	Wong et al, 2015
A390	ERR326651	N/A	4.1	unknown	unknown	Blood	2007	Asia	Southeast Asia	Indonesia	unknown	unknown	Wong et al, 2015
A392	ERR326652	N/A	4.1	unknown	unknown	Blood	2010	Asia	Southeast Asia	Indonesia	unknown	unknown	Wong et al, 2015
A394	ERR326653	N/A	2.1.9	unknown	unknown	Blood	2007	Asia	Southeast Asia	Indonesia	unknown	unknown	Wong et al, 2015
A395	ERR326654	N/A	4.1	unknown	unknown	Blood	2009	Asia	Southeast Asia	Indonesia	unknown	unknown	Wong et al, 2015
A396	ERR326655	N/A	4.1	unknown	unknown	Blood	2009	Asia	Southeast Asia	Indonesia	unknown	unknown	Wong et al, 2015
A399	ERR326656	N/A	3.1.2	unknown	unknown	Blood	2009	Asia	Southeast Asia	Indonesia	unknown	unknown	Wong et al, 2015
KM1646	ERR326657	N/A	3.4	unknown	unknown	Blood	2010	Asia	Southeast Asia	Laos	unknown	unknown	Wong et al, 2015
KM1647	ERR326658	N/A	3.4	unknown	unknown	Blood	2010	Asia	Southeast Asia	Laos	unknown	unknown	Wong et al, 2015
KM1648	ERR326659	N/A	3.4	unknown	unknown	Blood	2010	Asia	Southeast Asia	Laos	unknown	unknown	Wong et al, 2015
LNT1197	ERR326660	N/A	2.3.4	unknown	unknown	Blood	2009	Asia	Southeast Asia	Laos	unknown	unknown	Wong et al, 2015
LNT12	ERR326661	N/A	3.4	unknown	unknown	Blood	2007	Asia	Southeast Asia	Laos	unknown	unknown	Wong et al, 2015
LNT13	ERR326662	N/A	3.4	unknown	unknown	Blood	2007	Asia	Southeast Asia	Laos	unknown	unknown	Wong et al, 2015
LNT1339	ERR326663	N/A	3.4	unknown	unknown	Blood	2010	Asia	Southeast Asia	Laos	unknown	unknown	Wong et al, 2015
LNT1360	ERR326664	N/A	2.4	unknown	unknown	Blood	2010	Asia	Southeast Asia	Laos	unknown	unknown	Wong et al, 2015
LNT1365	ERR326665	N/A	2.3.4	unknown	unknown	Blood	2010	Asia	Southeast Asia	Laos	unknown	unknown	Wong et al, 2015
LNT1367-3	ERR326666	N/A	2.3.4	unknown	unknown	Blood	2010	Asia	Southeast Asia	Laos	unknown	unknown	Wong et al, 2015
LNT1374	ERR326667	N/A	3.4	unknown	unknown	Blood	2010	Asia	Southeast Asia	Laos	unknown	unknown	Wong et al, 2015
LNT1377	ERR326668	N/A	2.3.4	unknown	unknown	Blood	2010	Asia	Southeast Asia	Laos	unknown	unknown	Wong et al, 2015

LNT1378	ERR326669	N/A	3.4	unknown	unknown	Blood	2010	Asia	Southeast Asia	Laos	unknown	unknown	Wong et al, 2015
LNT1426	ERR326670	N/A	3.4	unknown	unknown	Blood	2010	Asia	Southeast Asia	Laos	unknown	unknown	Wong et al, 2015
LNT1497	ERR326671	N/A	3.4	unknown	unknown	Blood	2010	Asia	Southeast Asia	Laos	unknown	unknown	Wong et al, 2015
LNT1480	ERR326672	N/A	2.2.3,2.2.2	unknown	unknown	Blood	2010	Asia	Southeast Asia	Laos	unknown	unknown	Wong et al, 2015
LNT1516	ERR326673	N/A	3.4	unknown	unknown	Blood	2010	Asia	Southeast Asia	Laos	unknown	unknown	Wong et al, 2015
LNT1542	ERR326674	N/A	3.4	unknown	unknown	Blood	2010	Asia	Southeast Asia	Laos	unknown	unknown	Wong et al, 2015
LNT266	ERR326676	N/A	3.4	unknown	unknown	Blood	2008	Asia	Southeast Asia	Laos	unknown	unknown	Wong et al, 2015
LNT279	ERR326677	N/A	3.4	unknown	unknown	Blood	2008	Asia	Southeast Asia	Laos	unknown	unknown	Wong et al, 2015
LNT330	ERR326678	N/A	2.4	unknown	unknown	Blood	2008	Asia	Southeast Asia	Laos	unknown	unknown	Wong et al, 2015
LNT366	ERR326679	N/A	3.4	unknown	unknown	Blood	2008	Asia	Southeast Asia	Laos	unknown	unknown	Wong et al, 2015
LNT375	ERR326680	N/A	3.4	unknown	unknown	Blood	2008	Asia	Southeast Asia	Laos	unknown	unknown	Wong et al, 2015
LNT565	ERR326681	N/A	3.4	unknown	unknown	Blood	2008	Asia	Southeast Asia	Laos	unknown	unknown	Wong et al, 2015
LNT609	ERR326682	N/A	3.4	unknown	unknown	Blood	2008	Asia	Southeast Asia	Laos	unknown	unknown	Wong et al, 2015
LNT659	ERR326683	N/A	3.4	unknown	unknown	Blood	2008	Asia	Southeast Asia	Laos	unknown	unknown	Wong et al, 2015
LNT662	ERR326684	N/A	2.3.4	unknown	unknown	Blood	2008	Asia	Southeast Asia	Laos	unknown	unknown	Wong et al, 2015
LNT666	ERR326685	N/A	3.4	unknown	unknown	Blood	2008	Asia	Southeast Asia	Laos	unknown	unknown	Wong et al, 2015
LNT670	ERR326686	N/A	3.4	unknown	unknown	Blood	2008	Asia	Southeast Asia	Laos	unknown	unknown	Wong et al, 2015
LNT705	ERR326687	N/A	4.1	unknown	unknown	Blood	2008	Asia	Southeast Asia	Laos	unknown	unknown	Wong et al, 2015
LNT72	ERR326688	N/A	2.3.4	unknown	unknown	Blood	2007	Asia	Southeast Asia	Laos	unknown	unknown	Wong et al, 2015
LNT722	ERR326689	N/A	3.4	unknown	unknown	Blood	2008	Asia	Southeast Asia	Laos	unknown	unknown	Wong et al, 2015
2008-003680	ERR331205	N/A	4.3.1.1	unknown	unknown	Blood	2012	Asia	Southeast Asia	Cambodia	unknown	unknown	Wong et al, 2015
01-2011-002432	ERR331206	N/A	4.3.1.1	unknown	unknown	Blood	2012	Asia	Southeast Asia	Cambodia	unknown	unknown	Wong et al, 2015
2007-019648	ERR331207	N/A	4.3.1.1	unknown	unknown	Blood	2012	Asia	Southeast Asia	Cambodia	unknown	unknown	Wong et al, 2015
2012-010389	ERR331208	N/A	4.3.1.1	unknown	unknown	Blood	2012	Asia	Southeast Asia	Cambodia	unknown	unknown	Wong et al, 2015
A143	ERR331212	N/A	2.1.3	unknown	unknown	Blood	2008	Asia	Southeast Asia	Indonesia	unknown	unknown	Wong et al, 2015
A149	ERR331214	N/A	2.1.1	unknown	unknown	Blood	2006	Asia	Southeast Asia	Indonesia	unknown	unknown	Wong et al, 2015
A150	ERR331215	N/A	3	unknown	unknown	Blood	2006	Asia	Southeast Asia	Indonesia	unknown	unknown	Wong et al, 2015
A153	ERR331216	N/A	2.1.8	unknown	unknown	Blood	2010	Asia	Southeast Asia	Indonesia	unknown	unknown	Wong et al, 2015
A154	ERR331217	N/A	2.1.9	unknown	unknown	Blood	2009	Asia	Southeast Asia	Indonesia	unknown	unknown	Wong et al, 2015
A155	ERR331218	N/A	2.1.1	unknown	unknown	Blood	2006	Asia	Southeast Asia	Indonesia	unknown	unknown	Wong et al, 2015
A156	ERR331219	N/A	3	unknown	unknown	Blood	2007	Asia	Southeast Asia	Indonesia	unknown	unknown	Wong et al, 2015
A158	ERR331220	N/A	3.1.2	unknown	unknown	Blood	2009	Asia	Southeast Asia	Indonesia	unknown	unknown	Wong et al, 2015
A160	ERR331221	N/A	2.1.1	unknown	unknown	Blood	2006	Asia	Southeast Asia	Indonesia	unknown	unknown	Wong et al, 2015
A173	ERR331224	N/A	2.1.9	unknown	unknown	Blood	2009	Asia	Southeast Asia	Indonesia	unknown	unknown	Wong et al, 2015
A174	ERR331225	N/A	2.1.3	unknown	unknown	Blood	2011	Asia	Southeast Asia	Indonesia	unknown	unknown	Wong et al, 2015
A178	ERR331226	N/A	3.1.2	unknown	unknown	Blood	2009	Asia	Southeast Asia	Indonesia	unknown	unknown	Wong et al, 2015
A180	ERR331227	N/A	4.1	unknown	unknown	Blood	2009	Asia	Southeast Asia	Indonesia	unknown	unknown	Wong et al, 2015
A235	ERR331229	N/A	3	unknown	unknown	Blood	2009	Asia	Southeast Asia	Indonesia	unknown	unknown	Wong et al, 2015
A236	ERR331230	N/A	4.1	unknown	unknown	Blood	2009	Asia	Southeast Asia	Indonesia	unknown	unknown	Wong et al, 2015
A237	ERR331231	N/A	3	unknown	unknown	Blood	2010	Asia	Southeast Asia	Indonesia	unknown	unknown	Wong et al, 2015
A238	ERR331232	N/A	4.1	unknown	unknown	Blood	2009	Asia	Southeast Asia	Indonesia	unknown	unknown	Wong et al, 2015
A239	ERR331233	N/A	3.1.2	unknown	unknown	Blood	2009	Asia	Southeast Asia	Indonesia	unknown	unknown	Wong et al, 2015
A240	ERR331234	N/A	4.1	unknown	unknown	Blood	2010	Asia	Southeast Asia	Indonesia	unknown	unknown	Wong et al, 2015
A243	ERR331236	N/A	4.1	unknown	unknown	Blood	2011	Asia	Southeast Asia	Indonesia	unknown	unknown	Wong et al, 2015
A244	ERR331237	N/A	3	unknown	unknown	Blood	2011	Asia	Southeast Asia	Indonesia	unknown	unknown	Wong et al, 2015

A245	ERR331238	N/A	4.1	unknown	unknown	Blood	2009	Asia	Southeast Asia	Indonesia	unknown	unknown	Wong et al, 2015
A246	ERR331239	N/A	4.1	unknown	unknown	Blood	2010	Asia	Southeast Asia	Indonesia	unknown	unknown	Wong et al, 2015
A247	ERR331240	N/A	4.1	unknown	unknown	Blood	2010	Asia	Southeast Asia	Indonesia	unknown	unknown	Wong et al, 2015
A248	ERR331241	N/A	3	unknown	unknown	Blood	2009	Asia	Southeast Asia	Indonesia	unknown	unknown	Wong et al, 2015
A249	ERR331242	N/A	3.1.2	unknown	unknown	Blood	2010	Asia	Southeast Asia	Indonesia	unknown	unknown	Wong et al, 2015
A250	ERR331243	N/A	4.1	unknown	unknown	Blood	2009	Asia	Southeast Asia	Indonesia	unknown	unknown	Wong et al, 2015
A252	ERR331244	N/A	4.1	unknown	unknown	Blood	2011	Asia	Southeast Asia	Indonesia	unknown	unknown	Wong et al, 2015
A253	ERR331245	N/A	2.1.6	unknown	unknown	Blood	2009	Asia	Southeast Asia	Indonesia	unknown	unknown	Wong et al, 2015
A255	ERR331247	N/A	4.1	unknown	unknown	Blood	2011	Asia	Southeast Asia	Indonesia	unknown	unknown	Wong et al, 2015
A256	ERR331248	N/A	3	unknown	unknown	Blood	2010	Asia	Southeast Asia	Indonesia	unknown	unknown	Wong et al, 2015
A257	ERR331249	N/A	3	unknown	unknown	Blood	2010	Asia	Southeast Asia	Indonesia	unknown	unknown	Wong et al, 2015
A258	ERR331250	N/A	4.1	unknown	unknown	Blood	2009	Asia	Southeast Asia	Indonesia	unknown	unknown	Wong et al, 2015
A259	ERR331251	N/A	4.1	unknown	unknown	Blood	2010	Asia	Southeast Asia	Indonesia	unknown	unknown	Wong et al, 2015
A261	ERR331252	N/A	2.1.6	unknown	unknown	Blood	2009	Asia	Southeast Asia	Indonesia	unknown	unknown	Wong et al, 2015
A262	ERR331253	N/A	2.1.6	unknown	unknown	Blood	2010	Asia	Southeast Asia	Indonesia	unknown	unknown	Wong et al, 2015
A263	ERR331254	N/A	4.1	unknown	unknown	Blood	2009	Asia	Southeast Asia	Indonesia	unknown	unknown	Wong et al, 2015
A265	ERR331256	N/A	4.1	unknown	unknown	Blood	2010	Asia	Southeast Asia	Indonesia	unknown	unknown	Wong et al, 2015
A266	ERR331257	N/A	4.1	unknown	unknown	Blood	2010	Asia	Southeast Asia	Indonesia	unknown	unknown	Wong et al, 2015
A267	ERR331258	N/A	2.1.6	unknown	unknown	Blood	2008	Asia	Southeast Asia	Indonesia	unknown	unknown	Wong et al, 2015
100949	ERR331260	N/A	4.2.2	unknown	unknown	Unknown	2011	Australia & Oceania	Oceania	Fiji	unknown	unknown	Wong et al, 2015
102196	ERR331261	N/A	4.2.2	unknown	unknown	Unknown	2011	Australia & Oceania	Oceania	Fiji	unknown	unknown	Wong et al, 2015
103643	ERR331262	N/A	4.2.1	unknown	unknown	Unknown	2011	Australia & Oceania	Oceania	Fiji	unknown	unknown	Wong et al, 2015
106774	ERR331263	N/A	4.2.2	unknown	unknown	Unknown	2012	Australia & Oceania	Oceania	Fiji	unknown	unknown	Wong et al, 2015
107123	ERR331264	N/A	4.2.1	unknown	unknown	Unknown	2012	Australia & Oceania	Oceania	Fiji	unknown	unknown	Wong et al, 2015
107364	ERR331265	N/A	4.2.2	unknown	unknown	Unknown	2012	Australia & Oceania	Oceania	Fiji	unknown	unknown	Wong et al, 2015
108891	ERR331266	N/A	4.2.2	unknown	unknown	Unknown	2012	Australia & Oceania	Oceania	Fiji	unknown	unknown	Wong et al, 2015
108910	ERR331267	N/A	4.2.2	unknown	unknown	Unknown	2012	Australia & Oceania	Oceania	Fiji	unknown	unknown	Wong et al, 2015
109520	ERR331268	N/A	4.2.2	unknown	unknown	Unknown	2012	Australia & Oceania	Oceania	Fiji	unknown	unknown	Wong et al, 2015
110829	ERR331269	N/A	4.2.2	unknown	unknown	Unknown	2012	Australia & Oceania	Oceania	Fiji	unknown	unknown	Wong et al, 2015
B1271	ERR331270	N/A	4.2.1	unknown	unknown	Blood	2012	Australia & Oceania	Oceania	Fiji	unknown	unknown	Wong et al, 2015
B1305	ERR331271	N/A	4.2.2	unknown	unknown	Blood	2012	Australia & Oceania	Oceania	Fiji	unknown	unknown	Wong et al, 2015
B1357	ERR331273	N/A	4.2.2	unknown	unknown	Blood	2012	Australia & Oceania	Oceania	Fiji	unknown	unknown	Wong et al, 2015
B1472	ERR331274	N/A	4.2.2	unknown	unknown	Blood	2012	Australia & Oceania	Oceania	Fiji	unknown	unknown	Wong et al, 2015
B1501	ERR331275	N/A	4.2.2	unknown	unknown	Blood	2012	Australia & Oceania	Oceania	Fiji	unknown	unknown	Wong et al, 2015
B1502	ERR331276	N/A	4.2.2	unknown	unknown	Blood	2012	Australia & Oceania	Oceania	Fiji	unknown	unknown	Wong et al, 2015

B1542	ERR331278	N/A	4.2.2	unknown	unknown	Blood	2012	Australia & Oceania	Oceania	Fiji	unknown	unknown	Wong et al, 2015
B1563	ERR331279	N/A	4.2.2	unknown	unknown	Blood	2012	Australia & Oceania	Oceania	Fiji	unknown	unknown	Wong et al, 2015
B1596	ERR331280	N/A	4.2.2	unknown	unknown	Blood	2012	Australia & Oceania	Oceania	Fiji	unknown	unknown	Wong et al, 2015
B1616	ERR331281	N/A	4.2.2	unknown	unknown	Blood	2012	Australia & Oceania	Oceania	Fiji	unknown	unknown	Wong et al, 2015
B1628	ERR331282	N/A	4.2.2	unknown	unknown	Blood	2012	Australia & Oceania	Oceania	Fiji	unknown	unknown	Wong et al, 2015
B2667	ERR331283	N/A	4.2.2	unknown	unknown	Unknown	2008	Australia & Oceania	Oceania	Fiji	unknown	unknown	Wong et al, 2015
B4381	ERR331284	N/A	4.2.2	unknown	unknown	Unknown	2008	Australia & Oceania	Oceania	Fiji	unknown	unknown	Wong et al, 2015
B4387	ERR331285	N/A	4.2.2	unknown	unknown	Unknown	2008	Australia & Oceania	Oceania	Fiji	unknown	unknown	Wong et al, 2015
P749	ERR331287	N/A	4.2.2	unknown	unknown	Unknown	2008	Australia & Oceania	Oceania	Fiji	unknown	unknown	Wong et al, 2015
W478	ERR331288	N/A	4.2.2	unknown	unknown	Rectal swab	2012	Australia & Oceania	Oceania	Fiji	unknown	unknown	Wong et al, 2015
W794	ERR331289	N/A	4.2.2	unknown	unknown	Stool	2012	Australia & Oceania	Oceania	Fiji	unknown	unknown	Wong et al, 2015
1102	ERR331290	N/A	4.3.1.1	unknown	unknown	Blood	2011	Asia	South Asia	Bangladesh	unknown	unknown	Wong et al, 2015
1154	ERR331291	N/A	4.3.1.3	unknown	unknown	Blood	2011	Asia	South Asia	Bangladesh	unknown	unknown	Wong et al, 2015
1404	ERR331292	N/A	4.3.1.1	unknown	unknown	Blood	2011	Asia	South Asia	Bangladesh	unknown	unknown	Wong et al, 2015
1499	ERR331293	N/A	4.3.1.1	unknown	unknown	Blood	2011	Asia	South Asia	Bangladesh	unknown	unknown	Wong et al, 2015
1553	ERR331294	N/A	4.3.1.3.Bdq	unknown	unknown	Blood	2011	Asia	South Asia	Bangladesh	unknown	unknown	Wong et al, 2015
1607	ERR331295	N/A	4.3.1.3.Bdq	unknown	unknown	Blood	2011	Asia	South Asia	Bangladesh	unknown	unknown	Wong et al, 2015
1703	ERR331296	N/A	4.3.1.1	unknown	unknown	Blood	2008	Asia	South Asia	Bangladesh	unknown	unknown	Wong et al, 2015
1707	ERR331297	N/A	4.3.1.1	unknown	unknown	Blood	2012	Asia	South Asia	Bangladesh	unknown	unknown	Wong et al, 2015
LNT757	ERR331298	N/A	2.3.4	unknown	unknown	Blood	2009	Asia	Southeast Asia	Laos	unknown	unknown	Wong et al, 2015
LNT899	ERR331300	N/A	3.4	unknown	unknown	Blood	2009	Asia	Southeast Asia	Laos	unknown	unknown	Wong et al, 2015
LPB866	ERR331301	N/A	4.3.1.1	unknown	unknown	Blood	2008	Asia	Southeast Asia	Laos	unknown	unknown	Wong et al, 2015
ST716	ERR331302	N/A	3.4	unknown	unknown	Blood	2008	Asia	Southeast Asia	Laos	unknown	unknown	Wong et al, 2015
SV108	ERR331303	N/A	3.2.1	unknown	unknown	Blood	2008	Asia	Southeast Asia	Laos	unknown	unknown	Wong et al, 2015
SV170	ERR331304	N/A	3.2.1	unknown	unknown	Blood	2009	Asia	Southeast Asia	Laos	unknown	unknown	Wong et al, 2015
SV171	ERR331305	N/A	3.2.1	unknown	unknown	Blood	2009	Asia	Southeast Asia	Laos	unknown	unknown	Wong et al, 2015
SV179	ERR331306	N/A	3.2.1	unknown	unknown	Blood	2009	Asia	Southeast Asia	Laos	unknown	unknown	Wong et al, 2015
SV200	ERR331307	N/A	3.4	unknown	unknown	Blood	2009	Asia	Southeast Asia	Laos	unknown	unknown	Wong et al, 2015
SV211	ERR331308	N/A	3.2.1	unknown	unknown	Blood	2009	Asia	Southeast Asia	Laos	unknown	unknown	Wong et al, 2015
SV357	ERR331309	N/A	3.5.2	unknown	unknown	Blood	2009	Asia	Southeast Asia	Laos	unknown	unknown	Wong et al, 2015
SV430	ERR331310	N/A	2.2.3,2.2.2	unknown	unknown	Blood	2009	Asia	Southeast Asia	Laos	unknown	unknown	Wong et al, 2015
SV431	ERR331311	N/A	3.2.1	unknown	unknown	Blood	2009	Asia	Southeast Asia	Laos	unknown	unknown	Wong et al, 2015
SV500	ERR331312	N/A	3.5.2	unknown	unknown	Blood	2010	Asia	Southeast Asia	Laos	unknown	unknown	Wong et al, 2015
SV547	ERR331313	N/A	3.5.2	unknown	unknown	Blood	2010	Asia	Southeast Asia	Laos	unknown	unknown	Wong et al, 2015
SV552	ERR331314	N/A	3.5.2	unknown	unknown	Blood	2010	Asia	Southeast Asia	Laos	unknown	unknown	Wong et al, 2015
SV610	ERR331315	N/A	3.5.2	unknown	unknown	Blood	2010	Asia	Southeast Asia	Laos	unknown	unknown	Wong et al, 2015
UI10006	ERR331316	N/A	2.3.4	unknown	unknown	Blood	2007	Asia	Southeast Asia	Laos	unknown	unknown	Wong et al, 2015
UI10788	ERR331317	N/A	2.2.3,2.2.2	unknown	unknown	Blood	2007	Asia	Southeast Asia	Laos	unknown	unknown	Wong et al, 2015

UI11483	ERR331318	N/A	3.4	unknown	unknown	Blood	2008	Asia	Southeast Asia	Laos	unknown	unknown	Wong et al, 2015
UI11562	ERR331319	N/A	4.1	unknown	unknown	Blood	2008	Asia	Southeast Asia	Laos	unknown	unknown	Wong et al, 2015
UI11955	ERR331320	N/A	3.4	unknown	unknown	Blood	2008	Asia	Southeast Asia	Laos	unknown	unknown	Wong et al, 2015
UI12162	ERR331321	N/A	3.2.1	unknown	unknown	Blood	2008	Asia	Southeast Asia	Laos	unknown	unknown	Wong et al, 2015
UI13529-2	ERR331322	N/A	3.4	unknown	unknown	Blood	2009	Asia	Southeast Asia	Laos	unknown	unknown	Wong et al, 2015
UI13599	ERR331323	N/A	3.2.1	unknown	unknown	Blood	2009	Asia	Southeast Asia	Laos	unknown	unknown	Wong et al, 2015
UI13797	ERR331324	N/A	3.2.1	unknown	unknown	Blood	2009	Asia	Southeast Asia	Laos	unknown	unknown	Wong et al, 2015
UI13823	ERR331325	N/A	4.3.1.1	unknown	unknown	Blood	2009	Asia	Southeast Asia	Laos	unknown	unknown	Wong et al, 2015
UI14191/3	ERR331326	N/A	3.4	unknown	unknown	Blood	2009	Asia	Southeast Asia	Laos	unknown	unknown	Wong et al, 2015
UI14598	ERR331327	N/A	2.2.3,2.2.2	unknown	unknown	Blood	2009	Asia	Southeast Asia	Laos	unknown	unknown	Wong et al, 2015
UI15075	ERR331328	N/A	4.3.1.1	unknown	unknown	Blood	2008	Asia	Southeast Asia	Laos	unknown	unknown	Wong et al, 2015
UI16161	ERR331329	N/A	3.4	unknown	unknown	Blood	2010	Asia	Southeast Asia	Laos	unknown	unknown	Wong et al, 2015
UI16704	ERR331330	N/A	4.3.1.1	unknown	unknown	Blood	2010	Asia	Southeast Asia	Laos	unknown	unknown	Wong et al, 2015
UI17187	ERR331331	N/A	4.1	unknown	unknown	Blood	2010	Asia	Southeast Asia	Laos	unknown	unknown	Wong et al, 2015
UI17614	ERR331332	N/A	3.2.1	unknown	unknown	Blood	2010	Asia	Southeast Asia	Laos	unknown	unknown	Wong et al, 2015
UI3398	ERR331333	N/A	4.3.1.1	unknown	unknown	Blood	2003	Asia	Southeast Asia	Laos	unknown	unknown	Wong et al, 2015
UI3446	ERR331334	N/A	3	unknown	unknown	Blood	2003	Asia	Southeast Asia	Laos	unknown	unknown	Wong et al, 2015
UI3452	ERR331335	N/A	4.3.1.1	unknown	unknown	Blood	2003	Asia	Southeast Asia	Laos	unknown	unknown	Wong et al, 2015
UI3492	ERR331336	N/A	4.3.1.1	unknown	unknown	Blood	2003	Asia	Southeast Asia	Laos	unknown	unknown	Wong et al, 2015
UI3564	ERR331337	N/A	4.1	unknown	unknown	Blood	2003	Asia	Southeast Asia	Laos	unknown	unknown	Wong et al, 2015
UI3608	ERR331339	N/A	2.2	unknown	unknown	Blood	2003	Asia	Southeast Asia	Laos	unknown	unknown	Wong et al, 2015
UI3744	ERR331340	N/A	3.4	unknown	unknown	Blood	2003	Asia	Southeast Asia	Laos	unknown	unknown	Wong et al, 2015
UI3753	ERR331341	N/A	3.2.1	unknown	unknown	Blood	2003	Asia	Southeast Asia	Laos	unknown	unknown	Wong et al, 2015
UI3816	ERR331342	N/A	3.4	unknown	unknown	Blood	2003	Asia	Southeast Asia	Laos	unknown	unknown	Wong et al, 2015
UI3862	ERR331343	N/A	3.4	unknown	unknown	Blood	2003	Asia	Southeast Asia	Laos	unknown	unknown	Wong et al, 2015
UI3915	ERR331344	N/A	4.3.1.1	unknown	unknown	Blood	2003	Asia	Southeast Asia	Laos	unknown	unknown	Wong et al, 2015
UI3930	ERR331345	N/A	3.4	unknown	unknown	Blood	2003	Asia	Southeast Asia	Laos	unknown	unknown	Wong et al, 2015
UI4389	ERR331346	N/A	4.3.1.1	unknown	unknown	Blood	2003	Asia	Southeast Asia	Laos	unknown	unknown	Wong et al, 2015
UI4639	ERR331347	N/A	4.3.1.1	unknown	unknown	Blood	2003	Asia	Southeast Asia	Laos	unknown	unknown	Wong et al, 2015
UI4692	ERR331348	N/A	2.2.3,2.2.2	unknown	unknown	Blood	2004	Asia	Southeast Asia	Laos	unknown	unknown	Wong et al, 2015
UI4940	ERR331349	N/A	4.1	unknown	unknown	Blood	2004	Asia	Southeast Asia	Laos	unknown	unknown	Wong et al, 2015
UI5026	ERR331350	N/A	3.4	unknown	unknown	Blood	2004	Asia	Southeast Asia	Laos	unknown	unknown	Wong et al, 2015
UI5106	ERR331351	N/A	4.3.1.1	unknown	unknown	Blood	2004	Asia	Southeast Asia	Laos	unknown	unknown	Wong et al, 2015
UI5154	ERR331352	N/A	4.3.1.1	unknown	unknown	Blood	2004	Asia	Southeast Asia	Laos	unknown	unknown	Wong et al, 2015
UI5275	ERR331353	N/A	3.2.1	unknown	unknown	Blood	2004	Asia	Southeast Asia	Laos	unknown	unknown	Wong et al, 2015
UI5312	ERR331354	N/A	3.2.1	unknown	unknown	Blood	2004	Asia	Southeast Asia	Laos	unknown	unknown	Wong et al, 2015
UI5446	ERR331355	N/A	3.4	unknown	unknown	Blood	2004	Asia	Southeast Asia	Laos	unknown	unknown	Wong et al, 2015
UI5557	ERR331356	N/A	3.4	unknown	unknown	Blood	2004	Asia	Southeast Asia	Laos	unknown	unknown	Wong et al, 2015
UI5669	ERR331357	N/A	4.1	unknown	unknown	Blood	2004	Asia	Southeast Asia	Laos	unknown	unknown	Wong et al, 2015
UI5685-2	ERR331358	N/A	4.1	unknown	unknown	Blood	2004	Asia	Southeast Asia	Laos	unknown	unknown	Wong et al, 2015
UX229	ERR331359	N/A	3.4	unknown	unknown	Blood	2009	Asia	Southeast Asia	Laos	unknown	unknown	Wong et al, 2015
UX305	ERR331360	N/A	3.4	unknown	unknown	Blood	2009	Asia	Southeast Asia	Laos	unknown	unknown	Wong et al, 2015
UX309	ERR331361	N/A	3.4	unknown	unknown	Blood	2009	Asia	Southeast Asia	Laos	unknown	unknown	Wong et al, 2015
XK1682	ERR331363	N/A	3.2.1	unknown	unknown	Blood	2010	Asia	Southeast Asia	Laos	unknown	unknown	Wong et al, 2015
XK1684	ERR331364	N/A	3.2.1	unknown	unknown	Blood	2010	Asia	Southeast Asia	Laos	unknown	unknown	Wong et al, 2015

XN211	ERR331365	N/A	3.4	unknown	unknown	Blood	2010	Asia	Southeast Asia	Laos	unknown	unknown	Wong et al, 2015
60434	ERR331366	N/A	2.2.2	unknown	unknown	Blood	2006	Africa	East Africa	Tanzania	unknown	unknown	Wong et al, 2015
129-0242-M	ERR331368	N/A	4.3.1.1.EA1	unknown	unknown	Blood	2008	Africa	East Africa	Tanzania	unknown	unknown	Wong et al, 2015
129-0289-M	ERR331369	N/A	4.3.1.1.EA1	unknown	unknown	Blood	2008	Africa	East Africa	Tanzania	unknown	unknown	Wong et al, 2015
61025	ERR331370	N/A	2.2	unknown	unknown	Blood	2006	Africa	East Africa	Tanzania	unknown	unknown	Wong et al, 2015
63714	ERR331371	N/A	3.3.1	unknown	unknown	Blood	2007	Africa	East Africa	Tanzania	unknown	unknown	Wong et al, 2015
129-0327-M	ERR331372	N/A	4.3.1.1.EA1	unknown	unknown	Blood	2008	Africa	East Africa	Tanzania	unknown	unknown	Wong et al, 2015
129-0177-M	ERR331373	N/A	3.1	unknown	unknown	Blood	2007	Africa	East Africa	Tanzania	unknown	unknown	Wong et al, 2015
129-0268-M	ERR331374	N/A	4.3.1.1.EA1	unknown	unknown	Blood	2008	Africa	East Africa	Tanzania	unknown	unknown	Wong et al, 2015
129-0339-M	ERR331375	N/A	4.3.1.1.EA1	unknown	unknown	Blood	2008	Africa	East Africa	Tanzania	unknown	unknown	Wong et al, 2015
129-0303-M	ERR331376	N/A	4.3.1.1.EA1	unknown	unknown	Blood	2008	Africa	East Africa	Tanzania	unknown	unknown	Wong et al, 2015
129-0257-M	ERR331377	N/A	4.3.1.1.EA1	unknown	unknown	Blood	2008	Africa	East Africa	Tanzania	unknown	unknown	Wong et al, 2015
129-0230-K	ERR331378	N/A	4.3.1.1.EA1	unknown	unknown	Blood	2008	Africa	East Africa	Tanzania	unknown	unknown	Wong et al, 2015
129-0254-M	ERR331379	N/A	4.3.1.1.EA1	unknown	unknown	Blood	2008	Africa	East Africa	Tanzania	unknown	unknown	Wong et al, 2015
129-0238-M	ERR331380	N/A	4.3.1.1.EA1	unknown	unknown	Blood	2008	Africa	East Africa	Tanzania	unknown	unknown	Wong et al, 2015
62717	ERR331381	N/A	2.2	unknown	unknown	Blood	2006	Africa	East Africa	Tanzania	unknown	unknown	Wong et al, 2015
H062640481	ERR331382	N/A	4.3.1.1	unknown	unknown	Blood	2006	Asia	Western Asia	Iraq	unknown	unknown	Wong et al, 2015
H094620494	ERR331383	N/A	4.3.1.1	unknown	unknown	Blood	2009	Asia	Western Asia	Iraq	unknown	unknown	Wong et al, 2015
H083520583	ERR331384	N/A	4.3.1.1	unknown	unknown	Blood	2008	Asia	Western Asia	Iraq	unknown	unknown	Wong et al, 2015
H104280445	ERR331385	N/A	4.3.1.1	unknown	unknown	Blood	2010	Asia	Western Asia	Iraq	unknown	unknown	Wong et al, 2015
H075080543	ERR331386	N/A	4.3.1.1	unknown	unknown	Blood	2007	Asia	Western Asia	Iraq	unknown	unknown	Wong et al, 2015
H094420550	ERR331387	N/A	4.3.1.1	unknown	unknown	Stool	2009	Asia	Western Asia	Iraq	unknown	unknown	Wong et al, 2015
H084140247	ERR331388	N/A	4.3.1.1	unknown	unknown	Blood	2008	Asia	Western Asia	Iraq	unknown	unknown	Wong et al, 2015
H094320403	ERR331389	N/A	4.3.1.1	unknown	unknown	Blood	2009	Asia	Western Asia	Iraq	unknown	unknown	Wong et al, 2015
H084260341	ERR331390	N/A	4.3.1.1	unknown	unknown	Blood	2008	Asia	Western Asia	Iraq	unknown	unknown	Wong et al, 2015
1738	ERR337977	N/A	4.3.1.3.Bdq	unknown	unknown	Blood	2012	Asia	South Asia	Bangladesh	unknown	unknown	Wong et al, 2015
1877	ERR337978	N/A	4.3.1.3	unknown	unknown	Blood	2012	Asia	South Asia	Bangladesh	unknown	unknown	Wong et al, 2015
2181	ERR337979	N/A	3.3.2	unknown	unknown	Blood	2012	Asia	South Asia	Bangladesh	unknown	unknown	Wong et al, 2015
TY019	ERR337981	N/A	3.3.2	unknown	unknown	Blood	2012	Asia	South Asia	Bangladesh	unknown	unknown	Wong et al, 2015
TY029	ERR337982	N/A	3.3.2	unknown	unknown	Blood	2012	Asia	South Asia	Bangladesh	unknown	unknown	Wong et al, 2015
TY032	ERR337983	N/A	3.3.2	unknown	unknown	Blood	2012	Asia	South Asia	Bangladesh	unknown	unknown	Wong et al, 2015
TY053	ERR337984	N/A	4.3.1.1	unknown	unknown	Blood	2012	Asia	South Asia	Bangladesh	unknown	unknown	Wong et al, 2015
TY063	ERR337985	N/A	3.3.2	unknown	unknown	Blood	2012	Asia	South Asia	Bangladesh	unknown	unknown	Wong et al, 2015
TY068	ERR337986	N/A	3.2.2	unknown	unknown	Blood	2012	Asia	South Asia	Bangladesh	unknown	unknown	Wong et al, 2015
TY107	ERR337987	N/A	4.3.1.1	unknown	unknown	Blood	2012	Asia	South Asia	Bangladesh	unknown	unknown	Wong et al, 2015
TY108	ERR337988	N/A	3.3.2	unknown	unknown	Blood	2012	Asia	South Asia	Bangladesh	unknown	unknown	Wong et al, 2015
TY121	ERR337989	N/A	4.3.1.1	unknown	unknown	Blood	2012	Asia	South Asia	Bangladesh	unknown	unknown	Wong et al, 2015
TY132	ERR337990	N/A	4.3.1.1	unknown	unknown	Blood	2012	Asia	South Asia	Bangladesh	unknown	unknown	Wong et al, 2015
TY138	ERR337991	N/A	3.3.2	unknown	unknown	Blood	2012	Asia	South Asia	Bangladesh	unknown	unknown	Wong et al, 2015
TY524	ERR337992	N/A	3.3.2	unknown	unknown	Blood	2012	Asia	South Asia	Bangladesh	unknown	unknown	Wong et al, 2015
TY529	ERR337993	N/A	4.3.1.3	unknown	unknown	Blood	2012	Asia	South Asia	Bangladesh	unknown	unknown	Wong et al, 2015
TY585	ERR337995	N/A	4.3.1.1	unknown	unknown	Blood	2012	Asia	South Asia	Bangladesh	unknown	unknown	Wong et al, 2015
TY589	ERR337996	N/A	4.3.1.2	unknown	unknown	Blood	2012	Asia	South Asia	Bangladesh	unknown	unknown	Wong et al, 2015
TY647	ERR337997	N/A	4.3.1.3	unknown	unknown	Blood	2012	Asia	South Asia	Bangladesh	unknown	unknown	Wong et al, 2015
231697	ERR337998	N/A	2.4.1	unknown	unknown	Blood	2007	Africa	South Africa	South Africa	unknown	unknown	Wong et al, 2015

155749	ERR337999	N/A	2.5	unknown	unknown	Blood	2006	Africa	South Africa	South Africa	unknown	unknown	Wong et al, 2015
175806	ERR338001	N/A	2.4	unknown	unknown	Blood	2006	Africa	South Africa	South Africa	unknown	unknown	Wong et al, 2015
1647901	ERR338002	N/A	4.3.1.1.EA1	unknown	unknown	Blood	2005	Africa	South Africa	South Africa	unknown	unknown	Wong et al, 2015
1025041	ERR338003	N/A	2.4.1	unknown	unknown	Blood	2004	Africa	South Africa	South Africa	unknown	unknown	Wong et al, 2015
1025617	ERR338004	N/A	4.1.1	unknown	unknown	Blood	2004	Africa	South Africa	South Africa	unknown	unknown	Wong et al, 2015
1647624	ERR338005	N/A	2.4	unknown	unknown	Blood	2005	Africa	South Africa	South Africa	unknown	unknown	Wong et al, 2015
237091	ERR338006	N/A	2.4.1	unknown	unknown	Blood	2007	Africa	South Africa	South Africa	unknown	unknown	Wong et al, 2015
226195	ERR338007	N/A	2.4.1	unknown	unknown	Blood	2007	Africa	South Africa	South Africa	unknown	unknown	Wong et al, 2015
206926	ERR338008	N/A	1.1.2	unknown	unknown	Blood	2007	Africa	South Africa	South Africa	unknown	unknown	Wong et al, 2015
225555	ERR338009	N/A	2.4	unknown	unknown	Blood	2007	Africa	South Africa	South Africa	unknown	unknown	Wong et al, 2015
241451	ERR338010	N/A	2.4.1	unknown	unknown	Blood	2008	Africa	South Africa	South Africa	unknown	unknown	Wong et al, 2015
238675	ERR338011	N/A	2.4	unknown	unknown	Blood	2008	Africa	South Africa	South Africa	unknown	unknown	Wong et al, 2015
241454	ERR338012	N/A	2.4.1	unknown	unknown	Blood	2008	Africa	South Africa	South Africa	unknown	unknown	Wong et al, 2015
257401	ERR338013	N/A	2.4	unknown	unknown	Blood	2008	Africa	South Africa	South Africa	unknown	unknown	Wong et al, 2015
141461	ERR338016	N/A	2.4.1	unknown	unknown	Blood	2006	Africa	South Africa	South Africa	unknown	unknown	Wong et al, 2015
1650362	ERR338019	N/A	2.4	unknown	unknown	Blood	2005	Africa	South Africa	South Africa	unknown	unknown	Wong et al, 2015
1026656	ERR338020	N/A	2.4	unknown	unknown	Blood	2004	Africa	South Africa	South Africa	unknown	unknown	Wong et al, 2015
493790	ERR338023	N/A	4.3.1.1.EA1	unknown	unknown	Blood	2010	Africa	South Africa	South Africa	unknown	unknown	Wong et al, 2015
520772	ERR338024	N/A	2.4.1	unknown	unknown	Breast pus	2010	Africa	South Africa	South Africa	unknown	unknown	Wong et al, 2015
516981	ERR338025	N/A	2.4	unknown	unknown	Blood	2010	Africa	South Africa	South Africa	unknown	unknown	Wong et al, 2015
474899	ERR338026	N/A	4.3.1.3.Bdq	unknown	unknown	Blood	2010	Africa	South Africa	South Africa	unknown	unknown	Wong et al, 2015
427489	ERR338027	N/A	3.3.1	unknown	unknown	Blood & bone marrow	2010	Africa	South Africa	South Africa	unknown	unknown	Wong et al, 2015
412921	ERR338028	N/A	2.5	unknown	unknown	Blood	2009	Africa	South Africa	South Africa	unknown	unknown	Wong et al, 2015
410378	ERR338029	N/A	2.4	unknown	unknown	Blood	2009	Africa	South Africa	South Africa	unknown	unknown	Wong et al, 2015
410323	ERR338030	N/A	3.3.1	unknown	unknown	Blood	2009	Africa	South Africa	South Africa	unknown	unknown	Wong et al, 2015
542825	ERR338031	N/A	3.3.1	unknown	unknown	Blood	2011	Africa	South Africa	South Africa	unknown	unknown	Wong et al, 2015
540614	ERR338032	N/A	2.5	unknown	unknown	Blood	2011	Africa	South Africa	South Africa	unknown	unknown	Wong et al, 2015
583236	ERR338034	N/A	4.3.1.1.EA1	unknown	unknown	Blood	2011	Africa	South Africa	South Africa	unknown	unknown	Wong et al, 2015
627334	ERR338035	N/A	1.1.2	unknown	unknown	Blood	2012	Africa	South Africa	South Africa	unknown	unknown	Wong et al, 2015
648212	ERR338036	N/A	4.3.1.1.EA1	unknown	unknown	Blood	2012	Africa	South Africa	South Africa	unknown	unknown	Wong et al, 2015
639624	ERR338037	N/A	3.3.1	unknown	unknown	Blood	2012	Africa	South Africa	South Africa	unknown	unknown	Wong et al, 2015
363192	ERR338039	N/A	3.3.1	unknown	unknown	Blood	2009	Africa	South Africa	South Africa	unknown	unknown	Wong et al, 2015
316083	ERR338040	N/A	2.5	unknown	unknown	Blood	2009	Africa	South Africa	South Africa	unknown	unknown	Wong et al, 2015
671445	ERR338041	N/A	2.4.1	unknown	unknown	Stool	2012	Africa	South Africa	South Africa	unknown	unknown	Wong et al, 2015
325681	ERR338042	N/A	4.1.1	unknown	unknown	CSF & Blood	2009	Africa	South Africa	South Africa	unknown	unknown	Wong et al, 2015
338900	ERR338043	N/A	2.4.1	unknown	unknown	Blood	2009	Africa	South Africa	South Africa	unknown	unknown	Wong et al, 2015
400746	ERR338044	N/A	2.4	unknown	unknown	Blood	2009	Africa	South Africa	South Africa	unknown	unknown	Wong et al, 2015
379905	ERR338046	N/A	2.4.1	unknown	unknown	Blood	2009	Africa	South Africa	South Africa	unknown	unknown	Wong et al, 2015
634660	ERR338047	N/A	2.4	unknown	unknown	Blood	2012	Africa	South Africa	South Africa	unknown	unknown	Wong et al, 2015
671076	ERR338049	N/A	3.1	unknown	unknown	Blood	2012	Africa	South Africa	South Africa	unknown	unknown	Wong et al, 2015
A102	ERR338050	N/A	2.1.9	unknown	unknown	Blood	2005	Asia	Southeast Asia	Indonesia	unknown	unknown	Wong et al, 2015
A105	ERR338051	N/A	3.1.2	unknown	unknown	Blood	2012	Asia	Southeast Asia	Indonesia	unknown	unknown	Wong et al, 2015
A109	ERR338052	N/A	2.1.3	unknown	unknown	Blood	2004	Asia	Southeast Asia	Indonesia	unknown	unknown	Wong et al, 2015
A122	ERR338058	N/A	3.1.2	unknown	unknown	Blood	2005	Asia	Southeast Asia	Indonesia	unknown	unknown	Wong et al, 2015
A125	ERR338060	N/A	3.1.2	unknown	unknown	Blood	2005	Asia	Southeast Asia	Indonesia	unknown	unknown	Wong et al, 2015

A127	ERR338061	N/A	2.1.8	unknown	unknown	Blood	2011	Asia	Southeast Asia	Indonesia	unknown	unknown	Wong et al, 2015
A128	ERR338062	N/A	3.1.2	unknown	unknown	Blood	2008	Asia	Southeast Asia	Indonesia	unknown	unknown	Wong et al, 2015
A131	ERR338064	N/A	3	unknown	unknown	Blood	2004	Asia	Southeast Asia	Indonesia	unknown	unknown	Wong et al, 2015
A132	ERR338065	N/A	3.1.2	unknown	unknown	Blood	2011	Asia	Southeast Asia	Indonesia	unknown	unknown	Wong et al, 2015
A135	ERR338067	N/A	3.1.2	unknown	unknown	Blood	2004	Asia	Southeast Asia	Indonesia	unknown	unknown	Wong et al, 2015
A136	ERR338068	N/A	3.1.2	unknown	unknown	Blood	2010	Asia	Southeast Asia	Indonesia	unknown	unknown	Wong et al, 2015
A137	ERR338069	N/A	4.1	unknown	unknown	Blood	2008	Asia	Southeast Asia	Indonesia	unknown	unknown	Wong et al, 2015
Gen-0001	ERR338070	N/A	3.5.4	unknown	unknown	Not provided	2012	Australia & Oceania	Oceania	Samoa	unknown	unknown	Wong et al, 2015
Gen-0002	ERR338071	N/A	3.5.4	unknown	unknown	Not provided	2012	Australia & Oceania	Oceania	Samoa	unknown	unknown	Wong et al, 2015
Gen-0003	ERR338072	N/A	3.5.4	unknown	unknown	Not provided	2012	Australia & Oceania	Oceania	Samoa	unknown	unknown	Wong et al, 2015
Gen-0004	ERR338073	N/A	3.5.4	unknown	unknown	Not provided	2012	Australia & Oceania	Oceania	Samoa	unknown	unknown	Wong et al, 2015
Gen-0005	ERR338074	N/A	3.5.4	unknown	unknown	Not provided	2012	Australia & Oceania	Oceania	Samoa	unknown	unknown	Wong et al, 2015
Gen-0006	ERR338075	N/A	3.5.4	unknown	unknown	Not provided	2012	Australia & Oceania	Oceania	Samoa	unknown	unknown	Wong et al, 2015
Gen-0007	ERR338076	N/A	3.5.4	unknown	unknown	Not provided	2012	Australia & Oceania	Oceania	Samoa	unknown	unknown	Wong et al, 2015
Gen-0008	ERR338077	N/A	3.5.4	unknown	unknown	Not provided	2012	Australia & Oceania	Oceania	Samoa	unknown	unknown	Wong et al, 2015
Gen-0009	ERR338078	N/A	3.5.4	unknown	unknown	Not provided	2012	Australia & Oceania	Oceania	Samoa	unknown	unknown	Wong et al, 2015
Gen-0010	ERR338079	N/A	3.5.4	unknown	unknown	Not provided	2012	Australia & Oceania	Oceania	Samoa	unknown	unknown	Wong et al, 2015
Gen-0011	ERR338080	N/A	3.5.4	unknown	unknown	Not provided	2012	Australia & Oceania	Oceania	Samoa	unknown	unknown	Wong et al, 2015
Gen-0012	ERR338081	N/A	3.5.4	unknown	unknown	Not provided	2012	Australia & Oceania	Oceania	Samoa	unknown	unknown	Wong et al, 2015
Gen-0013	ERR338082	N/A	3.5.4	unknown	unknown	Not provided	2012	Australia & Oceania	Oceania	Samoa	unknown	unknown	Wong et al, 2015
Gen-0014	ERR338083	N/A	3.5.4	unknown	unknown	Not provided	2012	Australia & Oceania	Oceania	Samoa	unknown	unknown	Wong et al, 2015
Gen-0015	ERR338084	N/A	3.5.4	unknown	unknown	Not provided	2012	Australia & Oceania	Oceania	Samoa	unknown	unknown	Wong et al, 2015
Gen-0016	ERR338085	N/A	3.5.4	unknown	unknown	Not provided	2012	Australia & Oceania	Oceania	Samoa	unknown	unknown	Wong et al, 2015
Gen-0017	ERR338086	N/A	3.5.4	unknown	unknown	Not provided	2012	Australia & Oceania	Oceania	Samoa	unknown	unknown	Wong et al, 2015
Gen-0018	ERR338087	N/A	3.5.4	unknown	unknown	Not provided	2012	Australia & Oceania	Oceania	Samoa	unknown	unknown	Wong et al, 2015
Gen-0019	ERR338088	N/A	3.5.4	unknown	unknown	Not provided	2012	Australia & Oceania	Oceania	Samoa	unknown	unknown	Wong et al, 2015
Gen-0020	ERR338089	N/A	3.5.4	unknown	unknown	Not provided	2012	Australia & Oceania	Oceania	Samoa	unknown	unknown	Wong et al, 2015
Gen-0021	ERR338090	N/A	3.5.4	unknown	unknown	Not provided	2012	Australia & Oceania	Oceania	Samoa	unknown	unknown	Wong et al, 2015
Gen-0022	ERR338091	N/A	3.5.4	unknown	unknown	Not provided	2012	Australia & Oceania	Oceania	Samoa	unknown	unknown	Wong et al, 2015
Gen-0023	ERR338092	N/A	3.5.4	unknown	unknown	Not provided	2012	Australia & Oceania	Oceania	Samoa	unknown	unknown	Wong et al, 2015
Gen-0024	ERR338093	N/A	3.5.4	unknown	unknown	Not provided	2012	Australia & Oceania	Oceania	Samoa	unknown	unknown	Wong et al, 2015

Gen-0025	ERR338094	N/A	3.5.4	unknown	unknown	Not provided	2012	Australia & Oceania	Oceania	Samoa	unknown	unknown	Wong et al, 2015
Gen-0026	ERR338095	N/A	3.5.4	unknown	unknown	Not provided	2012	Australia & Oceania	Oceania	Samoa	unknown	unknown	Wong et al, 2015
Gen-0027	ERR338096	N/A	3.5.4	unknown	unknown	Not provided	2012	Australia & Oceania	Oceania	Samoa	unknown	unknown	Wong et al, 2015
Gen-0028	ERR338097	N/A	3.5.4	unknown	unknown	Not provided	2012	Australia & Oceania	Oceania	Samoa	unknown	unknown	Wong et al, 2015
Gen-0029	ERR338098	N/A	3.5.4	unknown	unknown	Not provided	2012	Australia & Oceania	Oceania	Samoa	unknown	unknown	Wong et al, 2015
Gen-0030	ERR338099	N/A	4.1	unknown	unknown	Not provided	2012	Australia & Oceania	Oceania	Samoa	unknown	unknown	Wong et al, 2015
Gen-0031	ERR338100	N/A	3.5.4	unknown	unknown	Not provided	2012	Australia & Oceania	Oceania	Samoa	unknown	unknown	Wong et al, 2015
Gen-0032	ERR338101	N/A	3.5.4	unknown	unknown	Not provided	2012	Australia & Oceania	Oceania	Samoa	unknown	unknown	Wong et al, 2015
Gen-0033	ERR338102	N/A	3.5.4	unknown	unknown	Not provided	2012	Australia & Oceania	Oceania	Samoa	unknown	unknown	Wong et al, 2015
Gen-0034	ERR338103	N/A	3.5.4	unknown	unknown	Not provided	2012	Australia & Oceania	Oceania	Samoa	unknown	unknown	Wong et al, 2015
Gen-0035	ERR338104	N/A	3.5.4	unknown	unknown	Not provided	2012	Australia & Oceania	Oceania	Samoa	unknown	unknown	Wong et al, 2015
Gen-0036	ERR338105	N/A	3.5.4	unknown	unknown	Not provided	2012	Australia & Oceania	Oceania	Samoa	unknown	unknown	Wong et al, 2015
Gen-0037	ERR338106	N/A	3.5.4	unknown	unknown	Not provided	2012	Australia & Oceania	Oceania	Samoa	unknown	unknown	Wong et al, 2015
Gen-0038	ERR338107	N/A	3.5.4	unknown	unknown	Not provided	2012	Australia & Oceania	Oceania	Samoa	unknown	unknown	Wong et al, 2015
Gen-0039	ERR338108	N/A	3.5.4	unknown	unknown	Not provided	2012	Australia & Oceania	Oceania	Samoa	unknown	unknown	Wong et al, 2015
Gen-0040	ERR338109	N/A	3.5.4	unknown	unknown	Not provided	2012	Australia & Oceania	Oceania	Samoa	unknown	unknown	Wong et al, 2015
Gen-0041	ERR338110	N/A	3.5.4	unknown	unknown	Not provided	2012	Australia & Oceania	Oceania	Samoa	unknown	unknown	Wong et al, 2015
Gen-0042	ERR338111	N/A	3.5.4	unknown	unknown	Not provided	2012	Australia & Oceania	Oceania	Samoa	unknown	unknown	Wong et al, 2015
Gen-0043	ERR338112	N/A	3.5.4	unknown	unknown	Not provided	2012	Australia & Oceania	Oceania	Samoa	unknown	unknown	Wong et al, 2015
Gen-0044	ERR338113	N/A	3.5.4	unknown	unknown	Not provided	2012	Australia & Oceania	Oceania	Samoa	unknown	unknown	Wong et al, 2015
Gen-0045	ERR338114	N/A	3.5.4	unknown	unknown	Not provided	2012	Australia & Oceania	Oceania	Samoa	unknown	unknown	Wong et al, 2015
Gen-0046	ERR338115	N/A	3.5.4	unknown	unknown	Not provided	2012	Australia & Oceania	Oceania	Samoa	unknown	unknown	Wong et al, 2015
Gen-0047	ERR338116	N/A	3.5.4	unknown	unknown	Not provided	2012	Australia & Oceania	Oceania	Samoa	unknown	unknown	Wong et al, 2015
Gen-0048	ERR338117	N/A	3.5.4	unknown	unknown	Not provided	2012	Australia & Oceania	Oceania	Samoa	unknown	unknown	Wong et al, 2015
Gen-0049	ERR338118	N/A	3.5.4	unknown	unknown	Not provided	2012	Australia & Oceania	Oceania	Samoa	unknown	unknown	Wong et al, 2015
Gen-0050	ERR338119	N/A	3.5.4	unknown	unknown	Not provided	2012	Australia & Oceania	Oceania	Samoa	unknown	unknown	Wong et al, 2015
Gen-0051	ERR338120	N/A	3.5.4	unknown	unknown	Not provided	2012	Australia & Oceania	Oceania	Samoa	unknown	unknown	Wong et al, 2015
Gen-0053	ERR338122	N/A	3.5.4	unknown	unknown	Not provided	2012	Australia & Oceania	Oceania	Samoa	unknown	unknown	Wong et al, 2015

Gen-0054	ERR338123	N/A	3.5.4	unknown	unknown	Not provided	2012	Australia & Oceania	Oceania	Samoa	unknown	unknown	Wong et al, 2015
Gen-0055	ERR338124	N/A	3.5.4	unknown	unknown	Not provided	2012	Australia & Oceania	Oceania	Samoa	unknown	unknown	Wong et al, 2015
Gen-0056	ERR338125	N/A	3.5.4	unknown	unknown	Not provided	2012	Australia & Oceania	Oceania	Samoa	unknown	unknown	Wong et al, 2015
Gen-0058	ERR338127	N/A	3.5.4	unknown	unknown	Not provided	2012	Australia & Oceania	Oceania	Samoa	unknown	unknown	Wong et al, 2015
Gen-0059	ERR338128	N/A	4.1	unknown	unknown	Not provided	2012	Australia & Oceania	Oceania	Samoa	unknown	unknown	Wong et al, 2015
Gen-0060	ERR338129	N/A	4.1	unknown	unknown	Not provided	2012	Australia & Oceania	Oceania	Samoa	unknown	unknown	Wong et al, 2015
2010K-0515	ERR338131	N/A	4.3.1.1.EA1	unknown	unknown	Blood	2007	Africa	East Africa	Kenya	unknown	unknown	Wong et al, 2015
2010K-0552	ERR338132	N/A	4.3.1.1.EA1	unknown	unknown	Blood	2008	Africa	East Africa	Kenya	unknown	unknown	Wong et al, 2015
2010K-0527	ERR338133	N/A	4.3.1.1.EA1	unknown	unknown	Blood	2007	Africa	East Africa	Kenya	unknown	unknown	Wong et al, 2015
2010K-0507	ERR338134	N/A	4.3.1.2.EA2	unknown	unknown	Blood	2007	Africa	East Africa	Kenya	unknown	unknown	Wong et al, 2015
2010K-0519	ERR338135	N/A	4.3.1.2.EA2	unknown	unknown	Blood	2007	Africa	East Africa	Kenya	unknown	unknown	Wong et al, 2015
2010K-0533	ERR338136	N/A	4.3.1.1.EA1	unknown	unknown	Blood	2008	Africa	East Africa	Kenya	unknown	unknown	Wong et al, 2015
2010K-0512	ERR338137	N/A	4.3.1.1.EA1	unknown	unknown	Blood	2007	Africa	East Africa	Kenya	unknown	unknown	Wong et al, 2015
2010K-0530	ERR338138	N/A	4.3.1.2.EA2	unknown	unknown	Blood	2007	Africa	East Africa	Kenya	unknown	unknown	Wong et al, 2015
2010K-0553	ERR338139	N/A	4.3.1.1.EA1	unknown	unknown	Blood	2008	Africa	East Africa	Kenya	unknown	unknown	Wong et al, 2015
2010K-0550	ERR338140	N/A	4.3.1.1.EA1	unknown	unknown	Blood	2009	Africa	East Africa	Kenya	unknown	unknown	Wong et al, 2015
2010K-0511	ERR338141	N/A	4.3.1.1.EA1	unknown	unknown	Blood	2007	Africa	East Africa	Kenya	unknown	unknown	Wong et al, 2015
2010K-0539	ERR338143	N/A	4.3.1.1.EA1	unknown	unknown	Blood	2008	Africa	East Africa	Kenya	unknown	unknown	Wong et al, 2015
2010K-0522	ERR338145	N/A	4.3.1.1.EA1	unknown	unknown	Blood	2007	Africa	East Africa	Kenya	unknown	unknown	Wong et al, 2015
2010K-0517	ERR338146	N/A	4.3.1.2.EA2	unknown	unknown	Blood	2007	Africa	East Africa	Kenya	unknown	unknown	Wong et al, 2015
2010K-0548	ERR338147	N/A	4.3.1.1.EA1	unknown	unknown	Blood	2008	Africa	East Africa	Kenya	unknown	unknown	Wong et al, 2015
2010K-0509	ERR338148	N/A	4.3.1.1.EA1	unknown	unknown	Blood	2007	Africa	East Africa	Kenya	unknown	unknown	Wong et al, 2015
2010K-0500	ERR338149	N/A	4.3.1.2.EA2	unknown	unknown	Stool	2007	Africa	East Africa	Kenya	unknown	unknown	Wong et al, 2015
2010K-0506	ERR338150	N/A	4.3.1.2.EA2	unknown	unknown	Blood	2007	Africa	East Africa	Kenya	unknown	unknown	Wong et al, 2015
2010K-0554	ERR338151	N/A	4.3.1.1.EA1	unknown	unknown	Blood	2008	Africa	East Africa	Kenya	unknown	unknown	Wong et al, 2015
2010K-0508	ERR338152	N/A	4.3.1.1.EA1	unknown	unknown	Blood	2007	Africa	East Africa	Kenya	unknown	unknown	Wong et al, 2015
2010K-0626	ERR338153	N/A	3.3.1	unknown	unknown	Blood	2009	Africa	East Africa	Kenya	unknown	unknown	Wong et al, 2015
2010K-0524	ERR338157	N/A	4.3.1.1.EA1	unknown	unknown	Blood	2007	Africa	East Africa	Kenya	unknown	unknown	Wong et al, 2015
2010K-0523	ERR338158	N/A	4.3.1.2.EA2	unknown	unknown	Blood	2007	Africa	East Africa	Kenya	unknown	unknown	Wong et al, 2015
2010K-0544	ERR338159	N/A	4.3.1.1.EA1	unknown	unknown	Blood	2009	Africa	East Africa	Kenya	unknown	unknown	Wong et al, 2015
2010K-0545	ERR338160	N/A	4.3.1.1.EA1	unknown	unknown	Blood	2008	Africa	East Africa	Kenya	unknown	unknown	Wong et al, 2015
2010K-0538	ERR338161	N/A	4.3.1.2.EA2	unknown	unknown	Blood	2008	Africa	East Africa	Kenya	unknown	unknown	Wong et al, 2015
2010K-0535	ERR338162	N/A	3.3.1	unknown	unknown	Blood	2009	Africa	East Africa	Kenya	unknown	unknown	Wong et al, 2015
2010K-0534	ERR338163	N/A	4.3.1.1.EA1	unknown	unknown	Blood	2008	Africa	East Africa	Kenya	unknown	unknown	Wong et al, 2015
2010K-0514	ERR338164	N/A	4.3.1.1.EA1	unknown	unknown	Blood	2007	Africa	East Africa	Kenya	unknown	unknown	Wong et al, 2015
2007-001525	ERR340757	N/A	4.3.1.1	unknown	unknown	Blood	2007	Asia	Southeast Asia	Cambodia	unknown	unknown	Wong et al, 2015
2007-001619	ERR340758	N/A	4.3.1.1	unknown	unknown	Blood	2007	Asia	Southeast Asia	Cambodia	unknown	unknown	Wong et al, 2015
2001-102645	ERR340759	N/A	4.3.1.1	unknown	unknown	Blood	2007	Asia	Southeast Asia	Cambodia	unknown	unknown	Wong et al, 2015
2007-003270	ERR340760	N/A	4.3.1.1	unknown	unknown	Blood	2007	Asia	Southeast Asia	Cambodia	unknown	unknown	Wong et al, 2015
2007-006110	ERR340761	N/A	4.3.1.1	unknown	unknown	Blood	2007	Asia	Southeast Asia	Cambodia	unknown	unknown	Wong et al, 2015
2007-005516	ERR340763	N/A	4.3.1.1	unknown	unknown	Blood	2007	Asia	Southeast Asia	Cambodia	unknown	unknown	Wong et al, 2015

2007-007073	ERR340765	N/A	3.2.1	unknown	unknown	Blood	2007	Asia	Southeast Asia	Cambodia	unknown	unknown	Wong et al, 2015
2007-007506	ERR340766	N/A	4.3.1.1	unknown	unknown	Blood	2007	Asia	Southeast Asia	Cambodia	unknown	unknown	Wong et al, 2015
2008-006685	ERR340767	N/A	4.3.1.1	unknown	unknown	Blood	2008	Asia	Southeast Asia	Cambodia	unknown	unknown	Wong et al, 2015
2007-011815	ERR340768	N/A	4.3.1.1	unknown	unknown	Blood	2007	Asia	Southeast Asia	Cambodia	unknown	unknown	Wong et al, 2015
2001-100239	ERR340769	N/A	3.2.1	unknown	unknown	Blood	2007	Asia	Southeast Asia	Cambodia	unknown	unknown	Wong et al, 2015
2007-015823	ERR340770	N/A	4.3.1.1	unknown	unknown	Blood	2007	Asia	Southeast Asia	Cambodia	unknown	unknown	Wong et al, 2015
2007-023117	ERR340771	N/A	4.3.1.1	unknown	unknown	Blood	2007	Asia	Southeast Asia	Cambodia	unknown	unknown	Wong et al, 2015
2007-024445	ERR340772	N/A	4.3.1.1	unknown	unknown	Blood	2007	Asia	Southeast Asia	Cambodia	unknown	unknown	Wong et al, 2015
2007-023116	ERR340773	N/A	4.3.1.1	unknown	unknown	Blood	2007	Asia	Southeast Asia	Cambodia	unknown	unknown	Wong et al, 2015
2007-024758	ERR340774	N/A	4.3.1.1	unknown	unknown	Blood	2007	Asia	Southeast Asia	Cambodia	unknown	unknown	Wong et al, 2015
2007-007363	ERR340775	N/A	4.3.1.1	unknown	unknown	Blood	2007	Asia	Southeast Asia	Cambodia	unknown	unknown	Wong et al, 2015
LNT 08	ERR340777	N/A	3.4	unknown	unknown	Blood	2001	Asia	Southeast Asia	Laos	unknown	unknown	Wong et al, 2015
LNT1108	ERR340778	N/A	3.4	unknown	unknown	Blood	2001	Asia	Southeast Asia	Laos	unknown	unknown	Wong et al, 2015
LNT1148	ERR340779	N/A	3.4	unknown	unknown	Blood	2001	Asia	Southeast Asia	Laos	unknown	unknown	Wong et al, 2015
LNT22	ERR340780	N/A	3.4	unknown	unknown	Blood	2001	Asia	Southeast Asia	Laos	unknown	unknown	Wong et al, 2015
LNT26	ERR340781	N/A	3.4	unknown	unknown	Blood	2001	Asia	Southeast Asia	Laos	unknown	unknown	Wong et al, 2015
LNT358	ERR340782	N/A	3.4	unknown	unknown	Blood	2000	Asia	Southeast Asia	Laos	unknown	unknown	Wong et al, 2015
UI1059	ERR340783	N/A	4.3.1.1	unknown	unknown	Blood	2001	Asia	Southeast Asia	Laos	unknown	unknown	Wong et al, 2015
UI1151	ERR340784	N/A	4.3.1.1	unknown	unknown	Blood	2001	Asia	Southeast Asia	Laos	unknown	unknown	Wong et al, 2015
UI1203	ERR340785	N/A	3.2.1	unknown	unknown	Blood	2001	Asia	Southeast Asia	Laos	unknown	unknown	Wong et al, 2015
UI1236	ERR340786	N/A	3.2.1	unknown	unknown	Blood	2001	Asia	Southeast Asia	Laos	unknown	unknown	Wong et al, 2015
UI1954-12/3	ERR340787	N/A	4.3.1.1	unknown	unknown	Blood	2002	Asia	Southeast Asia	Laos	unknown	unknown	Wong et al, 2015
UI1954-2/3	ERR340788	N/A	3.2.1	unknown	unknown	Blood	2002	Asia	Southeast Asia	Laos	unknown	unknown	Wong et al, 2015
UI1965	ERR340789	N/A	4.3.1.1	unknown	unknown	Blood	2002	Asia	Southeast Asia	Laos	unknown	unknown	Wong et al, 2015
UI1998	ERR340790	N/A	4.3.1.1	unknown	unknown	Blood	2002	Asia	Southeast Asia	Laos	unknown	unknown	Wong et al, 2015
UI2000	ERR340791	N/A	4.3.1.1	unknown	unknown	Blood	2002	Asia	Southeast Asia	Laos	unknown	unknown	Wong et al, 2015
UI2001	ERR340792	N/A	2.2.3,2.2.2	unknown	unknown	Blood	2002	Asia	Southeast Asia	Laos	unknown	unknown	Wong et al, 2015
UI2006	ERR340793	N/A	3.2.1	unknown	unknown	Blood	2002	Asia	Southeast Asia	Laos	unknown	unknown	Wong et al, 2015
UI2063	ERR340794	N/A	4.3.1.1	unknown	unknown	Blood	2002	Asia	Southeast Asia	Laos	unknown	unknown	Wong et al, 2015
UI2120	ERR340795	N/A	4.3.1.1	unknown	unknown	Blood	2002	Asia	Southeast Asia	Laos	unknown	unknown	Wong et al, 2015
UI2144	ERR340797	N/A	4.3.1.1	unknown	unknown	Blood	2002	Asia	Southeast Asia	Laos	unknown	unknown	Wong et al, 2015
UI2152	ERR340798	N/A	4.1	unknown	unknown	Blood	2002	Asia	Southeast Asia	Laos	unknown	unknown	Wong et al, 2015
UI2155	ERR340799	N/A	4.3.1.1	unknown	unknown	Blood	2002	Asia	Southeast Asia	Laos	unknown	unknown	Wong et al, 2015
UI2220	ERR340800	N/A	4.3.1.1	unknown	unknown	Blood	2002	Asia	Southeast Asia	Laos	unknown	unknown	Wong et al, 2015
UI2351	ERR340801	N/A	3.2.1	unknown	unknown	Blood	2002	Asia	Southeast Asia	Laos	unknown	unknown	Wong et al, 2015
UI247	ERR340802	N/A	4.3.1.1	unknown	unknown	Blood	2000	Asia	Southeast Asia	Laos	unknown	unknown	Wong et al, 2015
UI2591	ERR340803	N/A	3.4	unknown	unknown	Blood	2002	Asia	Southeast Asia	Laos	unknown	unknown	Wong et al, 2015
UI2658	ERR340804	N/A	3.4	unknown	unknown	Blood	2002	Asia	Southeast Asia	Laos	unknown	unknown	Wong et al, 2015
UI2679	ERR340805	N/A	3.2.1	unknown	unknown	Blood	2002	Asia	Southeast Asia	Laos	unknown	unknown	Wong et al, 2015
UI2824	ERR340806	N/A	4.3.1.1	unknown	unknown	Blood	2002	Asia	Southeast Asia	Laos	unknown	unknown	Wong et al, 2015
UI286	ERR340807	N/A	4.3.1.1	unknown	unknown	Blood	2000	Asia	Southeast Asia	Laos	unknown	unknown	Wong et al, 2015
UI3146	ERR340808	N/A	2.3.4	unknown	unknown	Blood	2002	Asia	Southeast Asia	Laos	unknown	unknown	Wong et al, 2015
UI315	ERR340809	N/A	4.3.1.1	unknown	unknown	Blood	2000	Asia	Southeast Asia	Laos	unknown	unknown	Wong et al, 2015
UI3151	ERR340810	N/A	2.3.4	unknown	unknown	Blood	2002	Asia	Southeast Asia	Laos	unknown	unknown	Wong et al, 2015
UI3260	ERR340811	N/A	3.2.1	unknown	unknown	Blood	2003	Asia	Southeast Asia	Laos	unknown	unknown	Wong et al, 2015

UI3275	ERR340812	N/A	4.3.1.1	unknown	unknown	Blood	2003	Asia	Southeast Asia	Laos	unknown	unknown	Wong et al, 2015
UI3313	ERR340813	N/A	3.4	unknown	unknown	Blood	2003	Asia	Southeast Asia	Laos	unknown	unknown	Wong et al, 2015
UI3396	ERR340814	N/A	2.2.3,2.2.2	unknown	unknown	Blood	2003	Asia	Southeast Asia	Laos	unknown	unknown	Wong et al, 2015
UI473	ERR340815	N/A	4.3.1.1	unknown	unknown	Blood	2000	Asia	Southeast Asia	Laos	unknown	unknown	Wong et al, 2015
UI54	ERR340816	N/A	4.1	unknown	unknown	Blood	2000	Asia	Southeast Asia	Laos	unknown	unknown	Wong et al, 2015
ERL021174	ERR343249	N/A	3.5.4	unknown	unknown	Blood	2002	Australia & Oceania	Oceania	Samoa	unknown	unknown	Wong et al, 2015
ERL022463	ERR343250	N/A	2.1.4	unknown	unknown	Stool	2002	Asia	Western Asia	Western Asia	unknown	unknown	Wong et al, 2015
ERL022464	ERR343251	N/A	2.1.2	unknown	unknown	Blood	2002	Asia	Western Asia	Western Asia	unknown	unknown	Wong et al, 2015
ERL024120	ERR343252	N/A	2.1.2	unknown	unknown	Stool	2002	Asia	Southeast Asia	Indonesia	unknown	unknown	Wong et al, 2015
ERL024182	ERR343253	N/A	2.5	unknown	unknown	Blood	2002	Asia	South Asia	India	unknown	unknown	Wong et al, 2015
ERL02425	ERR343254	N/A	2.5	unknown	unknown	Stool	2002	Asia	South Asia	India	unknown	unknown	Wong et al, 2015
ERL024919	ERR343255	N/A	3.5.4	unknown	unknown	Blood	2002	Australia & Oceania	Oceania	Samoa	unknown	unknown	Wong et al, 2015
ERL02732	ERR343256	N/A	3.1.2	unknown	unknown	Blood	2002	Asia	Southeast Asia	Indonesia	unknown	unknown	Wong et al, 2015
ERL032200	ERR343257	N/A	4.2	unknown	unknown	Blood	2003	Australia & Oceania	Oceania	Tonga	unknown	unknown	Wong et al, 2015
ERL032330	ERR343258	N/A	4.3.1.2	unknown	unknown	Blood	2003	Asia	South Asia	India	unknown	unknown	Wong et al, 2015
ERL034151	ERR343260	N/A	2.2.1	unknown	unknown	Blood	2003	Asia	Southeast Asia	Indonesia	unknown	unknown	Wong et al, 2015
ERL04140	ERR343262	N/A	4.3.1.2	unknown	unknown	Blood	2004	Asia	South Asia	India	unknown	unknown	Wong et al, 2015
ERL041419	ERR343263	N/A	4.1	unknown	unknown	Blood	2004	Asia	South Asia	India	unknown	unknown	Wong et al, 2015
ERL041834	ERR343264	N/A	3.3	unknown	unknown	Stool	2004	Asia	South Asia	India	unknown	unknown	Wong et al, 2015
ERL041932	ERR343265	N/A	4.1	unknown	unknown	Blood	2004	Australia & Oceania	Oceania	Samoa	unknown	unknown	Wong et al, 2015
ERL042857	ERR343266	N/A	3.5.4	unknown	unknown	Blood	2004	Australia & Oceania	Oceania	Samoa	unknown	unknown	Wong et al, 2015
ERL043008	ERR343267	N/A	3.5.4	unknown	unknown	Stool	2004	Australia & Oceania	Oceania	Samoa	unknown	unknown	Wong et al, 2015
ERL052042	ERR343269	N/A	4.3.1.1	unknown	unknown	Blood	2005	Asia	South Asia	India	unknown	unknown	Wong et al, 2015
ERL061748	ERR343270	N/A	4.3.1.1	unknown	unknown	Blood	2006	Asia	Southeast Asia	South-east Asia	unknown	unknown	Wong et al, 2015
ERL062282	ERR343271	N/A	4.3.1.2	unknown	unknown	Blood	2006	Asia	South Asia	India	unknown	unknown	Wong et al, 2015
ERL063423	ERR343272	N/A	4.3.1.1	unknown	unknown	Blood	2006	Asia	South Asia	India	unknown	unknown	Wong et al, 2015
ERL063424	ERR343273	N/A	4.3.1.2	unknown	unknown	Blood	2006	Asia	South Asia	India	unknown	unknown	Wong et al, 2015
ERL064553	ERR343274	N/A	3.5.4	unknown	unknown	Blood	2006	Australia & Oceania	Oceania	Samoa	unknown	unknown	Wong et al, 2015
ERL0661	ERR343275	N/A	2.5	unknown	unknown	Blood	2006	Asia	South Asia	India	unknown	unknown	Wong et al, 2015
ERL07263	ERR343276	N/A	3.5.4	unknown	unknown	Blood	2007	Australia & Oceania	Oceania	Samoa	unknown	unknown	Wong et al, 2015
ERL07264	ERR343277	N/A	3.5.4	unknown	unknown	Blood	2007	Australia & Oceania	Oceania	Samoa	unknown	unknown	Wong et al, 2015
ERL072830	ERR343278	N/A	3.5.4	unknown	unknown	Blood	2007	Australia & Oceania	Oceania	Samoa	unknown	unknown	Wong et al, 2015
ERL072973	ERR343279	N/A	4.2.2	unknown	unknown	Blood	2007	Australia & Oceania	Oceania	Fiji	unknown	unknown	Wong et al, 2015
ERL07434	ERR343280	N/A	3.5.4	unknown	unknown	Blood	2007	Australia & Oceania	Oceania	Samoa	unknown	unknown	Wong et al, 2015
ERL082325	ERR343281	N/A	4.2.1	unknown	unknown	Blood	2008	Australia & Oceania	Oceania	Fiji	unknown	unknown	Wong et al, 2015
ERL082356	ERR343282	N/A	4.3.1.2	unknown	unknown	Stool	2008	Asia	South Asia	India	unknown	unknown	Wong et al, 2015

ERL082408	ERR343283	N/A	3.5.4	unknown	unknown	Blood	2008	Australia & Oceania	Oceania	Samoa	unknown	unknown	Wong et al, 2015
ERL082444	ERR343284	N/A	3.5.4	unknown	unknown	Blood	2008	Australia & Oceania	Oceania	Samoa	unknown	unknown	Wong et al, 2015
ERL082759	ERR343285	N/A	4.2.2	unknown	unknown	Blood	2008	Australia & Oceania	Oceania	Fiji	unknown	unknown	Wong et al, 2015
ERL084047	ERR343286	N/A	2.2.2	unknown	unknown	Stool	2008	Asia	South Asia	India	unknown	unknown	Wong et al, 2015
ERL084170	ERR343287	N/A	3.1.2	unknown	unknown	Blood	2008	Asia	East Asia	China	unknown	unknown	Wong et al, 2015
ERL08619	ERR343288	N/A	4.3.1.2	unknown	unknown	Unknown	2008	Asia	South Asia	India	unknown	unknown	Wong et al, 2015
ERL08758	ERR343289	N/A	2.1.7	unknown	unknown	Blood	2008	Asia	South Asia	India	unknown	unknown	Wong et al, 2015
ERL091092	ERR343291	N/A	3.1.2	unknown	unknown	Blood	2009	Asia	Southeast Asia	Indonesia	unknown	unknown	Wong et al, 2015
ERL091300	ERR343292	N/A	3.5.4	unknown	unknown	Blood	2009	Australia & Oceania	Oceania	Samoa	unknown	unknown	Wong et al, 2015
ERL091788	ERR343293	N/A	3.2.2	unknown	unknown	Blood	2009	Asia	Southeast Asia	South-east Asia	unknown	unknown	Wong et al, 2015
ERL091797	ERR343294	N/A	4.3.1.2	unknown	unknown	Blood	2009	Asia	South Asia	India	unknown	unknown	Wong et al, 2015
ERL09383	ERR343295	N/A	3.1	unknown	unknown	Stool	2009	Asia	South Asia	India	unknown	unknown	Wong et al, 2015
ERL094053	ERR343296	N/A	3.5.4	unknown	unknown	Blood	2009	Australia & Oceania	Oceania	Samoa	unknown	unknown	Wong et al, 2015
ERL0982	ERR343297	N/A	3.5.4	unknown	unknown	Blood	2009	Australia & Oceania	Oceania	Samoa	unknown	unknown	Wong et al, 2015
ERL0983	ERR343298	N/A	3.5.4	unknown	unknown	Blood	2009	Australia & Oceania	Oceania	Samoa	unknown	unknown	Wong et al, 2015
ERL09892	ERR343299	N/A	3.5.4	unknown	unknown	Unknown	2009	Australia & Oceania	Oceania	Samoa	unknown	unknown	Wong et al, 2015
ERL101104	ERR343300	N/A	4.3.1.2	unknown	unknown	Blood	2010	Asia	South Asia	India	unknown	unknown	Wong et al, 2015
ERL101621	ERR343301	N/A	2.0.1	unknown	unknown	Blood	2010	Asia	South Asia	India	unknown	unknown	Wong et al, 2015
ERL102156	ERR343302	N/A	4.3.1.1	unknown	unknown	Blood	2010	Asia	South Asia	India	unknown	unknown	Wong et al, 2015
ERL102275	ERR343303	N/A	4.3.1.2	unknown	unknown	Blood	2010	Asia	South Asia	India	unknown	unknown	Wong et al, 2015
ERL102461	ERR343304	N/A	3.5.4	unknown	unknown	Blood	2010	Australia & Oceania	Oceania	Samoa	unknown	unknown	Wong et al, 2015
ERL10320	ERR343305	N/A	3.5.4	unknown	unknown	Stool	2010	Australia & Oceania	Oceania	Samoa	unknown	unknown	Wong et al, 2015
ERL10338	ERR343306	N/A	4.3.1	unknown	unknown	Blood	2010	Asia	Southeast Asia	Thailand	unknown	unknown	Wong et al, 2015
ERL103534	ERR343307	N/A	4.3.1.2	unknown	unknown	Blood	2010	Asia	South Asia	India	unknown	unknown	Wong et al, 2015
ERL103914	ERR343308	N/A	2.3.2	unknown	unknown	Blood	2010	South America	South America	South America	unknown	unknown	Wong et al, 2015
ERL1048	ERR343309	N/A	4.3.1.2	unknown	unknown	Blood	2010	Asia	South Asia	India	unknown	unknown	Wong et al, 2015
ERL10492	ERR343310	N/A	3.5.4	unknown	unknown	Blood	2010	Australia & Oceania	Oceania	Samoa	unknown	unknown	Wong et al, 2015
ERL10504	ERR343311	N/A	3.5.4	unknown	unknown	Blood	2010	Australia & Oceania	Oceania	Samoa	unknown	unknown	Wong et al, 2015
ERL111572	ERR343312	N/A	3.5.4	unknown	unknown	Blood	2011	Australia & Oceania	Oceania	Samoa	unknown	unknown	Wong et al, 2015
ERL111998	ERR343313	N/A	3.5.4	unknown	unknown	Blood	2011	Australia & Oceania	Oceania	Samoa	unknown	unknown	Wong et al, 2015
ERL11299	ERR343314	N/A	2.0.1	unknown	unknown	Stool	2011	Asia	South Asia	Pakistan	unknown	unknown	Wong et al, 2015
ERL113095	ERR343315	N/A	4.3.1.2	unknown	unknown	Blood	2011	Asia	South Asia	India	unknown	unknown	Wong et al, 2015
ERL113434	ERR343316	N/A	3.5.4	unknown	unknown	Blood	2011	Australia & Oceania	Oceania	Samoa	unknown	unknown	Wong et al, 2015
ERL114000	ERR343317	N/A	2.2	unknown	unknown	Blood	2011	Asia	South Asia	Nepal	unknown	unknown	Wong et al, 2015
ERL114070	ERR343318	N/A	2.2	unknown	unknown	Blood	2011	South America	South America	South America	unknown	unknown	Wong et al, 2015
ERL114224	ERR343319	N/A	4.3.1.2	unknown	unknown	Blood	2011	Asia	South Asia	India	unknown	unknown	Wong et al, 2015

ERL11877	ERR343320	N/A	4.3.1.2	unknown	unknown	Stool	2011	Asia	South Asia	India	unknown	unknown	Wong et al, 2015
ERL11909	ERR343321	N/A	2.0.2	unknown	unknown	Stool	2011	North America	North America	Mexico	unknown	unknown	Wong et al, 2015
ERL12148	ERR343322	N/A	4.3.1.1	unknown	unknown	Stool	2012	Asia	South Asia	India	unknown	unknown	Wong et al, 2015
ERL12375	ERR343323	N/A	4.3.1.2	unknown	unknown	Blood	2012	Asia	South Asia	India	unknown	unknown	Wong et al, 2015
ERL12590	ERR343324	N/A	4.1	unknown	unknown	Stool	2012	Asia	South Asia	India	unknown	unknown	Wong et al, 2015
ERL12680	ERR343325	N/A	2.2.4	unknown	unknown	Stool	2012	Asia	South Asia	India	unknown	unknown	Wong et al, 2015
ERL12959	ERR343326	N/A	3.1.2	unknown	unknown	Blood	2012	Asia	South Asia	India	unknown	unknown	Wong et al, 2015
ERL12960	ERR343327	N/A	4.3.1.1	unknown	unknown	Unknown	2012	Asia	South Asia	India	unknown	unknown	Wong et al, 2015
H12ESR00755-001A	ERR343328	N/A	3	unknown	unknown	Blood	2012	Asia	Southeast Asia	Phillipines	unknown	unknown	Wong et al, 2015
H12ESR02737-001A	ERR343329	N/A	2.4	unknown	unknown	Blood	2012	Asia	South Asia	India	unknown	unknown	Wong et al, 2015
H12ESR04734-001A	ERR343330	N/A	3.3	unknown	unknown	Blood	2012	Asia	South Asia	India	unknown	unknown	Wong et al, 2015
dtc8	ERR349331	N/A	4.3.1.1	unknown	unknown	Blood	1994	Asia	Southeast Asia	Vietnam	unknown	unknown	Wong et al, 2015
np69	ERR349332	N/A	4.3.1.2	unknown	unknown	Blood	2011	Asia	South Asia	Nepal	unknown	unknown	Wong et al, 2015
np74	ERR349333	N/A	4.3.1.2	unknown	unknown	Blood	2011	Asia	South Asia	Nepal	unknown	unknown	Wong et al, 2015
dtc86	ERR349334	N/A	4.3.1.1	unknown	unknown	Blood	1994	Asia	Southeast Asia	Vietnam	unknown	unknown	Wong et al, 2015
np45	ERR349335	N/A	3.3.2	unknown	unknown	Blood	2011	Asia	South Asia	Nepal	unknown	unknown	Wong et al, 2015
np80	ERR349336	N/A	4.3.1.2	unknown	unknown	Blood	2011	Asia	South Asia	Nepal	unknown	unknown	Wong et al, 2015
dtc105	ERR349337	N/A	4.3.1.1	unknown	unknown	Blood	1994	Asia	Southeast Asia	Vietnam	unknown	unknown	Wong et al, 2015
dtc153	ERR349338	N/A	4.3.1.1	unknown	unknown	Blood	1995	Asia	Southeast Asia	Vietnam	unknown	unknown	Wong et al, 2015
np27	ERR349339	N/A	4.3.1.2	unknown	unknown	Blood	2011	Asia	South Asia	Nepal	unknown	unknown	Wong et al, 2015
dtc174	ERR349341	N/A	4.3.1.1	unknown	unknown	Blood	1995	Asia	Southeast Asia	Vietnam	unknown	unknown	Wong et al, 2015
BRD948	ERR349343	N/A	4.1	unknown	unknown	Not provided	1996	Europe	Eastern Europe	Russia	unknown	unknown	Wong et al, 2015
dn191	ERR349345	N/A	4.3.1.1	unknown	unknown	Blood	1996	Asia	Southeast Asia	Vietnam	unknown	unknown	Wong et al, 2015
dn14	ERR349346	N/A	4.3.1.1	unknown	unknown	Blood	1995	Asia	Southeast Asia	Vietnam	unknown	unknown	Wong et al, 2015
dtc79	ERR349347	N/A	4.3.1.1	unknown	unknown	Blood	1994	Asia	Southeast Asia	Vietnam	unknown	unknown	Wong et al, 2015
dn63	ERR349348	N/A	4.3.1.1	unknown	unknown	Blood	1995	Asia	Southeast Asia	Vietnam	unknown	unknown	Wong et al, 2015
dn121	ERR349349	N/A	4.3.1.1	unknown	unknown	Blood	1996	Asia	Southeast Asia	Vietnam	unknown	unknown	Wong et al, 2015
dn136	ERR349350	N/A	4.3.1.1	unknown	unknown	Blood	1996	Asia	Southeast Asia	Vietnam	unknown	unknown	Wong et al, 2015
dtc116	ERR349351	N/A	3.4	unknown	unknown	Blood	1995	Asia	Southeast Asia	Vietnam	unknown	unknown	Wong et al, 2015
dn86	ERR349353	N/A	4.3.1.1	unknown	unknown	Blood	1995	Asia	Southeast Asia	Vietnam	unknown	unknown	Wong et al, 2015
dn93	ERR349354	N/A	4.3.1.1	unknown	unknown	Blood	1995	Asia	Southeast Asia	Vietnam	unknown	unknown	Wong et al, 2015
dn162	ERR349356	N/A	4.3.1.1	unknown	unknown	Blood	1996	Asia	Southeast Asia	Vietnam	unknown	unknown	Wong et al, 2015
dn18	ERR349357	N/A	4.3.1.1	unknown	unknown	Blood	1995	Asia	Southeast Asia	Vietnam	unknown	unknown	Wong et al, 2015
dtc103	ERR349359	N/A	4.3.1.1	unknown	unknown	Blood	1994	Asia	Southeast Asia	Vietnam	unknown	unknown	Wong et al, 2015
dn19	ERR349360	N/A	4.3.1.1	unknown	unknown	Blood	1995	Asia	Southeast Asia	Vietnam	unknown	unknown	Wong et al, 2015
dn160	ERR349361	N/A	4.3.1.1	unknown	unknown	Blood	1996	Asia	Southeast Asia	Vietnam	unknown	unknown	Wong et al, 2015
dtc111	ERR349362	N/A	4.1	unknown	unknown	Blood	1994	Asia	Southeast Asia	Vietnam	unknown	unknown	Wong et al, 2015
dn189	ERR349363	N/A	4.3.1.1	unknown	unknown	Blood	1996	Asia	Southeast Asia	Vietnam	unknown	unknown	Wong et al, 2015
dtc93	ERR349364	N/A	4.3.1.1	unknown	unknown	Blood	1994	Asia	Southeast Asia	Vietnam	unknown	unknown	Wong et al, 2015
dn61	ERR349367	N/A	4.3.1.1	unknown	unknown	Blood	1995	Asia	Southeast Asia	Vietnam	unknown	unknown	Wong et al, 2015
ct1-7	ERR349370	N/A	3.2.1	unknown	unknown	Blood	1993	Asia	Southeast Asia	Vietnam	unknown	unknown	Wong et al, 2015
ct1-40	ERR349372	N/A	4.3.1.1	unknown	unknown	Blood	1994	Asia	Southeast Asia	Vietnam	unknown	unknown	Wong et al, 2015
dty1-73	ERR349373	N/A	4.3.1.1	unknown	unknown	Blood	1997	Asia	Southeast Asia	Vietnam	unknown	unknown	Wong et al, 2015
ct1-17	ERR349375	N/A	4.3.1.1	unknown	unknown	Blood	1993	Asia	Southeast Asia	Vietnam	unknown	unknown	Wong et al, 2015
ipt57	ERR349376	N/A	2.3.4	unknown	unknown	Blood	1997	Asia	Southeast Asia	Vietnam	unknown	unknown	Wong et al, 2015

ty3-193	ERR349378	N/A	4.3.1.1	unknown	unknown	Blood	1997	Asia	Southeast Asia	Vietnam	unknown	unknown	Wong et al, 2015
ct1-3	ERR349381	N/A	4.3.1.1	unknown	unknown	Blood	1993	Asia	Southeast Asia	Vietnam	unknown	unknown	Wong et al, 2015
ty3-214	ERR349384	N/A	4.1	unknown	unknown	Blood	1997	Asia	Southeast Asia	Vietnam	unknown	unknown	Wong et al, 2015
ct1-102	ERR349385	N/A	4.3.1.1	unknown	unknown	Blood	1994	Asia	Southeast Asia	Vietnam	unknown	unknown	Wong et al, 2015
ct1-34	ERR349387	N/A	4.3.1.1	unknown	unknown	Blood	1994	Asia	Southeast Asia	Vietnam	unknown	unknown	Wong et al, 2015
ipt41	ERR349388	N/A	4.3.1.1	unknown	unknown	Blood	1997	Asia	Southeast Asia	Vietnam	unknown	unknown	Wong et al, 2015
ty1-35	ERR349390	N/A	3.5	unknown	unknown	Blood	1993	Asia	Southeast Asia	Vietnam	unknown	unknown	Wong et al, 2015
ty2-120	ERR349392	N/A	4.3.1.1	unknown	unknown	Blood	1994	Asia	Southeast Asia	Vietnam	unknown	unknown	Wong et al, 2015
ty2-91	ERR349397	N/A	3.2.1	unknown	unknown	Blood	1994	Asia	Southeast Asia	Vietnam	unknown	unknown	Wong et al, 2015
ty2-107	ERR349398	N/A	2.3.2	unknown	unknown	Blood	1994	Asia	Southeast Asia	Vietnam	unknown	unknown	Wong et al, 2015
ty2-32	ERR349400	N/A	3.2.1	unknown	unknown	Blood	1993	Asia	Southeast Asia	Vietnam	unknown	unknown	Wong et al, 2015
ty2-93	ERR349402	N/A	4.1	unknown	unknown	Blood	1994	Asia	Southeast Asia	Vietnam	unknown	unknown	Wong et al, 2015
ty2-108	ERR349403	N/A	4.3.1.1	unknown	unknown	Blood	1994	Asia	Southeast Asia	Vietnam	unknown	unknown	Wong et al, 2015
ty1-16	ERR349405	N/A	3.1	unknown	unknown	Blood	1993	Asia	Southeast Asia	Vietnam	unknown	unknown	Wong et al, 2015
ty2-80	ERR349406	N/A	3.4	unknown	unknown	Blood	1994	Asia	Southeast Asia	Vietnam	unknown	unknown	Wong et al, 2015
ty2-98	ERR349411	N/A	3.2.1	unknown	unknown	Blood	1994	Asia	Southeast Asia	Vietnam	unknown	unknown	Wong et al, 2015
ty2-111	ERR349412	N/A	2.1.7	unknown	unknown	Blood	1994	Asia	Southeast Asia	Vietnam	unknown	unknown	Wong et al, 2015
ty2-99	ERR349415	N/A	3.2.1	unknown	unknown	Blood	1994	Asia	Southeast Asia	Vietnam	unknown	unknown	Wong et al, 2015
ty2-86	ERR349423	N/A	3.2.1	unknown	unknown	Blood	1994	Asia	Southeast Asia	Vietnam	unknown	unknown	Wong et al, 2015
ty1-34	ERR349424	N/A	2.3.3	unknown	unknown	Blood	1993	Asia	Southeast Asia	Vietnam	unknown	unknown	Wong et al, 2015
ty2-75	ERR349426	N/A	3.2.1	unknown	unknown	Blood	1994	Asia	Southeast Asia	Vietnam	unknown	unknown	Wong et al, 2015
dtc95	ERR349523	N/A	4.3.1.1	unknown	unknown	Blood	1994	Asia	Southeast Asia	Vietnam	unknown	unknown	Wong et al, 2015
dtc110	ERR349524	N/A	4.3.1.1	unknown	unknown	Blood	1994	Asia	Southeast Asia	Vietnam	unknown	unknown	Wong et al, 2015
3525/3	ERR349525	N/A	2.5.1	unknown	unknown	Blood	2011	Africa	Central Africa	DRC	unknown	unknown	Wong et al, 2015
3080/3	ERR349526	N/A	2.5.1	unknown	unknown	Blood	2010	Africa	Central Africa	DRC	unknown	unknown	Wong et al, 2015
3069/3	ERR349527	N/A	2.5.1	unknown	unknown	Blood	2010	Africa	Central Africa	DRC	unknown	unknown	Wong et al, 2015
np61	ERR349528	N/A	4.3.1.2	unknown	unknown	Blood	2011	Asia	South Asia	Nepal	unknown	unknown	Wong et al, 2015
dtc139	ERR349531	N/A	4.3.1.1	unknown	unknown	Blood	1995	Asia	Southeast Asia	Vietnam	unknown	unknown	Wong et al, 2015
dtc102	ERR349532	N/A	4.3.1.1	unknown	unknown	Blood	1994	Asia	Southeast Asia	Vietnam	unknown	unknown	Wong et al, 2015
dn163	ERR349533	N/A	4.3.1.1	unknown	unknown	Blood	1996	Asia	Southeast Asia	Vietnam	unknown	unknown	Wong et al, 2015
dn45	ERR349534	N/A	4.3.1.1	unknown	unknown	Blood	1995	Asia	Southeast Asia	Vietnam	unknown	unknown	Wong et al, 2015
dtc3	ERR349535	N/A	4.3.1.1	unknown	unknown	Blood	1994	Asia	Southeast Asia	Vietnam	unknown	unknown	Wong et al, 2015
dn15	ERR349536	N/A	4.3.1.1	unknown	unknown	Blood	1995	Asia	Southeast Asia	Vietnam	unknown	unknown	Wong et al, 2015
dn110	ERR349537	N/A	3.4	unknown	unknown	Blood	1996	Asia	Southeast Asia	Vietnam	unknown	unknown	Wong et al, 2015
dtc150	ERR349538	N/A	4.3.1.1	unknown	unknown	Blood	1995	Asia	Southeast Asia	Vietnam	unknown	unknown	Wong et al, 2015
dtc92	ERR349539	N/A	4.3.1.1	unknown	unknown	Blood	1994	Asia	Southeast Asia	Vietnam	unknown	unknown	Wong et al, 2015
dn10	ERR349541	N/A	4.3.1.1	unknown	unknown	Blood	1995	Asia	Southeast Asia	Vietnam	unknown	unknown	Wong et al, 2015
dn109	ERR349543	N/A	4.3.1.1	unknown	unknown	Blood	1996	Asia	Southeast Asia	Vietnam	unknown	unknown	Wong et al, 2015
dtc138	ERR349545	N/A	4.3.1.1	unknown	unknown	Blood	1995	Asia	Southeast Asia	Vietnam	unknown	unknown	Wong et al, 2015
dtc69	ERR349546	N/A	4.3.1.1	unknown	unknown	Blood	1994	Asia	Southeast Asia	Vietnam	unknown	unknown	Wong et al, 2015
dn12	ERR349547	N/A	4.3.1.1	unknown	unknown	Blood	1995	Asia	Southeast Asia	Vietnam	unknown	unknown	Wong et al, 2015
dn108	ERR349548	N/A	4.3.1.1	unknown	unknown	Blood	1996	Asia	Southeast Asia	Vietnam	unknown	unknown	Wong et al, 2015
dn57	ERR349549	N/A	4.3.1.1	unknown	unknown	Blood	1995	Asia	Southeast Asia	Vietnam	unknown	unknown	Wong et al, 2015
dtc71	ERR349550	N/A	4.3.1.1	unknown	unknown	Blood	1994	Asia	Southeast Asia	Vietnam	unknown	unknown	Wong et al, 2015
dn20	ERR349551	N/A	4.3.1.1	unknown	unknown	Blood	1995	Asia	Southeast Asia	Vietnam	unknown	unknown	Wong et al, 2015

dtc21	ERR349552	N/A	4.3.1.1	unknown	unknown	Blood	1994	Asia	Southeast Asia	Vietnam	unknown	unknown	Wong et al, 2015
dn28	ERR349553	N/A	4.3.1.1	unknown	unknown	Blood	1995	Asia	Southeast Asia	Vietnam	unknown	unknown	Wong et al, 2015
ipt43	ERR349556	N/A	4.3.1.1	unknown	unknown	Blood	1997	Asia	Southeast Asia	Vietnam	unknown	unknown	Wong et al, 2015
ty2-160	ERR349557	N/A	4.3.1.1	unknown	unknown	Blood	1994	Asia	Southeast Asia	Vietnam	unknown	unknown	Wong et al, 2015
ipt47	ERR349558	N/A	4.3.1.1	unknown	unknown	Blood	1997	Asia	Southeast Asia	Vietnam	unknown	unknown	Wong et al, 2015
ty2-166	ERR349559	N/A	2.2	unknown	unknown	Blood	1994	Asia	Southeast Asia	Vietnam	unknown	unknown	Wong et al, 2015
ct1-45	ERR349560	N/A	4.3.1.1	unknown	unknown	Blood	1994	Asia	Southeast Asia	Vietnam	unknown	unknown	Wong et al, 2015
dty1-75	ERR349562	N/A	4.3.1.1	unknown	unknown	Blood	1997	Asia	Southeast Asia	Vietnam	unknown	unknown	Wong et al, 2015
ty2-170	ERR349563	N/A	4.3.1.1	unknown	unknown	Blood	1995	Asia	Southeast Asia	Vietnam	unknown	unknown	Wong et al, 2015
ty3-253	ERR349564	N/A	4.3.1.1	unknown	unknown	Blood	1998	Asia	Southeast Asia	Vietnam	unknown	unknown	Wong et al, 2015
as16	ERR349565	N/A	2.3.2	unknown	unknown	Blood	1998	Asia	Southeast Asia	Vietnam	unknown	unknown	Wong et al, 2015
ipt79	ERR349568	N/A	4.3.1.1	unknown	unknown	Blood	1997	Asia	Southeast Asia	Vietnam	unknown	unknown	Wong et al, 2015
dty1-86	ERR349569	N/A	4.3.1.1	unknown	unknown	Blood	1997	Asia	Southeast Asia	Vietnam	unknown	unknown	Wong et al, 2015
ct1-23	ERR349570	N/A	4.3.1.1	unknown	unknown	Blood	1993	Asia	Southeast Asia	Vietnam	unknown	unknown	Wong et al, 2015
ipt76	ERR349571	N/A	4.3.1.1	unknown	unknown	Blood	1997	Asia	Southeast Asia	Vietnam	unknown	unknown	Wong et al, 2015
dty1-88	ERR349572	N/A	4.3.1.1	unknown	unknown	Blood	1997	Asia	Southeast Asia	Vietnam	unknown	unknown	Wong et al, 2015
ty3-212	ERR349573	N/A	4.3.1.1	unknown	unknown	Blood	1997	Asia	Southeast Asia	Vietnam	unknown	unknown	Wong et al, 2015
as257	ERR349575	N/A	3.2.1	unknown	unknown	Blood	1999	Asia	Southeast Asia	Vietnam	unknown	unknown	Wong et al, 2015
dty1-89	ERR349576	N/A	4.3.1.1	unknown	unknown	Blood	1997	Asia	Southeast Asia	Vietnam	unknown	unknown	Wong et al, 2015
ct1-65	ERR349578	N/A	4.3.1.1	unknown	unknown	Blood	1994	Asia	Southeast Asia	Vietnam	unknown	unknown	Wong et al, 2015
dty1-46	ERR349579	N/A	4.3.1.1	unknown	unknown	Blood	1995	Asia	Southeast Asia	Vietnam	unknown	unknown	Wong et al, 2015
dty1-91	ERR349580	N/A	4.3.1.1	unknown	unknown	Blood	1997	Asia	Southeast Asia	Vietnam	unknown	unknown	Wong et al, 2015
dty1-58	ERR349581	N/A	4.3.1.1	unknown	unknown	Blood	1995	Asia	Southeast Asia	Vietnam	unknown	unknown	Wong et al, 2015
dty1-93	ERR349582	N/A	4.3.1.1	unknown	unknown	Blood	1997	Asia	Southeast Asia	Vietnam	unknown	unknown	Wong et al, 2015
ct1-33	ERR349583	N/A	4.3.1.1	unknown	unknown	Blood	1994	Asia	Southeast Asia	Vietnam	unknown	unknown	Wong et al, 2015
dty1-67	ERR349584	N/A	4.3.1.1	unknown	unknown	Blood	1995	Asia	Southeast Asia	Vietnam	unknown	unknown	Wong et al, 2015
ty3-215	ERR349585	N/A	4.3.1.1	unknown	unknown	Blood	1997	Asia	Southeast Asia	Vietnam	unknown	unknown	Wong et al, 2015
ty2-76	ERR349588	N/A	4.3.1.1	unknown	unknown	Blood	1994	Asia	Southeast Asia	Vietnam	unknown	unknown	Wong et al, 2015
ty2-17	ERR349590	N/A	4.3.1.1	unknown	unknown	Blood	1993	Asia	Southeast Asia	Vietnam	unknown	unknown	Wong et al, 2015
ty2-90	ERR349591	N/A	4.3.1.1	unknown	unknown	Blood	1994	Asia	Southeast Asia	Vietnam	unknown	unknown	Wong et al, 2015
ty2-139	ERR349592	N/A	4.3.1.1	unknown	unknown	Blood	1994	Asia	Southeast Asia	Vietnam	unknown	unknown	Wong et al, 2015
ty2-123	ERR349593	N/A	4.3.1.1	unknown	unknown	Blood	1994	Asia	Southeast Asia	Vietnam	unknown	unknown	Wong et al, 2015
ty2-144	ERR349595	N/A	4.3.1.1	unknown	unknown	Blood	1994	Asia	Southeast Asia	Vietnam	unknown	unknown	Wong et al, 2015
ty2-146	ERR349597	N/A	4.3.1.1	unknown	unknown	Blood	1994	Asia	Southeast Asia	Vietnam	unknown	unknown	Wong et al, 2015
ty2-100	ERR349599	N/A	4.3.1.1	unknown	unknown	Blood	1994	Asia	Southeast Asia	Vietnam	unknown	unknown	Wong et al, 2015
ty2-114	ERR349600	N/A	4.3.1.1	unknown	unknown	Blood	1994	Asia	Southeast Asia	Vietnam	unknown	unknown	Wong et al, 2015
ty2-131	ERR349601	N/A	4.3.1.1	unknown	unknown	Blood	1994	Asia	Southeast Asia	Vietnam	unknown	unknown	Wong et al, 2015
ty2-71	ERR349602	N/A	4.3.1.1	unknown	unknown	Blood	1994	Asia	Southeast Asia	Vietnam	unknown	unknown	Wong et al, 2015
ty2-101	ERR349603	N/A	4.3.1.1	unknown	unknown	Blood	1994	Asia	Southeast Asia	Vietnam	unknown	unknown	Wong et al, 2015
ty2-115	ERR349604	N/A	4.1	unknown	unknown	Blood	1994	Asia	Southeast Asia	Vietnam	unknown	unknown	Wong et al, 2015
ty2-132	ERR349605	N/A	4.3.1.1	unknown	unknown	Blood	1994	Asia	Southeast Asia	Vietnam	unknown	unknown	Wong et al, 2015
ty2-150	ERR349606	N/A	4.3.1.1	unknown	unknown	Blood	1994	Asia	Southeast Asia	Vietnam	unknown	unknown	Wong et al, 2015
ty2-153	ERR349609	N/A	4.3.1.1	unknown	unknown	Blood	1994	Asia	Southeast Asia	Vietnam	unknown	unknown	Wong et al, 2015
ty2-103	ERR349613	N/A	4.3.1.1	unknown	unknown	Blood	1994	Asia	Southeast Asia	Vietnam	unknown	unknown	Wong et al, 2015
ty2-134	ERR349615	N/A	4.3.1.1	unknown	unknown	Blood	1994	Asia	Southeast Asia	Vietnam	unknown	unknown	Wong et al, 2015

ty2-104	ERR349616	N/A	4.3.1.1	unknown	unknown	Blood	1994	Asia	Southeast Asia	Vietnam	unknown	unknown	Wong et al, 2015
ty2-155	ERR349617	N/A	4.3.1.1	unknown	unknown	Blood	1994	Asia	Southeast Asia	Vietnam	unknown	unknown	Wong et al, 2015
UI 3257	ERR352253	N/A	3.2.1	unknown	unknown	Blood	2003	Asia	Southeast Asia	Laos	unknown	unknown	Wong et al, 2015
MDUST106	ERR352254	N/A	3.1	unknown	unknown	Blood	2006	Asia	Western Asia	Lebanon	unknown	unknown	Wong et al, 2015
MDUST115	ERR352255	N/A	2.1.7.1	unknown	unknown	Stool	2009	Australia & Oceania	Oceania	Papua New Guinea	unknown	unknown	Wong et al, 2015
MDUST120	ERR352256	N/A	3.1	unknown	unknown	Stool	2009	Europe	Southern Europe	Malta	unknown	unknown	Wong et al, 2015
MDUST121	ERR352257	N/A	2.2.1	unknown	unknown	Blood	2010	Australia & Oceania	Australia	Australia	unknown	unknown	Wong et al, 2015
MDUST123	ERR352258	N/A	3.5.4	unknown	unknown	Blood	2010	Australia & Oceania	Oceania	Samoa	unknown	unknown	Wong et al, 2015
MDUST127	ERR352259	N/A	4.3.1.2	unknown	unknown	Blood	2011	Asia	South Asia	India	unknown	unknown	Wong et al, 2015
MDUST130	ERR352260	N/A	3	unknown	unknown	Stool	2011	Asia	Southeast Asia	Malaysia	unknown	unknown	Wong et al, 2015
MDUST133	ERR352261	N/A	3.5.4	unknown	unknown	Blood	2011	Australia & Oceania	Oceania	Samoa	unknown	unknown	Wong et al, 2015
MDUST135	ERR352262	N/A	2.3.5	unknown	unknown	Blood	2011	Australia & Oceania	Oceania	Tonga	unknown	unknown	Wong et al, 2015
MDUST139	ERR352263	N/A	3.3.1	unknown	unknown	Stool	2012	Asia	Southeast Asia	Myanmar	unknown	unknown	Wong et al, 2015
MDUST141	ERR352264	N/A	4.3.1.1	unknown	unknown	Blood	2012	Asia	South Asia	India	unknown	unknown	Wong et al, 2015
MDUST145	ERR352265	N/A	4.3.1.2	unknown	unknown	Blood	2012	Asia	South Asia	India	unknown	unknown	Wong et al, 2015
MDUST147	ERR352266	N/A	4.3.1.2	unknown	unknown	Blood	2012	Asia	South Asia	India	unknown	unknown	Wong et al, 2015
MDUST149	ERR352267	N/A	3.0.2	unknown	unknown	Blood	2012	Asia	South Asia	India	unknown	unknown	Wong et al, 2015
MDUST151	ERR352268	N/A	4.3.1.2	unknown	unknown	Blood	2012	Unknown	Unknown	Unknown	unknown	unknown	Wong et al, 2015
MDUST154	ERR352269	N/A	2.3.4	unknown	unknown	Stool	2011	Asia	East Asia	China	unknown	unknown	Wong et al, 2015
MDUST158	ERR352270	N/A	4.3.1.2	unknown	unknown	Blood	2012	Asia	South Asia	India	unknown	unknown	Wong et al, 2015
MDUST161	ERR352271	N/A	4.3.1.2	unknown	unknown	Blood	2011	Asia	South Asia	India	unknown	unknown	Wong et al, 2015
MDUST166	ERR352272	N/A	4.3.1.1	unknown	unknown	Blood	2011	Asia	South Asia	India	unknown	unknown	Wong et al, 2015
MDUST169	ERR352273	N/A	4.3.1.2	unknown	unknown	Blood	2011	Asia	South Asia	India	unknown	unknown	Wong et al, 2015
MDUST182	ERR352274	N/A	4.3.1.1	unknown	unknown	Blood	2011	Asia	South Asia	India	unknown	unknown	Wong et al, 2015
MDUST185	ERR352275	N/A	4.3.1	unknown	unknown	Blood	2011	Asia	South Asia	India	unknown	unknown	Wong et al, 2015
MDUST187	ERR352276	N/A	4.3.1.2	unknown	unknown	Blood	2011	Unknown	Unknown	Unknown	unknown	unknown	Wong et al, 2015
MDUST196	ERR352277	N/A	4.3.1.3.Bdq	unknown	unknown	Blood	2011	Asia	South Asia	Bangladesh	unknown	unknown	Wong et al, 2015
MDUST197	ERR352278	N/A	4.3.1.2	unknown	unknown	Blood	2010	Asia	South Asia	India	unknown	unknown	Wong et al, 2015
MDUST198	ERR352279	N/A	4.3.1.1	unknown	unknown	Blood	2010	Asia	Southeast Asia	Myanmar	unknown	unknown	Wong et al, 2015
MDUST199	ERR352280	N/A	4.3.1.1	unknown	unknown	Blood	2011	Asia	South Asia	India	unknown	unknown	Wong et al, 2015
MDUST200	ERR352281	N/A	4.3.1	unknown	unknown	Blood	2011	Asia	South Asia	India	unknown	unknown	Wong et al, 2015
MDUST206	ERR352282	N/A	3	unknown	unknown	Blood	2010	Asia	Southeast Asia	Indonesia	unknown	unknown	Wong et al, 2015
MDUST211	ERR352283	N/A	4.3.1.2	unknown	unknown	Blood	2011	Asia	South Asia	India	unknown	unknown	Wong et al, 2015
MDUST216	ERR352284	N/A	4.3.1.2	unknown	unknown	Blood	2011	Asia	South Asia	India	unknown	unknown	Wong et al, 2015
MDUST217	ERR352285	N/A	3.2.1	unknown	unknown	Blood	2011	Asia	South Asia	India	unknown	unknown	Wong et al, 2015
MDUST223	ERR352286	N/A	4.3.1.2	unknown	unknown	Blood	2011	Unknown	Unknown	Unknown	unknown	unknown	Wong et al, 2015
MDUST226	ERR352287	N/A	2.1.7.1	unknown	unknown	Blood	2010	Australia & Oceania	Oceania	Papua New Guinea	unknown	unknown	Wong et al, 2015
MDUST229	ERR352288	N/A	2.1.7.1	unknown	unknown	Blood	2010	Australia & Oceania	Oceania	Papua New Guinea	unknown	unknown	Wong et al, 2015
MDUST231	ERR352289	N/A	4.3.1.2	unknown	unknown	Blood	2010	Asia	Western Asia	Lebanon	unknown	unknown	Wong et al, 2015
MDUST234	ERR352290	N/A	3.3.2.Bd2	unknown	unknown	Blood	2010	Asia	South Asia	Bangladesh	unknown	unknown	Wong et al, 2015

MDUST237	ERR352291	N/A	4.3.1.2	unknown	unknown	Stool	2010	Asia	South Asia	Nepal	unknown	unknown	Wong et al, 2015
MDUST239	ERR352292	N/A	3.5.4	unknown	unknown	Blood	2011	Unknown	Unknown	Unknown	unknown	unknown	Wong et al, 2015
MDUST241	ERR352293	N/A	4.3.1.3.Bdq	unknown	unknown	Blood	2010	Asia	South Asia	Bangladesh	unknown	unknown	Wong et al, 2015
MDUST242	ERR352294	N/A	4.3.1.1	unknown	unknown	Blood	2011	Asia	South Asia	India	unknown	unknown	Wong et al, 2015
MDUST243	ERR352295	N/A	4.3.1.2	unknown	unknown	Blood	2011	Asia	South Asia	India	unknown	unknown	Wong et al, 2015
MDUST248	ERR352296	N/A	4.3.1.2	unknown	unknown	Blood	2011	Asia	South Asia	India	unknown	unknown	Wong et al, 2015
MDUST249	ERR352297	N/A	4.3.1.2	unknown	unknown	Blood	2011	Asia	South Asia	India	unknown	unknown	Wong et al, 2015
MDUST252	ERR352298	N/A	3.3.2.Bd1	unknown	unknown	Blood	2010	Asia	South Asia	Bangladesh	unknown	unknown	Wong et al, 2015
MDUST253	ERR352299	N/A	3.1.2	unknown	unknown	Blood	2010	Asia	Southeast Asia	Indonesia	unknown	unknown	Wong et al, 2015
MDUST255	ERR352300	N/A	2.1.7.1	unknown	unknown	Blood	2010	Australia & Oceania	Oceania	Papua New Guinea	unknown	unknown	Wong et al, 2015
MDUST256	ERR352301	N/A	2.1.7.1	unknown	unknown	Blood	2010	Australia & Oceania	Oceania	Papua New Guinea	unknown	unknown	Wong et al, 2015
MDUST257	ERR352302	N/A	2.1.5	unknown	unknown	Blood	2010	Asia	Southeast Asia	Indonesia	unknown	unknown	Wong et al, 2015
MDUST258	ERR352303	N/A	3.2.2	unknown	unknown	Stool	2010	Asia	South Asia	Bangladesh	unknown	unknown	Wong et al, 2015
MDUST259	ERR352304	N/A	4.3.1.1	unknown	unknown	Blood	2010	Asia	South Asia	Bangladesh	unknown	unknown	Wong et al, 2015
MDUST265	ERR352305	N/A	4.3.1.3.Bdq	unknown	unknown	Blood	2011	Unknown	Unknown	Unknown	unknown	unknown	Wong et al, 2015
MDUST267	ERR352306	N/A	2.5	unknown	unknown	Blood	2011	Asia	South Asia	India	unknown	unknown	Wong et al, 2015
MDUST269	ERR352307	N/A	4.3.1.1	unknown	unknown	Blood	2011	Asia	South Asia	Pakistan	unknown	unknown	Wong et al, 2015
MDUST270	ERR352308	N/A	4.3.1.1	unknown	unknown	Stool	2011	Asia	South Asia	Bangladesh	unknown	unknown	Wong et al, 2015
MDUST274	ERR352309	N/A	2.1.7.2	unknown	unknown	Blood	1996	Australia & Oceania	Oceania	Papua New Guinea	unknown	unknown	Wong et al, 2015
MDUST295	ERR352310	N/A	2.1	unknown	unknown	Blood	2010	Asia	Southeast Asia	Indonesia	unknown	unknown	Wong et al, 2015
MDUST310	ERR352311	N/A	3.5	unknown	unknown	Stool	1991	Australia & Oceania	Oceania	Fiji	unknown	unknown	Wong et al, 2015
MDUST319	ERR352312	N/A	4.2.1	unknown	unknown	Blood	1983	Australia & Oceania	Oceania	Fiji	unknown	unknown	Wong et al, 2015
MDUST326	ERR352313	N/A	4.3.1.1	unknown	unknown	Stool	1992	Australia & Oceania	Oceania	Fiji	unknown	unknown	Wong et al, 2015
MDUST328	ERR352314	N/A	2.1.7.1	unknown	unknown	Blood	1992	Australia & Oceania	Oceania	Papua New Guinea	unknown	unknown	Wong et al, 2015
MDUST335	ERR352315	N/A	4.3.1.1	unknown	unknown	Stool	1993	Australia & Oceania	Oceania	Fiji	unknown	unknown	Wong et al, 2015
MDUST337	ERR352316	N/A	4.2.1	unknown	unknown	Blood	1984	Australia & Oceania	Oceania	Fiji	unknown	unknown	Wong et al, 2015
MDUST354	ERR352317	N/A	4.2.3	unknown	unknown	Blood	1993	Australia & Oceania	Oceania	Fiji	unknown	unknown	Wong et al, 2015
MDUST355	ERR352318	N/A	2.1.7	unknown	unknown	Blood	1994	Australia & Oceania	Oceania	Papua New Guinea	unknown	unknown	Wong et al, 2015
MDUST361	ERR352319	N/A	2.1.7.1	unknown	unknown	Stool	1998	Australia & Oceania	Oceania	Papua New Guinea	unknown	unknown	Wong et al, 2015
MDUST362	ERR352320	N/A	2.1.7.1	unknown	unknown	Blood	1998	Australia & Oceania	Oceania	Papua New Guinea	unknown	unknown	Wong et al, 2015
MDUST363	ERR352321	N/A	2.1.7.1	unknown	unknown	Blood	1998	Australia & Oceania	Oceania	Papua New Guinea	unknown	unknown	Wong et al, 2015
MDUST364	ERR352322	N/A	2.1.7.1	unknown	unknown	Stool	1999	Australia & Oceania	Oceania	Papua New Guinea	unknown	unknown	Wong et al, 2015
MDUST366	ERR352323	N/A	3.3.1	unknown	unknown	Blood	2011	Asia	Western Asia	Iraq	unknown	unknown	Wong et al, 2015
MDUST382	ERR352324	N/A	3.3.1	unknown	unknown	Stool	2011	Asia	South Asia	India	unknown	unknown	Wong et al, 2015
MDUST385	ERR352325	N/A	4.3.1.2	unknown	unknown	Blood	2011	Asia	South Asia	India	unknown	unknown	Wong et al, 2015
MDUST386	ERR352326	N/A	4.3.1.1	unknown	unknown	Blood	2011	Asia	Western Asia	Iraq	unknown	unknown	Wong et al, 2015

MDUST387	ERR352327	N/A	4.3.1	unknown	unknown	Blood	2011	Asia	South Asia	India	unknown	unknown	Wong et al, 2015
MDUST391	ERR352328	N/A	2.2	unknown	unknown	Blood	2011	Asia	South Asia	India	unknown	unknown	Wong et al, 2015
MDUST393	ERR352329	N/A	4.3.1.2	unknown	unknown	Stool	2011	Asia	South Asia	India	unknown	unknown	Wong et al, 2015
MDUST396	ERR352330	N/A	2.3.2	unknown	unknown	Stool	2012	South America	South America	El Salvador	unknown	unknown	Wong et al, 2015
MDUST397	ERR352331	N/A	3	unknown	unknown	Blood	2011	Asia	Southeast Asia	Indonesia	unknown	unknown	Wong et al, 2015
MDUST403	ERR352332	N/A	4.3.1.1	unknown	unknown	Blood	2012	Asia	Southeast Asia	Cambodia	unknown	unknown	Wong et al, 2015
MDUST404	ERR352333	N/A	4.2.2	unknown	unknown	Blood	2012	Australia & Oceania	Oceania	Fiji	unknown	unknown	Wong et al, 2015
MDUST406	ERR352334	N/A	3.5.4	unknown	unknown	Blood	2012	Australia & Oceania	Oceania	Samoa	unknown	unknown	Wong et al, 2015
MDUST408	ERR352335	N/A	4.3.1.2	unknown	unknown	Blood	2012	Asia	South Asia	India	unknown	unknown	Wong et al, 2015
MDUST410	ERR352336	N/A	4.3.1	unknown	unknown	Blood	2011	Asia	South Asia	India	unknown	unknown	Wong et al, 2015
MDUST411	ERR352337	N/A	4.1	unknown	unknown	Blood	2011	Unknown	Unknown	Unknown	unknown	unknown	Wong et al, 2015
MDUST412	ERR352338	N/A	4.3.1.2	unknown	unknown	Stool	2011	Asia	South Asia	Nepal	unknown	unknown	Wong et al, 2015
MDUST418	ERR352339	N/A	4.3.1	unknown	unknown	Blood	2011	Asia	South Asia	Pakistan	unknown	unknown	Wong et al, 2015
MDUST108	ERR352426	N/A	3.1	unknown	unknown	Stool	2007	Africa	West Africa	Liberia	unknown	unknown	Wong et al, 2015
MDUST109	ERR352427	N/A	2.1.7.1	unknown	unknown	Blood	2007	Australia & Oceania	Oceania	Papua New Guinea	unknown	unknown	Wong et al, 2015
MDUST113	ERR352428	N/A	2.2.1	unknown	unknown	Blood	2009	Asia	Western Asia	Lebanon	unknown	unknown	Wong et al, 2015
MDUST116	ERR352429	N/A	3.5.4	unknown	unknown	Blood	2009	Australia & Oceania	Oceania	Samoa	unknown	unknown	Wong et al, 2015
MDUST117	ERR352430	N/A	3.5.4	unknown	unknown	Blood	2009	Australia & Oceania	Oceania	Samoa	unknown	unknown	Wong et al, 2015
MDUST118	ERR352431	N/A	3.5.4	unknown	unknown	Blood	2009	Australia & Oceania	Oceania	Samoa	unknown	unknown	Wong et al, 2015
MDUST125	ERR352432	N/A	3.5.4	unknown	unknown	Blood	2011	Australia & Oceania	Oceania	Samoa	unknown	unknown	Wong et al, 2015
MDUST126	ERR352433	N/A	2.2.1	unknown	unknown	Blood	2010	Asia	Southeast Asia	Malaysia	unknown	unknown	Wong et al, 2015
MDUST128	ERR352434	N/A	4.3.1.2	unknown	unknown	Blood	2011	Asia	South Asia	India	unknown	unknown	Wong et al, 2015
MDUST129	ERR352435	N/A	3	unknown	unknown	Blood	2011	Asia	Southeast Asia	Phillipines	unknown	unknown	Wong et al, 2015
MDUST131	ERR352436	N/A	3.3	unknown	unknown	Blood	2011	Unknown	Unknown	Unknown	unknown	unknown	Wong et al, 2015
MDUST132	ERR352437	N/A	3.5.4	unknown	unknown	Blood	2011	Australia & Oceania	Oceania	Samoa	unknown	unknown	Wong et al, 2015
MDUST136	ERR352438	N/A	3.3.1	unknown	unknown	Blood	2011	Asia	South Asia	India	unknown	unknown	Wong et al, 2015
MDUST140	ERR352439	N/A	4.3.1.2	unknown	unknown	Blood	2011	Asia	South Asia	India	unknown	unknown	Wong et al, 2015
MDUST143	ERR352440	N/A	2.2.1	unknown	unknown	Stool	2012	Asia	South Asia	India	unknown	unknown	Wong et al, 2015
MDUST150	ERR352441	N/A	3	unknown	unknown	Blood	2012	Asia	Southeast Asia	Indonesia	unknown	unknown	Wong et al, 2015
MDUST152	ERR352442	N/A	4.3.1.1.EA1	unknown	unknown	Blood	2012	Africa	Africa	Africa	unknown	unknown	Wong et al, 2015
MDUST153	ERR352443	N/A	4.3.1	unknown	unknown	Blood	2012	Asia	South Asia	India	unknown	unknown	Wong et al, 2015
MDUST156	ERR352444	N/A	4.3.1.1	unknown	unknown	Blood	2012	Asia	South Asia	Pakistan	unknown	unknown	Wong et al, 2015
MDUST157	ERR352445	N/A	4.3.1.2	unknown	unknown	Stool	2012	Asia	South Asia	Nepal	unknown	unknown	Wong et al, 2015
MDUST159	ERR352446	N/A	4.3.1	unknown	unknown	Blood	2012	Asia	South Asia	India	unknown	unknown	Wong et al, 2015
MDUST162	ERR352447	N/A	4.3.1.2	unknown	unknown	Blood	2011	Unknown	Unknown	Unknown	unknown	unknown	Wong et al, 2015
MDUST163	ERR352448	N/A	4.3.1.2	unknown	unknown	Blood	2011	Asia	South Asia	India	unknown	unknown	Wong et al, 2015
MDUST168	ERR352449	N/A	4.3.1	unknown	unknown	Blood	2011	Asia	South Asia	India	unknown	unknown	Wong et al, 2015
MDUST174	ERR352451	N/A	4.3.1	unknown	unknown	Blood	2011	Asia	South Asia	Sri Lanka	unknown	unknown	Wong et al, 2015
MDUST175	ERR352452	N/A	4.3.1	unknown	unknown	Blood	2011	Asia	South Asia	Pakistan	unknown	unknown	Wong et al, 2015
MDUST177	ERR352453	N/A	1.1.4	unknown	unknown	Urine	2011	Unknown	Unknown	Unknown	unknown	unknown	Wong et al, 2015

MDUST179	ERR352454	N/A	3.2.2	unknown	unknown	Blood	2011	Asia	South Asia	India	unknown	unknown	Wong et al, 2015
MDUST183	ERR352455	N/A	4.3.1.2	unknown	unknown	Blood	2011	Asia	South Asia	India	unknown	unknown	Wong et al, 2015
MDUST184	ERR352456	N/A	4.3.1.1	unknown	unknown	Blood	2011	Asia	South Asia	India	unknown	unknown	Wong et al, 2015
MDUST194	ERR352457	N/A	3.2.2	unknown	unknown	Blood	2011	Asia	South Asia	India	unknown	unknown	Wong et al, 2015
MDUST201	ERR352458	N/A	4.3.1.2	unknown	unknown	Blood	2011	Asia	South Asia	India	unknown	unknown	Wong et al, 2015
MDUST202	ERR352459	N/A	2	unknown	unknown	Blood	2010	Asia	South Asia	Bangladesh	unknown	unknown	Wong et al, 2015
MDUST203	ERR352460	N/A	2	unknown	unknown	Blood	2010	Asia	South Asia	Bangladesh	unknown	unknown	Wong et al, 2015
MDUST204	ERR352461	N/A	2	unknown	unknown	Blood	2010	Asia	South Asia	Bangladesh	unknown	unknown	Wong et al, 2015
MDUST205	ERR352462	N/A	2	unknown	unknown	Blood	2010	Asia	South Asia	Bangladesh	unknown	unknown	Wong et al, 2015
MDUST207	ERR352463	N/A	4.3.1.1	unknown	unknown	Blood	2010	Asia	South Asia	Pakistan	unknown	unknown	Wong et al, 2015
MDUST214	ERR352464	N/A	4.3.1.2	unknown	unknown	Blood	2011	Asia	South Asia	India	unknown	unknown	Wong et al, 2015
MDUST215	ERR352465	N/A	3	unknown	unknown	Stool	2011	Asia	South Asia	India	unknown	unknown	Wong et al, 2015
MDUST220	ERR352466	N/A	4.3.1.1	unknown	unknown	Blood	2011	Asia	South Asia	India	unknown	unknown	Wong et al, 2015
MDUST222	ERR352467	N/A	2.1.5	unknown	unknown	Blood	2011	Asia	Southeast Asia	Indonesia	unknown	unknown	Wong et al, 2015
MDUST232	ERR352468	N/A	4.3.1.1	unknown	unknown	Blood	2010	Asia	South Asia	Bangladesh	unknown	unknown	Wong et al, 2015
MDUST235	ERR352469	N/A	3.2.1	unknown	unknown	Stool	2010	Asia	Southeast Asia	Vietnam	unknown	unknown	Wong et al, 2015
MDUST236	ERR352470	N/A	4.3.1.1	unknown	unknown	Blood	2010	Asia	Southeast Asia	Thailand	unknown	unknown	Wong et al, 2015
MDUST244	ERR352471	N/A	4.3.1.2	unknown	unknown	Blood	2011	Asia	South Asia	India	unknown	unknown	Wong et al, 2015
MDUST246	ERR352472	N/A	2.1	unknown	unknown	Blood	2011	Asia	Southeast Asia	Indonesia	unknown	unknown	Wong et al, 2015
MDUST250	ERR352473	N/A	0.0.2	unknown	unknown	Blood	2010	Asia	Southeast Asia	Indonesia	unknown	unknown	Wong et al, 2015
MDUST261	ERR352474	N/A	4.3.1	unknown	unknown	Blood	2011	Unknown	Unknown	Unknown	unknown	unknown	Wong et al, 2015
MDUST268	ERR352475	N/A	2.5	unknown	unknown	Blood	2011	Unknown	Unknown	Unknown	unknown	unknown	Wong et al, 2015
MDUST276	ERR352476	N/A	2.1.7.2	unknown	unknown	Stool	1994	Australia & Oceania	Oceania	Papua New Guinea	unknown	unknown	Wong et al, 2015
MDUST278	ERR352477	N/A	2.1.7.1	unknown	unknown	Blood	2001	Australia & Oceania	Oceania	Papua New Guinea	unknown	unknown	Wong et al, 2015
MDUST279	ERR352478	N/A	2.1.7.1	unknown	unknown	Blood	2002	Australia & Oceania	Oceania	Papua New Guinea	unknown	unknown	Wong et al, 2015
MDUST282	ERR352479	N/A	3.5.4	unknown	unknown	Blood	2004	Australia & Oceania	Oceania	Samoa	unknown	unknown	Wong et al, 2015
MDUST283	ERR352480	N/A	3.5.4	unknown	unknown	Blood	2004	Australia & Oceania	Oceania	Samoa	unknown	unknown	Wong et al, 2015
MDUST286	ERR352481	N/A	3.5.4	unknown	unknown	Blood	2005	Australia & Oceania	Oceania	Samoa	unknown	unknown	Wong et al, 2015
MDUST287	ERR352482	N/A	2.1.7.1	unknown	unknown	Blood	2007	Australia & Oceania	Oceania	Papua New Guinea	unknown	unknown	Wong et al, 2015
MDUST288	ERR352483	N/A	2.1.7.1	unknown	unknown	Blood	2007	Australia & Oceania	Oceania	Papua New Guinea	unknown	unknown	Wong et al, 2015
MDUST289	ERR352484	N/A	3.5.4	unknown	unknown	Blood	2008	Australia & Oceania	Oceania	Samoa	unknown	unknown	Wong et al, 2015
MDUST291	ERR352485	N/A	3.5.4	unknown	unknown	Stool	2008	Australia & Oceania	Oceania	Samoa	unknown	unknown	Wong et al, 2015
MDUST292	ERR352486	N/A	3.5.4	unknown	unknown	Blood	2008	Australia & Oceania	Oceania	Samoa	unknown	unknown	Wong et al, 2015
MDUST296	ERR352487	N/A	2.1.7.1	unknown	unknown	Blood	2010	Australia & Oceania	Oceania	Papua New Guinea	unknown	unknown	Wong et al, 2015
MDUST297	ERR352488	N/A	3.5.4	unknown	unknown	Blood	2011	Unknown	Unknown	Unknown	unknown	unknown	Wong et al, 2015
MDUST298	ERR352489	N/A	2.1.7.2	unknown	unknown	Blood	1998	Australia & Oceania	Oceania	Papua New Guinea	unknown	unknown	Wong et al, 2015

MDUST299	ERR352490	N/A	2.1.7.2	unknown	unknown	Blood	1998	Australia & Oceania	Oceania	Papua New Guinea	unknown	unknown	Wong et al, 2015
MDUST300	ERR352491	N/A	2.1.7.1	unknown	unknown	Stool	1998	Australia & Oceania	Oceania	Papua New Guinea	unknown	unknown	Wong et al, 2015
MDUST301	ERR352492	N/A	2.1.7.1	unknown	unknown	Stool	1988	Asia	South Asia	India	unknown	unknown	Wong et al, 2015
MDUST313	ERR352493	N/A	2.1.7.1	unknown	unknown	Blood	1994	Australia & Oceania	Oceania	Papua New Guinea	unknown	unknown	Wong et al, 2015
MDUST321	ERR352494	N/A	4.2.1	unknown	unknown	Blood	1983	Australia & Oceania	Oceania	Fiji	unknown	unknown	Wong et al, 2015
MDUST322	ERR352495	N/A	2.3.5	unknown	unknown	Blood	1983	Australia & Oceania	Oceania	Fiji	unknown	unknown	Wong et al, 2015
MDUST330	ERR352496	N/A	2.1.7.2	unknown	unknown	Blood	1992	Australia & Oceania	Oceania	Papua New Guinea	unknown	unknown	Wong et al, 2015
MDUST331	ERR352497	N/A	2.1.7.1	unknown	unknown	Blood	1992	Australia & Oceania	Oceania	Papua New Guinea	unknown	unknown	Wong et al, 2015
MDUST336	ERR352498	N/A	4.3.1.1	unknown	unknown	Stool	1993	Australia & Oceania	Oceania	Fiji	unknown	unknown	Wong et al, 2015
MDUST339	ERR352499	N/A	2.3.5	unknown	unknown	Stool	1984	Australia & Oceania	Oceania	Fiji	unknown	unknown	Wong et al, 2015
MDUST348	ERR352500	N/A	2.1.7.2	unknown	unknown	Unknown	1985	Australia & Oceania	Oceania	Papua New Guinea	unknown	unknown	Wong et al, 2015
MDUST351	ERR352501	N/A	3.5.1	unknown	unknown	Stool	1985	Australia & Oceania	Oceania	Fiji	unknown	unknown	Wong et al, 2015
MDUST352	ERR352502	N/A	2.3.5	unknown	unknown	Blood	1986	Australia & Oceania	Oceania	Fiji	unknown	unknown	Wong et al, 2015
MDUST358	ERR352503	N/A	2.1.7.2	unknown	unknown	Stool	1996	Australia & Oceania	Oceania	Papua New Guinea	unknown	unknown	Wong et al, 2015
MDUST368	ERR352504	N/A	4.3.1.2	unknown	unknown	Blood	2011	Asia	South Asia	India	unknown	unknown	Wong et al, 2015
MDUST383	ERR352505	N/A	4.3.1.1	unknown	unknown	Blood	2011	Asia	South Asia	India	unknown	unknown	Wong et al, 2015
MDUST388	ERR352506	N/A	4.3.1.1	unknown	unknown	Blood	2011	Asia	South Asia	India	unknown	unknown	Wong et al, 2015
MDUST389	ERR352507	N/A	4.3.1.2	unknown	unknown	Blood	2011	Asia	Southeast Asia	Indonesia	unknown	unknown	Wong et al, 2015
MDUST401	ERR352508	N/A	2.0.1	unknown	unknown	Blood	2012	Asia	South Asia	Bangladesh	unknown	unknown	Wong et al, 2015
MDUST402	ERR352509	N/A	4.3.1.1	unknown	unknown	Blood	2012	Asia	South Asia	Afghanistan	unknown	unknown	Wong et al, 2015
MDUST407	ERR352510	N/A	2.2.2	unknown	unknown	Stool	2007	Asia	Western Asia	Lebanon	unknown	unknown	Wong et al, 2015
MDUST415	ERR352511	N/A	3	unknown	unknown	Blood	2012	Unknown	Unknown	Unknown	unknown	unknown	Wong et al, 2015
3592/3	ERR352599	N/A	2.5.1	unknown	unknown	Blood	2011	Africa	Central Africa	DRC	unknown	unknown	Wong et al, 2015
3306/3	ERR352602	N/A	2.5.1	unknown	unknown	Blood	2011	Africa	Central Africa	DRC	unknown	unknown	Wong et al, 2015
3322/3	ERR352604	N/A	2.5.1	unknown	unknown	Blood	2011	Africa	Central Africa	DRC	unknown	unknown	Wong et al, 2015
dn51	ERR352605	N/A	4.3.1.1	unknown	unknown	Blood	1995	Asia	Southeast Asia	Vietnam	unknown	unknown	Wong et al, 2015
dn17	ERR352606	N/A	4.3.1.1	unknown	unknown	Blood	1995	Asia	Southeast Asia	Vietnam	unknown	unknown	Wong et al, 2015
dn40	ERR352607	N/A	4.3.1.1	unknown	unknown	Blood	1995	Asia	Southeast Asia	Vietnam	unknown	unknown	Wong et al, 2015
dtc84	ERR352608	N/A	4.3.1.1	unknown	unknown	Blood	1994	Asia	Southeast Asia	Vietnam	unknown	unknown	Wong et al, 2015
dn126	ERR352609	N/A	4.3.1.1	unknown	unknown	Blood	1996	Asia	Southeast Asia	Vietnam	unknown	unknown	Wong et al, 2015
dtc81	ERR352611	N/A	4.3.1.1	unknown	unknown	Blood	1994	Asia	Southeast Asia	Vietnam	unknown	unknown	Wong et al, 2015
dn192	ERR352612	N/A	4.3.1.1	unknown	unknown	Blood	1996	Asia	Southeast Asia	Vietnam	unknown	unknown	Wong et al, 2015
dn152	ERR352613	N/A	4.3.1.1	unknown	unknown	Blood	1996	Asia	Southeast Asia	Vietnam	unknown	unknown	Wong et al, 2015
dtc76	ERR352614	N/A	4.3.1.1	unknown	unknown	Blood	1994	Asia	Southeast Asia	Vietnam	unknown	unknown	Wong et al, 2015
dn95	ERR352615	N/A	4.3.1.1	unknown	unknown	Blood	1995	Asia	Southeast Asia	Vietnam	unknown	unknown	Wong et al, 2015
dn182	ERR352616	N/A	4.3.1.1	unknown	unknown	Blood	1996	Asia	Southeast Asia	Vietnam	unknown	unknown	Wong et al, 2015
dtc109	ERR352617	N/A	4.3.1.1	unknown	unknown	Blood	1994	Asia	Southeast Asia	Vietnam	unknown	unknown	Wong et al, 2015

dn38	ERR352618	N/A	4.3.1.1	unknown	unknown	Blood	1995	Asia	Southeast Asia	Vietnam	unknown	unknown	Wong et al, 2015
dn122	ERR352619	N/A	4.3.1.1	unknown	unknown	Blood	1996	Asia	Southeast Asia	Vietnam	unknown	unknown	Wong et al, 2015
dn154	ERR352620	N/A	4.3.1.1	unknown	unknown	Blood	1996	Asia	Southeast Asia	Vietnam	unknown	unknown	Wong et al, 2015
dn67	ERR352621	N/A	4.3.1.1	unknown	unknown	Blood	1995	Asia	Southeast Asia	Vietnam	unknown	unknown	Wong et al, 2015
dn174	ERR352623	N/A	4.3.1.1	unknown	unknown	Blood	1996	Asia	Southeast Asia	Vietnam	unknown	unknown	Wong et al, 2015
dn145	ERR352624	N/A	4.3.1.1	unknown	unknown	Blood	1996	Asia	Southeast Asia	Vietnam	unknown	unknown	Wong et al, 2015
dn56	ERR352625	N/A	4.3.1.1	unknown	unknown	Blood	1995	Asia	Southeast Asia	Vietnam	unknown	unknown	Wong et al, 2015
dtc115	ERR352626	N/A	4.3.1.1	unknown	unknown	Blood	1995	Asia	Southeast Asia	Vietnam	unknown	unknown	Wong et al, 2015
dn113	ERR352627	N/A	4.3.1.1	unknown	unknown	Blood	1996	Asia	Southeast Asia	Vietnam	unknown	unknown	Wong et al, 2015
dn26	ERR352628	N/A	4.3.1.1	unknown	unknown	Blood	1995	Asia	Southeast Asia	Vietnam	unknown	unknown	Wong et al, 2015
dn137	ERR352629	N/A	4.3.1.1	unknown	unknown	Blood	1996	Asia	Southeast Asia	Vietnam	unknown	unknown	Wong et al, 2015
dn178	ERR352631	N/A	4.3.1.1	unknown	unknown	Blood	1996	Asia	Southeast Asia	Vietnam	unknown	unknown	Wong et al, 2015
dn22	ERR352632	N/A	4.3.1.1	unknown	unknown	Blood	1995	Asia	Southeast Asia	Vietnam	unknown	unknown	Wong et al, 2015
dn89	ERR352633	N/A	4.3.1.1	unknown	unknown	Blood	1995	Asia	Southeast Asia	Vietnam	unknown	unknown	Wong et al, 2015
dtc80	ERR352635	N/A	4.3.1.1	unknown	unknown	Blood	1994	Asia	Southeast Asia	Vietnam	unknown	unknown	Wong et al, 2015
dn21	ERR352636	N/A	4.3.1.1	unknown	unknown	Blood	1995	Asia	Southeast Asia	Vietnam	unknown	unknown	Wong et al, 2015
dn176	ERR352637	N/A	4.3.1.1	unknown	unknown	Blood	1996	Asia	Southeast Asia	Vietnam	unknown	unknown	Wong et al, 2015
dn148	ERR352638	N/A	4.3.1.1	unknown	unknown	Blood	1996	Asia	Southeast Asia	Vietnam	unknown	unknown	Wong et al, 2015
dn149	ERR352639	N/A	4.3.1.1	unknown	unknown	Blood	1996	Asia	Southeast Asia	Vietnam	unknown	unknown	Wong et al, 2015
dn123	ERR352640	N/A	4.3.1.1	unknown	unknown	Blood	1996	Asia	Southeast Asia	Vietnam	unknown	unknown	Wong et al, 2015
dtc57	ERR352641	N/A	4.3.1.1	unknown	unknown	Blood	1994	Asia	Southeast Asia	Vietnam	unknown	unknown	Wong et al, 2015
dtc90	ERR352642	N/A	4.3.1.1	unknown	unknown	Blood	1994	Asia	Southeast Asia	Vietnam	unknown	unknown	Wong et al, 2015
ty3-201	ERR352643	N/A	4.3.1.1	unknown	unknown	Blood	1997	Asia	Southeast Asia	Vietnam	unknown	unknown	Wong et al, 2015
ty3-224	ERR352644	N/A	4.3.1.1	unknown	unknown	Blood	1997	Asia	Southeast Asia	Vietnam	unknown	unknown	Wong et al, 2015
ct1-105	ERR352645	N/A	4.3.1.1	unknown	unknown	Blood	1994	Asia	Southeast Asia	Vietnam	unknown	unknown	Wong et al, 2015
ty3-202	ERR352646	N/A	4.3.1.1	unknown	unknown	Blood	1997	Asia	Southeast Asia	Vietnam	unknown	unknown	Wong et al, 2015
ty3-226	ERR352647	N/A	4.3.1.1	unknown	unknown	Blood	1997	Asia	Southeast Asia	Vietnam	unknown	unknown	Wong et al, 2015
ct1-107	ERR352648	N/A	4.3.1.1	unknown	unknown	Blood	1994	Asia	Southeast Asia	Vietnam	unknown	unknown	Wong et al, 2015
ty3-205	ERR352649	N/A	4.3.1.1	unknown	unknown	Blood	1997	Asia	Southeast Asia	Vietnam	unknown	unknown	Wong et al, 2015
ty3-243	ERR352650	N/A	4.3.1.1	unknown	unknown	Blood	1998	Asia	Southeast Asia	Vietnam	unknown	unknown	Wong et al, 2015
ipt51	ERR352651	N/A	4.3.1.1	unknown	unknown	Blood	1997	Asia	Southeast Asia	Vietnam	unknown	unknown	Wong et al, 2015
ty3-206	ERR352652	N/A	4.3.1.1	unknown	unknown	Blood	1997	Asia	Southeast Asia	Vietnam	unknown	unknown	Wong et al, 2015
dty1-80	ERR352654	N/A	4.3.1.1	unknown	unknown	Blood	1997	Asia	Southeast Asia	Vietnam	unknown	unknown	Wong et al, 2015
ty2-173	ERR352655	N/A	4.3.1.1	unknown	unknown	Blood	1995	Asia	Southeast Asia	Vietnam	unknown	unknown	Wong et al, 2015
ty3-207	ERR352656	N/A	4.3.1.1	unknown	unknown	Blood	1997	Asia	Southeast Asia	Vietnam	unknown	unknown	Wong et al, 2015
ty3-254	ERR352657	N/A	4.3.1.1	unknown	unknown	Blood	1998	Asia	Southeast Asia	Vietnam	unknown	unknown	Wong et al, 2015
ct1-66	ERR352658	N/A	4.3.1.1	unknown	unknown	Blood	1994	Asia	Southeast Asia	Vietnam	unknown	unknown	Wong et al, 2015
as118	ERR352659	N/A	4.3.1.1	unknown	unknown	Blood	1998	Asia	Southeast Asia	Vietnam	unknown	unknown	Wong et al, 2015
ipt61	ERR352661	N/A	4.3.1.1	unknown	unknown	Blood	1997	Asia	Southeast Asia	Vietnam	unknown	unknown	Wong et al, 2015
ty3-191	ERR352662	N/A	4.3.1.1	unknown	unknown	Blood	1997	Asia	Southeast Asia	Vietnam	unknown	unknown	Wong et al, 2015
ty3-269	ERR352663	N/A	4.3.1.1	unknown	unknown	Blood	1998	Asia	Southeast Asia	Vietnam	unknown	unknown	Wong et al, 2015
as250	ERR352665	N/A	4.3.1.1	unknown	unknown	Blood	1999	Asia	Southeast Asia	Vietnam	unknown	unknown	Wong et al, 2015
ty3-211	ERR352667	N/A	4.3.1.1	unknown	unknown	Blood	1997	Asia	Southeast Asia	Vietnam	unknown	unknown	Wong et al, 2015
ct1-70	ERR352669	N/A	4.3.1.1	unknown	unknown	Blood	1994	Asia	Southeast Asia	Vietnam	unknown	unknown	Wong et al, 2015
as252	ERR352671	N/A	4.3.1.1	unknown	unknown	Blood	1999	Asia	Southeast Asia	Vietnam	unknown	unknown	Wong et al, 2015

ty3-194	ERR352673	N/A	4.3.1.1	unknown	unknown	Blood	1997	Asia	Southeast Asia	Vietnam	unknown	unknown	Wong et al, 2015
ipt83	ERR352677	N/A	4.3.1.1	unknown	unknown	Blood	1998	Asia	Southeast Asia	Vietnam	unknown	unknown	Wong et al, 2015
ty3-196	ERR352679	N/A	4.3.1.1	unknown	unknown	Blood	1997	Asia	Southeast Asia	Vietnam	unknown	unknown	Wong et al, 2015
ty3-222	ERR352681	N/A	4.3.1.1	unknown	unknown	Blood	1997	Asia	Southeast Asia	Vietnam	unknown	unknown	Wong et al, 2015
ty3-197	ERR352685	N/A	4.3.1.1	unknown	unknown	Blood	1997	Asia	Southeast Asia	Vietnam	unknown	unknown	Wong et al, 2015
ty3-213	ERR352687	N/A	3.2.1	unknown	unknown	Blood	1997	Asia	Southeast Asia	Vietnam	unknown	unknown	Wong et al, 2015
ct1-69	ERR352689	N/A	4.3.1.1	unknown	unknown	Blood	1994	Asia	Southeast Asia	Vietnam	unknown	unknown	Wong et al, 2015
ipt16	ERR352691	N/A	4.3.1.1	unknown	unknown	Blood	1995	Asia	Southeast Asia	Vietnam	unknown	unknown	Wong et al, 2015
ipt19	ERR352697	N/A	4.3.1.1	unknown	unknown	Blood	1995	Asia	Southeast Asia	Vietnam	unknown	unknown	Wong et al, 2015
ty3-199	ERR352699	N/A	3.2.1	unknown	unknown	Blood	1997	Asia	Southeast Asia	Vietnam	unknown	unknown	Wong et al, 2015
ty2-105	ERR352703	N/A	4.3.1.1	unknown	unknown	Blood	1994	Asia	Southeast Asia	Vietnam	unknown	unknown	Wong et al, 2015
ty2-138	ERR352705	N/A	4.1	unknown	unknown	Blood	1994	Asia	Southeast Asia	Vietnam	unknown	unknown	Wong et al, 2015
ty2-106	ERR352709	N/A	4.3.1.1	unknown	unknown	Blood	1994	Asia	Southeast Asia	Vietnam	unknown	unknown	Wong et al, 2015
ty2-141	ERR352711	N/A	4.3.1.1	unknown	unknown	Blood	1994	Asia	Southeast Asia	Vietnam	unknown	unknown	Wong et al, 2015
ty2-143	ERR352713	N/A	4.3.1.1	unknown	unknown	Blood	1994	Asia	Southeast Asia	Vietnam	unknown	unknown	Wong et al, 2015
ty2-113	ERR352715	N/A	4.3.1.1	unknown	unknown	Blood	1994	Asia	Southeast Asia	Vietnam	unknown	unknown	Wong et al, 2015
ty2-116	ERR352717	N/A	4.3.1.1	unknown	unknown	Blood	1994	Asia	Southeast Asia	Vietnam	unknown	unknown	Wong et al, 2015
ty2-133	ERR352719	N/A	4.3.1.1	unknown	unknown	Blood	1994	Asia	Southeast Asia	Vietnam	unknown	unknown	Wong et al, 2015
ty2-154	ERR352721	N/A	4.3.1.1	unknown	unknown	Blood	1994	Asia	Southeast Asia	Vietnam	unknown	unknown	Wong et al, 2015
ty2-119	ERR352725	N/A	4.3.1.1	unknown	unknown	Blood	1994	Asia	Southeast Asia	Vietnam	unknown	unknown	Wong et al, 2015
MDUST107	ERR352941	N/A	2.1.7.1	unknown	unknown	Blood	2007	Australia & Oceania	Oceania	Papua New Guinea	unknown	unknown	Wong et al, 2015
MDUST111	ERR352942	N/A	3.3	unknown	unknown	Urine	2008	Africa	North Africa	Sudan	unknown	unknown	Wong et al, 2015
MDUST181	ERR352943	N/A	4.3.1.2	unknown	unknown	Blood	2011	Unknown	Unknown	Unknown	unknown	unknown	Wong et al, 2015
MDUST186	ERR352944	N/A	4.3.1.2	unknown	unknown	Blood	2011	Asia	South Asia	India	unknown	unknown	Wong et al, 2015
MDUST188	ERR352945	N/A	3	unknown	unknown	Blood	2011	Asia	Southeast Asia	Indonesia	unknown	unknown	Wong et al, 2015
MDUST189	ERR352946	N/A	4.3.1.2	unknown	unknown	Blood	2011	Asia	South Asia	India	unknown	unknown	Wong et al, 2015
MDUST208	ERR352947	N/A	2.2.1	unknown	unknown	Blood	2010	Australia & Oceania	Australia	Australia	unknown	unknown	Wong et al, 2015
MDUST209	ERR352948	N/A	4.3.1.1	unknown	unknown	Blood	2011	Asia	South Asia	India	unknown	unknown	Wong et al, 2015
MDUST210	ERR352949	N/A	4.3.1.2	unknown	unknown	Blood	2011	Asia	South Asia	India	unknown	unknown	Wong et al, 2015
MDUST212	ERR352950	N/A	2.2.2	unknown	unknown	Blood	2011	Asia	South Asia	India	unknown	unknown	Wong et al, 2015
MDUST213	ERR352951	N/A	4.3.1	unknown	unknown	Blood	2011	Asia	South Asia	India	unknown	unknown	Wong et al, 2015
MDUST219	ERR352952	N/A	2.2.2	unknown	unknown	Stool	2011	Asia	Western Asia	Lebanon	unknown	unknown	Wong et al, 2015
MDUST224	ERR352953	N/A	4.3.1.2	unknown	unknown	Blood	2011	Asia	South Asia	India	unknown	unknown	Wong et al, 2015
MDUST225	ERR352954	N/A	4.3.1.2	unknown	unknown	Blood	2011	Asia	South Asia	India	unknown	unknown	Wong et al, 2015
ST1134/01	ERR353330	N/A	2	unknown	unknown	Stool	2001	South America	South America	Argentina	unknown	unknown	Wong et al, 2015
ST1197/88	ERR353331	N/A	2.3.3	unknown	unknown	Urine	1988	South America	South America	Argentina	unknown	unknown	Wong et al, 2015
ST1309/04	ERR353332	N/A	2.3.3	unknown	unknown	Blood	2004	South America	South America	Argentina	unknown	unknown	Wong et al, 2015
ST1625/88	ERR353334	N/A	4.1	unknown	unknown	Stool	1988	South America	South America	Argentina	unknown	unknown	Wong et al, 2015
ST1921/06	ERR353335	N/A	2.3.3	unknown	unknown	Stool	2006	South America	South America	Argentina	unknown	unknown	Wong et al, 2015
ST2338/98	ERR353336	N/A	2.3.3	unknown	unknown	Blood	1905	South America	South America	Argentina	unknown	unknown	Wong et al, 2015
ST3090/99	ERR353338	N/A	2	unknown	unknown	Stool	1999	South America	South America	Argentina	unknown	unknown	Wong et al, 2015
ST472/01	ERR353339	N/A	4.1	unknown	unknown	Stool	1905	South America	South America	Argentina	unknown	unknown	Wong et al, 2015
ST805/02	ERR353340	N/A	2.3.2	unknown	unknown	Stool	2002	South America	South America	Argentina	unknown	unknown	Wong et al, 2015

ST821/98	ERR353341	N/A	4.1	unknown	unknown	Stool	1905	South America	South America	Argentina	unknown	unknown	Wong et al, 2015
ST860/95	ERR353342	N/A	2.3.2	unknown	unknown	Stool	1905	South America	South America	Argentina	unknown	unknown	Wong et al, 2015
MDUST114	ERR357440	N/A	3.5.4	unknown	unknown	Blood	2009	Australia & Oceania	Oceania	Samoa	unknown	unknown	Wong et al, 2015
MDUST138	ERR357441	N/A	3.3.1	unknown	unknown	Stool	2012	Asia	Southeast Asia	Myanmar	unknown	unknown	Wong et al, 2015
MDUST155	ERR357442	N/A	4.3.1.1	unknown	unknown	Blood	2012	Asia	South Asia	India	unknown	unknown	Wong et al, 2015
MDUST160	ERR357443	N/A	3.0.2	unknown	unknown	Stool	2012	Asia	South Asia	India	unknown	unknown	Wong et al, 2015
MDUST164	ERR357444	N/A	4.3.1.2	unknown	unknown	Blood	2011	Asia	South Asia	India	unknown	unknown	Wong et al, 2015
MDUST178	ERR357445	N/A	2.1	unknown	unknown	Stool	2011	Asia	Southeast Asia	EastTimor	unknown	unknown	Wong et al, 2015
MDUST180	ERR357446	N/A	4.3.1.2	unknown	unknown	Blood	2011	Asia	South Asia	India	unknown	unknown	Wong et al, 2015
MDUST277	ERR357447	N/A	2.1.7.1	unknown	unknown	Blood	2001	Australia & Oceania	Oceania	Papua New Guinea	unknown	unknown	Wong et al, 2015
MDUST281	ERR357449	N/A	2.1.7.1	unknown	unknown	Blood	2002	Australia & Oceania	Oceania	Papua New Guinea	unknown	unknown	Wong et al, 2015
MDUST284	ERR357450	N/A	4.1	unknown	unknown	Blood	2004	Australia & Oceania	Oceania	Samoa	unknown	unknown	Wong et al, 2015
MDUST285	ERR357451	N/A	4.1	unknown	unknown	Blood	2004	Australia & Oceania	Oceania	Samoa	unknown	unknown	Wong et al, 2015
MDUST293	ERR357452	N/A	2.1.7.1	unknown	unknown	Stool	2009	Australia & Oceania	Oceania	Papua New Guinea	unknown	unknown	Wong et al, 2015
MDUST294	ERR357453	N/A	4.3.1.2	unknown	unknown	Blood	2010	Asia	South Asia	Nepal	unknown	unknown	Wong et al, 2015
MDUST303	ERR357454	N/A	4.2	unknown	unknown	Unknown	1980	Australia & Oceania	Oceania	Tonga	unknown	unknown	Wong et al, 2015
MDUST304	ERR357455	N/A	2.1.7.1	unknown	unknown	Stool	1990	Australia & Oceania	Oceania	Papua New Guinea	unknown	unknown	Wong et al, 2015
MDUST305	ERR357456	N/A	2.1.7.1	unknown	unknown	Blood	1990	Australia & Oceania	Oceania	Papua New Guinea	unknown	unknown	Wong et al, 2015
MDUST306	ERR357457	N/A	4.2.1	unknown	unknown	Unknown	1981	Australia & Oceania	Oceania	Fiji	unknown	unknown	Wong et al, 2015
MDUST309	ERR357459	N/A	4.2	unknown	unknown	Unknown	1981	Australia & Oceania	Oceania	Fiji	unknown	unknown	Wong et al, 2015
MDUST311	ERR357460	N/A	4.2	unknown	unknown	Stool	1982	Australia & Oceania	Oceania	Fiji	unknown	unknown	Wong et al, 2015
MDUST312	ERR357461	N/A	4.2	unknown	unknown	Stool	1982	Australia & Oceania	Oceania	Fiji	unknown	unknown	Wong et al, 2015
MDUST315	ERR357462	N/A	4.1	unknown	unknown	Stool	1991	Australia & Oceania	Oceania	Vanuatu	unknown	unknown	Wong et al, 2015
MDUST316	ERR357463	N/A	4.2	unknown	unknown	Unknown	1982	Australia & Oceania	Oceania	Fiji	unknown	unknown	Wong et al, 2015
MDUST317	ERR357464	N/A	4.2.1	unknown	unknown	Knee aspirate	1982	Australia & Oceania	Oceania	Fiji	unknown	unknown	Wong et al, 2015
MDUST318	ERR357465	N/A	4.2	unknown	unknown	Unknown	1982	Australia & Oceania	Oceania	Fiji	unknown	unknown	Wong et al, 2015
MDUST320	ERR357466	N/A	4.2.1	unknown	unknown	Stool	1983	Australia & Oceania	Oceania	Fiji	unknown	unknown	Wong et al, 2015
MDUST323	ERR357467	N/A	4.2.1	unknown	unknown	Blood	1983	Australia & Oceania	Oceania	Fiji	unknown	unknown	Wong et al, 2015
MDUST325	ERR357468	N/A	3.5.4	unknown	unknown	Blood	1992	Australia & Oceania	Oceania	Samoa	unknown	unknown	Wong et al, 2015
MDUST327	ERR357469	N/A	4.3.1.1	unknown	unknown	Blood	1992	Australia & Oceania	Oceania	Fiji	unknown	unknown	Wong et al, 2015
MDUST329	ERR357470	N/A	2.1.7.1	unknown	unknown	Blood	1992	Australia & Oceania	Oceania	Papua New Guinea	unknown	unknown	Wong et al, 2015

MDUST332	ERR357471	N/A	2.1.7.2	unknown	unknown	Blood	1992	Australia & Oceania	Oceania	Papua New Guinea	unknown	unknown	Wong et al, 2015
MDUST333	ERR357472	N/A	2.1.7.1	unknown	unknown	Blood	1992	Australia & Oceania	Oceania	Papua New Guinea	unknown	unknown	Wong et al, 2015
MDUST334	ERR357473	N/A	3	unknown	unknown	Blood	1984	Australia & Oceania	Oceania	Fiji	unknown	unknown	Wong et al, 2015
MDUST340	ERR357474	N/A	4.3.1.1	unknown	unknown	Stool	1993	Australia & Oceania	Oceania	Fiji	unknown	unknown	Wong et al, 2015
MDUST341	ERR357475	N/A	4.3.1.1	unknown	unknown	Stool	1993	Australia & Oceania	Oceania	Fiji	unknown	unknown	Wong et al, 2015
MDUST342	ERR357476	N/A	4.3.1.1	unknown	unknown	Stool	1993	Australia & Oceania	Oceania	Fiji	unknown	unknown	Wong et al, 2015
MDUST344	ERR357477	N/A	4.2.1	unknown	unknown	Stool	1985	Australia & Oceania	Oceania	Fiji	unknown	unknown	Wong et al, 2015
MDUST345	ERR357478	N/A	2.1.7.2	unknown	unknown	Unknown	1985	Australia & Oceania	Oceania	Papua New Guinea	unknown	unknown	Wong et al, 2015
MDUST349	ERR357479	N/A	2.3.5	unknown	unknown	Stool	1985	Australia & Oceania	Oceania	Fiji	unknown	unknown	Wong et al, 2015
MDUST356	ERR357480	N/A	2.1.7.1	unknown	unknown	Blood	1994	Australia & Oceania	Oceania	Papua New Guinea	unknown	unknown	Wong et al, 2015
MDUST360	ERR357481	N/A	2.1.7.1	unknown	unknown	Stool	1998	Australia & Oceania	Oceania	Papua New Guinea	unknown	unknown	Wong et al, 2015
np4	ERR357576	N/A	4.3.1.2	unknown	unknown	Blood	2011	Asia	South Asia	Nepal	unknown	unknown	Wong et al, 2015
np41	ERR357577	N/A	3.3.2	unknown	unknown	Blood	2011	Asia	South Asia	Nepal	unknown	unknown	Wong et al, 2015
np94	ERR357578	N/A	4.3.1.2	unknown	unknown	Blood	2011	Asia	South Asia	Nepal	unknown	unknown	Wong et al, 2015
3671/3	ERR357579	N/A	2.5.1	unknown	unknown	Blood	2011	Africa	Central Africa	DRC	unknown	unknown	Wong et al, 2015
3332/3	ERR357580	N/A	2.5.1	unknown	unknown	Blood	2011	Africa	Central Africa	DRC	unknown	unknown	Wong et al, 2015
ERL113717	ERR357581	N/A	4.3.1	unknown	unknown	Blood	2011	Asia	South Asia	India	unknown	unknown	Wong et al, 2015
np5	ERR357583	N/A	4.3.1.2	unknown	unknown	Blood	2011	Asia	South Asia	Nepal	unknown	unknown	Wong et al, 2015
np43	ERR357584	N/A	4.3.1.2	unknown	unknown	Blood	2011	Asia	South Asia	Nepal	unknown	unknown	Wong et al, 2015
np95	ERR357585	N/A	4.3.1.2	unknown	unknown	Blood	2011	Asia	South Asia	Nepal	unknown	unknown	Wong et al, 2015
np8	ERR357587	N/A	4.3.1.2	unknown	unknown	Blood	2011	Asia	South Asia	Nepal	unknown	unknown	Wong et al, 2015
np44	ERR357588	N/A	4.3.1.2	unknown	unknown	Blood	2011	Asia	South Asia	Nepal	unknown	unknown	Wong et al, 2015
np75	ERR357589	N/A	4.3.1.2	unknown	unknown	Blood	2011	Asia	South Asia	Nepal	unknown	unknown	Wong et al, 2015
np97	ERR357590	N/A	4.3.1.2	unknown	unknown	Blood	2011	Asia	South Asia	Nepal	unknown	unknown	Wong et al, 2015
3632/3	ERR357591	N/A	2.5.1	unknown	unknown	Blood	2011	Africa	Central Africa	DRC	unknown	unknown	Wong et al, 2015
3139/3	ERR357592	N/A	2.5.1	unknown	unknown	Blood	2010	Africa	Central Africa	DRC	unknown	unknown	Wong et al, 2015
H12ESR01052-001A	ERR357593	N/A	3.5.4	unknown	unknown	Blood	2012	Australia & Oceania	Oceania	Samoa	unknown	unknown	Wong et al, 2015
np11	ERR357594	N/A	4.3.1.2	unknown	unknown	Blood	2011	Asia	South Asia	Nepal	unknown	unknown	Wong et al, 2015
np99	ERR357595	N/A	3.3.2	unknown	unknown	Blood	2011	Asia	South Asia	Nepal	unknown	unknown	Wong et al, 2015
3182/3	ERR357596	N/A	2.5.1	unknown	unknown	Blood	2010	Africa	Central Africa	DRC	unknown	unknown	Wong et al, 2015
H12ESR01946-001A	ERR357598	N/A	3.5.4	unknown	unknown	Unknown	2012	Australia & Oceania	Oceania	Samoa	unknown	unknown	Wong et al, 2015
dtc97	ERR357599	N/A	4.3.1.1	unknown	unknown	Blood	1994	Asia	Southeast Asia	Vietnam	unknown	unknown	Wong et al, 2015
np12	ERR357600	N/A	3.3.2	unknown	unknown	Blood	2011	Asia	South Asia	Nepal	unknown	unknown	Wong et al, 2015
np49	ERR357601	N/A	4.3.1.2	unknown	unknown	Blood	2011	Asia	South Asia	Nepal	unknown	unknown	Wong et al, 2015
np81	ERR357602	N/A	4.3.1.2	unknown	unknown	Blood	2011	Asia	South Asia	Nepal	unknown	unknown	Wong et al, 2015
np101	ERR357603	N/A	4.3.1.1	unknown	unknown	Blood	2011	Asia	South Asia	Nepal	unknown	unknown	Wong et al, 2015
3157/3	ERR357604	N/A	2.5.1	unknown	unknown	Blood	2010	Africa	Central Africa	DRC	unknown	unknown	Wong et al, 2015

H12ESR00394-001A	ERR357605	N/A	3.5.4	unknown	unknown	Blood	2012	Australia & Oceania	Oceania	Samoa	unknown	unknown	Wong et al, 2015
np13	ERR357606	N/A	4.3.1.2	unknown	unknown	Blood	2011	Asia	South Asia	Nepal	unknown	unknown	Wong et al, 2015
np50	ERR357607	N/A	3.2.2	unknown	unknown	Blood	2011	Asia	South Asia	Nepal	unknown	unknown	Wong et al, 2015
np83	ERR357608	N/A	4.3.1.2	unknown	unknown	Blood	2011	Asia	South Asia	Nepal	unknown	unknown	Wong et al, 2015
3653/3	ERR357610	N/A	2.5.1	unknown	unknown	Blood	2011	Africa	Central Africa	DRC	unknown	unknown	Wong et al, 2015
H12ESR04893-001A	ERR357611	N/A	3.5.4	unknown	unknown	Stool	2012	Australia & Oceania	Oceania	Samoa	unknown	unknown	Wong et al, 2015
np16	ERR357612	N/A	3.3.2	unknown	unknown	Blood	2011	Asia	South Asia	Nepal	unknown	unknown	Wong et al, 2015
np51	ERR357613	N/A	4.3.1.2	unknown	unknown	Blood	2011	Asia	South Asia	Nepal	unknown	unknown	Wong et al, 2015
np87	ERR357614	N/A	4.3.1.2	unknown	unknown	Blood	2011	Asia	South Asia	Nepal	unknown	unknown	Wong et al, 2015
ERL022368	ERR357615	N/A	4.3.1.1	unknown	unknown	Blood	2002	Asia	Western Asia	Western Asia	unknown	unknown	Wong et al, 2015
H12ESR04732-001A	ERR357617	N/A	3.5.4	unknown	unknown	Blood	2012	Australia & Oceania	Oceania	Samoa	unknown	unknown	Wong et al, 2015
dtc122	ERR357618	N/A	4.3.1.1	unknown	unknown	Blood	1995	Asia	Southeast Asia	Vietnam	unknown	unknown	Wong et al, 2015
np22	ERR357619	N/A	4.3.1.2	unknown	unknown	Blood	2011	Asia	South Asia	Nepal	unknown	unknown	Wong et al, 2015
np57	ERR357620	N/A	4.3.1.2	unknown	unknown	Blood	2011	Asia	South Asia	Nepal	unknown	unknown	Wong et al, 2015
np88	ERR357621	N/A	4.3.1.2	unknown	unknown	Blood	2011	Asia	South Asia	Nepal	unknown	unknown	Wong et al, 2015
3673/3	ERR357623	N/A	2.5.1	unknown	unknown	Blood	2011	Africa	Central Africa	DRC	unknown	unknown	Wong et al, 2015
H12ESR00753-001A	ERR357624	N/A	3.5.4	unknown	unknown	Stool	2012	Australia & Oceania	Oceania	Samoa	unknown	unknown	Wong et al, 2015
np60	ERR357625	N/A	4.3.1.2	unknown	unknown	Blood	2011	Asia	South Asia	Nepal	unknown	unknown	Wong et al, 2015
np89	ERR357626	N/A	4.3.1.2	unknown	unknown	Blood	2011	Asia	South Asia	Nepal	unknown	unknown	Wong et al, 2015
H12ESR04835-001A	ERR357627	N/A	3.5.4	unknown	unknown	Blood	2012	Australia & Oceania	Oceania	Samoa	unknown	unknown	Wong et al, 2015
np31	ERR357628	N/A	4.3.1.2	unknown	unknown	Blood	2011	Asia	South Asia	Nepal	unknown	unknown	Wong et al, 2015
np90	ERR357629	N/A	4.3.1.2	unknown	unknown	Blood	2011	Asia	South Asia	Nepal	unknown	unknown	Wong et al, 2015
ERL09896	ERR357630	N/A	4.3.1.2	unknown	unknown	Blood	2009	Asia	South Asia	India	unknown	unknown	Wong et al, 2015
H12ESR04928-001A	ERR357631	N/A	2.2.1	unknown	unknown	Stool	2012	Australia & Oceania	Oceania	Samoa	unknown	unknown	Wong et al, 2015
np39	ERR357632	N/A	4.3.1.2	unknown	unknown	Blood	2011	Asia	South Asia	Nepal	unknown	unknown	Wong et al, 2015
np65	ERR357633	N/A	4.3.1.2	unknown	unknown	Blood	2011	Asia	South Asia	Nepal	unknown	unknown	Wong et al, 2015
np92	ERR357634	N/A	4.3.1.2	unknown	unknown	Blood	2011	Asia	South Asia	Nepal	unknown	unknown	Wong et al, 2015
3143/3	ERR357635	N/A	2.5.1	unknown	unknown	Blood	2010	Africa	Central Africa	DRC	unknown	unknown	Wong et al, 2015
ERL102292	ERR357636	N/A	4.3.1	unknown	unknown	Blood	2010	Asia	South Asia	India	unknown	unknown	Wong et al, 2015
np40	ERR357637	N/A	4.3.1.2	unknown	unknown	Blood	2011	Asia	South Asia	Nepal	unknown	unknown	Wong et al, 2015
np67	ERR357638	N/A	4.3.1.2	unknown	unknown	Blood	2011	Asia	South Asia	Nepal	unknown	unknown	Wong et al, 2015
np93	ERR357639	N/A	4.3.1.2	unknown	unknown	Blood	2011	Asia	South Asia	Nepal	unknown	unknown	Wong et al, 2015
ERL101102	ERR357641	N/A	4.3.1.2	unknown	unknown	Stool	2010	Asia	South Asia	India	unknown	unknown	Wong et al, 2015
dtc99	ERR357642	N/A	4.3.1.1	unknown	unknown	Blood	1994	Asia	Southeast Asia	Vietnam	unknown	unknown	Wong et al, 2015
dtc98	ERR357643	N/A	4.3.1.1	unknown	unknown	Blood	1994	Asia	Southeast Asia	Vietnam	unknown	unknown	Wong et al, 2015
dn88	ERR357644	N/A	4.3.1.1	unknown	unknown	Blood	1995	Asia	Southeast Asia	Vietnam	unknown	unknown	Wong et al, 2015
dtc20	ERR357645	N/A	4.3.1.1	unknown	unknown	Blood	1994	Asia	Southeast Asia	Vietnam	unknown	unknown	Wong et al, 2015
dtc131	ERR357646	N/A	3.4	unknown	unknown	Blood	1995	Asia	Southeast Asia	Vietnam	unknown	unknown	Wong et al, 2015
dtc180	ERR357647	N/A	4.3.1.1	unknown	unknown	Blood	1995	Asia	Southeast Asia	Vietnam	unknown	unknown	Wong et al, 2015
dtc5	ERR357648	N/A	3.2.1	unknown	unknown	Blood	1994	Asia	Southeast Asia	Vietnam	unknown	unknown	Wong et al, 2015
dtc107	ERR357649	N/A	4.3.1.1	unknown	unknown	Blood	1994	Asia	Southeast Asia	Vietnam	unknown	unknown	Wong et al, 2015

ct1-13	ERR357651	N/A	4.3.1.1	unknown	unknown	Blood	1993	Asia	Southeast Asia	Vietnam	unknown	unknown	Wong et al, 2015
ct1-19	ERR357652	N/A	1.2.1	unknown	unknown	Blood	1993	Asia	Southeast Asia	Vietnam	unknown	unknown	Wong et al, 2015
dty1-99	ERR357653	N/A	4.3.1.1	unknown	unknown	Blood	1997	Asia	Southeast Asia	Vietnam	unknown	unknown	Wong et al, 2015
dty1-71	ERR357654	N/A	4.3.1.1	unknown	unknown	Blood	1997	Asia	Southeast Asia	Vietnam	unknown	unknown	Wong et al, 2015
ty2-121	ERR357655	N/A	4.3.1.1	unknown	unknown	Blood	1994	Asia	Southeast Asia	Vietnam	unknown	unknown	Wong et al, 2015
ty2-122	ERR357657	N/A	4.3.1.1	unknown	unknown	Blood	1994	Asia	Southeast Asia	Vietnam	unknown	unknown	Wong et al, 2015
ty1-48	ERR357659	N/A	4.3.1.1	unknown	unknown	Blood	1993	Asia	Southeast Asia	Vietnam	unknown	unknown	Wong et al, 2015
ty2-124	ERR357661	N/A	4.3.1.1	unknown	unknown	Blood	1994	Asia	Southeast Asia	Vietnam	unknown	unknown	Wong et al, 2015
ty2-68	ERR357663	N/A	3	unknown	unknown	Blood	1994	Asia	Southeast Asia	Vietnam	unknown	unknown	Wong et al, 2015
ty2-69	ERR357665	N/A	4.1	unknown	unknown	Blood	1994	Asia	Southeast Asia	Vietnam	unknown	unknown	Wong et al, 2015
ty1-30	ERR357667	N/A	3.2.1	unknown	unknown	Blood	1993	Asia	Southeast Asia	Vietnam	unknown	unknown	Wong et al, 2015
MDUST110	ERR357756	N/A	2.2.1	unknown	unknown	Blood	2007	Africa	North Africa	Egypt	unknown	unknown	Wong et al, 2015
MDUST112	ERR357757	N/A	3.3	unknown	unknown	Urine	2008	Africa	North Africa	Sudan	unknown	unknown	Wong et al, 2015
MDUST119	ERR357758	N/A	3.5.4	unknown	unknown	Blood	2009	Australia & Oceania	Oceania	Samoa	unknown	unknown	Wong et al, 2015
MDUST122	ERR357759	N/A	3.5.4	unknown	unknown	Urine	2010	Australia & Oceania	Oceania	Samoa	unknown	unknown	Wong et al, 2015
MDUST124	ERR357760	N/A	3.5.4	unknown	unknown	Blood	2011	Australia & Oceania	Oceania	Samoa	unknown	unknown	Wong et al, 2015
MDUST134	ERR357761	N/A	3	unknown	unknown	Stool	2011	Asia	Southeast Asia	Phillipines	unknown	unknown	Wong et al, 2015
MDUST137	ERR357762	N/A	3.3.1	unknown	unknown	Blood	2011	Asia	South Asia	India	unknown	unknown	Wong et al, 2015
MDUST142	ERR357763	N/A	4.3.1.1	unknown	unknown	Blood	2012	Asia	South Asia	India	unknown	unknown	Wong et al, 2015
MDUST144	ERR357764	N/A	4.3.1.1	unknown	unknown	Blood	2012	Australia & Oceania	Australia	Australia	unknown	unknown	Wong et al, 2015
MDUST146	ERR357765	N/A	4.3.1.2	unknown	unknown	Blood	2012	Unknown	Unknown	Unknown	unknown	unknown	Wong et al, 2015
MDUST148	ERR357766	N/A	4.3.1.2	unknown	unknown	Blood	2012	Asia	South Asia	India	unknown	unknown	Wong et al, 2015
MDUST165	ERR357767	N/A	2.2.2	unknown	unknown	Blood	2011	Asia	South Asia	India	unknown	unknown	Wong et al, 2015
MDUST167	ERR357768	N/A	4.3.1.2	unknown	unknown	Blood	2011	Asia	South Asia	India	unknown	unknown	Wong et al, 2015
MDUST170	ERR357769	N/A	4.3.1	unknown	unknown	Stool	2011	Asia	Southeast Asia	South-east Asia	unknown	unknown	Wong et al, 2015
MDUST171	ERR357770	N/A	3.3	unknown	unknown	Blood	2011	Asia	South Asia	India	unknown	unknown	Wong et al, 2015
MDUST172	ERR357771	N/A	4.3.1.1	unknown	unknown	Blood	2011	Asia	Southeast Asia	Thailand	unknown	unknown	Wong et al, 2015
MDUST176	ERR357772	N/A	3.3.2.Bd2	unknown	unknown	Blood	2011	Unknown	Unknown	Unknown	unknown	unknown	Wong et al, 2015
MDUST190	ERR357773	N/A	4.3.1.2	unknown	unknown	Blood	2011	Unknown	Unknown	Unknown	unknown	unknown	Wong et al, 2015
MDUST191	ERR357774	N/A	3.3.2.Bd1	unknown	unknown	Blood	2011	Asia	South Asia	Bangladesh	unknown	unknown	Wong et al, 2015
MDUST192	ERR357775	N/A	0.0.2	unknown	unknown	Blood	2011	Asia	Southeast Asia	Indonesia	unknown	unknown	Wong et al, 2015
MDUST193	ERR357776	N/A	4.3.1.1	unknown	unknown	Blood	2011	Asia	South Asia	Bangladesh	unknown	unknown	Wong et al, 2015
MDUST195	ERR357777	N/A	3.3	unknown	unknown	Blood	2011	Asia	South Asia	Bangladesh	unknown	unknown	Wong et al, 2015
MDUST218	ERR357778	N/A	2.2.2	unknown	unknown	Blood	2011	Asia	Western Asia	Lebanon	unknown	unknown	Wong et al, 2015
MDUST221	ERR357779	N/A	3.3	unknown	unknown	Blood	2011	Asia	South Asia	India	unknown	unknown	Wong et al, 2015
MDUST227	ERR357780	N/A	3.2.2	unknown	unknown	Blood	2010	Asia	South Asia	Bangladesh	unknown	unknown	Wong et al, 2015
MDUST228	ERR357781	N/A	3.5.4	unknown	unknown	Urine	2010	Australia & Oceania	Oceania	Samoa	unknown	unknown	Wong et al, 2015
MDUST230	ERR357782	N/A	4.3.1.1	unknown	unknown	Blood	2010	Asia	South Asia	Bangladesh	unknown	unknown	Wong et al, 2015
MDUST233	ERR357783	N/A	4.3.1.2	unknown	unknown	Blood	2010	Asia	South Asia	India	unknown	unknown	Wong et al, 2015
MDUST238	ERR357784	N/A	4.3.1.2	unknown	unknown	Blood	2011	Asia	South Asia	India	unknown	unknown	Wong et al, 2015
MDUST240	ERR357785	N/A	4.3.1.2	unknown	unknown	Blood	2011	Asia	South Asia	India	unknown	unknown	Wong et al, 2015
MDUST245	ERR357786	N/A	4.3.1.2	unknown	unknown	Blood	2011	Asia	South Asia	India	unknown	unknown	Wong et al, 2015

MDUST247	ERR357787	N/A	4.3.1.2	unknown	unknown	Stool	2011	Asia	South Asia	India	unknown	unknown	Wong et al, 2015
MDUST251	ERR357788	N/A	4.3.1.1	unknown	unknown	Blood	2010	Asia	South Asia	Pakistan	unknown	unknown	Wong et al, 2015
MDUST254	ERR357789	N/A	4.3.1.2	unknown	unknown	Blood	2010	Asia	South Asia	India	unknown	unknown	Wong et al, 2015
MDUST260	ERR357790	N/A	2.2.2	unknown	unknown	Blood	2011	Unknown	Unknown	Unknown	unknown	unknown	Wong et al, 2015
MDUST262	ERR357791	N/A	4.3.1.1	unknown	unknown	Blood	2011	Asia	South Asia	India	unknown	unknown	Wong et al, 2015
MDUST263	ERR357792	N/A	4.3.1.1	unknown	unknown	Blood	2011	Asia	South Asia	Bangladesh	unknown	unknown	Wong et al, 2015
MDUST264	ERR357793	N/A	4.3.1.1	unknown	unknown	Blood	2011	Asia	South Asia	India	unknown	unknown	Wong et al, 2015
MDUST266	ERR357794	N/A	4.3.1	unknown	unknown	Stool	2011	Asia	South Asia	Pakistan	unknown	unknown	Wong et al, 2015
MDUST271	ERR357795	N/A	4.3.1.1	unknown	unknown	Blood	2011	Asia	South Asia	India	unknown	unknown	Wong et al, 2015
MDUST272	ERR357796	N/A	4.3.1.1	unknown	unknown	Blood	2011	Asia	South Asia	India	unknown	unknown	Wong et al, 2015
MDUST273	ERR357797	N/A	4.3.1.2	unknown	unknown	Stool	2011	Asia	South Asia	India	unknown	unknown	Wong et al, 2015
MDUST275	ERR357798	N/A	2.1.7.1	unknown	unknown	Stool	1992	Australia & Oceania	Oceania	Papua New Guinea	unknown	unknown	Wong et al, 2015
MDUST290	ERR357799	N/A	3.5.4	unknown	unknown	Blood	2008	Australia & Oceania	Oceania	Samoa	unknown	unknown	Wong et al, 2015
MDUST302	ERR357800	N/A	4.1	unknown	unknown	Unknown	1980	Australia & Oceania	Oceania	Papua New Guinea	unknown	unknown	Wong et al, 2015
MDUST307	ERR357801	N/A	4.2.1	unknown	unknown	Unknown	1981	Australia & Oceania	Oceania	Fiji	unknown	unknown	Wong et al, 2015
MDUST314	ERR357802	N/A	4.1	unknown	unknown	Blood	1991	Australia & Oceania	Oceania	Vanuatu	unknown	unknown	Wong et al, 2015
MDUST324	ERR357803	N/A	4.3.1.1	unknown	unknown	Stool	1992	Australia & Oceania	Oceania	Fiji	unknown	unknown	Wong et al, 2015
MDUST338	ERR357804	N/A	2.1.7.1	unknown	unknown	Blood	1993	Australia & Oceania	Oceania	Papua New Guinea	unknown	unknown	Wong et al, 2015
MDUST343	ERR357805	N/A	2.3.5	unknown	unknown	Blood	1993	Australia & Oceania	Oceania	Fiji	unknown	unknown	Wong et al, 2015
MDUST346	ERR357806	N/A	4.2.1	unknown	unknown	Blood	1984	Australia & Oceania	Oceania	Fiji	unknown	unknown	Wong et al, 2015
MDUST347	ERR357807	N/A	4.3.1.1	unknown	unknown	Stool	1993	Australia & Oceania	Oceania	Fiji	unknown	unknown	Wong et al, 2015
MDUST350	ERR357808	N/A	2.3.5	unknown	unknown	Blood	1985	Australia & Oceania	Oceania	Fiji	unknown	unknown	Wong et al, 2015
MDUST353	ERR357809	N/A	4.1	unknown	unknown	Blood	1986	Australia & Oceania	Oceania	Papua New Guinea	unknown	unknown	Wong et al, 2015
MDUST357	ERR357810	N/A	2.1.7.2	unknown	unknown	Blood	1994	Australia & Oceania	Oceania	Papua New Guinea	unknown	unknown	Wong et al, 2015
MDUST359	ERR357811	N/A	2.1.7.1	unknown	unknown	Blood	1996	Australia & Oceania	Oceania	Papua New Guinea	unknown	unknown	Wong et al, 2015
MDUST367	ERR357813	N/A	4.3.1.2	unknown	unknown	Stool	2011	Asia	South Asia	India	unknown	unknown	Wong et al, 2015
MDUST369	ERR357814	N/A	4.3.1.2	unknown	unknown	Stool	2011	Asia	South Asia	India	unknown	unknown	Wong et al, 2015
MDUST370	ERR357815	N/A	4.3.1.1	unknown	unknown	Blood	2011	Asia	South Asia	India	unknown	unknown	Wong et al, 2015
MDUST371	ERR357816	N/A	3.5.4	unknown	unknown	Blood	2011	Australia & Oceania	Oceania	Samoa	unknown	unknown	Wong et al, 2015
MDUST372	ERR357817	N/A	4.3.1	unknown	unknown	Stool	2011	Asia	South Asia	Pakistan	unknown	unknown	Wong et al, 2015
MDUST373	ERR357818	N/A	2.5	unknown	unknown	Blood	2011	Asia	South Asia	India	unknown	unknown	Wong et al, 2015
MDUST374	ERR357819	N/A	4.3.1.1	unknown	unknown	Stool	2011	Asia	South Asia	Pakistan	unknown	unknown	Wong et al, 2015
MDUST375	ERR357820	N/A	4.3.1.1	unknown	unknown	Blood	2011	Asia	South Asia	India	unknown	unknown	Wong et al, 2015
MDUST376	ERR357821	N/A	4.3.1.1	unknown	unknown	Blood	2011	Asia	South Asia	India	unknown	unknown	Wong et al, 2015
MDUST377	ERR357822	N/A	4.3.1.2	unknown	unknown	Stool	2011	Asia	South Asia	India	unknown	unknown	Wong et al, 2015
MDUST378	ERR357823	N/A	3	unknown	unknown	Stool	2011	Asia	South Asia	India	unknown	unknown	Wong et al, 2015

MDUST379	ERR357824	N/A	4.3.1.1	unknown	unknown	Stool	2011	Asia	Southeast Asia	Myanmar	unknown	unknown	Wong et al, 2015
MDUST380	ERR357825	N/A	3.2.1	unknown	unknown	Blood	2011	Asia	South Asia	India	unknown	unknown	Wong et al, 2015
MDUST381	ERR357826	N/A	3.3.1	unknown	unknown	Stool	2011	Asia	South Asia	India	unknown	unknown	Wong et al, 2015
MDUST384	ERR357827	N/A	2.1	unknown	unknown	Stool	2011	Asia	Southeast Asia	Indonesia	unknown	unknown	Wong et al, 2015
MDUST390	ERR357828	N/A	4.3.1.3	unknown	unknown	Stool	2011	Asia	South Asia	Bangladesh	unknown	unknown	Wong et al, 2015
MDUST392	ERR357829	N/A	4.3.1	unknown	unknown	Unknown	2011	Asia	South Asia	India	unknown	unknown	Wong et al, 2015
MDUST394	ERR357830	N/A	4.3.1.1	unknown	unknown	Blood	2011	Asia	South Asia	India	unknown	unknown	Wong et al, 2015
MDUST395	ERR357831	N/A	2.1.6	unknown	unknown	Blood	2012	Asia	Southeast Asia	Indonesia	unknown	unknown	Wong et al, 2015
MDUST398	ERR357832	N/A	4.3.1.2	unknown	unknown	Blood	2011	Asia	Southeast Asia	Indonesia	unknown	unknown	Wong et al, 2015
MDUST399	ERR357833	N/A	4.3.1.1	unknown	unknown	Blood	2011	Asia	Southeast Asia	Vietnam	unknown	unknown	Wong et al, 2015
MDUST400	ERR357834	N/A	4.3.1.2	unknown	unknown	Blood	2012	Asia	South Asia	India	unknown	unknown	Wong et al, 2015
MDUST405	ERR357835	N/A	3.5.4	unknown	unknown	Blood	2012	Australia & Oceania	Oceania	Samoa	unknown	unknown	Wong et al, 2015
MDUST409	ERR357836	N/A	4.3.1.2	unknown	unknown	Blood	2011	Asia	South Asia	India	unknown	unknown	Wong et al, 2015
MDUST413	ERR357837	N/A	2.2.1	unknown	unknown	Blood	2011	Asia	South Asia	India	unknown	unknown	Wong et al, 2015
MDUST414	ERR357838	N/A	4.3.1.2	unknown	unknown	Blood	2011	Asia	South Asia	India	unknown	unknown	Wong et al, 2015
MDUST416	ERR357839	N/A	3.5.4	unknown	unknown	Blood	2012	Australia & Oceania	Oceania	Samoa	unknown	unknown	Wong et al, 2015
MDUST417	ERR357840	N/A	3.5.4	unknown	unknown	Blood	2012	Australia & Oceania	Oceania	Samoa	unknown	unknown	Wong et al, 2015
MDUST419	ERR357841	N/A	3.3.1	unknown	unknown	Stool	2011	Australia & Oceania	Oceania	Fiji	unknown	unknown	Wong et al, 2015
820	ERR360449	N/A	2.0.1	unknown	unknown	Blood	2003	Asia	South Asia	Pakistan	unknown	unknown	Wong et al, 2015
5504	ERR360450	N/A	2.0.1	unknown	unknown	Blood	2003	Asia	South Asia	Pakistan	unknown	unknown	Wong et al, 2015
3657	ERR360451	N/A	2.0.1	unknown	unknown	Blood	2003	Asia	South Asia	Pakistan	unknown	unknown	Wong et al, 2015
1093	ERR360452	N/A	4.3.1.1	unknown	unknown	Blood	2003	Asia	South Asia	Pakistan	unknown	unknown	Wong et al, 2015
17311	ERR360453	N/A	2	unknown	unknown	Blood	2003	Asia	South Asia	Pakistan	unknown	unknown	Wong et al, 2015
18763	ERR360454	N/A	3.3.1	unknown	unknown	Blood	2003	Asia	South Asia	Pakistan	unknown	unknown	Wong et al, 2015
3802	ERR360455	N/A	2	unknown	unknown	Blood	2003	Asia	South Asia	Pakistan	unknown	unknown	Wong et al, 2015
16637	ERR360456	N/A	2.0.1	unknown	unknown	Blood	2003	Asia	South Asia	Pakistan	unknown	unknown	Wong et al, 2015
3723	ERR360457	N/A	4.3.1	unknown	unknown	Blood	2003	Asia	South Asia	Pakistan	unknown	unknown	Wong et al, 2015
3484	ERR360458	N/A	4.3.1.1	unknown	unknown	Blood	2003	Asia	South Asia	Pakistan	unknown	unknown	Wong et al, 2015
16599	ERR360459	N/A	4.3.1	unknown	unknown	Blood	2003	Asia	South Asia	Pakistan	unknown	unknown	Wong et al, 2015
2439	ERR360460	N/A	2.0.1	unknown	unknown	Blood	2003	Asia	South Asia	Pakistan	unknown	unknown	Wong et al, 2015
1382	ERR360461	N/A	4.3.1.2	unknown	unknown	Blood	2003	Asia	South Asia	Pakistan	unknown	unknown	Wong et al, 2015
200	ERR360462	N/A	2	unknown	unknown	Blood	2003	Asia	South Asia	Pakistan	unknown	unknown	Wong et al, 2015
20753	ERR360463	N/A	3.2.2	unknown	unknown	Blood	2003	Asia	South Asia	Pakistan	unknown	unknown	Wong et al, 2015
3163	ERR360464	N/A	3.3.1	unknown	unknown	Blood	2003	Asia	South Asia	Pakistan	unknown	unknown	Wong et al, 2015
11193	ERR360465	N/A	3.3	unknown	unknown	Blood	2003	Asia	South Asia	Pakistan	unknown	unknown	Wong et al, 2015
6289	ERR360466	N/A	2	unknown	unknown	Blood	2003	Asia	South Asia	Pakistan	unknown	unknown	Wong et al, 2015
2064	ERR360467	N/A	2.0.1	unknown	unknown	Blood	2003	Asia	South Asia	Pakistan	unknown	unknown	Wong et al, 2015
1041	ERR360468	N/A	4.3.1.1	unknown	unknown	Blood	2003	Asia	South Asia	Pakistan	unknown	unknown	Wong et al, 2015
4180	ERR360469	N/A	2.3.3	unknown	unknown	Blood	2003	Asia	South Asia	Pakistan	unknown	unknown	Wong et al, 2015
21801	ERR360470	N/A	4.3.1.2	unknown	unknown	Blood	2003	Asia	South Asia	Pakistan	unknown	unknown	Wong et al, 2015
3283	ERR360471	N/A	4.3.1	unknown	unknown	Blood	2003	Asia	South Asia	Pakistan	unknown	unknown	Wong et al, 2015
10109	ERR360472	N/A	4.3.1	unknown	unknown	Blood	2003	Asia	South Asia	Pakistan	unknown	unknown	Wong et al, 2015

20911	ERR360473	N/A	3.2.2	unknown	unknown	Blood	2003	Asia	South Asia	Pakistan	unknown	unknown	Wong et al, 2015
21095	ERR360474	N/A	4.3.1	unknown	unknown	Blood	2003	Asia	South Asia	Pakistan	unknown	unknown	Wong et al, 2015
21669	ERR360475	N/A	3.2.2	unknown	unknown	Blood	2003	Asia	South Asia	Pakistan	unknown	unknown	Wong et al, 2015
2176	ERR360476	N/A	4.3.1.1	unknown	unknown	Blood	2003	Asia	South Asia	Pakistan	unknown	unknown	Wong et al, 2015
11110	ERR360477	N/A	3.2.2	unknown	unknown	Blood	2003	Asia	South Asia	Pakistan	unknown	unknown	Wong et al, 2015
2513	ERR360478	N/A	4.3.1	unknown	unknown	Blood	2003	Asia	South Asia	Pakistan	unknown	unknown	Wong et al, 2015
3164	ERR360479	N/A	2.3.3	unknown	unknown	Blood	2003	Asia	South Asia	Pakistan	unknown	unknown	Wong et al, 2015
42	ERR360480	N/A	4.3.1.1	unknown	unknown	Blood	2003	Asia	South Asia	Pakistan	unknown	unknown	Wong et al, 2015
3769	ERR360481	N/A	4.3.1	unknown	unknown	Blood	2003	Asia	South Asia	Pakistan	unknown	unknown	Wong et al, 2015
7415	ERR360482	N/A	3.0.1	unknown	unknown	Blood	2003	Asia	South Asia	Pakistan	unknown	unknown	Wong et al, 2015
E99-6646	ERR360483	N/A	3.1	unknown	unknown	Not provided	1999	Africa	North Africa	Algeria	unknown	unknown	Wong et al, 2015
Sep-18	ERR360484	N/A	0.1	unknown	unknown	Not provided	2009	Africa	North Africa	Algeria	unknown	unknown	Wong et al, 2015
77-303	ERR360485	N/A	2.5	unknown	unknown	Not provided	1977	Asia	South Asia	India	unknown	unknown	Wong et al, 2015
E98-8119	ERR360486	N/A	0.1.3	unknown	unknown	Not provided	1998	South America	South America	Peru	unknown	unknown	Wong et al, 2015
E99-8013	ERR360487	N/A	2.2	unknown	unknown	Not provided	1999	Africa	North Africa	Morocco	unknown	unknown	Wong et al, 2015
Jul-65	ERR360488	N/A	3.1.1	unknown	unknown	Not provided	2007	Africa	West Africa	IvoryCoast	unknown	unknown	Wong et al, 2015
E98-2107	ERR360489	N/A	2.3.1	unknown	unknown	Not provided	1998	Africa	West Africa	Senegal	unknown	unknown	Wong et al, 2015
E98-2601	ERR360490	N/A	3.3	unknown	unknown	Not provided	1998	Africa	West Africa	Gabon	unknown	unknown	Wong et al, 2015
E99-9794	ERR360491	N/A	3	unknown	unknown	Not provided	1999	Africa	East Africa	Comoros	unknown	unknown	Wong et al, 2015
E00-1382	ERR360492	N/A	2.0.2	unknown	unknown	Not provided	2000	Africa	North Africa	Algeria	unknown	unknown	Wong et al, 2015
E97-2364	ERR360493	N/A	2.5	unknown	unknown	Not provided	1997	Asia	South Asia	India	unknown	unknown	Wong et al, 2015
IPCU	ERR360494	N/A	2.3.1	unknown	unknown	Not provided	2007	Africa	Central Africa	Cameroon	unknown	unknown	Wong et al, 2015
Sep-21	ERR360495	N/A	3.1.1	unknown	unknown	Not provided	2009	Africa	West Africa	Guinea	unknown	unknown	Wong et al, 2015
E97-3246	ERR360496	N/A	2.5.2	unknown	unknown	Not provided	1997	Africa	East Africa	Madagascar	unknown	unknown	Wong et al, 2015
E02-2364	ERR360497	N/A	2.5	unknown	unknown	Not provided	2002	Africa	East Africa	Comoros	unknown	unknown	Wong et al, 2015
E98-4364	ERR360498	N/A	2.3.2	unknown	unknown	Not provided	1998	North America	North America	Mexico	unknown	unknown	Wong et al, 2015
E99-4879	ERR360499	N/A	2	unknown	unknown	Not provided	1999	Africa	North Africa	Morocco	unknown	unknown	Wong et al, 2015
Sep-84	ERR360500	N/A	3.1.1	unknown	unknown	Not provided	2009	Africa	Africa	Africa	unknown	unknown	Wong et al, 2015
Sep-14	ERR360501	N/A	3.1.1	unknown	unknown	Not provided	2009	Europe	Western Europe	France (mother-child, African name)	unknown	unknown	Wong et al, 2015
E00-6657	ERR360502	N/A	4.1	unknown	unknown	Not provided	2000	Africa	North Africa	Morocco	unknown	unknown	Wong et al, 2015
E98-11555	ERR360503	N/A	2	unknown	unknown	Not provided	1998	Asia	Western Asia	Armenia	unknown	unknown	Wong et al, 2015
E02-1963	ERR360504	N/A	4.3.1.1	unknown	unknown	Blood	2002	Asia	Southeast Asia	Laos	unknown	unknown	Wong et al, 2015
E01-1747	ERR360505	N/A	0.1.1	unknown	unknown	Not provided	2001	Africa	Central Africa	Cameroon	unknown	unknown	Wong et al, 2015
Sep-06	ERR360506	N/A	2.5.1	unknown	unknown	Not provided	2009	Africa	Central Africa	CAR	unknown	unknown	Wong et al, 2015
E99-6359	ERR360507	N/A	2.3.2	unknown	unknown	Not provided	1999	Africa	West Africa	Mali	unknown	unknown	Wong et al, 2015
80-2002	ERR360509	N/A	2.2	unknown	unknown	Not provided	1980	Africa	East Africa	Madagascar	unknown	unknown	Wong et al, 2015
E99-8635	ERR360510	N/A	4.3.1.3	unknown	unknown	Blood	1999	Asia	South Asia	Nepal	unknown	unknown	Wong et al, 2015
76-1292	ERR360511	N/A	1.1.3	unknown	unknown	Blood	1976	Africa	Central Africa	DRC	unknown	unknown	Wong et al, 2015
Sep-24	ERR360513	N/A	3.1.1	unknown	unknown	Not provided	2009	Africa	Central Africa	Cameroon	unknown	unknown	Wong et al, 2015
E96-12081	ERR360514	N/A	4.1	unknown	unknown	Not provided	1996	Africa	East Africa	Madagascar	unknown	unknown	Wong et al, 2015
E97-2307	ERR360515	N/A	3.3.2	unknown	unknown	Not provided	1997	Asia	South Asia	India	unknown	unknown	Wong et al, 2015
E99-1028	ERR360516	N/A	0.1	unknown	unknown	Not provided	1999	Africa	West Africa	Senegal	unknown	unknown	Wong et al, 2015
E02-0530	ERR360517	N/A	2.3.2	unknown	unknown	Not provided	2002	Africa	West Africa	Nigeria	unknown	unknown	Wong et al, 2015
E98-6926	ERR360518	N/A	4.1.1	unknown	unknown	Not provided	1998	Africa	West Africa	Mauritania	unknown	unknown	Wong et al, 2015

IPCC	ERR360519	N/A	2.3.1	unknown	unknown	Not provided	2002	Africa	Central Africa	Cameroon	unknown	unknown	Wong et al, 2015
IPCG	ERR360520	N/A	2.3.1	unknown	unknown	Not provided	2003	Africa	Central Africa	Cameroon	unknown	unknown	Wong et al, 2015
E01-8716	ERR360521	N/A	3	unknown	unknown	Not provided	2001	Asia	South Asia	Sri Lanka	unknown	unknown	Wong et al, 2015
E99-7012	ERR360522	N/A	3.1	unknown	unknown	Not provided	1999	Africa	North Africa	Morocco	unknown	unknown	Wong et al, 2015
Sep-32	ERR360523	N/A	3.1.1	unknown	unknown	Not provided	2009	Africa	West Africa	Mauritania	unknown	unknown	Wong et al, 2015
IPCO	ERR360524	N/A	2.3.1	unknown	unknown	Not provided	2006	Africa	Central Africa	Cameroon	unknown	unknown	Wong et al, 2015
Jun-73	ERR360525	N/A	3.1.1	unknown	unknown	Not provided	2006	Africa	West Africa	Ivory Coast	unknown	unknown	Wong et al, 2015
Jul-20	ERR360526	N/A	4.3.1.1	unknown	unknown	Not provided	2007	Asia	South Asia	Bangladesh	unknown	unknown	Wong et al, 2015
Jun-55	ERR360527	N/A	3.1.1	unknown	unknown	Not provided	2006	Africa	West Africa	Burkina Faso	unknown	unknown	Wong et al, 2015
E03-6418	ERR360528	N/A	4.3.1.3	unknown	unknown	Not provided	2003	Asia	South Asia	Bangladesh	unknown	unknown	Wong et al, 2015
Sep-13	ERR360529	N/A	3.1.1	unknown	unknown	Not provided	2009	Europe	Western Europe	France (mother-child, African name)	unknown	unknown	Wong et al, 2015
Sep-93	ERR360530	N/A	3.1.1	unknown	unknown	Not provided	2009	Africa	West Africa	Nigeria	unknown	unknown	Wong et al, 2015
E00-3459	ERR360614	N/A	2.2	unknown	unknown	Not provided	2000	Africa	East Africa	Madagascar	unknown	unknown	Wong et al, 2015
72-1258	ERR360615	N/A	2.3.2	unknown	unknown	Not provided	1972	North America	North America	Mexico	unknown	unknown	Wong et al, 2015
IPCK	ERR360616	N/A	2.3.1	unknown	unknown	Not provided	2004	Africa	Central Africa	Cameroon	unknown	unknown	Wong et al, 2015
E00-2388	ERR360617	N/A	2.5	unknown	unknown	Not provided	2000	Africa	North Africa	Egypt	unknown	unknown	Wong et al, 2015
E00-2756	ERR360618	N/A	2	unknown	unknown	Not provided	2000	Asia	South Asia	India	unknown	unknown	Wong et al, 2015
E00-3370	ERR360619	N/A	0.1	unknown	unknown	Not provided	2000	Africa	North Africa	Morocco	unknown	unknown	Wong et al, 2015
E02-0945	ERR360620	N/A	2.3.1	unknown	unknown	Not provided	2002	Africa	Central Africa	Cameroon	unknown	unknown	Wong et al, 2015
04-0339	ERR360621	N/A	3.1.1	unknown	unknown	Not provided	2004	Africa	West Africa	Togo	unknown	unknown	Wong et al, 2015
E99-6785	ERR360622	N/A	0.1	unknown	unknown	Not provided	1999	Africa	North Africa	Morocco	unknown	unknown	Wong et al, 2015
E02-2159	ERR360623	N/A	4.3.1.2	unknown	unknown	Not provided	2002	Asia	South Asia	SriLanka	unknown	unknown	Wong et al, 2015
E02-1687	ERR360624	N/A	2.3.2	unknown	unknown	Not provided	2002	Asia	Southeast Asia	Thailand	unknown	unknown	Wong et al, 2015
Sep-92	ERR360625	N/A	3.1.1	unknown	unknown	Not provided	2009	Africa	Africa	Africa	unknown	unknown	Wong et al, 2015
E99-9082	ERR360626	N/A	2.3.2	unknown	unknown	Not provided	1999	Africa	West Africa	Niger	unknown	unknown	Wong et al, 2015
May-83	ERR360627	N/A	1.1.1	unknown	unknown	Not provided	2005	Africa	North Africa	Algeria	unknown	unknown	Wong et al, 2015
Jul-08	ERR360628	N/A	2.3.1	unknown	unknown	Not provided	2007	Africa	Central Africa	Cameroon	unknown	unknown	Wong et al, 2015
69-61	ERR360629	N/A	3.1	unknown	unknown	Not provided	1961	Africa	North Africa	Tunisia	unknown	unknown	Wong et al, 2015
Apr-67	ERR360630	N/A	3.1.1	unknown	unknown	Not provided	2004	Africa	West Africa	Benin	unknown	unknown	Wong et al, 2015
E02-5919	ERR360631	N/A	3.1	unknown	unknown	Not provided	2002	Asia	East Asia	China	unknown	unknown	Wong et al, 2015
06-1510	ERR360632	N/A	3.1.1	unknown	unknown	Not provided	2006	Africa	West Africa	Burkina Faso	unknown	unknown	Wong et al, 2015
Jul-94	ERR360633	N/A	3.1.1	unknown	unknown	Not provided	2007	Africa	West Africa	Ivory Coast	unknown	unknown	Wong et al, 2015
14-58	ERR360634	N/A	2.3.1	unknown	unknown	Not provided	1958	Africa	Central Africa	Cameroon	unknown	unknown	Wong et al, 2015
E01-1811	ERR360635	N/A	2.2	unknown	unknown	Not provided	2001	Africa	West Africa	Mali	unknown	unknown	Wong et al, 2015
IPCE	ERR360636	N/A	2.3.1	unknown	unknown	Not provided	2002	Africa	Central Africa	Cameroon	unknown	unknown	Wong et al, 2015
E99-2862	ERR360637	N/A	2.2	unknown	unknown	Not provided	1999	Africa	North Africa	Egypt	unknown	unknown	Wong et al, 2015
E00-9345	ERR360639	N/A	4.3.1.2	unknown	unknown	Not provided	2000	Asia	South Asia	India	unknown	unknown	Wong et al, 2015
Apr-20	ERR360640	N/A	2.3.1	unknown	unknown	Not provided	2004	Africa	Central Africa	Cameroon	unknown	unknown	Wong et al, 2015
Sep-74	ERR360641	N/A	3.1.1	unknown	unknown	Not provided	2009	Africa	West Africa	Benin	unknown	unknown	Wong et al, 2015
76-1406	ERR360642	N/A	3.2.1	unknown	unknown	Blood	1976	Asia	Southeast Asia	Indonesia	unknown	unknown	Wong et al, 2015
E02-1739	ERR360643	N/A	3.1.1	unknown	unknown	Not provided	2002	Africa	West Africa	Benin	unknown	unknown	Wong et al, 2015
Dec-58	ERR360644	N/A	0.1.1	unknown	unknown	Not provided	1958	Africa	Central Africa	Cameroon	unknown	unknown	Wong et al, 2015
Sep-19	ERR360645	N/A	3.1.1	unknown	unknown	Not provided	2009	Europe	Western Europe	France (African meal)	unknown	unknown	Wong et al, 2015
May-83	ERR360646	N/A	2.5.1	unknown	unknown	Not provided	2005	Africa	Central Africa	Angola	unknown	unknown	Wong et al, 2015

IPCI	ERR360647	N/A	2.3.1	unknown	unknown	Not provided	2004	Africa	Central Africa	Cameroon	unknown	unknown	Wong et al, 2015
IPCL	ERR360648	N/A	2.3.1	unknown	unknown	Not provided	2004	Africa	Central Africa	Cameroon	unknown	unknown	Wong et al, 2015
72-1907	ERR360649	N/A	3.4	unknown	unknown	Not provided	1972	Asia	Southeast Asia	Vietnam	unknown	unknown	Wong et al, 2015
E00-6999	ERR360650	N/A	2.3.3	unknown	unknown	Not provided	2000	South America	South America	Peru	unknown	unknown	Wong et al, 2015
06-467	ERR360651	N/A	2.3.1	unknown	unknown	Not provided	2006	Africa	Central Africa	Cameroon	unknown	unknown	Wong et al, 2015
E02-0232	ERR360652	N/A	2.5	unknown	unknown	Not provided	2002	South America	South America	French Guiana	unknown	unknown	Wong et al, 2015
E97-9141	ERR360653	N/A	2.3.2	unknown	unknown	Not provided	1997	Asia	Western Asia	Turkey	unknown	unknown	Wong et al, 2015
E00-6599	ERR360654	N/A	3.3.1	unknown	unknown	Not provided	2000	Africa	West Africa	Cape Verde	unknown	unknown	Wong et al, 2015
IPCA	ERR360655	N/A	0.0.1	unknown	unknown	Not provided	2000	Africa	Central Africa	Cameroon	unknown	unknown	Wong et al, 2015
E00-7878	ERR360656	N/A	2	unknown	unknown	Not provided	2000	Africa	North Africa	Morocco	unknown	unknown	Wong et al, 2015
E98-8120	ERR360657	N/A	0.0.1	unknown	unknown	Not provided	1998	Africa	Central Africa	Cameroon	unknown	unknown	Wong et al, 2015
E00-7463	ERR360658	N/A	3.0.1	unknown	unknown	Not provided	2000	Africa	North Africa	Morocco	unknown	unknown	Wong et al, 2015
E99-6478	ERR360659	N/A	3.1	unknown	unknown	Not provided	1999	Africa	West Africa	Guinea	unknown	unknown	Wong et al, 2015
IPCR	ERR360660	N/A	2.3.1	unknown	unknown	Not provided	2006	Africa	Central Africa	Cameroon	unknown	unknown	Wong et al, 2015
73-1102	ERR360661	N/A	4.1	unknown	unknown	Not provided	1973	Asia	Southeast Asia	Vietnam	unknown	unknown	Wong et al, 2015
E00-5869	ERR360662	N/A	2.3.3	unknown	unknown	Not provided	2000	Asia	South Asia	Bangladesh	unknown	unknown	Wong et al, 2015
IPCT	ERR360663	N/A	2.3.1	unknown	unknown	Not provided	2007	Africa	Central Africa	Cameroon	unknown	unknown	Wong et al, 2015
E02-2612	ERR360664	N/A	2.2	unknown	unknown	Not provided	2002	Asia	South Asia	India	unknown	unknown	Wong et al, 2015
IPCQ	ERR360665	N/A	2.3.1	unknown	unknown	Not provided	2006	Africa	Central Africa	Cameroon	unknown	unknown	Wong et al, 2015
07-291	ERR360666	N/A	2.3.1	unknown	unknown	Not provided	2007	Africa	Central Africa	Cameroon	unknown	unknown	Wong et al, 2015
E99-5920	ERR360667	N/A	2.0.2	unknown	unknown	Not provided	1999	Africa	North Africa	Tunisia	unknown	unknown	Wong et al, 2015
E99-8067	ERR360668	N/A	0.1.2	unknown	unknown	Not provided	1999	Africa	North Africa	Algeria	unknown	unknown	Wong et al, 2015
07-702	ERR360669	N/A	2.3.1	unknown	unknown	Not provided	2007	Africa	Central Africa	Cameroon	unknown	unknown	Wong et al, 2015
08-10571	ERR360670	N/A	3.1.1	unknown	unknown	Not provided	2008	Africa	West Africa	Mali	unknown	unknown	Wong et al, 2015
Apr-45	ERR360671	N/A	3.1.1	unknown	unknown	Not provided	2004	Africa	West Africa	Benin	unknown	unknown	Wong et al, 2015
E00-3201	ERR360672	N/A	2.3.2	unknown	unknown	Not provided	2000	Africa	West Africa	Mali	unknown	unknown	Wong et al, 2015
E01-0407	ERR360673	N/A	4.1	unknown	unknown	Not provided	2001	Asia	Western Asia	Turkey	unknown	unknown	Wong et al, 2015
IPCP	ERR360674	N/A	2.3.1	unknown	unknown	Not provided	2006	Africa	Central Africa	Cameroon	unknown	unknown	Wong et al, 2015
06-1513	ERR360675	N/A	3.1.1	unknown	unknown	Not provided	2006	Africa	West Africa	Burkina Faso	unknown	unknown	Wong et al, 2015
Sep-77	ERR360676	N/A	0.1	unknown	unknown	Not provided	2009	Africa	North Africa	Algeria	unknown	unknown	Wong et al, 2015
Aug-14	ERR360677	N/A	2.5.1	unknown	unknown	Blood	2008	Africa	Central Africa	DRC	unknown	unknown	Wong et al, 2015
07-977	ERR360678	N/A	2.5.1	unknown	unknown	Not provided	2007	Africa	Central Africa	CAR	unknown	unknown	Wong et al, 2015
Jun-29	ERR360679	N/A	2.3.1	unknown	unknown	Not provided	2006	Africa	Central Africa	Cameroon	unknown	unknown	Wong et al, 2015
E02-1536	ERR360680	N/A	2.5	unknown	unknown	Not provided	2002	South America	South America	French Guiana	unknown	unknown	Wong et al, 2015
E02-0937	ERR360681	N/A	2.5	unknown	unknown	Not provided	2002	Africa	East Africa	Comoros	unknown	unknown	Wong et al, 2015
Jun-90	ERR360682	N/A	3.1.1	unknown	unknown	Not provided	2006	Africa	West Africa	Togo	unknown	unknown	Wong et al, 2015
Q0904130	ERR360685	N/A	2.5.1	unknown	unknown	Not provided	2006	Africa	Central Africa	CAR	unknown	unknown	Wong et al, 2015
Aug-27	ERR360686	N/A	3.1.1	unknown	unknown	Not provided	2008	Africa	West Africa	Ivory Coast	unknown	unknown	Wong et al, 2015
Sep-97	ERR360687	N/A	3.1.1	unknown	unknown	Not provided	2009	Europe	Western Europe	France (African meal)	unknown	unknown	Wong et al, 2015
E99-8095	ERR360688	N/A	4.1	unknown	unknown	Not provided	1999	Africa	North Africa	Algeria	unknown	unknown	Wong et al, 2015
IPCS	ERR360689	N/A	4.1.1	unknown	unknown	Not provided	2006	Africa	Central Africa	Cameroon	unknown	unknown	Wong et al, 2015
IPCN	ERR360690	N/A	3.1.1	unknown	unknown	Not provided	2005	Africa	Central Africa	Cameroon	unknown	unknown	Wong et al, 2015
E01-7006	ERR360691	N/A	4.1	unknown	unknown	Not provided	2001	Asia	Western Asia	Lebanon	unknown	unknown	Wong et al, 2015
E01-7101	ERR360692	N/A	2.3.2	unknown	unknown	Not provided	2001	Africa	West Africa	Togo	unknown	unknown	Wong et al, 2015
Aug-02	ERR360693	N/A	4.3.1.1	unknown	unknown	Not provided	2008	Asia	Western Asia	Palestine	unknown	unknown	Wong et al, 2015

E00-7666	ERR360694	N/A	3.1	unknown	unknown	Not provided	2000	Africa	North Africa	Tunisia	unknown	unknown	Wong et al, 2015
E01-5741	ERR360695	N/A	1.1.3	unknown	unknown	Not provided	2001	Africa	Central Africa	Angola	unknown	unknown	Wong et al, 2015
E00-6172	ERR360696	N/A	3.5	unknown	unknown	Blood	2000	Asia	Southeast Asia	Indonesia	unknown	unknown	Wong et al, 2015
2003-013044	ERR360738	N/A	4.3.1.1	unknown	unknown	Blood	2008	Asia	Southeast Asia	Cambodia	unknown	unknown	Wong et al, 2015
2008-006720	ERR360739	N/A	4.3.1.1	unknown	unknown	Blood	2008	Asia	Southeast Asia	Cambodia	unknown	unknown	Wong et al, 2015
2008-007824	ERR360740	N/A	4.3.1.1	unknown	unknown	Blood	2008	Asia	Southeast Asia	Cambodia	unknown	unknown	Wong et al, 2015
2008-010439	ERR360741	N/A	4.3.1.1	unknown	unknown	Blood	2008	Asia	Southeast Asia	Cambodia	unknown	unknown	Wong et al, 2015
2008-011433	ERR360742	N/A	4.3.1.1	unknown	unknown	Blood	2008	Asia	Southeast Asia	Cambodia	unknown	unknown	Wong et al, 2015
2005-005326	ERR360743	N/A	4.3.1.1	unknown	unknown	Blood	2008	Asia	Southeast Asia	Cambodia	unknown	unknown	Wong et al, 2015
2009-019983	ERR360744	N/A	4.3.1.1	unknown	unknown	Blood	2009	Asia	Southeast Asia	Cambodia	unknown	unknown	Wong et al, 2015
2009-020666	ERR360745	N/A	4.3.1.1	unknown	unknown	Thigh pus	2009	Asia	Southeast Asia	Cambodia	unknown	unknown	Wong et al, 2015
2003-011175	ERR360746	N/A	4.3.1.1	unknown	unknown	Blood	2009	Asia	Southeast Asia	Cambodia	unknown	unknown	Wong et al, 2015
2010-002168	ERR360747	N/A	3.4	unknown	unknown	Blood	2010	Asia	Southeast Asia	Cambodia	unknown	unknown	Wong et al, 2015
2010-012339	ERR360748	N/A	4.3.1.1	unknown	unknown	Blood	2010	Asia	Southeast Asia	Cambodia	unknown	unknown	Wong et al, 2015
2009-008387	ERR360754	N/A	4.3.1.1	unknown	unknown	Pleural fluid	2010	Asia	Southeast Asia	Cambodia	unknown	unknown	Wong et al, 2015
2011-002088	ERR360755	N/A	4.3.1.1	unknown	unknown	Blood	2011	Asia	Southeast Asia	Cambodia	unknown	unknown	Wong et al, 2015
01-2010-000934	ERR360756	N/A	4.3.1.1	unknown	unknown	Blood	2011	Asia	Southeast Asia	Cambodia	unknown	unknown	Wong et al, 2015
2011-007188	ERR360758	N/A	4.3.1.1	unknown	unknown	Gallbladder fluid	2011	Asia	Southeast Asia	Cambodia	unknown	unknown	Wong et al, 2015
2002-214318	ERR360759	N/A	4.3.1.1	unknown	unknown	Blood	2011	Asia	Southeast Asia	Cambodia	unknown	unknown	Wong et al, 2015
2011-008491	ERR360760	N/A	4.3.1.1	unknown	unknown	Blood	2011	Asia	Southeast Asia	Cambodia	unknown	unknown	Wong et al, 2015
2010-006459	ERR360761	N/A	4.3.1.1	unknown	unknown	Blood	2011	Asia	Southeast Asia	Cambodia	unknown	unknown	Wong et al, 2015
2002-205957	ERR360762	N/A	4.3.1.1	unknown	unknown	Blood	2011	Asia	Southeast Asia	Cambodia	unknown	unknown	Wong et al, 2015
2007-017523	ERR360763	N/A	4.3.1.1	unknown	unknown	Blood	2011	Asia	Southeast Asia	Cambodia	unknown	unknown	Wong et al, 2015
2011-009404	ERR360765	N/A	4.3.1.1	unknown	unknown	Blood	2011	Asia	Southeast Asia	Cambodia	unknown	unknown	Wong et al, 2015
2009-004677	ERR360766	N/A	4.3.1.1	unknown	unknown	Blood	2011	Asia	Southeast Asia	Cambodia	unknown	unknown	Wong et al, 2015
2011-015914	ERR360769	N/A	4.3.1.1	unknown	unknown	Blood	2011	Asia	Southeast Asia	Cambodia	unknown	unknown	Wong et al, 2015
2011-017747	ERR360770	N/A	4.3.1.1	unknown	unknown	Blood	2011	Asia	Southeast Asia	Cambodia	unknown	unknown	Wong et al, 2015
2011-017563	ERR360771	N/A	4.3.1.1	unknown	unknown	Blood	2011	Asia	Southeast Asia	Cambodia	unknown	unknown	Wong et al, 2015
2011-019378	ERR360772	N/A	4.3.1.1	unknown	unknown	Blood	2011	Asia	Southeast Asia	Cambodia	unknown	unknown	Wong et al, 2015
2011-019409	ERR360773	N/A	4.3.1.1	unknown	unknown	Hip joint pus	2011	Asia	Southeast Asia	Cambodia	unknown	unknown	Wong et al, 2015
01-2011-005712	ERR360774	N/A	4.3.1.1	unknown	unknown	Blood	2011	Asia	Southeast Asia	Cambodia	unknown	unknown	Wong et al, 2015
2012-010218	ERR360775	N/A	4.3.1.1	unknown	unknown	Blood	2012	Asia	Southeast Asia	Cambodia	unknown	unknown	Wong et al, 2015
2012-011302	ERR360776	N/A	4.3.1.1	unknown	unknown	Stool	2012	Asia	Southeast Asia	Cambodia	unknown	unknown	Wong et al, 2015
2012-011333	ERR360777	N/A	4.3.1.1	unknown	unknown	Blood	2012	Asia	Southeast Asia	Cambodia	unknown	unknown	Wong et al, 2015
2010-011024	ERR360778	N/A	4.3.1.1	unknown	unknown	Blood	2012	Asia	Southeast Asia	Cambodia	unknown	unknown	Wong et al, 2015
2011-019645	ERR360779	N/A	4.3.1.1	unknown	unknown	Blood	2012	Asia	Southeast Asia	Cambodia	unknown	unknown	Wong et al, 2015
2009-019909	ERR360780	N/A	4.3.1.1	unknown	unknown	Blood	2012	Asia	Southeast Asia	Cambodia	unknown	unknown	Wong et al, 2015
2012-011344	ERR360781	N/A	4.3.1.1	unknown	unknown	Blood	2012	Asia	Southeast Asia	Cambodia	unknown	unknown	Wong et al, 2015
2008-011089	ERR360782	N/A	4.3.1.1	unknown	unknown	Blood	2012	Asia	Southeast Asia	Cambodia	unknown	unknown	Wong et al, 2015
2012-010551	ERR360783	N/A	4.3.1.1	unknown	unknown	Blood	2012	Asia	Southeast Asia	Cambodia	unknown	unknown	Wong et al, 2015
2012-010616	ERR360785	N/A	4.3.1.1	unknown	unknown	Blood	2012	Asia	Southeast Asia	Cambodia	unknown	unknown	Wong et al, 2015
01-2010-000073	ERR360786	N/A	4.3.1.1	unknown	unknown	Blood	2012	Asia	Southeast Asia	Cambodia	unknown	unknown	Wong et al, 2015
2012-011809	ERR360787	N/A	4.3.1.1	unknown	unknown	Blood	2012	Asia	Southeast Asia	Cambodia	unknown	unknown	Wong et al, 2015
2012-010204	ERR360788	N/A	4.3.1.1	unknown	unknown	Blood	2012	Asia	Southeast Asia	Cambodia	unknown	unknown	Wong et al, 2015
2005-012355	ERR360789	N/A	4.3.1.1	unknown	unknown	Blood	2012	Asia	Southeast Asia	Cambodia	unknown	unknown	Wong et al, 2015

2012-015100	ERR360791	N/A	4.3.1.1	unknown	unknown	Blood	2012	Asia	Southeast Asia	Cambodia	unknown	unknown	Wong et al, 2015
2012-017695	ERR360792	N/A	4.3.1.1	unknown	unknown	Blood	2012	Asia	Southeast Asia	Cambodia	unknown	unknown	Wong et al, 2015
1035759	ERR360793	N/A	4.3.1.1.EA1	unknown	unknown	Blood	2012	Africa	Southern Africa	Malawi	unknown	unknown	Wong et al, 2015
BKQ4CF	ERR360794	N/A	4.3.1.1.EA1	unknown	unknown	Blood	2013	Africa	Southern Africa	Malawi	unknown	unknown	Wong et al, 2015
A57501	ERR360795	N/A	4.1.1	unknown	unknown	Blood	2010	Africa	Southern Africa	Malawi	unknown	unknown	Wong et al, 2015
A58372	ERR360796	N/A	4.1.1	unknown	unknown	Blood	2010	Africa	Southern Africa	Malawi	unknown	unknown	Wong et al, 2015
1005624	ERR360797	N/A	4.3.1.1.EA1	unknown	unknown	Blood	2010	Africa	Southern Africa	Malawi	unknown	unknown	Wong et al, 2015
1003108	ERR360798	N/A	4.3.1.1.EA1	unknown	unknown	Blood	2010	Africa	Southern Africa	Malawi	unknown	unknown	Wong et al, 2015
BKQ22S	ERR360799	N/A	4.3.1.1.EA1	unknown	unknown	Blood	2012	Africa	Southern Africa	Malawi	unknown	unknown	Wong et al, 2015
1004971	ERR360800	N/A	2.4.1	unknown	unknown	Blood	2010	Africa	Southern Africa	Malawi	unknown	unknown	Wong et al, 2015
1006099	ERR360801	N/A	4.3.1.1.EA1	unknown	unknown	Blood	2010	Africa	Southern Africa	Malawi	unknown	unknown	Wong et al, 2015
1003117	ERR360802	N/A	4.3.1.1.EA1	unknown	unknown	Blood	2010	Africa	Southern Africa	Malawi	unknown	unknown	Wong et al, 2015
BKQ1GA	ERR360803	N/A	4.1.1	unknown	unknown	Blood	2012	Africa	Southern Africa	Malawi	unknown	unknown	Wong et al, 2015
1007000	ERR360804	N/A	2.2.2	unknown	unknown	Blood	2010	Africa	Southern Africa	Malawi	unknown	unknown	Wong et al, 2015
BHA1P5	ERR360805	N/A	4.3.1.1.EA1	unknown	unknown	Blood	2012	Africa	Southern Africa	Malawi	unknown	unknown	Wong et al, 2015
BKQ2KZ	ERR360806	N/A	4.3.1.1.EA1	unknown	unknown	Blood	2012	Africa	Southern Africa	Malawi	unknown	unknown	Wong et al, 2015
BKQ4SJ	ERR360807	N/A	4.3.1.1.EA1	unknown	unknown	Blood	2013	Africa	Southern Africa	Malawi	unknown	unknown	Wong et al, 2015
BKQ4NZ	ERR360809	N/A	4.3.1.1.EA1	unknown	unknown	Blood	2013	Africa	Southern Africa	Malawi	unknown	unknown	Wong et al, 2015
BKQ2H5	ERR360810	N/A	4.3.1.1.EA1	unknown	unknown	Blood	2012	Africa	Southern Africa	Malawi	unknown	unknown	Wong et al, 2015
BHA2HH	ERR360811	N/A	4.3.1.1.EA1	unknown	unknown	Blood	2012	Africa	Southern Africa	Malawi	unknown	unknown	Wong et al, 2015
1003078	ERR360812	N/A	4.3.1.1	unknown	unknown	Blood	2010	Africa	Southern Africa	Malawi	unknown	unknown	Wong et al, 2015
D56675	ERR360813	N/A	2.4.1	unknown	unknown	Blood	2010	Africa	Southern Africa	Malawi	unknown	unknown	Wong et al, 2015
A59204	ERR360814	N/A	4.1.1	unknown	unknown	Blood	2010	Africa	Southern Africa	Malawi	unknown	unknown	Wong et al, 2015
BHA2QL	ERR360815	N/A	4.3.1.1.EA1	unknown	unknown	Blood	2013	Africa	Southern Africa	Malawi	unknown	unknown	Wong et al, 2015
BKQ4H1	ERR360816	N/A	4.3.1.1.EA1	unknown	unknown	Blood	2013	Africa	Southern Africa	Malawi	unknown	unknown	Wong et al, 2015
1038377	ERR360817	N/A	4.3.1.1.EA1	unknown	unknown	Blood	2012	Africa	Southern Africa	Malawi	unknown	unknown	Wong et al, 2015
1037921	ERR360818	N/A	4.3.1.1.EA1	unknown	unknown	Blood	2012	Africa	Southern Africa	Malawi	unknown	unknown	Wong et al, 2015
BKQ4E3	ERR360819	N/A	4.3.1.1.EA1	unknown	unknown	Blood	2013	Africa	Southern Africa	Malawi	unknown	unknown	Wong et al, 2015
D55055	ERR360820	N/A	2.2	unknown	unknown	Blood	2010	Africa	Southern Africa	Malawi	unknown	unknown	Wong et al, 2015
D56393	ERR360821	N/A	4.1.1	unknown	unknown	Blood	2010	Africa	Southern Africa	Malawi	unknown	unknown	Wong et al, 2015
A58452	ERR360822	N/A	3.3.1	unknown	unknown	Blood	2010	Africa	Southern Africa	Malawi	unknown	unknown	Wong et al, 2015
A59217	ERR360824	N/A	4.3.1.2	unknown	unknown	Blood	2010	Africa	Southern Africa	Malawi	unknown	unknown	Wong et al, 2015
A58450	ERR360825	N/A	4.1.1	unknown	unknown	Blood	2010	Africa	Southern Africa	Malawi	unknown	unknown	Wong et al, 2015
D54782	ERR360826	N/A	2.4.1	unknown	unknown	Blood	2010	Africa	Southern Africa	Malawi	unknown	unknown	Wong et al, 2015
BKQ4JU	ERR360827	N/A	4.3.1.1.EA1	unknown	unknown	Blood	2013	Africa	Southern Africa	Malawi	unknown	unknown	Wong et al, 2015
1036491	ERR360828	N/A	4.3.1.1.EA1	unknown	unknown	Blood	2012	Africa	Southern Africa	Malawi	unknown	unknown	Wong et al, 2015
1036143	ERR360829	N/A	4.3.1.1.EA1	unknown	unknown	Blood	2012	Africa	Southern Africa	Malawi	unknown	unknown	Wong et al, 2015
BKQ4D9	ERR360830	N/A	4.3.1.1.EA1	unknown	unknown	Blood	2013	Africa	Southern Africa	Malawi	unknown	unknown	Wong et al, 2015
A58390	ERR360832	N/A	4.3.1.2	unknown	unknown	Blood	2010	Africa	Southern Africa	Malawi	unknown	unknown	Wong et al, 2015
A59307	ERR360833	N/A	4.1.1	unknown	unknown	Blood	2010	Africa	Southern Africa	Malawi	unknown	unknown	Wong et al, 2015
2010-013700	ERR360845	N/A	4.3.1.1	unknown	unknown	Blood	2010	Asia	Southeast Asia	Cambodia	unknown	unknown	Wong et al, 2015
2004-007692	ERR360846	N/A	4.3.1.1	unknown	unknown	Blood	2010	Asia	Southeast Asia	Cambodia	unknown	unknown	Wong et al, 2015
2008-000234	ERR360847	N/A	4.3.1.1	unknown	unknown	Blood	2010	Asia	Southeast Asia	Cambodia	unknown	unknown	Wong et al, 2015
2009-017391	ERR360848	N/A	4.3.1.1	unknown	unknown	Blood	2010	Asia	Southeast Asia	Cambodia	unknown	unknown	Wong et al, 2015
2008-003275	ERR360889	N/A	4.3.1.1	unknown	unknown	Blood	2008	Asia	Southeast Asia	Cambodia	unknown	unknown	Wong et al, 2015

2007-025892	ERR360890	N/A	4.3.1.1	unknown	unknown	Blood	2008	Asia	Southeast Asia	Cambodia	unknown	unknown	Wong et al, 2015
2008-007203	ERR360891	N/A	4.3.1.1	unknown	unknown	Blood	2008	Asia	Southeast Asia	Cambodia	unknown	unknown	Wong et al, 2015
2008-007991	ERR360948	N/A	4.3.1.1	unknown	unknown	Blood	2008	Asia	Southeast Asia	Cambodia	unknown	unknown	Wong et al, 2015
2007-016977	ERR360949	N/A	4.3.1.1	unknown	unknown	Blood	2008	Asia	Southeast Asia	Cambodia	unknown	unknown	Wong et al, 2015
2008-012004	ERR360950	N/A	4.3.1.1	unknown	unknown	Blood	2008	Asia	Southeast Asia	Cambodia	unknown	unknown	Wong et al, 2015
2010-012486	ERR360952	N/A	4.3.1.1	unknown	unknown	Blood	2010	Asia	Southeast Asia	Cambodia	unknown	unknown	Wong et al, 2015
2010-015926	ERR360954	N/A	4.3.1.1	unknown	unknown	Blood	2010	Asia	Southeast Asia	Cambodia	unknown	unknown	Wong et al, 2015
2010-018118	ERR360955	N/A	4.3.1.1	unknown	unknown	Blood	2010	Asia	Southeast Asia	Cambodia	unknown	unknown	Wong et al, 2015
2010-018908	ERR360956	N/A	4.3.1.1	unknown	unknown	Blood	2010	Asia	Southeast Asia	Cambodia	unknown	unknown	Wong et al, 2015
01-2010-005821	ERR360958	N/A	4.3.1.1	unknown	unknown	Blood	2010	Asia	Southeast Asia	Cambodia	unknown	unknown	Wong et al, 2015
2010-020905	ERR360959	N/A	4.3.1.1	unknown	unknown	Blood	2010	Asia	Southeast Asia	Cambodia	unknown	unknown	Wong et al, 2015
2006-024861	ERR360960	N/A	4.3.1.1	unknown	unknown	Blood	2010	Asia	Southeast Asia	Cambodia	unknown	unknown	Wong et al, 2015
01-2011-000664	ERR360961	N/A	4.3.1.1	unknown	unknown	Blood	2011	Asia	Southeast Asia	Cambodia	unknown	unknown	Wong et al, 2015
2011-024146	ERR360962	N/A	4.3.1.1	unknown	unknown	Blood	2011	Asia	Southeast Asia	Cambodia	unknown	unknown	Wong et al, 2015
2012-010535	ERR360963	N/A	4.3.1.1	unknown	unknown	Blood	2012	Asia	Southeast Asia	Cambodia	unknown	unknown	Wong et al, 2015
2012-010742	ERR360964	N/A	4.3.1.1	unknown	unknown	Blood	2012	Asia	Southeast Asia	Cambodia	unknown	unknown	Wong et al, 2015
2012-009188	ERR360965	N/A	4.3.1.1	unknown	unknown	Blood	2012	Asia	Southeast Asia	Cambodia	unknown	unknown	Wong et al, 2015
2009-018790	ERR360966	N/A	4.3.1.1	unknown	unknown	Blood	2012	Asia	Southeast Asia	Cambodia	unknown	unknown	Wong et al, 2015
01-2012-002891	ERR360967	N/A	4.3.1.1	unknown	unknown	Blood	2012	Asia	Southeast Asia	Cambodia	unknown	unknown	Wong et al, 2015
2008-020523	ERR360968	N/A	4.3.1.1	unknown	unknown	Blood	2012	Asia	Southeast Asia	Cambodia	unknown	unknown	Wong et al, 2015
2003-000735	ERR360970	N/A	4.3.1.1	unknown	unknown	Blood	2012	Asia	Southeast Asia	Cambodia	unknown	unknown	Wong et al, 2015
2012-011897	ERR360971	N/A	4.3.1.1	unknown	unknown	Blood	2012	Asia	Southeast Asia	Cambodia	unknown	unknown	Wong et al, 2015
2012-011885	ERR360972	N/A	4.3.1.1	unknown	unknown	Blood	2012	Asia	Southeast Asia	Cambodia	unknown	unknown	Wong et al, 2015
2012-011284	ERR360973	N/A	4.3.1.1	unknown	unknown	Blood	2012	Asia	Southeast Asia	Cambodia	unknown	unknown	Wong et al, 2015
01-2012-003910	ERR360975	N/A	4.3.1.1	unknown	unknown	Stool	2012	Asia	Southeast Asia	Cambodia	unknown	unknown	Wong et al, 2015
2012-004687	ERR360976	N/A	4.3.1.1	unknown	unknown	Blood	2012	Asia	Southeast Asia	Cambodia	unknown	unknown	Wong et al, 2015
2012-016786	ERR360977	N/A	4.3.1.1	unknown	unknown	Blood	2012	Asia	Southeast Asia	Cambodia	unknown	unknown	Wong et al, 2015
01-2010-001056	ERR360978	N/A	4.3.1.1	unknown	unknown	Blood	2012	Asia	Southeast Asia	Cambodia	unknown	unknown	Wong et al, 2015
2012-010759	ERR360979	N/A	4.3.1.1	unknown	unknown	Blood	2012	Asia	Southeast Asia	Cambodia	unknown	unknown	Wong et al, 2015
2012-000742	ERR360980	N/A	4.3.1.1	unknown	unknown	Blood	2012	Asia	Southeast Asia	Cambodia	unknown	unknown	Wong et al, 2015
2012-010559	ERR360981	N/A	4.3.1.1	unknown	unknown	Blood	2012	Asia	Southeast Asia	Cambodia	unknown	unknown	Wong et al, 2015
01-2012-002876	ERR360982	N/A	4.3.1.1	unknown	unknown	Blood	2012	Asia	Southeast Asia	Cambodia	unknown	unknown	Wong et al, 2015
2009-012656	ERR360983	N/A	4.3.1.1	unknown	unknown	Blood	2012	Asia	Southeast Asia	Cambodia	unknown	unknown	Wong et al, 2015
2010-019292	ERR360984	N/A	4.3.1.1	unknown	unknown	Blood	2012	Asia	Southeast Asia	Cambodia	unknown	unknown	Wong et al, 2015
01-2010-005164	ERR360985	N/A	4.3.1.1	unknown	unknown	Blood	2012	Asia	Southeast Asia	Cambodia	unknown	unknown	Wong et al, 2015
2012-012029	ERR360986	N/A	4.3.1.1	unknown	unknown	Blood	2012	Asia	Southeast Asia	Cambodia	unknown	unknown	Wong et al, 2015
2012-011846	ERR360987	N/A	4.3.1.1	unknown	unknown	Blood	2012	Asia	Southeast Asia	Cambodia	unknown	unknown	Wong et al, 2015
2012-012131	ERR360988	N/A	4.3.1.1	unknown	unknown	Blood	2012	Asia	Southeast Asia	Cambodia	unknown	unknown	Wong et al, 2015
2004-006350	ERR360990	N/A	4.3.1.1	unknown	unknown	Blood	2012	Asia	Southeast Asia	Cambodia	unknown	unknown	Wong et al, 2015
2002-213689	ERR360991	N/A	4.3.1.1	unknown	unknown	Blood	2012	Asia	Southeast Asia	Cambodia	unknown	unknown	Wong et al, 2015
2002-215815	ERR360992	N/A	4.3.1.1	unknown	unknown	Blood	2012	Asia	Southeast Asia	Cambodia	unknown	unknown	Wong et al, 2015
01-2011-005568	ERR360993	N/A	4.3.1.1	unknown	unknown	Blood	2012	Asia	Southeast Asia	Cambodia	unknown	unknown	Wong et al, 2015
01-2011-004759	ERR360994	N/A	4.3.1.1	unknown	unknown	Blood	2012	Asia	Southeast Asia	Cambodia	unknown	unknown	Wong et al, 2015
2012-014737	ERR360995	N/A	4.1	unknown	unknown	Blood	2012	Asia	Southeast Asia	Cambodia	unknown	unknown	Wong et al, 2015
2012-015146	ERR360996	N/A	4.3.1.1	unknown	unknown	Blood	2012	Asia	Southeast Asia	Cambodia	unknown	unknown	Wong et al, 2015

2012-019308	ERR360997	N/A	4.3.1.1	unknown	unknown	Blood	2012	Asia	Southeast Asia	Cambodia	unknown	unknown	Wong et al, 2015
2012-020127	ERR360998	N/A	4.3.1.1	unknown	unknown	Blood	2012	Asia	Southeast Asia	Cambodia	unknown	unknown	Wong et al, 2015
2011-008969	ERR360999	N/A	4.3.1.1	unknown	unknown	Blood	2012	Asia	Southeast Asia	Cambodia	unknown	unknown	Wong et al, 2015
2012-022646	ERR361000	N/A	4.3.1.1	unknown	unknown	Blood	2012	Asia	Southeast Asia	Cambodia	unknown	unknown	Wong et al, 2015
BCR43	ERR420413	N/A	4.3.1.2	unknown	unknown	Blood	2009	Asia	South Asia	India	unknown	unknown	Wong et al, 2015
BCR48	ERR420414	N/A	4.3.1.2	unknown	unknown	Blood	2009	Asia	South Asia	India	unknown	unknown	Wong et al, 2015
BCR49	ERR420415	N/A	4.3.1.1	unknown	unknown	Blood	2009	Asia	South Asia	India	unknown	unknown	Wong et al, 2015
BCR52	ERR420416	N/A	2.2.1	unknown	unknown	Blood	2009	Asia	South Asia	India	unknown	unknown	Wong et al, 2015
BCR62	ERR420417	N/A	4.3.1.2	unknown	unknown	Blood	2009	Asia	South Asia	India	unknown	unknown	Wong et al, 2015
BCR89	ERR420418	N/A	2.2.1	unknown	unknown	Blood	2010	Asia	South Asia	India	unknown	unknown	Wong et al, 2015
BCR108	ERR420420	N/A	4.3.1.2	unknown	unknown	Blood	2010	Asia	South Asia	India	unknown	unknown	Wong et al, 2015
BCR110	ERR420421	N/A	4.3.1.2	unknown	unknown	Blood	2010	Asia	South Asia	India	unknown	unknown	Wong et al, 2015
BCR162	ERR420422	N/A	4.3.1.2	unknown	unknown	Blood	2011	Asia	South Asia	India	unknown	unknown	Wong et al, 2015
BCR170	ERR420423	N/A	4.3.1.2	unknown	unknown	Blood	2011	Asia	South Asia	India	unknown	unknown	Wong et al, 2015
BCR175	ERR420424	N/A	4.3.1.2	unknown	unknown	Blood	2011	Asia	South Asia	India	unknown	unknown	Wong et al, 2015
BCR177	ERR420425	N/A	4.3.1.1	unknown	unknown	Blood	2011	Asia	South Asia	India	unknown	unknown	Wong et al, 2015
BCR191	ERR420426	N/A	4.3.1.2	unknown	unknown	Blood	2011	Asia	South Asia	India	unknown	unknown	Wong et al, 2015
BCR211	ERR420427	N/A	4.3.1.2	unknown	unknown	Blood	2011	Asia	South Asia	India	unknown	unknown	Wong et al, 2015
BCR232	ERR420428	N/A	2	unknown	unknown	Blood	2011	Asia	South Asia	India	unknown	unknown	Wong et al, 2015
SP2	ERR420429	N/A	4.3.1.1	unknown	unknown	Blood	2009	Asia	South Asia	India	unknown	unknown	Wong et al, 2015
SP9	ERR420430	N/A	4.3.1.2	unknown	unknown	Blood	2009	Asia	South Asia	India	unknown	unknown	Wong et al, 2015
SP11	ERR420431	N/A	4.3.1.2	unknown	unknown	Blood	2009	Asia	South Asia	India	unknown	unknown	Wong et al, 2015
SP47	ERR420432	N/A	4.3.1.1	unknown	unknown	Blood	2009	Asia	South Asia	India	unknown	unknown	Wong et al, 2015
SP66	ERR420433	N/A	3.3.2	unknown	unknown	Blood	2009	Asia	South Asia	India	unknown	unknown	Wong et al, 2015
SP80	ERR420434	N/A	4.3.1.2	unknown	unknown	Blood	2011	Asia	South Asia	India	unknown	unknown	Wong et al, 2015
H09176224	ERR422751	N/A	4.3.1.2	unknown	unknown	Blood	2009	Asia	South Asia	India	unknown	unknown	Wong et al, 2016
H09188293	ERR422752	N/A	4.3.1.1	unknown	unknown	Blood	2009	Asia	South Asia	Bangladesh	unknown	unknown	Wong et al, 2016
H0920834	ERR422754	N/A	3.3.2.Bd1	unknown	unknown	Blood	2009	Asia	South Asia	Bangladesh	unknown	unknown	Wong et al, 2016
H09266336	ERR422757	N/A	4.3.1	unknown	unknown	Blood	2009	Unknown	Unknown	Unknown	unknown	unknown	Wong et al, 2016
H09354568	ERR422759	N/A	4.3.1.1	unknown	unknown	Blood	2009	Asia	South Asia	India	unknown	unknown	Wong et al, 2016
H10044399	ERR422762	N/A	4.3.1.2	unknown	unknown	Blood	2010	Asia	South Asia	India	unknown	unknown	Wong et al, 2016
H10046338	ERR422764	N/A	4.3.1.3	unknown	unknown	Blood	2010	Unknown	Unknown	Unknown	unknown	unknown	Wong et al, 2016
H10048267	ERR422765	N/A	4.3.1.2	unknown	unknown	Blood	2010	Asia	South Asia	India	unknown	unknown	Wong et al, 2016
H10084431	ERR422766	N/A	4.3.1.1	unknown	unknown	Blood	2010	Asia	South Asia	Bangladesh	unknown	unknown	Wong et al, 2016
H10090344	ERR422767	N/A	3.3.2	unknown	unknown	Blood	2010	Asia	South Asia	Bangladesh, India	unknown	unknown	Wong et al, 2016
H10092333	ERR422768	N/A	3.3.2	unknown	unknown	Blood	2010	Asia	South Asia	Bangladesh, India	unknown	unknown	Wong et al, 2016
H10100338	ERR422769	N/A	3.3.2.Bd2	unknown	unknown	Blood	2010	Asia	South Asia	Bangladesh	unknown	unknown	Wong et al, 2016
H10106250	ERR422770	N/A	4.3.1.2	unknown	unknown	Blood	2010	Asia	South Asia	India	unknown	unknown	Wong et al, 2016
H10108318	ERR422771	N/A	3.3.2.Bd2	unknown	unknown	Blood	2010	Asia	South Asia	Bangladesh	unknown	unknown	Wong et al, 2016
H1011698	ERR422772	N/A	3.3.2.Bd2	unknown	unknown	Blood	2010	Asia	South Asia	Bangladesh	unknown	unknown	Wong et al, 2016
H10122493	ERR422773	N/A	3.3.2.Bd2	unknown	unknown	Blood	2010	Asia	South Asia	Bangladesh	unknown	unknown	Wong et al, 2016
H10134171	ERR422774	N/A	3.3.2.Bd2	unknown	unknown	Blood	2010	Asia	South Asia	Bangladesh	unknown	unknown	Wong et al, 2016
H10148201	ERR422775	N/A	4.3.1.1	unknown	unknown	Blood	2010	Asia	South Asia	Bangladesh	unknown	unknown	Wong et al, 2016
H1015074	ERR422776	N/A	4.3.1.1	unknown	unknown	Blood	2010	Asia	South Asia	Bangladesh	unknown	unknown	Wong et al, 2016
H10182335	ERR422778	N/A	3.3.1	unknown	unknown	Blood	2010	Unknown	Unknown	Unknown	unknown	unknown	Wong et al, 2016

H10184197	ERR422780	N/A	4.3.1	unknown	unknown	Blood	2010	Asia	South Asia	India	unknown	unknown	Wong et al, 2016
H10202409	ERR422781	N/A	4.3.1.1	unknown	unknown	Blood	2010	Asia	South Asia	Pakistan	unknown	unknown	Wong et al, 2016
H10294193	ERR422787	N/A	4.3.1.2	unknown	unknown	Blood	2010	Asia	South Asia	India	unknown	unknown	Wong et al, 2016
H10310204	ERR422788	N/A	2.3.3	unknown	unknown	Blood	2010	Asia	South Asia	Bangladesh	unknown	unknown	Wong et al, 2016
H10334602	ERR422790	N/A	2.1.7	unknown	unknown	Blood	2010	Asia	South Asia	India	unknown	unknown	Wong et al, 2016
H10340496	ERR422792	N/A	2.1.7	unknown	unknown	Blood	2010	Asia	South Asia	India	unknown	unknown	Wong et al, 2016
H10382491	ERR422796	N/A	4.3.1	unknown	unknown	Blood	2010	Unknown	Unknown	Unknown	unknown	unknown	Wong et al, 2016
H10394694	ERR422798	N/A	4.3.1	unknown	unknown	Blood	2010	Unknown	Unknown	Unknown	unknown	unknown	Wong et al, 2016
H10432531	ERR422800	N/A	4.3.1.1	unknown	unknown	Blood	2010	Asia	South Asia	Bangladesh	unknown	unknown	Wong et al, 2016
H10462591	ERR422801	N/A	4.3.1.1	unknown	unknown	Blood	2010	Asia	South Asia	Bangladesh	unknown	unknown	Wong et al, 2016
H11044442	ERR422803	N/A	4.3.1	unknown	unknown	Blood	2011	Asia	South Asia	Pakistan	unknown	unknown	Wong et al, 2016
H11054345	ERR422804	N/A	4.3.1.2	unknown	unknown	Blood	2011	Asia	South Asia	India	unknown	unknown	Wong et al, 2016
H11096403	ERR422805	N/A	4.3.1.1	unknown	unknown	Blood	2011	Asia	South Asia	Pakistan	unknown	unknown	Wong et al, 2016
H11150244	ERR422807	N/A	4.3.1.3	unknown	unknown	Blood	2011	Asia	South Asia	Bangladesh	unknown	unknown	Wong et al, 2016
H11194353	ERR422810	N/A	4.3.1.1	unknown	unknown	Blood	2011	Asia	South Asia	Bangladesh	unknown	unknown	Wong et al, 2016
H11244554	ERR422811	N/A	2.3.3	unknown	unknown	Blood	2011	Asia	South Asia	Bangladesh	unknown	unknown	Wong et al, 2016
H11254619	ERR422812	N/A	4.3.1.1	unknown	unknown	Blood	2011	Asia	South Asia	Bangladesh	unknown	unknown	Wong et al, 2016
H11344588	ERR422813	N/A	4.3.1.1	unknown	unknown	Blood	2011	Asia	South Asia	Bangladesh	unknown	unknown	Wong et al, 2016
H11354680	ERR422814	N/A	4.3.1.2	unknown	unknown	Blood	2011	Asia	South Asia	India	unknown	unknown	Wong et al, 2016
H11372597	ERR422815	N/A	4.3.1.1	unknown	unknown	Blood	2011	Asia	South Asia	Bangladesh	unknown	unknown	Wong et al, 2016
H11372598	ERR422816	N/A	4.3.1	unknown	unknown	Blood	2011	Unknown	Unknown	Unknown	unknown	unknown	Wong et al, 2016
H11374579	ERR422817	N/A	4.3.1.1	unknown	unknown	Blood	2011	Asia	South Asia	India	unknown	unknown	Wong et al, 2016
H11388492	ERR422818	N/A	4.3.1.2	unknown	unknown	Blood	2011	Asia	South Asia	Bangladesh	unknown	unknown	Wong et al, 2016
H11420359	ERR422819	N/A	4.3.1	unknown	unknown	Blood	2011	Asia	South Asia	India	unknown	unknown	Wong et al, 2016
H11424491	ERR422820	N/A	3.2.2	unknown	unknown	Blood	2011	Asia	South Asia	Bangladesh	unknown	unknown	Wong et al, 2016
H11436323	ERR422822	N/A	3.3.2.Bd1	unknown	unknown	Blood	2011	Asia	South Asia	Bangladesh	unknown	unknown	Wong et al, 2016
H12014687	ERR422824	N/A	4.3.1.1	unknown	unknown	Blood	2011	Asia	South Asia	Bangladesh	unknown	unknown	Wong et al, 2016
H12054632	ERR422826	N/A	4.3.1.3.Bdq	unknown	unknown	Blood	2012	Asia	South Asia	Bangladesh	unknown	unknown	Wong et al, 2016
H121541053	ERR422828	N/A	4.3.1	unknown	unknown	Blood	2012	Asia	South Asia	India	unknown	unknown	Wong et al, 2016
H12164448	ERR422829	N/A	4.3.1.1	unknown	unknown	Blood	2012	Asia	South Asia	Bangladesh	unknown	unknown	Wong et al, 2016
H12200559	ERR422830	N/A	2.0.1	unknown	unknown	Blood	2012	Asia	South Asia	Bangladesh	unknown	unknown	Wong et al, 2016
H12276630	ERR422831	N/A	4.3.1.1	unknown	unknown	Blood	2012	Asia	South Asia	Bangladesh	unknown	unknown	Wong et al, 2016
H12282824	ERR422832	N/A	3.2.2	unknown	unknown	Blood	2012	Asia	South Asia	Pakistan	unknown	unknown	Wong et al, 2016
H12382595	ERR422833	N/A	4.3.1	unknown	unknown	Blood	2012	Asia	South Asia	Pakistan	unknown	unknown	Wong et al, 2016
H12414817	ERR422834	N/A	4.3.1	unknown	unknown	Blood	2012	Asia	South Asia	Pakistan	unknown	unknown	Wong et al, 2016
H05196408	ERR485127	N/A	4.3.1	unknown	unknown	Blood	2005	Unknown	Unknown	Unknown	unknown	unknown	Wong et al, 2016
H05212226	ERR485129	N/A	4.3.1	unknown	unknown	Blood	2005	Unknown	Unknown	Unknown	unknown	unknown	Wong et al, 2016
H06136379	ERR485132	N/A	4.3.1.3	unknown	unknown	Blood	2006	Unknown	Unknown	Unknown	unknown	unknown	Wong et al, 2016
H06136380	ERR485133	N/A	4.3.1.3	unknown	unknown	Blood	2006	Unknown	Unknown	Unknown	unknown	unknown	Wong et al, 2016
H06414500	ERR485138	N/A	4.3.1.1	unknown	unknown	Blood	2006	Asia	South Asia	Bangladesh	unknown	unknown	Wong et al, 2016
H06448472	ERR485139	N/A	4.1	unknown	unknown	Blood	2006	Asia	South Asia	India	unknown	unknown	Wong et al, 2016
H07188291	ERR485140	N/A	3.3.2.Bd2	unknown	unknown	Blood	2007	Asia	South Asia	Bangladesh	unknown	unknown	Wong et al, 2016
H08248234	ERR485145	N/A	3.3.2.Bd2	unknown	unknown	Blood	2008	Asia	South Asia	Bangladesh	unknown	unknown	Wong et al, 2016
H09176223	ERR485147	N/A	3.3.2.Bd2	unknown	unknown	Blood	2009	Unknown	Unknown	Unknown	unknown	unknown	Wong et al, 2016

Table 1 citations: Mashe et al, 2021 (1), Wong et al, 2015 (2), Wong et al, 2016 (3), Ingle et al, 2019 (4)

References

1. Mashe T, Leekitcharoenphon P, Mtapuri-Zinyowera S, Kingsley RA, Robertson V, Tarupiwa A, et al. Salmonella enterica serovar Typhi H58 clone has been endemic in Zimbabwe from 2012 to 2019. J Antimicrob Chemother. 2021;76(5):1160-7.
2. Wong VK, Baker S, Pickard DJ, Parkhill J, Page AJ, Feasey NA, et al. Phylogeographical analysis of the dominant multidrug-resistant H58 clade of Salmonella Typhi identifies inter- and intracontinental transmission events. Nat Genet. 2015;47(6):632-9.
3. Wong VK, Baker S, Connor TR, Pickard D, Page AJ, Dave J, et al. An extended genotyping framework for Salmonella enterica serovar Typhi, the cause of human typhoid. Nat Commun. 2016;7:12827.
4. Ingle DJ, Nair S, Hartman H, Ashton PM, Dyson ZA, Day M, et al. Informal genomic surveillance of regional distribution of Salmonella Typhi genotypes and antimicrobial resistance via returning travellers. PLoS Negl Trop Dis. 2019;13(9):e0007620.
